# Subtle Stereochemical Effects Influence Binding and Purification Abilities of an Fe^II^_4_L_4_ Cage

**DOI:** 10.1021/jacs.3c00294

**Published:** 2023-02-27

**Authors:** Weichao Xue, Luca Pesce, Adinarayana Bellamkonda, Tanya K. Ronson, Kai Wu, Dawei Zhang, Nicolas Vanthuyne, Thierry Brotin, Alexandre Martinez, Giovanni M. Pavan, Jonathan R. Nitschke

**Affiliations:** † Yusuf Hamied Department of Chemistry, University of Cambridge, Cambridge CB2 1EW, U.K.; ‡ Department of Innovative Technologies, University of Applied Sciences and Arts of Southern Switzerland, CH-6962 Lugano-Viganello, Switzerland; § Shanghai Key Laboratory of Green Chemistry and Chemical Processes, School of Chemistry and Molecular Engineering, East China Normal University, Shanghai 200062, China; ∥ Aix Marseille Université, Centrale Marseille, CNRS, iSm2 UMR 7313, 13397 Marseille, France; ⊥ Laboratoire de Chimie, Université Lyon, Ens de Lyon, CNRS UMR 5182, Lyon F69342, France; # Department of Applied Science and Techology, Politecnico di Torino, 10129 Torino, Italy

## Abstract

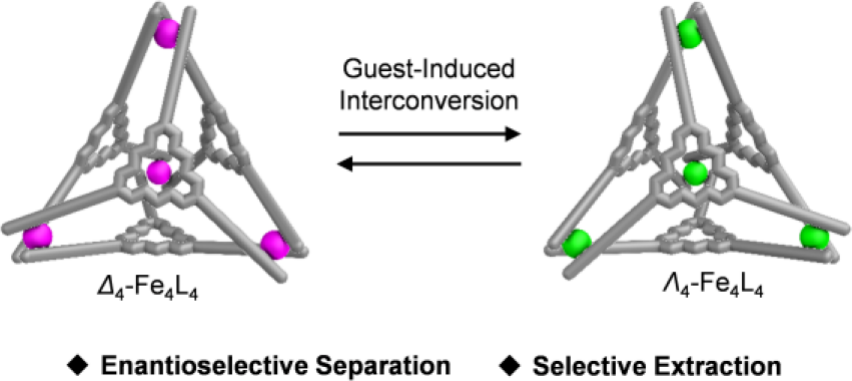

A tetrahedral Fe^II^_4_L_4_ cage assembled from the coordination of triangular chiral, face-capping ligands to iron(II). This cage exists as two diastereomers in solution, which differ in the stereochemistry of their metal vertices, but share the same point chirality of the ligand. The equilibrium between these cage diastereomers was subtly perturbed by guest binding. This perturbation from equilibrium correlated with the size and shape fit of the guest within the host; insight as to the interplay between stereochemistry and fit was provided by atomistic well-tempered metadynamics simulations. The understanding thus gained as to the stereochemical impact on guest binding enabled the design of a straightforward process for the resolution of the enantiomers of a racemic guest.

## Introduction

Enzymes possess chirotopic cavities, enabling stereoselective recognition of target substrates and stereospecific chemical reactions.^1−3^ Enantiopure metal–organic cages with enclosed cavities,^4^ constructed by coordination-driven self-assembly, are able to mimic the functions of enzymes and have found uses across diverse areas, including stereoselective sensing, separation,^5^ and catalysis.^6,7^ Studies on the communication of stereochemistry within cages also help to elucidate the flow of stereochemical information in both artificial and living systems and may lead to the discovery of bioinspired applications.^8^

The stereochemistry of metal–organic cages can be influenced by enantiopure counterions and guests, through templation during cage formation or postassembly resolution of racemic cage mixtures.^4,9^ More frequently, enantiopure components, i.e., ligands and metal complexes, are used to control the stereochemistry of self-assembled structures, whereby the resulting metal–organic cages are enantiopure.^4,10^ In cases where the metal ions, particularly those from the d-block or f-block, have octahedral^11^ or pseudotricapped trigonal prismatic geometry,^12^ stereochemical information from the ligands can transfer to the metal vertices to produce either a preferred Δ or Λ handedness during higher-order self-assembly. Based upon this strategy, examples of the diastereoselective formation of homochiral cages, with precise control of the handedness of both metal vertices and the final assembled structure, have been reported.^4,11,12^

Metal–organic cages with electron-deficient walls have displayed extensive host–guest properties, binding electron-rich and even electron-poor guests with high affinities.^13^ We therefore envisioned that the incorporation of an electron-poor ligand into a chiral cage framework might optimize binding ability,^14^ resulting in the discovery of self-assembled cages with new potential applications.^15^ Herein, we describe the self-assembly of an electron-deficient enantiopure ligand with Fe^II^ to afford an Fe^II^_4_L_4_ tetrahedron existing as a pair of distinct diastereomers, adopting either an all Δ or Λ configuration of metal centers, with moderate diastereocontrol. The ratio of the Δ_4_ to Λ_4_ configurations was then subtly modulated and even inverted by the encapsulation of guests. The diastereoenriched cage enabled the selective encapsulation of functionalized fullerenes from mixtures and enantioselective separation of racemic cryptophane-A (CRY-A).

## Results and Discussion

**Figure 1:**
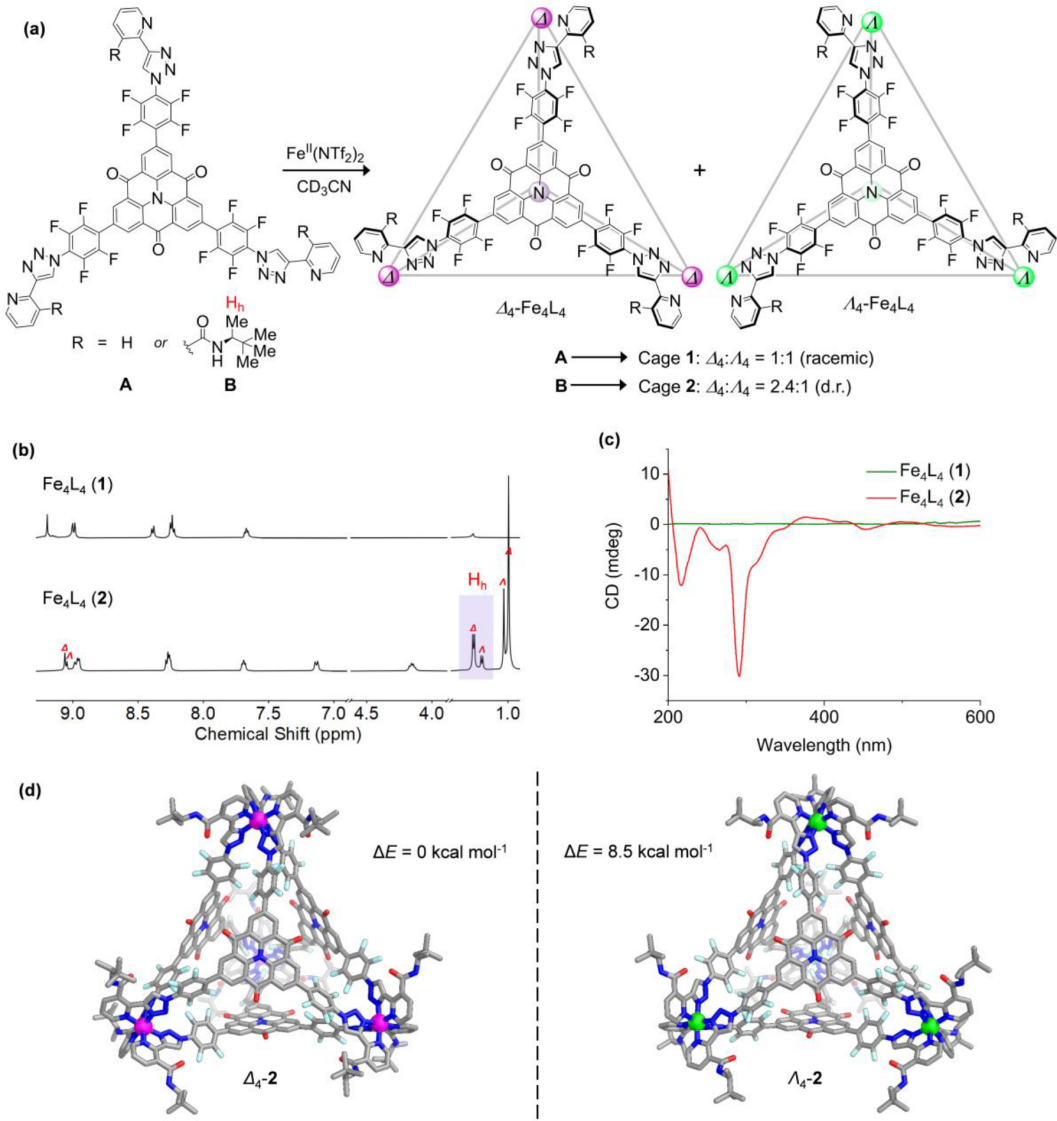
(a) Self-assembly of cages **1** and **2** from ligands **A** and **B**, respectively. (b) Partial ^1^H NMR spectra of cages **1** and **2**, with H_h_ used to gauge the d.r. of **2** (500 MHz, CD_3_CN, 298 K). (c) CD spectra of cages **1** and **2**. (d) Front view of the DFT-optimized molecular models of Δ_4_-**2** and Λ_4_-**2**, with Δ*E* representing the difference in total energy between the two diastereomers at 298 K as estimated by molecular dynamics.

Tritopic ligand **A** with pyridyl-triazole “click” chelates^14,16^ was synthesized from iodinated *N*-heterotriangulene over three steps (Figure S1). The carbonyl groups and perfluorophenyl rings ensure its electron-deficient nature. The self-assembly of **A** (4 equiv) with iron(II) bis(trifluoromethanesulfonyl)imide (Fe(NTf_2_)_2_, 4 equiv) in acetonitrile at 343 K gave rise to cage **1** (Figure 1a). Its Fe^II^_4_L_4_ composition, as anticipated following the foundational work of Lusby,^16^ was confirmed by electrospray ionization mass spectrometry (ESI-MS, Figure S21). One set of proton signals in the ^1^H NMR spectrum of **1** indicated the exclusive formation of a *T*-symmetric framework, with each octahedral tris-chelate metal vertex displaying the same handedness (Figure 1b). The absence of Cotton effects in the circular dichroism (CD) spectrum was consistent with the formation of a racemic mixture of Δ_4_-**1** and Λ_4_-**1** in solution (Figure 1c).

We hypothesized that the stereochemistry of such Fe^II^_4_L_4_ cages might be controlled by using an enantiopure ligand, which could dictate the configuration of the iron centers. Chiral ligand **B**, having the same ligand core as **A**, was therefore prepared, with each arm bearing an amide-containing chiral directing group (Figure S7). The stereocenter-containing side chain was incorporated at the 3-position of the pyridyl ring to secure its proximity to the metal vertex. Such a design should also avoid steric clash within the coordination environment around the metal centers that would be induced by substituents at the pyridyl 6-position, which might destabilize assembled structures.^17^

Ligand **B** underwent self-assembly with Fe(NTf_2_)_2_ to produce Fe^II^_4_L_4_ cage **2** (Figure 1a), as confirmed by ESI-MS (Figure S30). The ^1^H NMR spectrum of cage **2** shows two sets of proton signals, consistent with the formation of Δ_4_-**2** and Λ_4_-**2** as diastereomeric complexes. The well-separated signals of H_h_ allowed determination of a diastereomeric ratio (d.r.) of 2.4:1 (Figure 1b). The same diffusion coefficient was observed for all peaks in the diffusion-ordered spectroscopy (DOSY) spectrum, indicating similar hydrodynamic radii for both diastereomers (Figure S25). Other *N*-heterotriangulene-based chiral ligands bearing modified chiral directing groups were also employed in the self-assembly process; however, lower diastereomeric ratios were observed in all cases compared to the present ratio of 2.4:1 (Figures S34 and S35).

The CD spectrum of cage **2** displayed intense negative signals around 240–340 nm, corresponding to high-energy π–π* transitions in the ligands, while metal-to-ligand charge transfer (MLCT) and d–d transitions produced weaker signals from 360 to 540 nm (Figure 1c). The Cotton effects observed in the CD spectrum are correlated with the handedness of the octahedral tris-chelate iron vertices. Comparison of the CD spectra of structurally similar Δ*-* and Λ*-*[Fe(bpy)_3_]^2+^ complexes, particularly peaks resulting from π–π* and MLCT transitions, allowed us to infer there is an excess of iron centers having Δ configuration within cage **2**.^18,19^ The major diastereomer of **2** was thus determined to be Δ_4_-**2**, and the minor diastereomer was Λ_4_-**2**.

After many unsuccessful attempts to grow crystals suitable for X-ray diffraction, we undertook density functional theory (DFT) calculations to obtain the energy-minimized molecular models of Δ_4_-**2** and Λ_4_-**2** (Figure 1d; for details, see Supporting Information Section 10).^20^ In accordance with previous observations of face-capped M_4_L_4_ tetrahedral cages,^5i,9h^ Δ_4_-**2** adopts a clockwise orientation of its four ligand faces, while Λ_4_-**2** is paired with ligands of anticlockwise orientation. The Fe^II^···Fe^II^ distances in both diastereomers are similar (ca. 23 Å). The calculated cavity volumes are only slightly different: 1281 Å^3^ for Δ_4_-**2** and 1266 Å^3^ for Λ_4_-**2** (Figure S107).^21^

In control experiments, an Fe^II^L_3_ complex was formed by the reaction of Fe(NTf_2_)_2_ with a monomeric pyridyl-triazole ligand bearing the same chiral side chain (Figure S36). Very weak signals observed in the CD spectrum indicated a weaker chiral induction effect in this mononuclear complex relative to that of the tetranuclear cage (Figure S38). These results reflect that diastereoselectivity during the formation of **2** emerges as a result of higher-order assembly, in which the stereochemical information transfer from ligand to metal vertex and stereochemical communication between metal centers may cooperatively play a role in amplifying the energy differences between the two diastereomers.^22^

The relative energy differences (Δ*E*) between Δ_4_-**2** and Λ_4_-**2** was gauged to be 8.5 kcal mol^–1^ by performing molecular dynamics simulations on a model of cage **2** in explicit acetonitrile at 298 K performed using the GROMACS software package patched with plumed^23^ (model description and simulation setup in Supporting Information Section 10).^24^ These calculations supported the conclusion that Δ_4_-**2** is more favored from an enthalpic point of view, as this difference mainly arises from the difference in potential energy.

**Figure 2:**
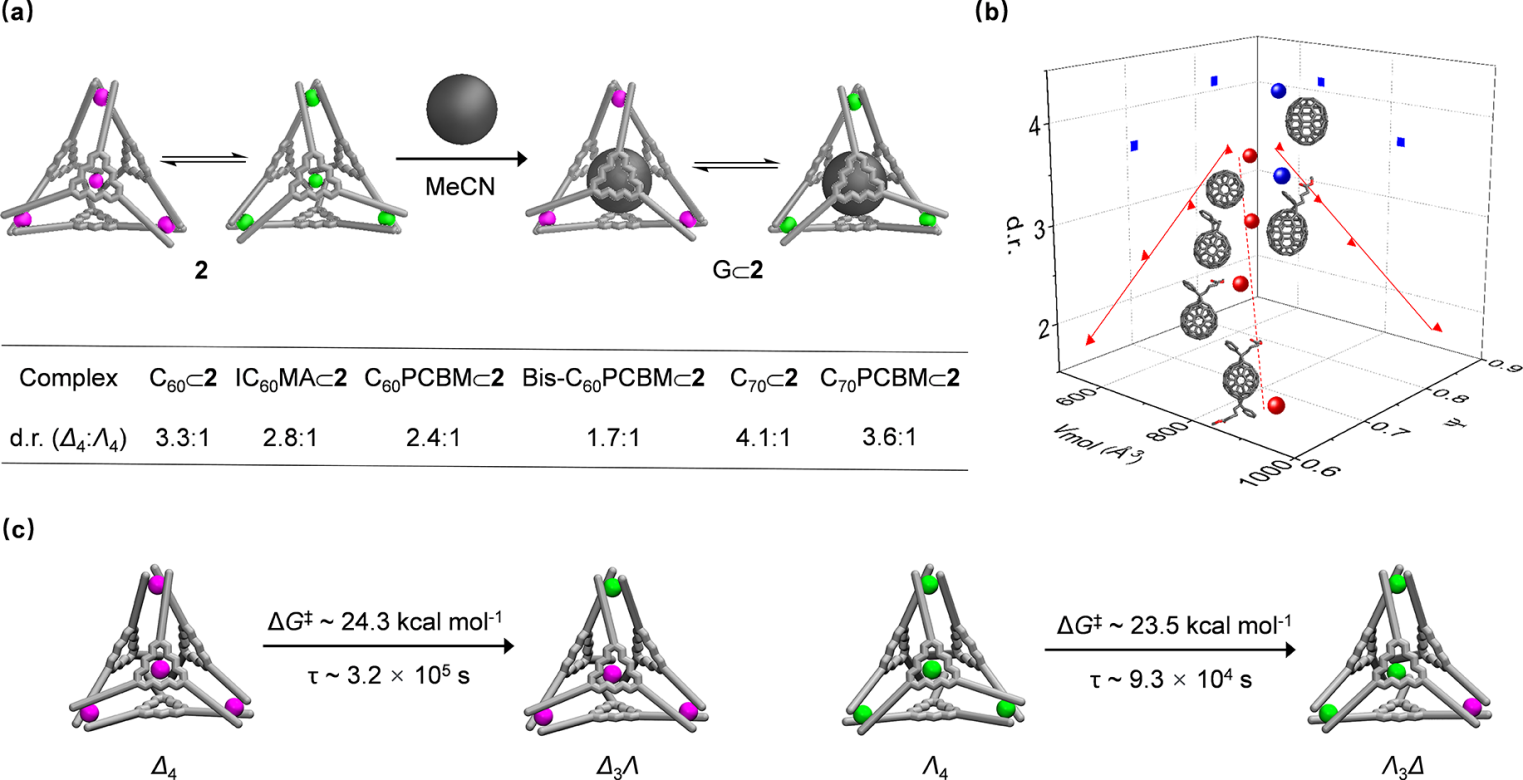
(a) Schematic and table showing guest-binding-induced Δ_4_ ⇄ Λ_4_ interconversion, with d.r. determined by ^1^H NMR. (b) Diastereomeric ratio of the host–guest complex plotted against the molecular volume (*V*_mol_) and sphericity (Ψ) of guests. PCBM = [6,6]-phenyl-C_*n*_-butyric acid methyl ester (*n* = 61 or 71). IC_60_MA = indene-C_60_ monoadduct. Bis-C_60_PCBM exists as a mixture of regioisomers. C_70_PCBM exists as a mixture of regioisomers with about 85% α-C_70_PCBM. (c) Schematic showing conversion of one metal vertex within the framework of **2**, with Δ*G*^⧧^ representing the calculated transition energy barrier and τ representing the characteristic transition timescale at 298 K.

Both diastereomers of **2** have flat ligand cores and enclosed cavities, thus rendering **2** a good prospective host for large π-extended guests. We therefore began to investigate the host–guest properties of **2** with fullerenes and fullerene derivatives (Figure 2a). Heating an equimolar mixture of guest and **2** in acetonitrile at 343 K for 30 min resulted in the quantitative formation of the 1:1 host–guest complexes **G**⊂**2**, as confirmed by ^1^H NMR, ^19^F NMR, and ESI-MS spectra for all investigated guests (Supporting Information, Section 6.1). The major contributions to binding were inferred to be extensive stacking interactions between host and guest, as well as solvophobic effects in acetonitrile. The insolubility of these π-extended guests in acetonitrile prevented quantification of binding strength through ^1^H NMR titration experiments.

^1^H NMR and CD spectra confirmed that both Δ_4_-**2** and Λ_4_-**2** were able to accommodate the guests, with **G**⊂Δ_4_-**2** as the major species (Figures 2a and S69); Δ_4_⇄Λ_4_ interconversion was also observed during the binding process. We inferred that the size and shape fit between guest and cage change the energy differences between two configurations. To quantify this phenomenon, molecular volume (*V*_mol_)^21^ was used to determine the size of the guest, while sphericity (Ψ)^25^ was employed to reflect the shape of the guest considering the near-spherical cavity of **2** (Table S2). The plot of d.r. against *V*_mol_ and Ψ revealed that binding a smaller and more spherical guest resulted in a stronger energetic preference for the Δ_4_ configuration, whereas binding larger and less spherical guests reduced energy differences between diastereomers (Figure 2b). Although the stereochemical effects of guests upon the host observed here were subtle, the model plotted in Figure 2b allowed quantification of guest-induced diastereomer interconversion for binding C_60_ and its adducts, according to the linear relationships between d.r. and *V*_mol_ and between d.r. and Ψ. However, two outlying points prevented good linear regression fits for binding C_70_ and C_70_PCBM ([6,6]-phenyl-C_71_-butyric acid methyl ester).

To obtain information related to the energy barriers for the interconversion between Δ_4_-**2** and Λ_4_-**2**, we employed multiple infrequent well-tempered metadynamics (WT-MetaD) simulations.^26^ These biased simulations allowed us to activate the escape from the two local Δ and Λ minima and to obtain information on the associated barriers and characteristic timescales expected for these transitions in unbiased conditions. In particular, we activated the transition of one of the four metal vertices, exploring the Δ_4_ → Δ_3_Λ and Λ_4_ → Λ_3_Δ transitions (Figure 2c), which are first necessary steps in the Δ_4_ ⇄ Λ_4_ isomerization. Fifty infrequent WT-MetaD simulations were run for both transitions.

For the Δ_4_ → Δ_3_Λ transition, the infrequent WT-MetaD simulations provided a transition energy barrier Δ*G*^⧧^ ∼ 24.3 kcal mol^–1^ and a characteristic transition timescale τ ∼ 3.2 × 10^5^ s, while the corresponding energy barrier and timescale for Λ_4_ → Λ_3_Δ were calculated to be Δ*G*^⧧^ ∼ 23.5 kcal mol^–1^ and τ ∼ 9.3 × 10^4^ s, respectively. These results suggested that the dynamics of interconversion between Δ_4_ and Λ_4_ diastereomers are slow at room temperature. Similar energy barriers were obtained for the diastereomer transformations of C_70_⊂**2**. However, by rescaling the obtained transition timescales at 343 K (mixing condition), we could estimate that these transition events can occur within a timescale of minutes. This suggests that the Δ_4_ and Λ_4_ configurations dynamically equilibrate during the self-assembly process and reequilibrate during mixing with guest molecules.

**Figure 3:**
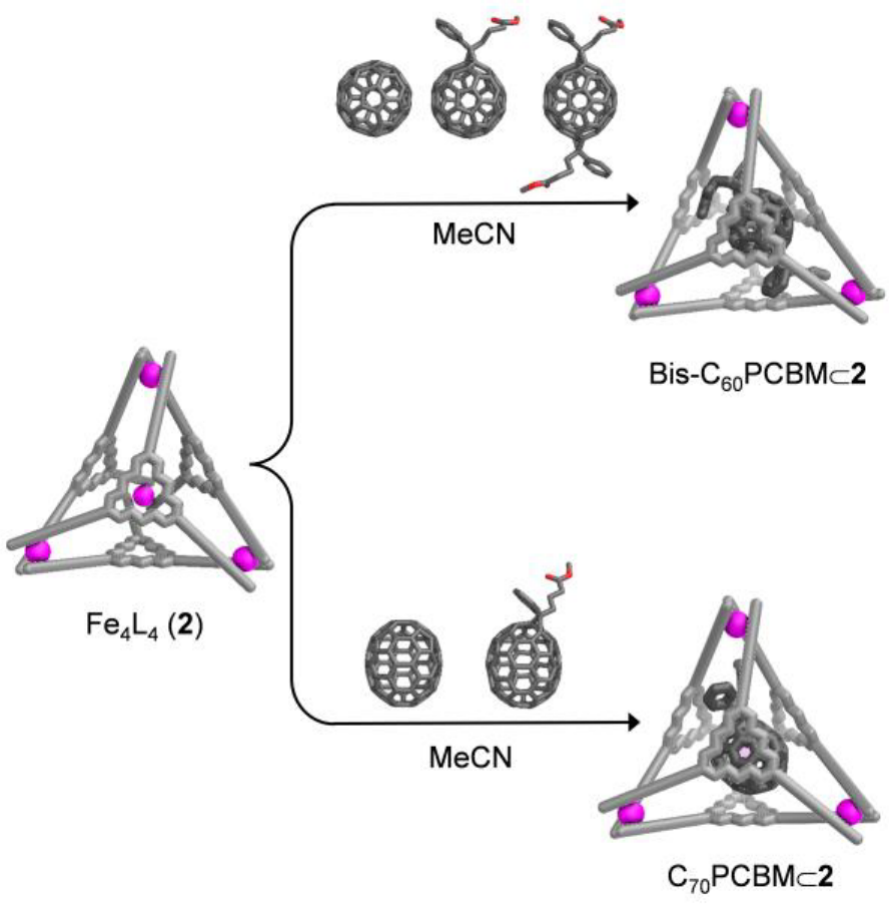
Schematic showing the selective encapsulation of bis-C_60_PCBM and C_70_PCBM.

We then investigated the ability of **2** to purify high-value fullerenes, starting with preparing a mixture consisting of equimolar amounts of C_60_, C_60_PCBM, bis-C_60_PCBM, and **2** in acetonitrile (Figure 3). Notably, after being kept at 343 K for 30 min, the host–guest complex bis-C_60_PCBM⊂**2** was observed to form exclusively, as confirmed by ESI-MS and ^1^H NMR (Figures S89 and S90). Likewise, cage **2** was also able to selectively extract C_70_PCBM from a mixture with C_70_ (Figures S91 and S92). The efficient and selective encapsulation of bis-C_60_PCBM and C_70_PCBM by **2** may provide an alternative method for the purification of fullerene covalent adducts contaminated with numerous side-products from reaction mixtures.^27^ We attribute the excellent selectivity observed here to the higher solubility of these alkyl chain-substituted fullerenes in organic solvents.^28^

**Figure 4:**
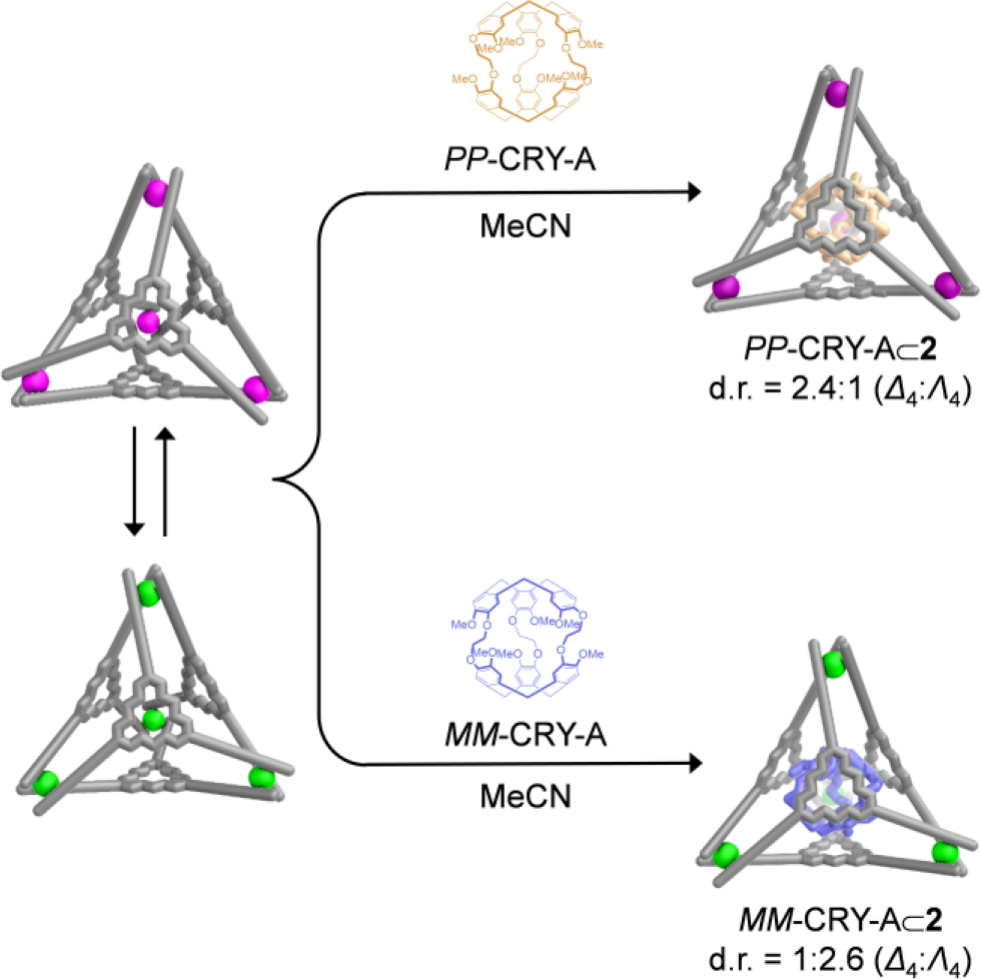
Schematic showing the stereochemical communication between **2** and CRY-A.

Electron-deficient **2** was also observed to bind electron-rich enantiopure cryptophane-A, which is an example of an important class of organic supramolecular host.^29^ The host–guest adduct CRY-A⊂**2** was formed upon heating equimolar amounts of CRY-A and **2** in acetonitrile at 343 K for 30 min (Figure 4). *PP*-CRY-A⊂**2** (Δ_4_:Λ_4_ = 2.4:1) retained the stereochemical configuration of the parent cage **2** (Δ_4_:Λ_4_ = 2.4:1), whereas the encapsulation of *MM*-CRY-A occurred with inversion of host stereochemistry, providing *MM*-CRY-A⊂**2** in a d.r. of Δ_4_:Λ_4_ = 1:2.6. Opposite Cotton effects observed in the CD spectra of both host–guest complexes also confirmed such stereochemical outcomes upon encapsulation of enantiopure CRY-A (Figure S94). The Δ_4_ configuration was thus favored by *PP*-CRY-A, whereas the Λ_4_ configuration was preferred by *MM*-CRY-A. The inversion of the stereochemistry of **2** induced by *MM*-CRY-A reflected that host **2** can dynamically adapt its stereochemistry and chiral inner void to maximize binding affinity for a chiral guest.

**Figure 5:**
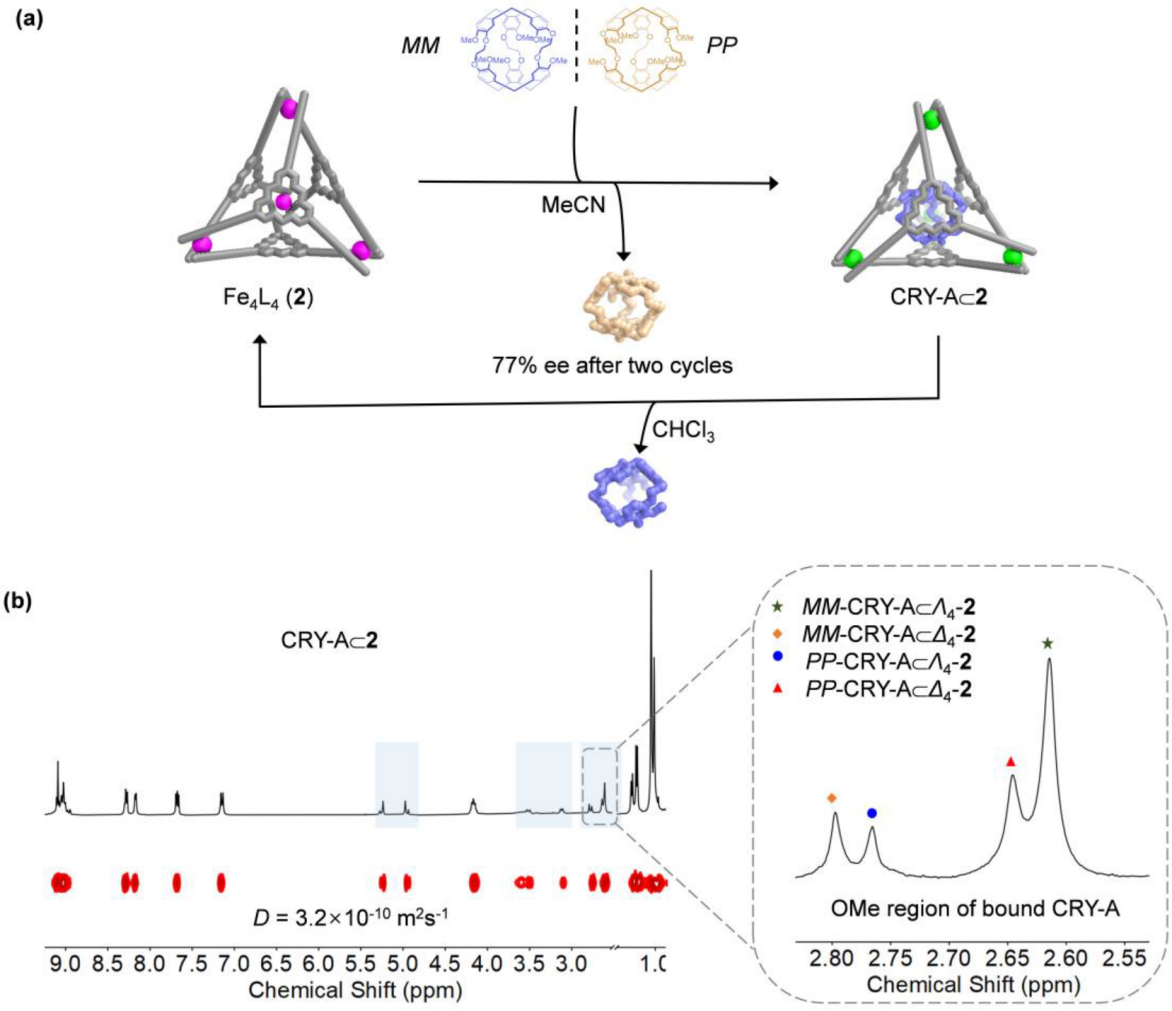
(a) Schematic showing the enantioselective resolution of CRY-A by cage **2** in acetonitrile and the recycling of **2** in chloroform. (b) Partial ^1^H NMR spectrum of CRY-A⊂**2** obtained in the first round of the resolution procedure, with the peaks for the encapsulated guest highlighted with a light blue background, showing an expansion of the OMe region of bound CRY-A (400 MHz, CD_3_CN, 298 K).

We next explored the enantioselective separation of racemic guests by **2**. We observed that **2** displayed no enantioselectivity in binding racemic C_70_PCBM, as confirmed by ^1^H NMR and CD spectra (Figures S65 and S105). Diastereoenriched **2** was nonetheless capable of enantioselectively separating racemic CRY-A (Figure 5a).

Two equivalents of racemic CRY-A were added to an acetonitrile solution of **2**, and the reaction mixture was maintained at 343 K for 30 min. The host–guest complex CRY-A⊂**2** was isolated by precipitation with diethyl ether; evaporating the excess diethyl ether subsequently afforded unbound CRY-A. All signals in the ^1^H NMR spectrum of CRY-A⊂**2** had the same diffusion coefficient, with proton signals corresponding to bound CRY-A shifted upfield due to host shielding effects (Figure 5b). Four sets of signals from the methoxy groups of CRY-A were observed in the 2.55–2.83 ppm region, indicating that CRY-A⊂**2** consists of four diastereomers, *PP*-CRY-A⊂Δ_4_-**2**, *PP*-CRY-A⊂Λ_4_-**2**, *MM*-CRY-A⊂Δ_4_-**2**, and *MM*-CRY-A⊂Λ_4_-**2**. Comparison with the ^1^H NMR spectra for *PP*-CRY-A⊂**2** and *MM*-CRY-A⊂**2** allowed us to identify each diastereomer in solution (Figure S93).

The ^1^H NMR spectrum clearly showed that more *MM*-CRY-A was encapsulated when two equivalents of racemic guest were used. Cotton effects assigned to *MM*-CRY-A were also observed in the CD spectrum of CRY-A⊂**2** (Figure S94). The enantiomeric excess (ee) of the unbound CRY-A was determined to be 32% by chiral HPLC, with *PP*-CRY-A being enriched (Figure S96). The bound CRY-A was released by sonicating a suspension of the host–guest complex in chloroform (Figure S102), and the solid **2** was then recovered by centrifugation (Figures S103 and S104).

In control experiments, *PP*-CRY-A was observed to be encapsulated kinetically faster than *MM*-CRY-A at the initial stage of the binding process, as *PP*-CRY-A is bound more strongly by the Δ_4_ configuration of **2** (Δ_4_:Λ_4_ = 2.4:1 for this guest). Heating the reaction mixture resulted in re-equilibration of the cage framework, eventually giving *MM*-CRY-A⊂Λ_4_-**2** as the major host–guest complex, indicating that *MM*-CRY-A⊂Λ_4_-**2** is the thermodynamically favored diastereomer within the four-diastereomer CRY-A⊂**2** system (Figure S101). These results indicated that the enantioselectivity in binding racemic CRY-A by **2** is driven by the formation of the thermodynamically stable host–guest diastereomer, *MM*-CRY-A⊂Λ_4_-**2**.

Encouraged by the chiral resolution observed, we ran a second round of separation experiments, through addition of the CRY-A (32% ee) obtained from the first round to a fresh acetonitrile solution of **2**. After precipitation of the host–guest adduct and removal of the solvent, the unbound CRY-A was obtained in 77% ee, as determined by chiral HPLC (Figure S98).

## Conclusions

The moderately stereoselective self-assembly of Fe^II^_4_L_4_ tetrahedron **2** thus can serve as the basis of an enantioseparation process, based upon a nuanced understanding of how stereochemistry influences guest fit within this host. Cage **2** was also capable of selectively extracting bis-C_60_PCBM and C_70_PCBM from mixtures of their derivatives. The strategy outlined herein may thus become applicable to the design of new cage-based purification methods, particularly stereoselective ones. Future work will focus on the immobilization of such *N*-heterotriangulene-based cages on solid supports, such as alumina, for the development of efficient large-scale separation and purification processes.^30^

## Data Availability

Structure and parameters of the computational models used in atomistic molecular dynamics are available at 10.5281/zenodo.7350671 (ref (24)).

## Supporting Information Available

Experimental procedures; NMR characterizations; mass spectrometry data; CD spectra; volume calculations; HPLC data; computational model parametrization; model simulation protocol (PDF)
