# Supplementary material for: Subtle Stereochemical Effects Influence Binding and Purification Abilities of an FeII4L4 Cage
Source: J Am Chem Soc. 2023 Feb 27;145(9):5570–7. doi: 10.1021/jacs.3c00294 (PMC9999408; doi:10.1021/jacs.3c00294)
Supplement: Supplementary file 1 — ja3c00294_si_001.pdf [file ja3c00294_si_001.pdf]

Subtle Stereochemical Effects Influence Binding and Purification Abilities of an $Fe^{II}_4L_4$ Cage

Weichao Xue,[1] Luca Pesce,[2] B. Adinarayana,[1] Tanya K. Ronson,[1] Kai Wu,[1] Dawei Zhang,[3] Nicolas Vanthuyne,[4] Thierry Brotin,[5] Alexandre Martinez,[4] Giovanni M. Pavan,*[,2,6] Jonathan R. Nitschke*[,1]

[1]Yusuf Hamied Department of Chemistry, University of Cambridge, Cambridge CB2 1EW, U.K.

[2]Department of Innovative Technologies, University of Applied Sciences and Arts of Southern Switzerland, CH-6962 Lugano-Viganello, Switzerland

[3]Shanghai Key Laboratory of Green Chemistry and Chemical Processes, School of Chemistry and Molecular Engineering, East China Normal University, Shanghai 200062, China

[4]Aix Marseille University, CNRS, Centrale Marseille, iSm2, Marseille, France

[5]Laboratoire de chimie, Université Lyon, Ens de Lyon, CNRS UMR 5182, Lyon F69342, France

[6]Department of Applied Science and Techology, Politecnico di Torino, 10129 Torino, Italy

Supporting Information

Table of Contents

1 General Information S3

2 Synthesis and Characterization of Ligands **A** and **B** S5

3 Self-Assembly and Characterization of Cages **1** and **2** S13

3.1 Self-Assembly of Cage **1** in MeCN S13

3.2 Self-Assembly of Cage **2** in MeCN S19

4 Investigation of Temperatures and Chiral Side Chains S26

4.1 Investigation of Temperatures S26
4.2 Investigation of Chiral Side Chains S27
5 Investigation of the Monomeric $Fe^{II}L_3$ Complex S29
6 Host-Guest Studies Using Cage **2** S31
6.1 Encapsulation of Fullerenes and Fullerene Adducts S31
6.2 Encapsulation of Enantiopure Cryptophane-A S54
7 Selective Encapsulation of Functionalized Fullerenes S67
8 Enantioselective Separation of Racemic Cryptophane-A S72
9 Volume Calculations S84
10 Computational Studies S85
11 References S106

1 General Information

Unless otherwise specified, all reagents were purchased from commercial sources and used as received. C_{60} (Alfa Aesar); C_{70} (Alfa Aesar); C_{60}PCBM (Ossila); bis-C_{60}PCBM (Ossila); C_{70}PCBM (Ossila); carbon nanobelt (TCI); enantiopure 2-ethynylpyridine derivative (**S4**) was prepared from commercially available 2-bromonicotinic acid according to a reported procedure;[1] precursor **S1** was prepared from iodinated *N*-heterotriangulene according to a reported procedure[2] and $Fe(NTf_2)_2 \cdot 4.5H_2O$ was prepared according to a reported procedure.[3] Self-assembly reactions were performed in either CD_3CN or distilled MeCN.

NMR spectra were recorded using the following NMR spectrometers: Bruker 400 MHz Avance III HD smart probe (^{1}H, ^{13}C, ^{19}F, ^{1}H-DOSY, and 2D NMR), Bruker 500 MHz AVIII HD Smart Probe (^{1}H, ^{1}H-DOSY and ^{19}F), Bruker Avance 500 MHz DCH cryoprobe (^{1}H, ^{13}C, and 2D NMR), Bruker Avance 500 MHz TCI cryoprobe (^{1}H NMR) or 700 MHz TXO Cryoprobe (^{1}H NMR). Chemical shifts of the NMR spectra are reported relative to $CDCl_3$ (^{1}H NMR: δ = 7.26 ppm, ^{13}C NMR: δ = 77.0 ppm), CD_3CN (^{1}H NMR: δ = 1.94 ppm, ^{13}C NMR: δ = 118.3 ppm). Data for ^{1}H NMR spectra were reported as follows: chemical shift (ppm), peak shape (s = singlet, d = doublet, t = triplet, m = multiplet), coupling constant (Hz), and integration. Data for ^{13}C NMR are reported with chemical shift (ppm) values referenced to the residual solvent peak; data for ^{19}F NMR are reported with chemical shift (ppm) values as observed.

UV-vis measurements were employed to fine-tune the solution concentration for subsequent CD measurements, and were performed on a Varian Cary 400 scan UV-vis spectrophotometer with a 1 mm path-length cuvette at 25 °C. Circular Dichroism was performed on an Applied-Photophysics Chirascan CD spectrometer using a 1 mm path-length cuvette. Experiments were recorded at 298 K, maintained with a Peltier temperature control. Measurements were background subtracted from blank solvent in an identical cuvette. The sample concentrations were adjusted to maintain a HV below 800 V.

Low resolution electrospray ionization mass spectrometry was undertaken on a Micromass Quattro LC mass spectrometer (cone voltage 20-50 eV; desolvation temp. 40 °C; ionization temp. 40 °C) infused from a Harvard syringe pump at a rate of 10 µL/min. High resolution electrospray ionisation mass spectra (ESI–HRMS) were recorded on a Waters Synapt G2-Si instrument.

The ee values for cryptophane-A were determined on an Agilent by HPLC on chiral support with an Agilent 1260 Infinity unit (pump G1311B, autosampler G1329B, DAD G1315D), with Igloo-Cil ovens, monitored by SRA Instruments Seleccol software (Version 1.2.3.0) and Agilent OpenLAB Chemstation. Chiroptical detection was obtained with CD-2095, circular dichroism detector. The analytical column (250 x 4.6 mm) used is Chiralpak ID from Chiral Technology Europa (Illkirch, France).

2 Synthesis and Characterization of Ligands A and B

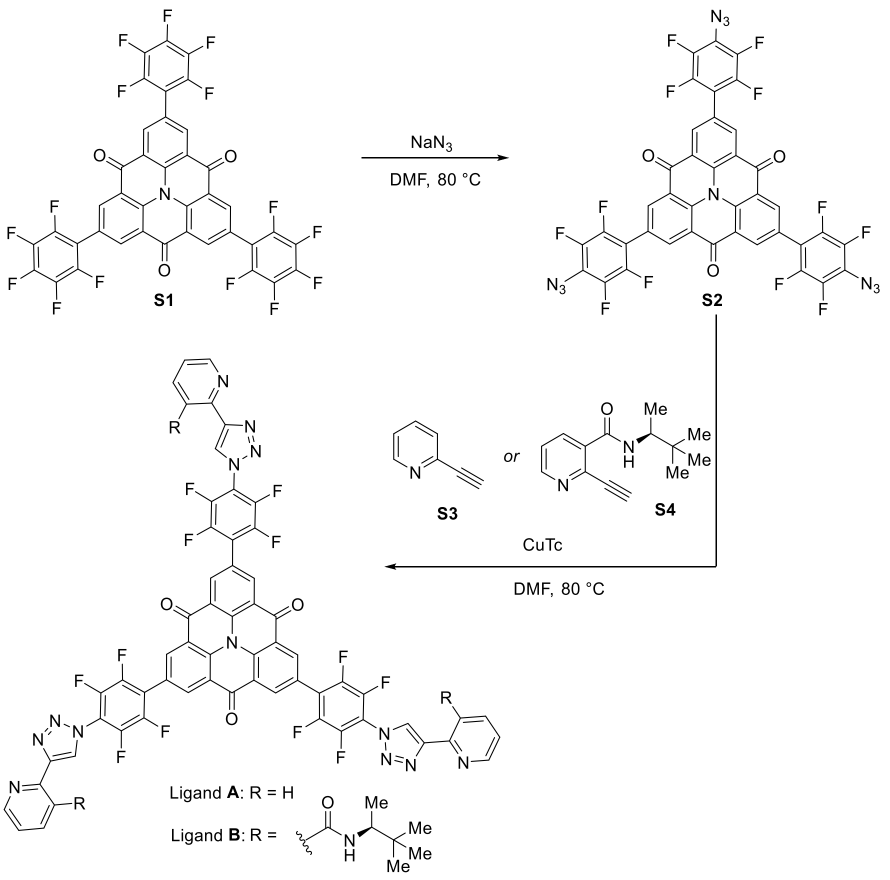

A suspension of **S1** (2.46 g, 3.0 mmol, 1.0 equiv) and NaN_3 (780 mg, 12.0 mmol, 4.0 equiv) in dimethylformamide (DMF, 30 mL) was heated at 80 °C for 16 hours. After cooling down to room temperature, excess DMF was evaporated under reduced pressure. The dark solid was transferred to a Büchner funnel and isolated by vacuum filtration, and then washed with H_2O (500 mL). After drying under reduced pressure, crude compound **S2** was obtained as a yellow solid (1.75 g, 66% yield) and used without purification.

To a solution of **S2** (267 mg, 0.3 mmol, 1.0 equiv) in DMF (15 mL) was added copper(I) thiophene-2-carboxylate (CuTc, 25.7 mg, 0.135 mmol, 0.45 equiv) and 2-ethynylpyridine **S3** (124 mg, 1.2 mmol, 4.0 equiv). The reaction mixture was heated at 80 °C for 16 hours under nitrogen. After cooling down to room temperature, excess DMF was evaporated under reduced pressure. The residual solid was directly loaded onto a chromatography column filled with silica gel. Purification by flash column

chromatography using EtOAc/hexane= 60/40 as eluent afforded ligand **A** as a brown solid (162 mg, 45%).

R_f = 0.60 (EtOAc/hexane = 60/40). **^{1}H NMR** (500 MHz, $CDCl_3$): δ 7.30–7.36 (m, 3H), 7.87 (td, *J* = 7.7, 1.8 Hz, 3H), 8.30 (d, *J* = 7.7 Hz, 3H), 8.59 (s, 3H), 8.67 (d, *J* = 4.1 Hz, 3H), 9.26 (s, 6H) ppm. **^{13}C NMR** (126 MHz, $CDCl_3$): δ 117.0–117.5 (m, C_6F_4), 118.8–119.3 (m, C_6F_4), 120.7, 123.6, 124.1, 124.3, 124.9, 136.4, 137.1, 138.1, 140.8–141.2 (m, C_6F_4), 142.9–143.2 (m, C_6F_4), 143.3–143.6 (m, C_6F_4), 145.2–145.7 (m, C_6F_4), 148.9, 149.2, 149.7, 175.0 ppm. **^{19}F NMR** (470 MHz, $CDCl_3$): δ -140.96 (dd, *J* = 20.4, 8.7 Hz, 6F), -145.22 (dd, *J* = 20.4, 8.7 Hz, 6F) ppm.

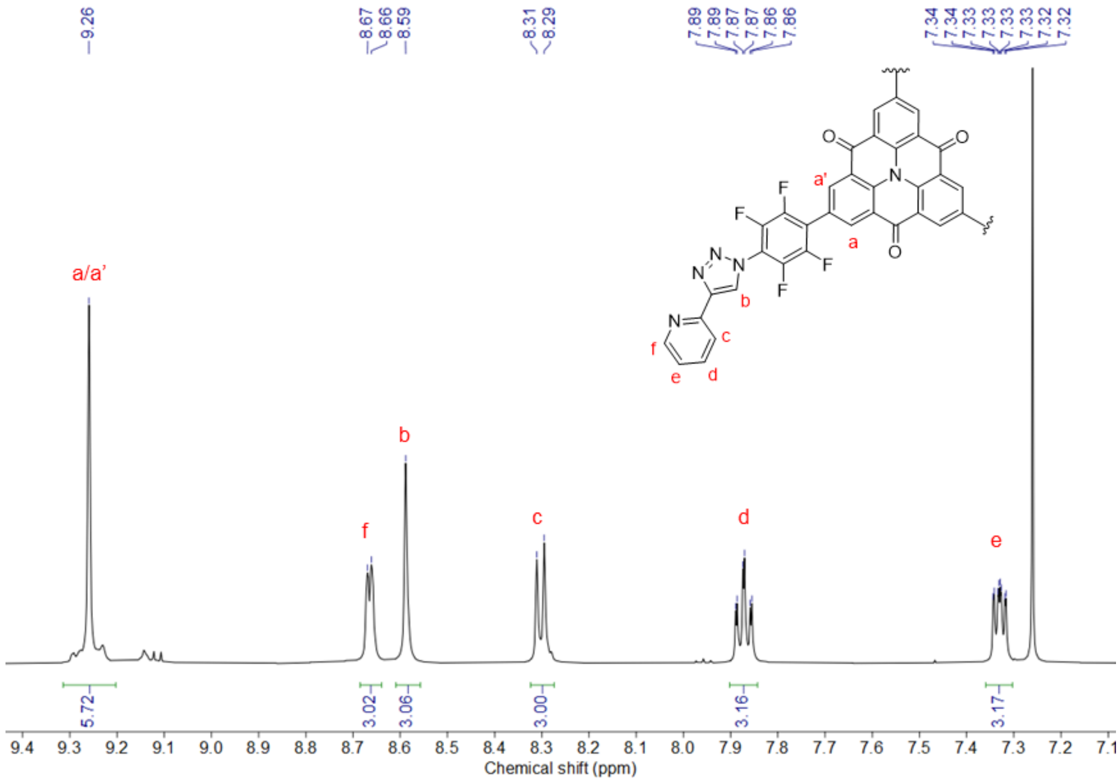

Figure S1. ^{1}H NMR spectrum of ligand **A** (500 MHz, $CDCl_3$, 25 °C).

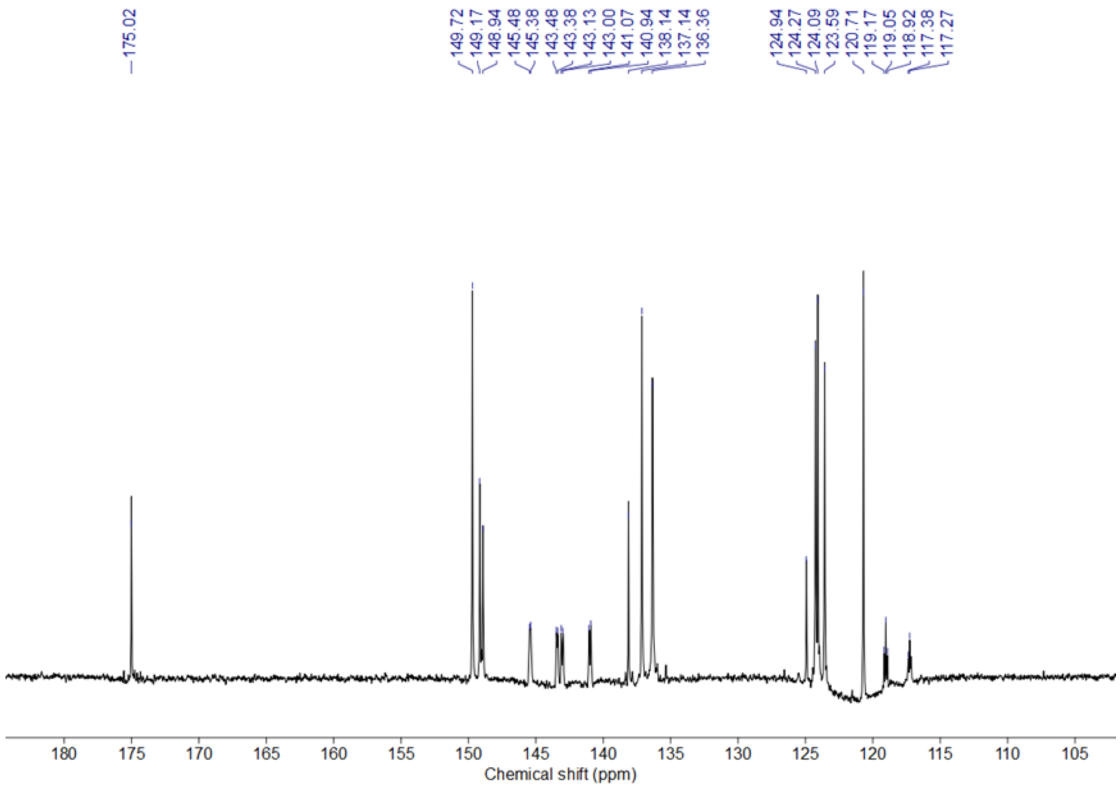

Figure S2. ^{13}C NMR spectrum of ligand **A** (126 MHz, $CDCl_3$, 25 °C).

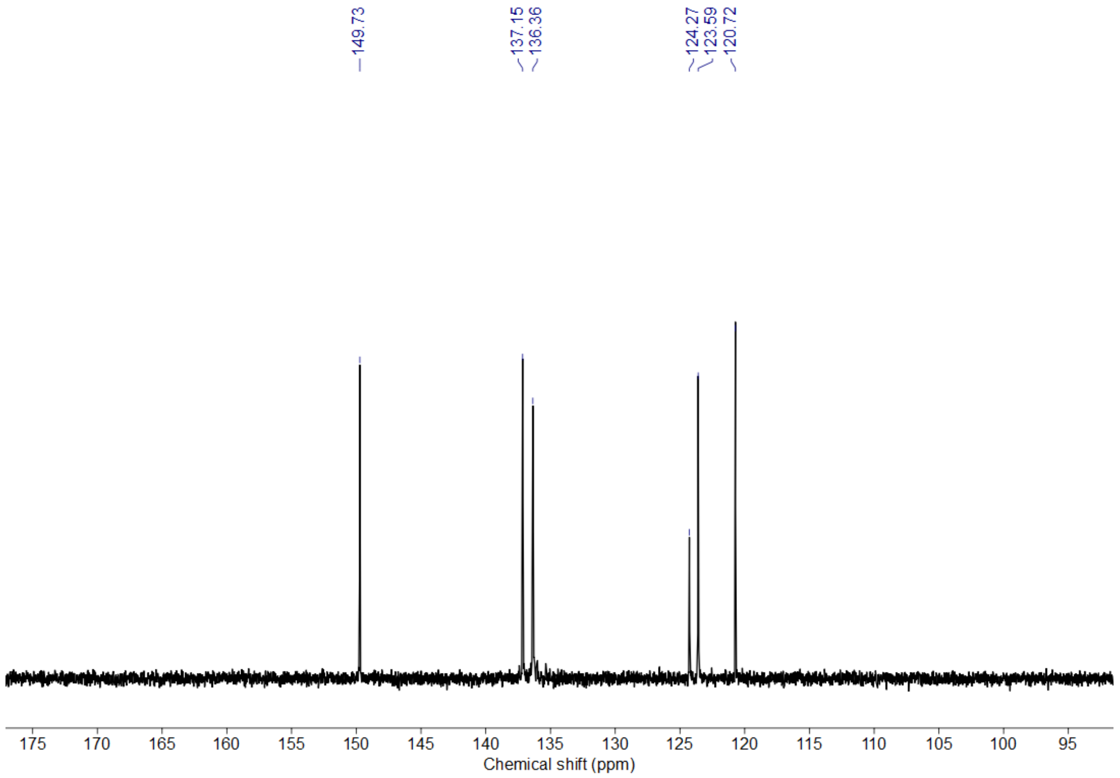

Figure S3. ^{13}C DEPT-135 NMR spectrum of ligand **A** (126 MHz, $CDCl_3$, 25 °C).

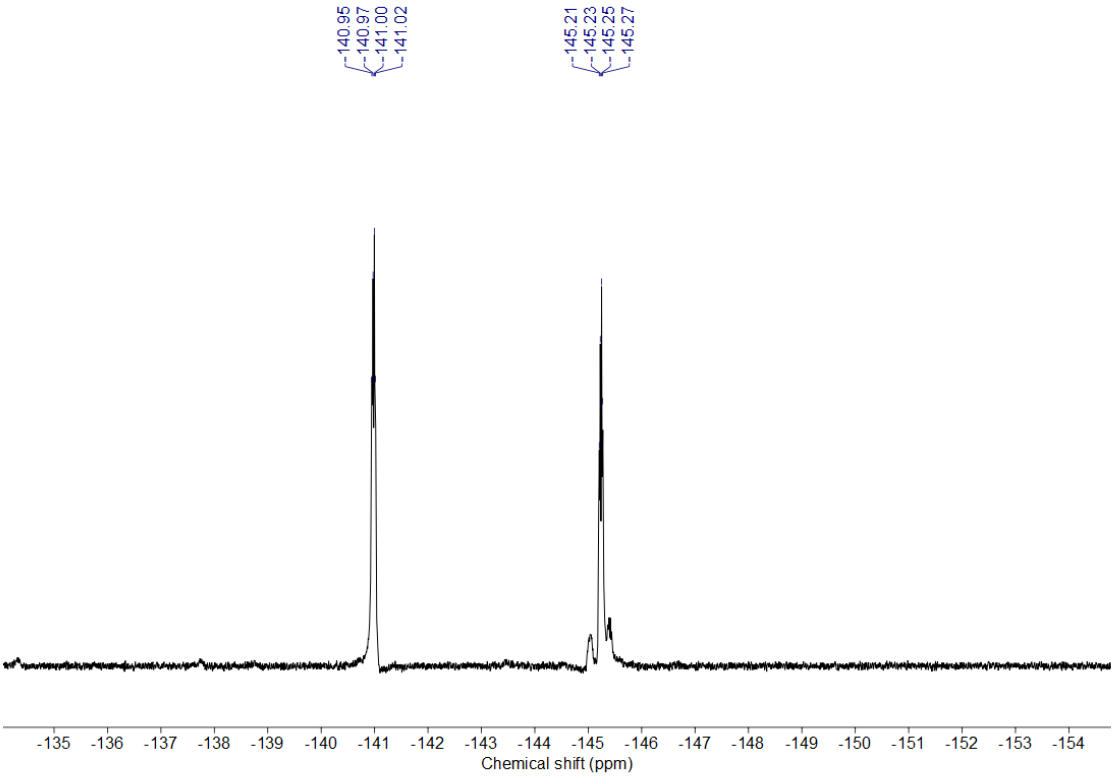

Figure S4. ^{19}F NMR spectrum of ligand **A** (470 MHz, $CDCl_3$, 25 °C).

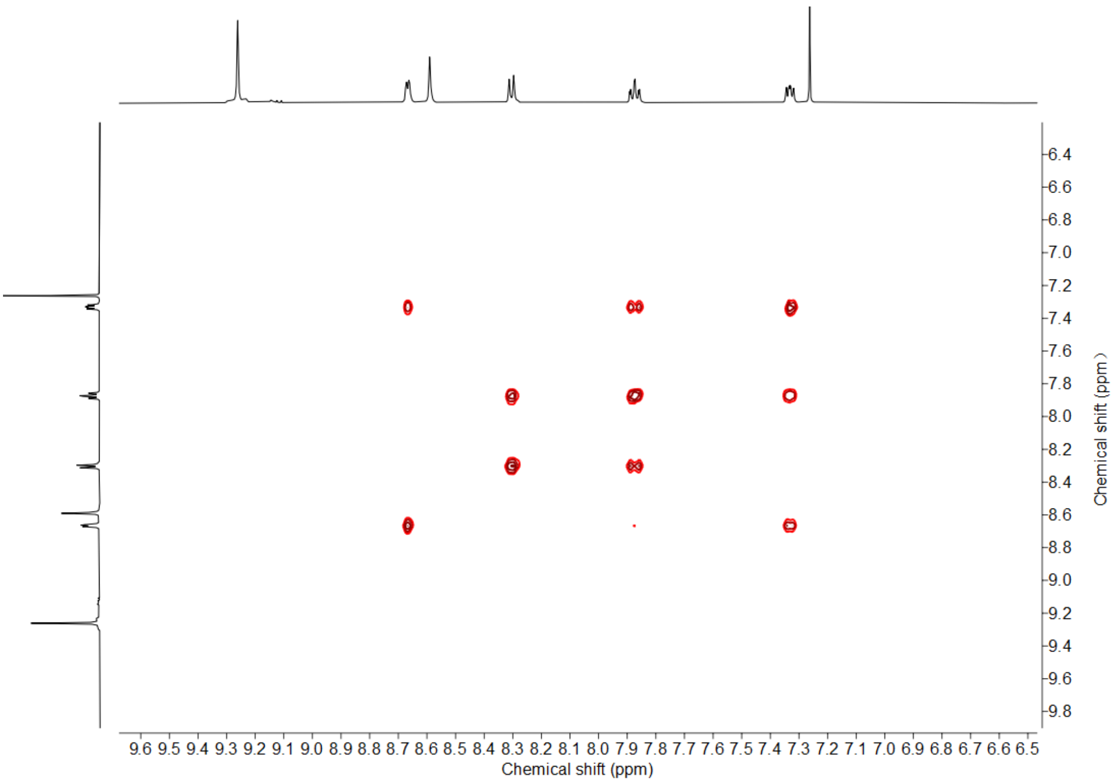

Figure S5. ^{1}H-^{1}H COSY NMR spectrum of ligand **A** (500 MHz, $CDCl_3$, 25 °C).

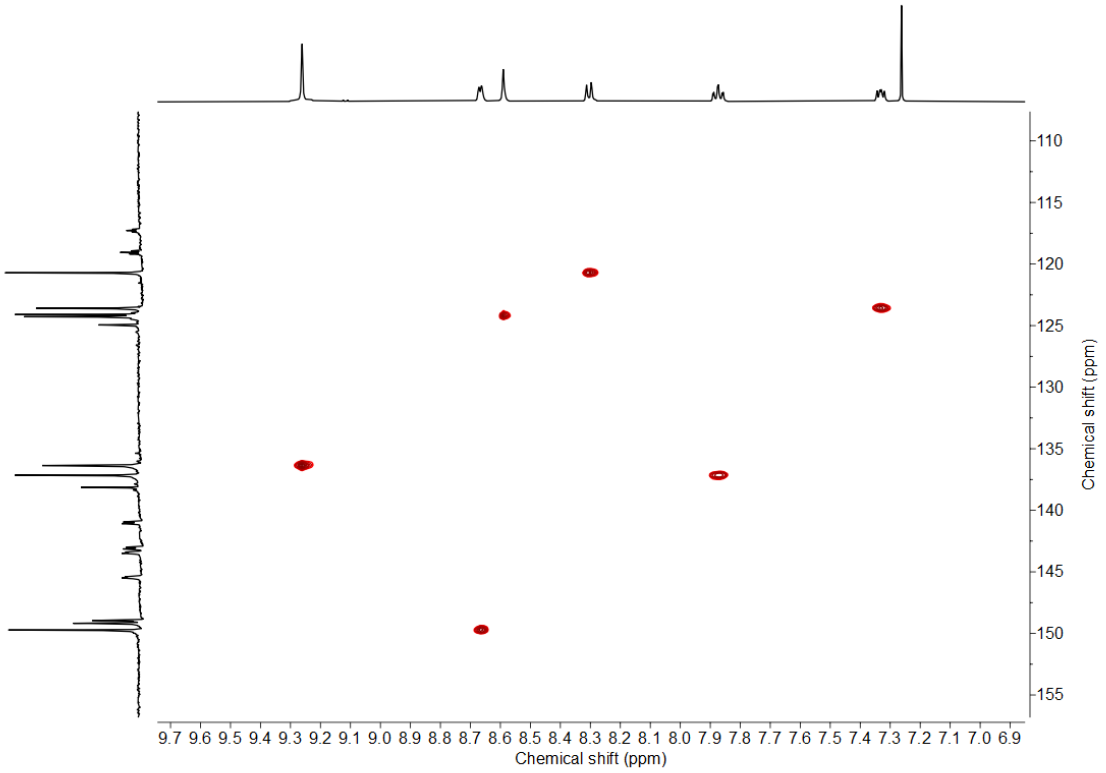

Figure S6. ^{1}H-^{13}C HSQC NMR spectrum of ligand **A** (500 MHz, $CDCl_3$, 25 °C).

To a solution of **S2** (267 mg, 0.3 mmol, 1.0 equiv) in DMF (15 mL) was added copper(I) thiophene-2-carboxylate (CuTc, 25.7 mg, 0.135 mmol, 0.45 equiv) and 2-ethynylpyridine derivative **S4** (276 mg, 1.2 mmol, 4.0 equiv). The reaction mixture was heated at 80 °C for 16 hours under nitrogen. After cooling down to room temperature, excess DMF was evaporated under reduced pressure. The residual solid was directly loaded onto a chromatography column filled with silica gel. Purification by flash column chromatography using EtOAc/MeOH = 50/1 as eluent afforded ligand **B** as a light brown solid (190 mg, 40%).

$\mathbf{R}_f$ = 0.35 (EtOAc/MeOH = 20/1). **^{1}H NMR** (500 MHz, $CDCl_3$): δ 0.96 (s, 27H), 1.19 (d, *J* = 6.8 Hz, 9H), 4.05–4.16 (m, 3H), 6.39 (d, *J* = 9.6 Hz, 3H), 7.34–7.45 (m, 3H), 7.96 (d, *J* = 7.4 Hz, 3H), 8.53 (s, 3H), 8.76 (s, 3H), 9.22 (s, 6H) ppm. **^{13}C NMR** (126 MHz, $CDCl_3$): δ 15.6, 26.2, 34.2, 54.1, 116.7–117.2 (m, C_6F_4), 118.9–119.2 (m, C_6F_4), 123.2, 124.1, 124.9, 126.4, 132.6, 136.3, 136.8, 138.1, 140.8–141.1 (m, C_6F_4), 142.9–143.2 (m, C_6F_4), 143.3–143.5 (m, C_6F_4), 145.1, 145.2–145.6 (m, C_6F_4), 147.2, 150.5, 167.2,

175.0 ppm. **^{19}F NMR** (470 MHz, $CDCl_3$): δ -140.91 (dd, *J* = 20.4, 8.7 Hz, 6F), -144.99 (dd, *J* = 20.4, 8.7 Hz, 6F) ppm.

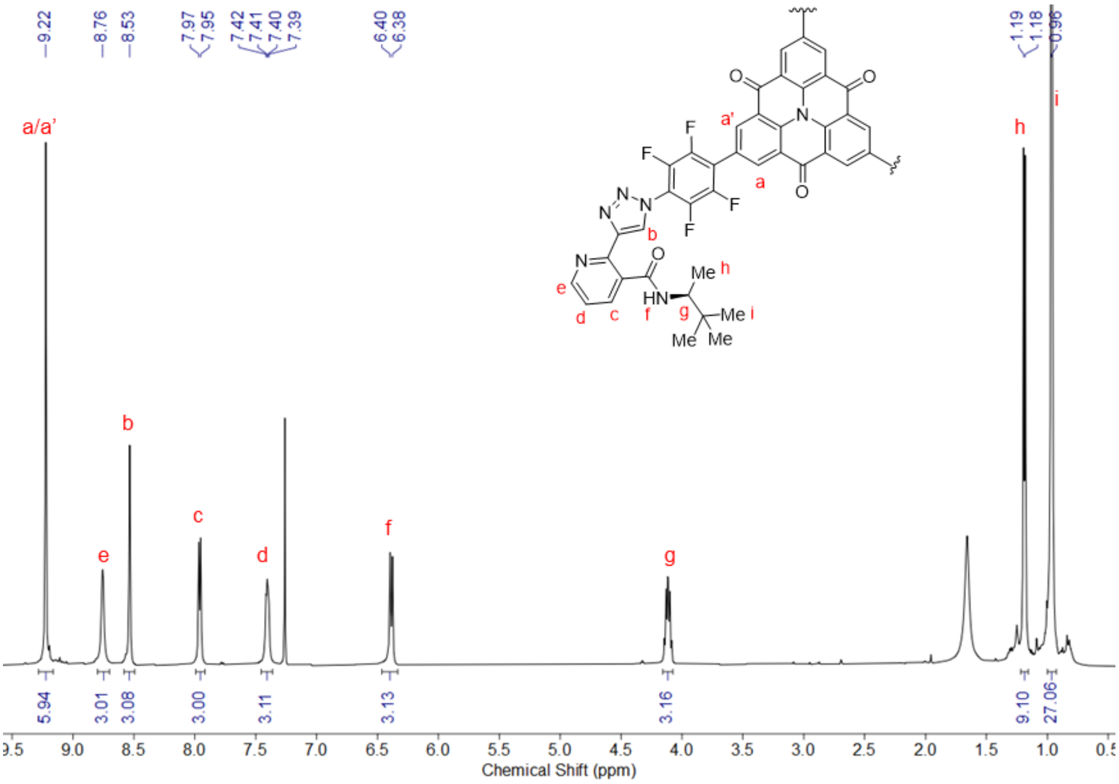

Figure S7. ^{1}H NMR spectrum of ligand **A** (500 MHz, $CDCl_3$, 25 °C).

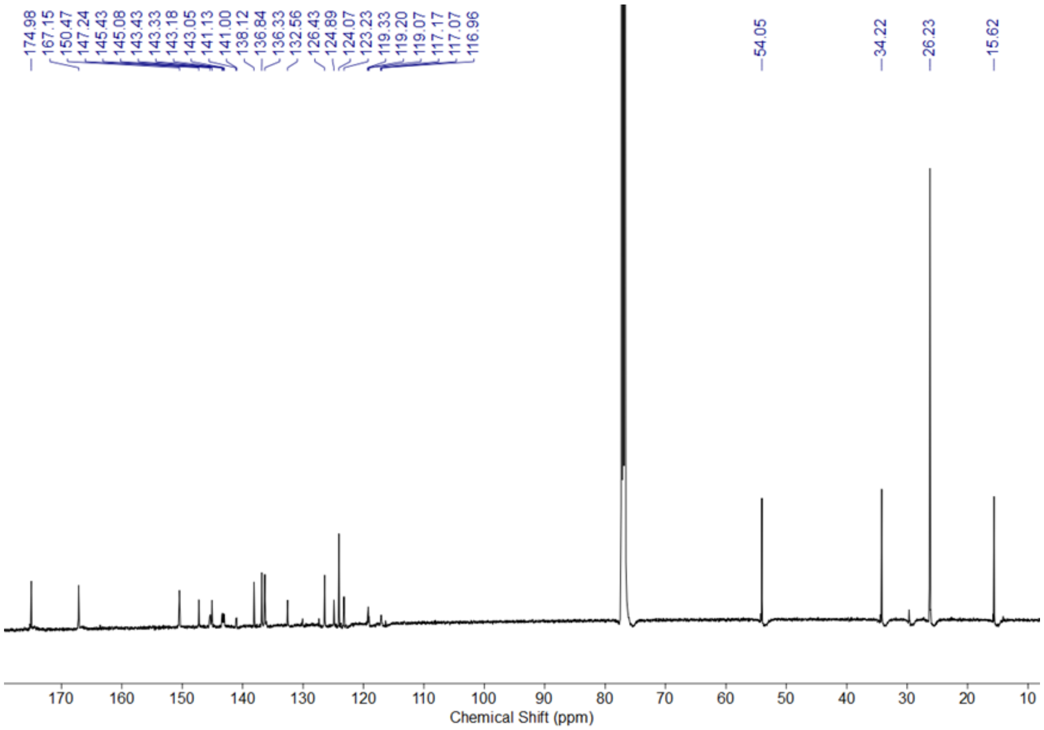

Figure S8. ^{13}C NMR spectrum of ligand **A** (126 MHz, $CDCl_3$, 25 °C).

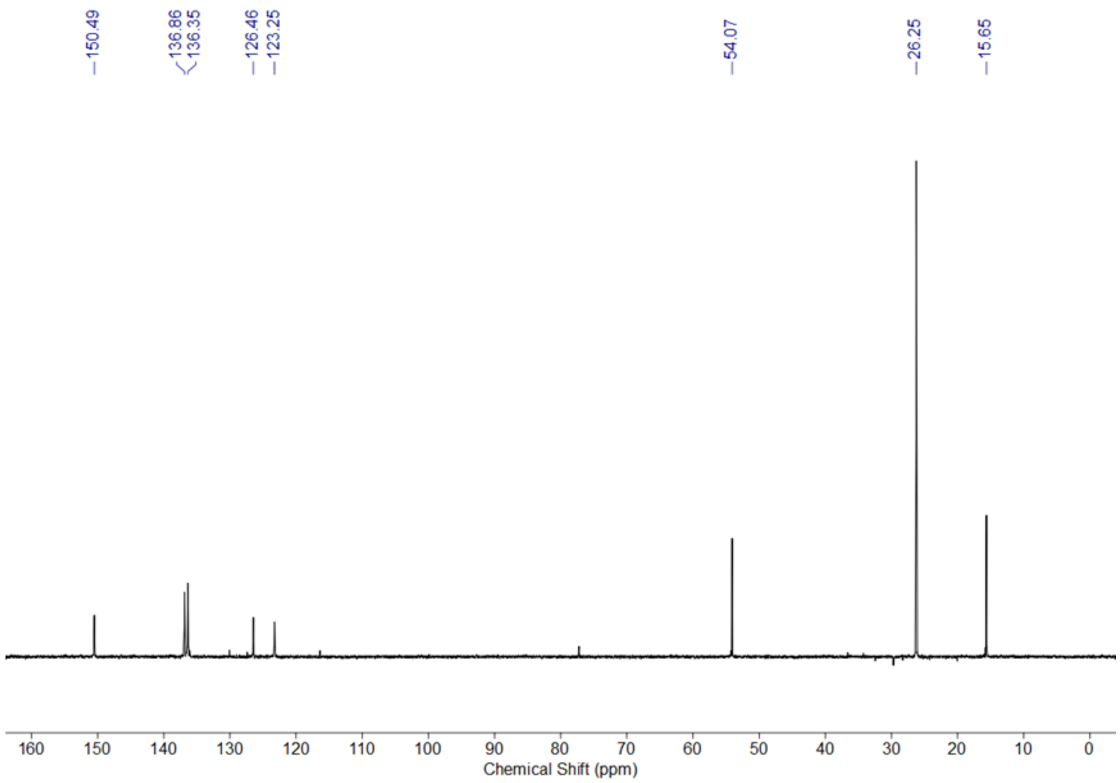

Figure S9. ^{13}C DEPT-135 NMR spectrum of ligand **A** (126 MHz, $CDCl_3$, 25 °C).

.

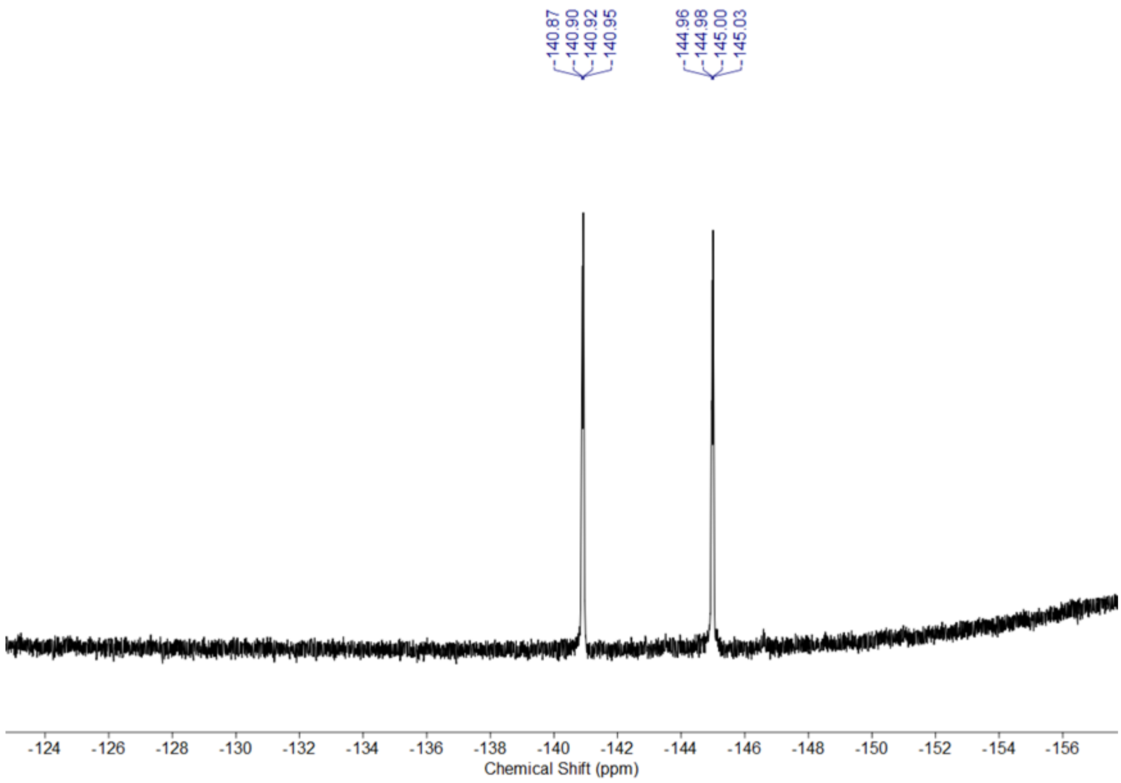

Figure S10. ^{19}F NMR spectrum of ligand **A** (470 MHz, $CDCl_3$, 25 °C).

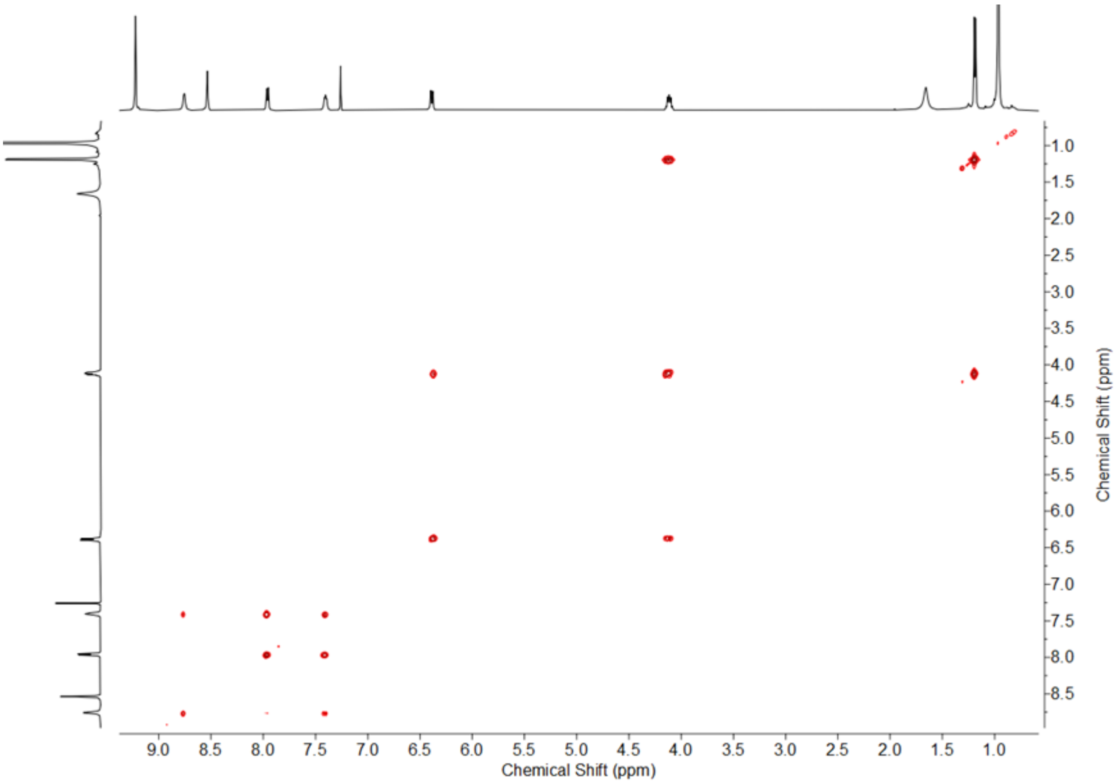

Figure S11. ^{1}H-^{1}H COSY NMR spectrum of ligand **A** (500 MHz, $CDCl_3$, 25 °C).

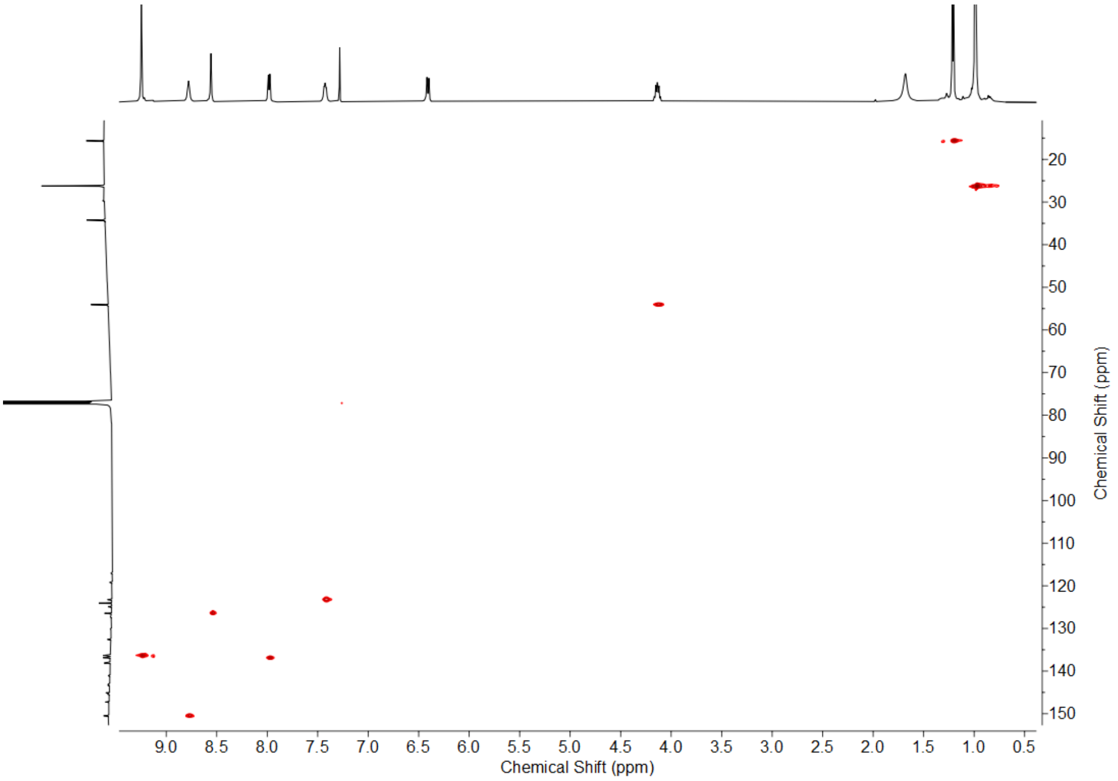

Figure S12. ^{1}H-^{13}C HSQC NMR spectrum of ligand **A** (500 MHz, $CDCl_3$, 25 °C).

3 Self-Assembly and Characterization of Cages 1 and 2

3.1 Self-Assembly of Cage 1 in MeCN

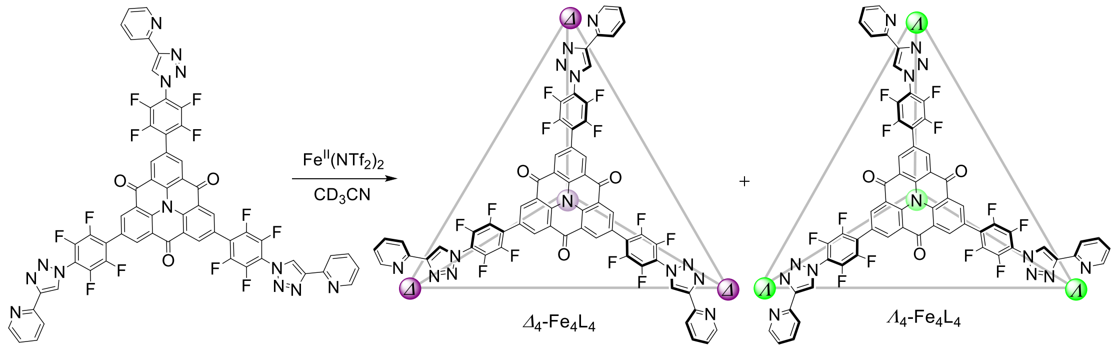

A

Cage **1**: Δ_4:Λ_4 = 1:1 (racemic)

Ligand **A** (24.0 mg, 20.0 μmol, 1.0 equiv) and $Fe(NTf_2)_2 \cdot 4.5H_2O$ (13.9 mg, 20.0 μmol, 1.0 equiv) were combined in MeCN (5 mL) in a 15 mL tube. The reaction mixture was stirred at 70 °C for 2 hours under nitrogen. The solvent was evaporated to around 2 mL, and Et_2O (30 mL) was then added. The precipitate was collected by centrifugation and washed with excess Et_2O, affording cage **1** as a brown solid (33.0 mg, 91%).

One set of signals was observed in the ^{1}H NMR spectrum of cage **1**. No Cotton effects were observed in the CD spectrum of **1** (Figure S31). These data indicate that cage **1** exists as a racemic mixture of Δ_4-**1** and Λ_4-**1**.

^{1}H NMR (500 MHz, CD_3CN): δ 7.64–7.70 (m, 12H), 8.14–8.31 (m, 24H), 8.39 (d, *J* = 8.0 Hz, 12H), 8.99 (s, 12H), 9.00 (s, 12H), 9.19 (s, 12H) ppm. **^{13}C NMR** (125 MHz, CD_3CN): δ 121.6–121.9 (m, C_6F_4), 122.0–122.2 (m, C_6F_4), 124.4, 124.9, 125.0, 128.0, 128.1, 136.2, 139.6, 140.8, 141.7–142.3 (m, C_6F_4), 143.8–144.2 (m, C_6F_4), 144.3–144.9 (m, C_6F_4), 146.2–146.9 (m, C_6F_4), 151.6, 152.8, 156.6, 176.4 ppm. **^{19}F NMR** (376 MHz, CD_3CN): δ -80.2 (s, NTf_2^-), -143.8– -144.2 (m, C_6F_4), -147.3– -147.9 (m, C_6F_4) ppm.

HR-ESI-MS: m/z = 627.8021 [**1**-8(NTf_2)]$^{8+}$, 757.4743 [**1**-7(NTf_2)]$^{7+}$, 930.5415 [**1**-6(NTf_2)]$^{6+}$, 1172.6337 [**1**-5(NTf_2)]$^{5+}$, 1535.7728 [**1**-4(NTf_2)]$^{4+}$.

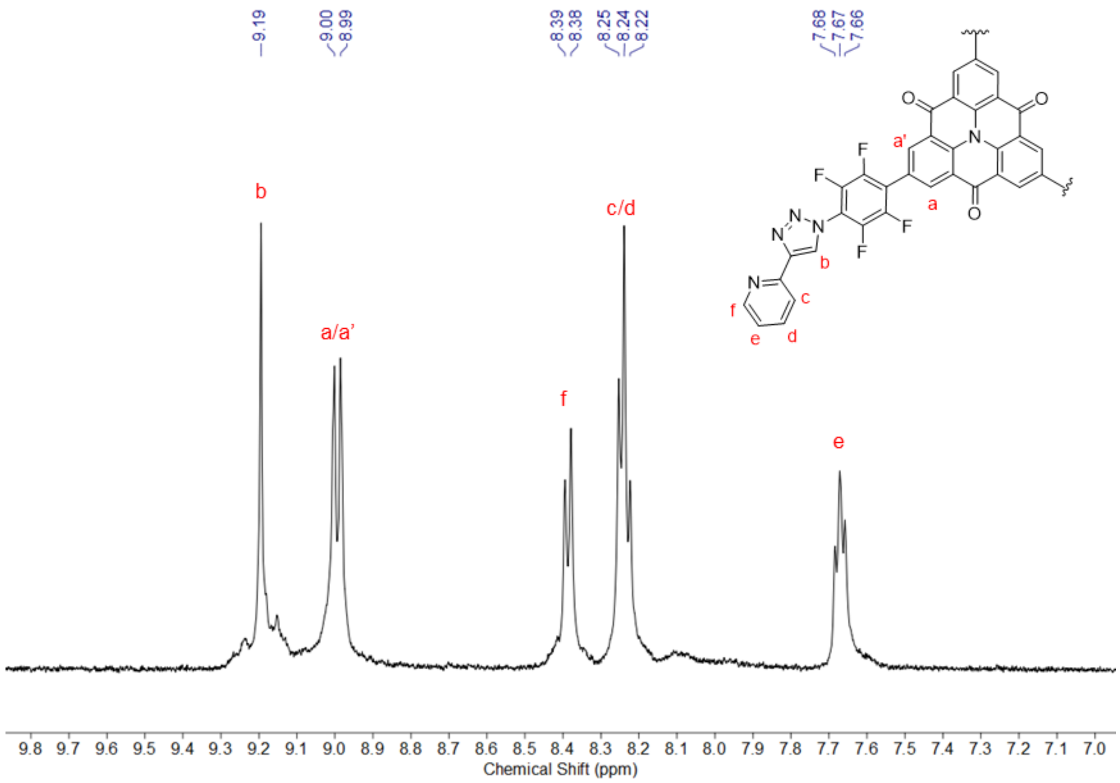

Figure S13. ^{1}H NMR spectrum of cage **1** (500 MHz, CD_3CN, 25 °C).

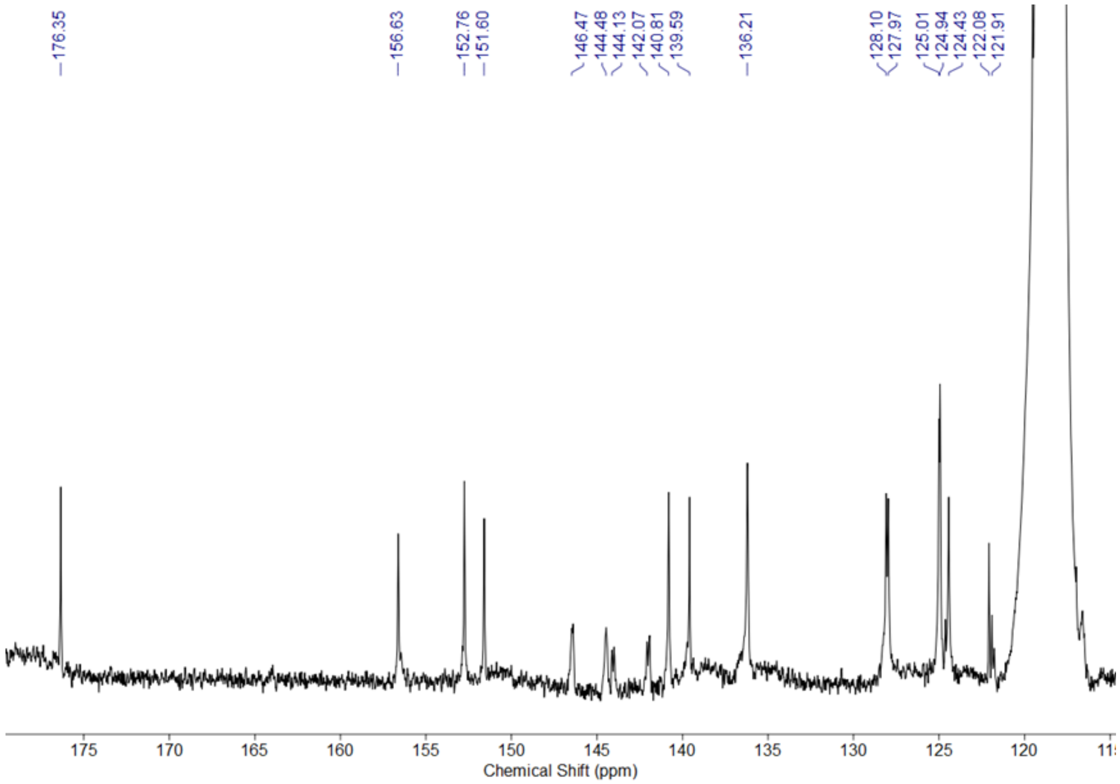

Figure S14. ^{13}CNMR spectrum of cage **1** (126 MHz, CD_3CN, 25 °C).

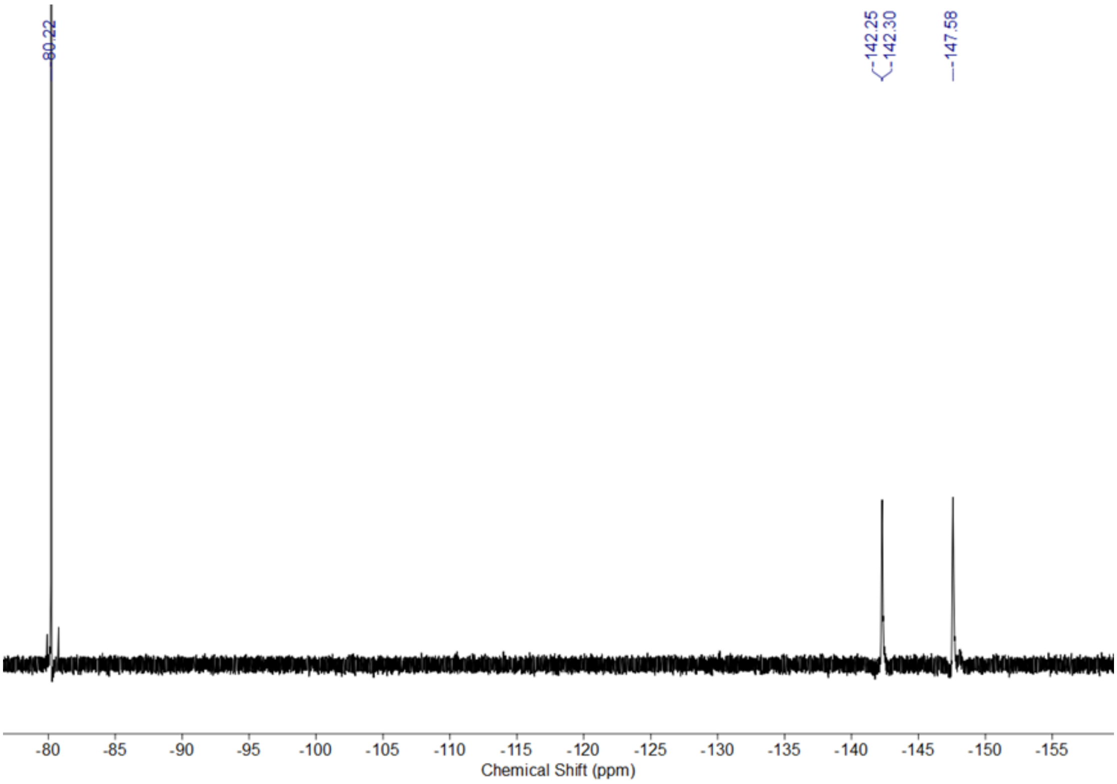

Figure S15. ^{19}F NMR spectrum of cage **1** (376 MHz, CD_3CN, 25 °C).

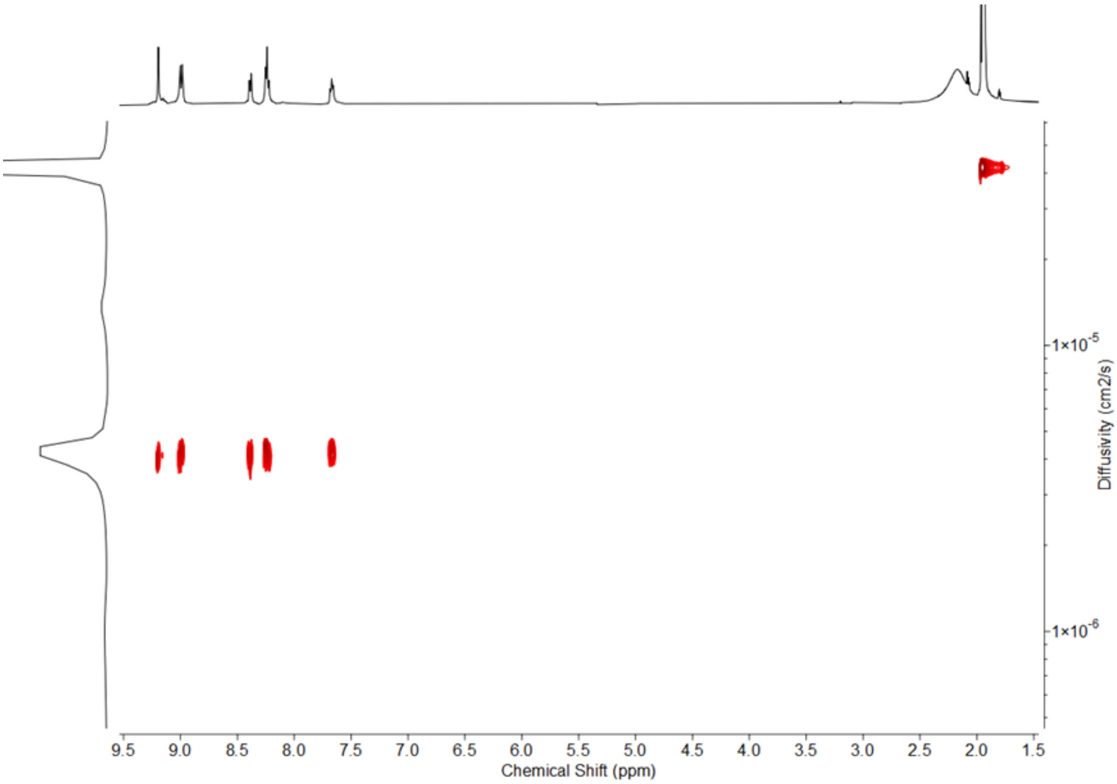

Figure S16. ^{1}H DOSY spectrum of cage **1** (400 MHz, CD_3CN, 25 °C). The diffusion coefficient was measured to be 4.21×10^{-6} cm^2/s.

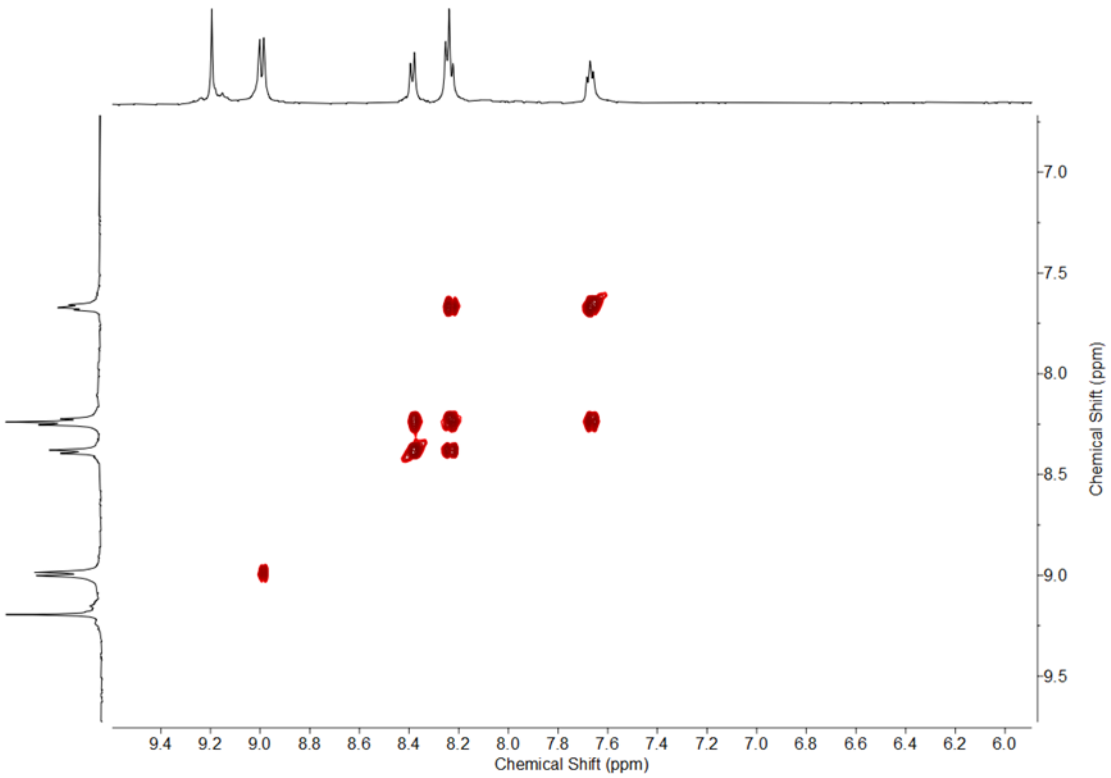

Figure S17. ^{1}H-^{1}H COSY NMR spectrum of cage **1** (500 MHz, CD_3CN, 25 °C).

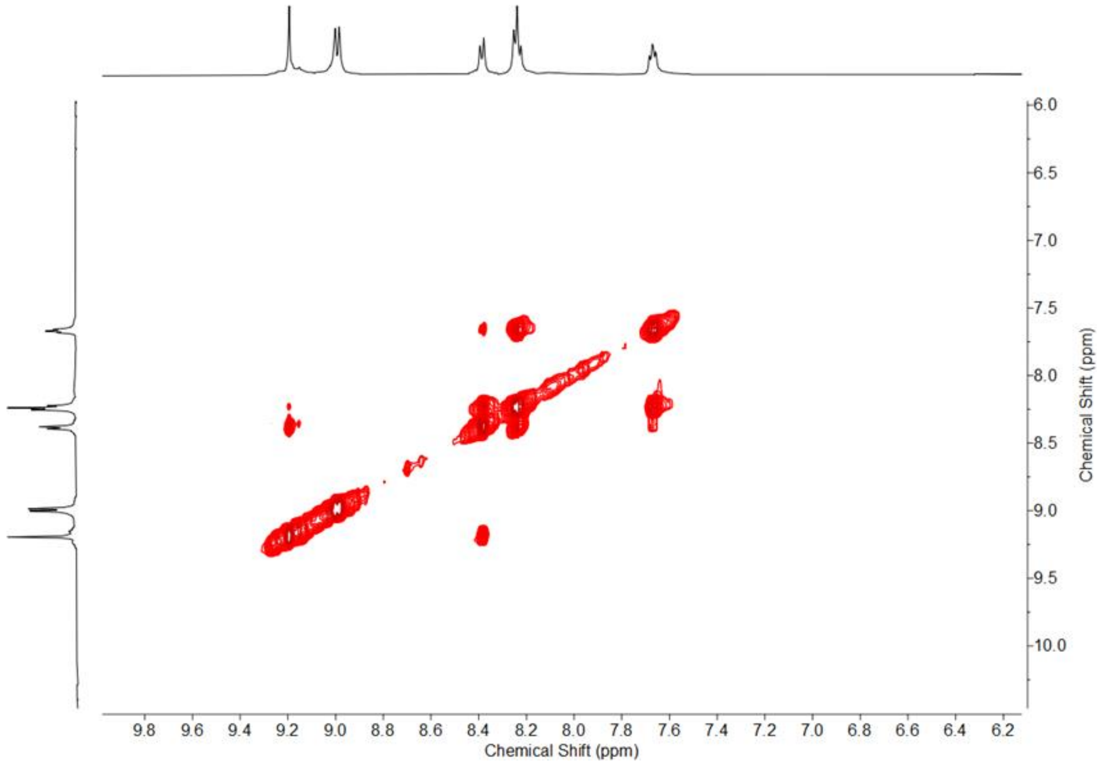

Figure S18. ^{1}H-^{1}H NOESY NMR spectrum of cage **1** (500 MHz, CD_3CN, 25 °C).

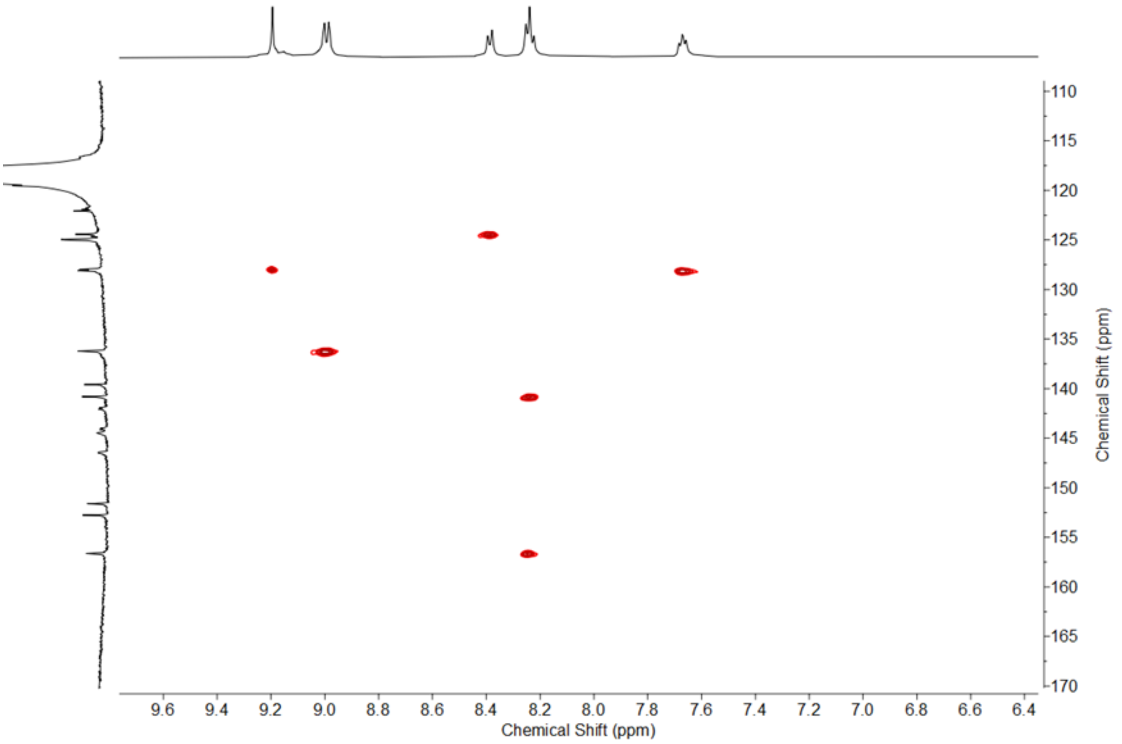

Figure S19. ^{1}H-^{13}C HSQC NMR spectrum of cage **1** (500 MHz, CD_3CN, 25 °C).

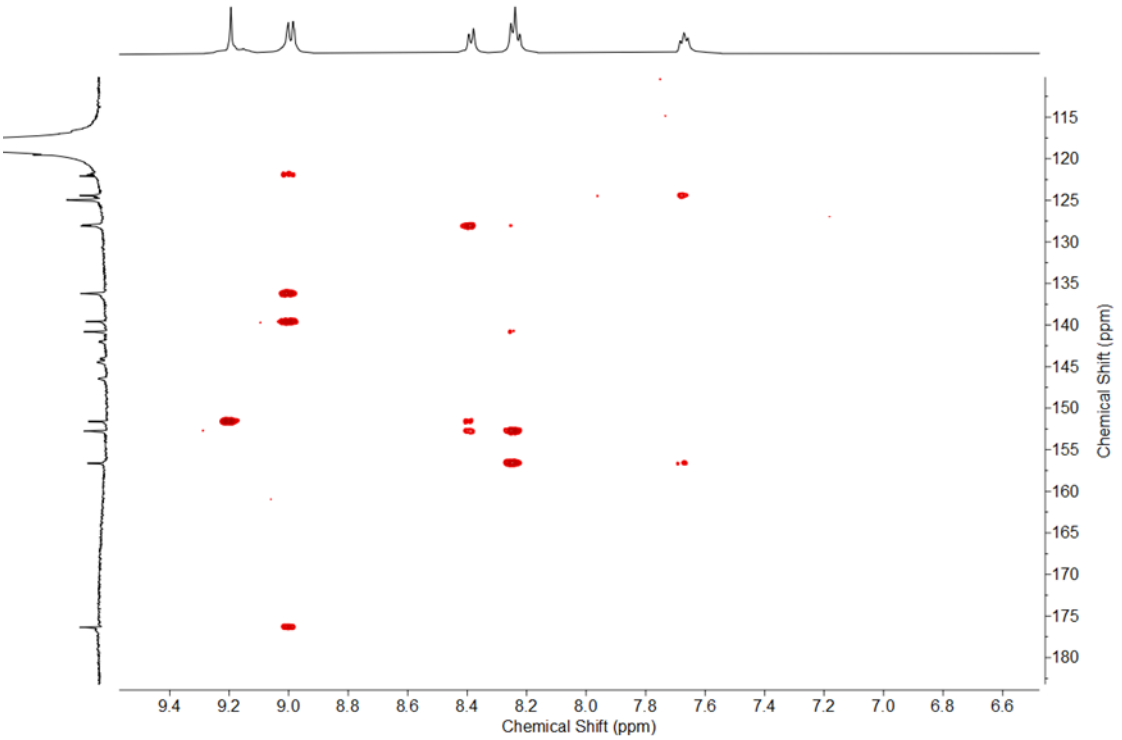

Figure S20. ^{1}H-^{13}C HMBC NMR spectrum of cage **1** (500 MHz, CD_3CN, 25 °C).

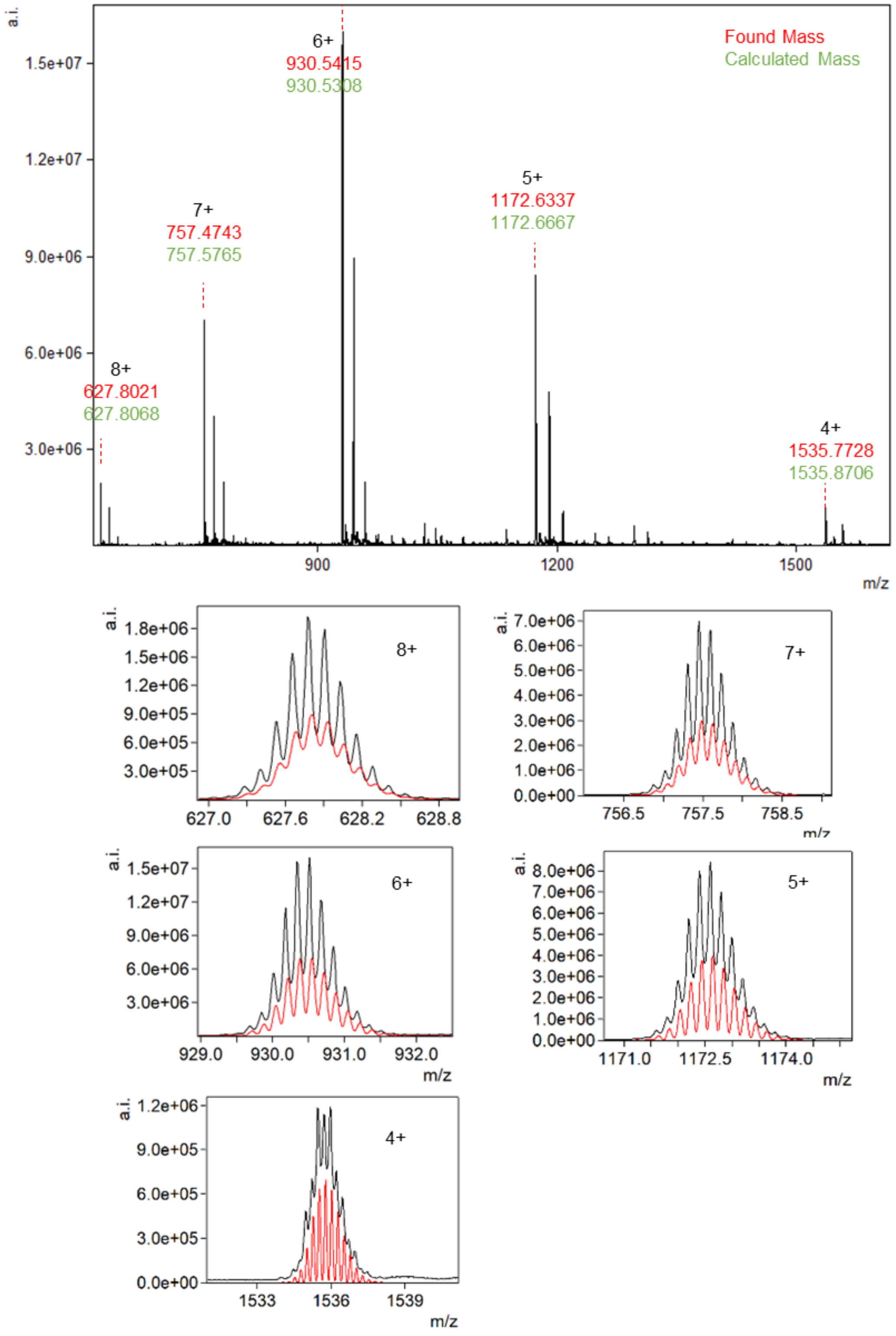

Figure S21. High-resolution ESI-MS spectrum of cage **1** in MeCN. Three sets of peaks were observed in the spectrum; the other two sets correspond to **1**·2MeCN and **1**·4MeCN, respectively.

3.2 Self-Assembly of Cage 2 in MeCN

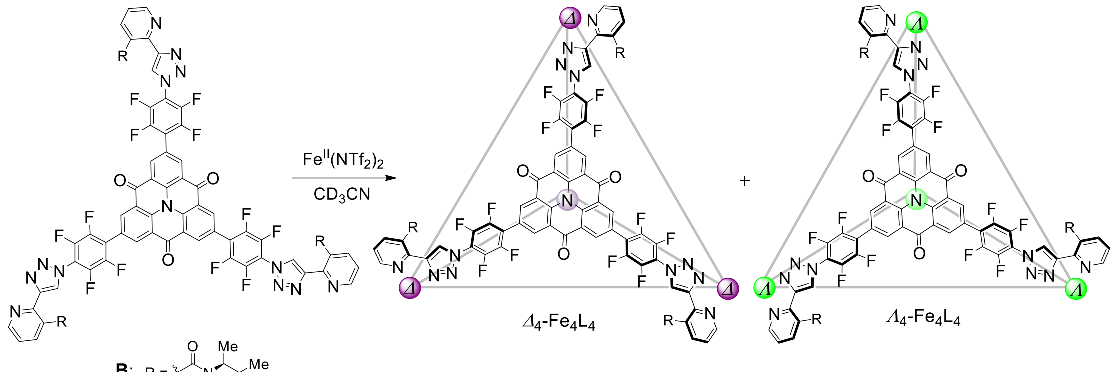

Cage **2**: Δ_4:Λ_4 = 2.4:1 (d.r.)

Ligand **B** (31.6 mg, 20.0 μmol, 1.0 equiv) and $Fe(NTf_2)_2 \cdot 4.5H_2O$ (13.9 mg, 20.0 μmol, 1.0 equiv) were combined in MeCN (5 mL) in a 15 mL tube. The reaction mixture was stirred at 70 °C for 2 hours under nitrogen. The solvent was evaporated to around 2 mL, and Et_2O (30 mL) was then added. The precipitate was collected by centrifugation and washed with excess Et_2O, affording cage **2** as a brown solid (39.5 mg, 90%).

Cage **2** consists of a pair of diastereomers, with each having either four *Δ* or four *Λ* zinc vertices. According to the ^1H NMR spectrum, the diastereomeric ratio (d.r.) was determined to be 2.4:1. Comparison with the CD spectra of structurally similar *Δ*- and *Λ*-$[Fe(bpy)_3]^{2+}$ complexes allowed us to infer there is an excess of the *Δ* configuration of iron centers within cage **2**.[4,5] The major diastereomer of **2** is thus determined to be Δ_4-**2**, whereas the minor diastereomer is Λ_4-**2**.

^{1}H NMR (500 MHz, CD_3CN): δ 1.00 (s, 76.2H, Δ_4-**2**), 1.03 (s, 31.8H, Λ_4-**2**), 1.20 (d, *J* = 6.5 Hz, 10.6H, Λ_4-**2**), 1.26 (d, *J* = 6.5 Hz, 25.4H, Δ_4-**2**), 4.08–4.21 (m, 12H), 7.13 (d, *J* = 9.6 Hz, 12H), 7.62–7.72 (m, 12H), 8.19–8.36 (m, 24H), 8.87–9.00 (m, 24H), 9.04 (s, 3.5H, Λ_4-**2**), 9.06 (s, 8.5H, Δ_4-**2**).

HR-ESI-MS: m/z = 818.5426 [**2**-8(NTf$_2$)]$^{8+}$, 975.4662 [**2**-7(NTf$_2$)]$^{7+}$, 1184.8581 [**2**-6(NTf$_2$)]$^{6+}$, 1477.8074 [**2**-5(NTf$_2$)]$^{5+}$, 1917.2447 [**2**-4(NTf$_2$)]$^{4+}$.

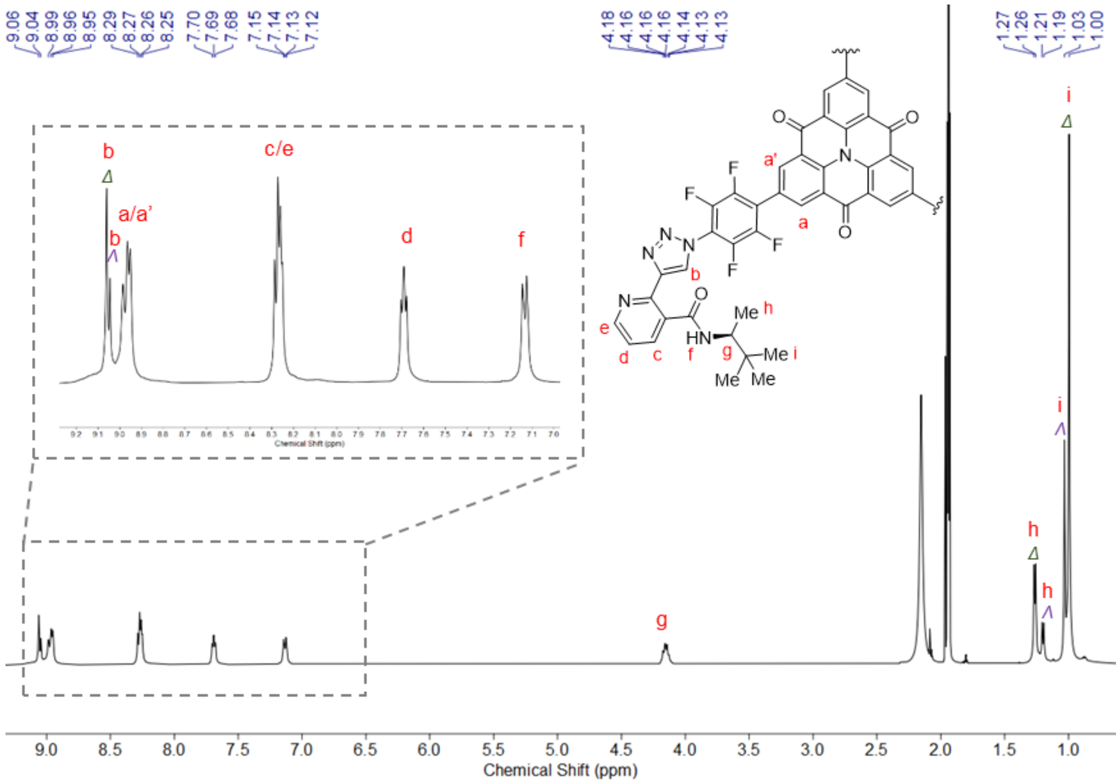

Figure S22. ^{1}H NMR spectrum of cage **2** (500 MHz, CD_3CN, 25 °C).

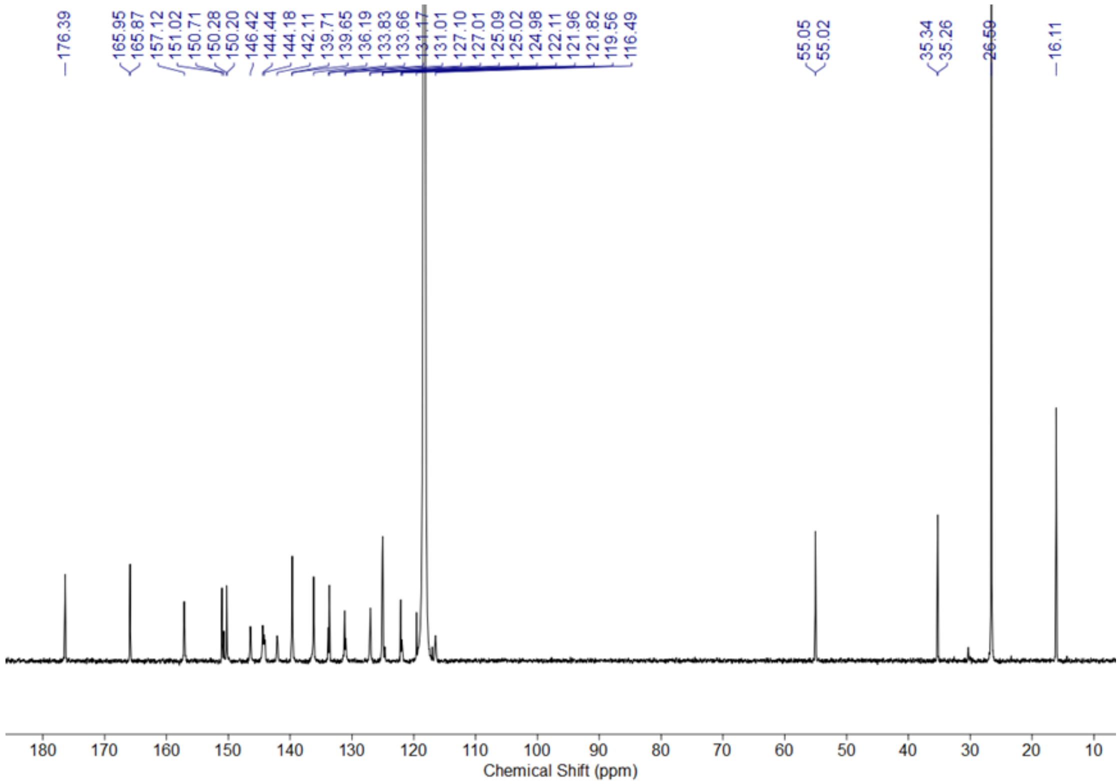

Figure S23. ^{13}C NMR spectrum of cage **2** (126 MHz, CD_3CN, 25 °C).

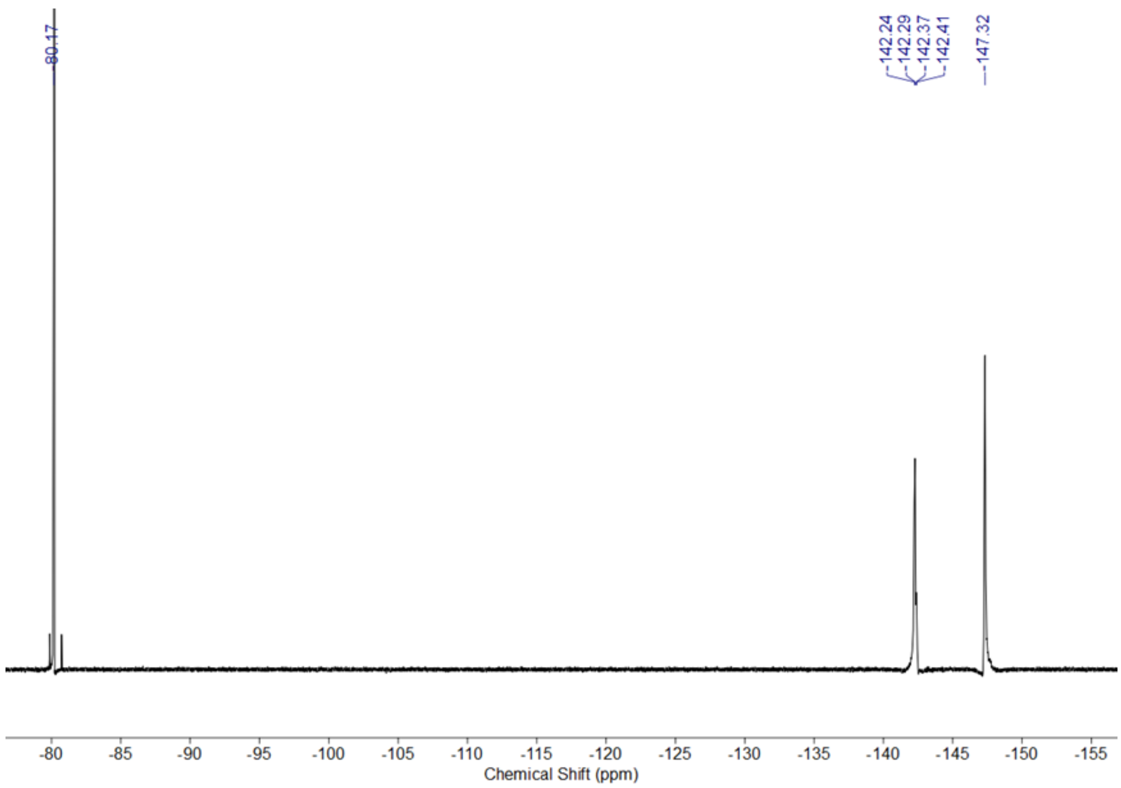

Figure S24. ^{19}F NMR spectrum of cage **2** (376 MHz, CD_3CN, 25 °C).

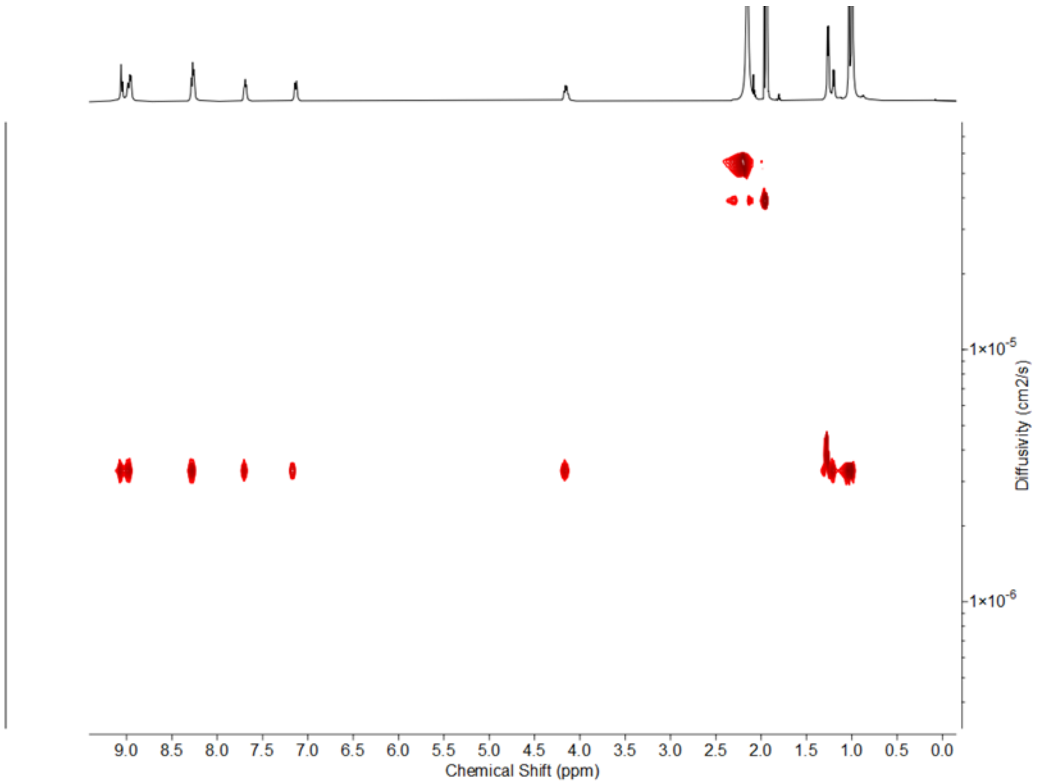

Figure S25. ^{1}H DOSY spectrum of cage **2** (400 MHz, CD_3CN, 25 °C). The diffusion coefficient was measured to be 3.35×10^{-6} cm^2/s.

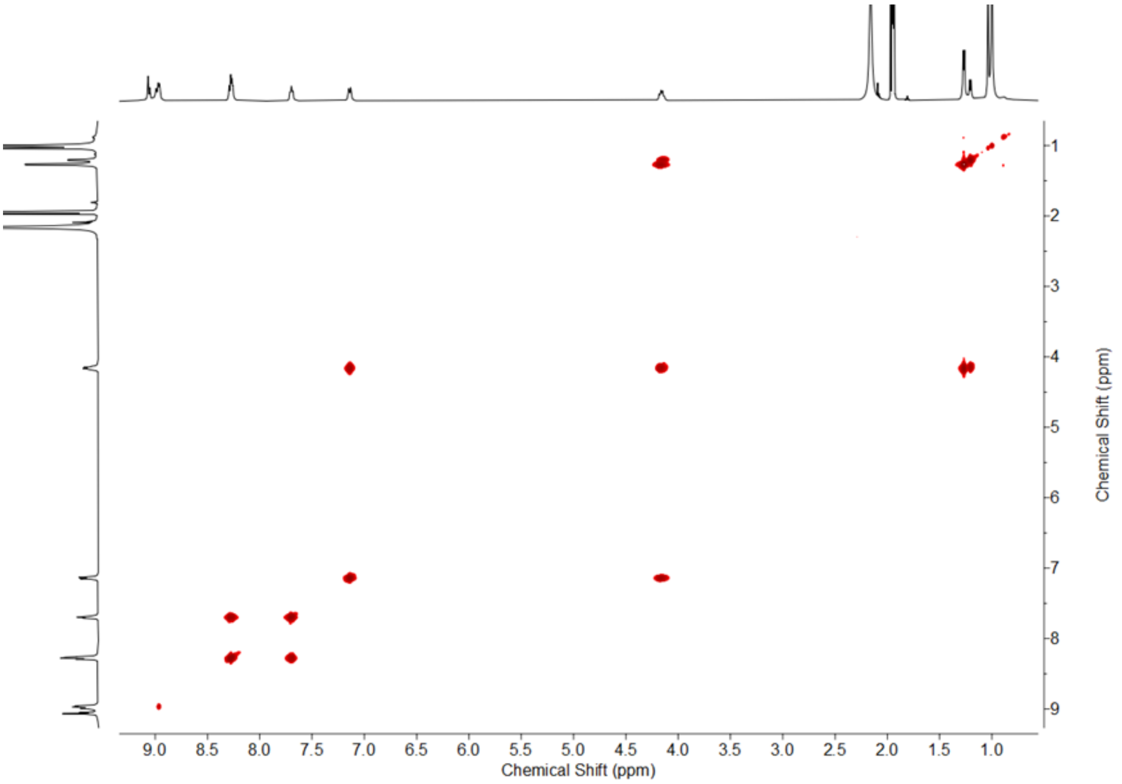

Figure S26. ^{1}H-^{1}H COSY NMR spectrum of cage **2** (500 MHz, CD_3CN, 25 °C).

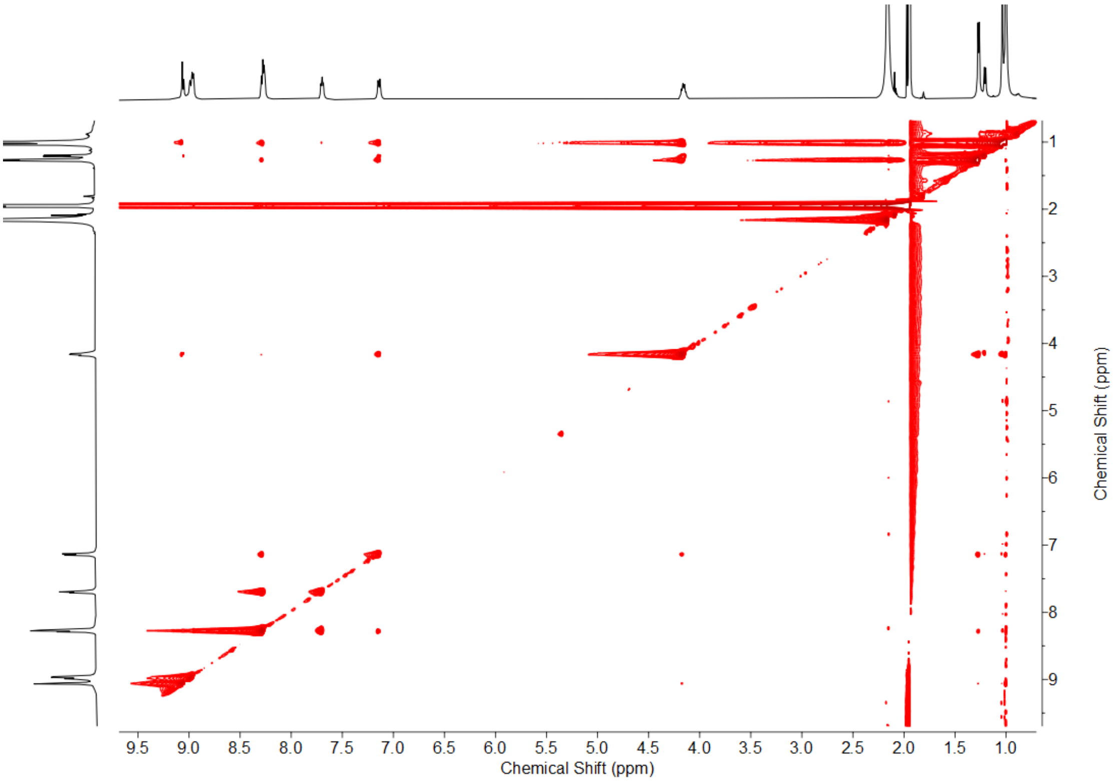

Figure S27. ^{1}H-^{1}H NOESY NMR spectrum of cage **2** (500 MHz, CD_3CN, 25 °C).

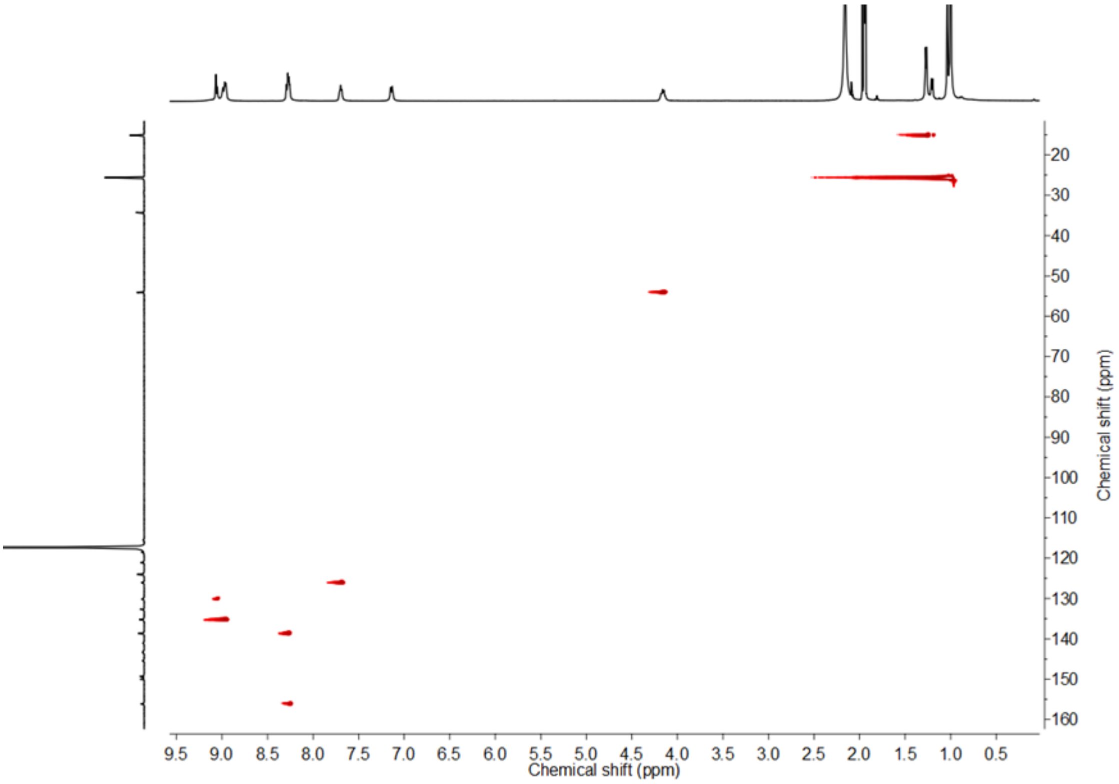

Figure S28. ^{1}H-^{13}C HSQC NMR spectrum of cage **2** (500 MHz, CD_3CN, 25 °C).

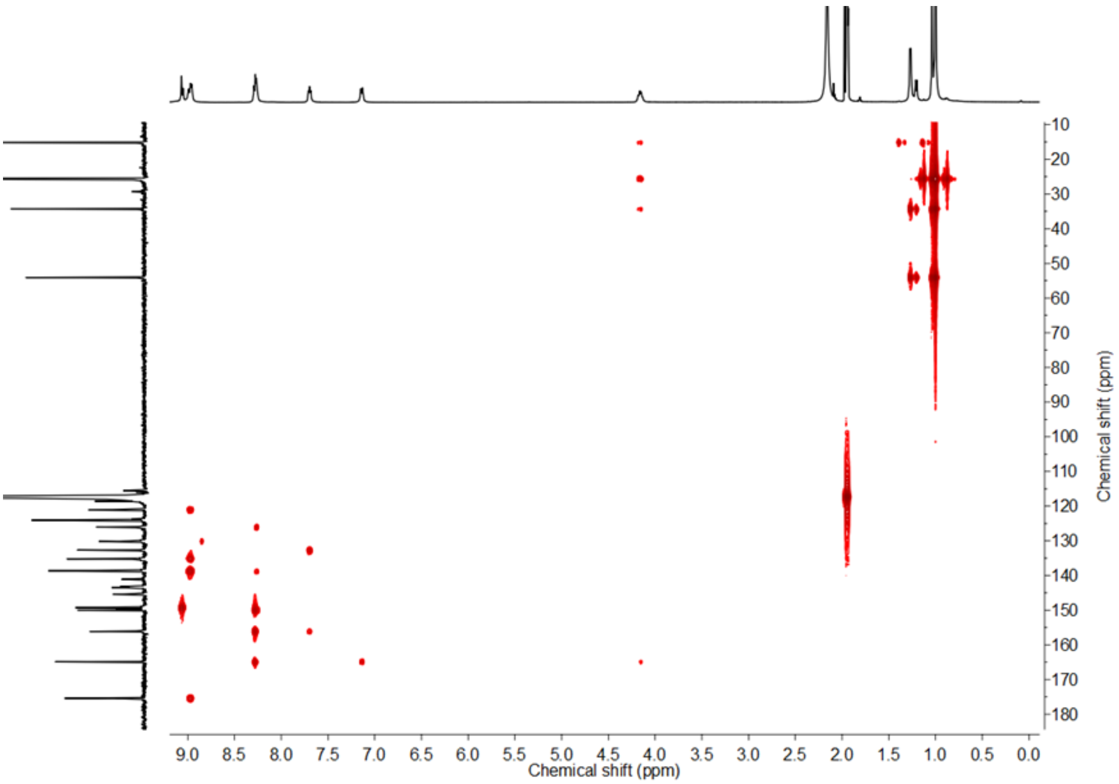

Figure S29. ^{1}H-^{13}C HMBC NMR spectrum of cage **2** (500 MHz, CD_3CN, 25 °C).

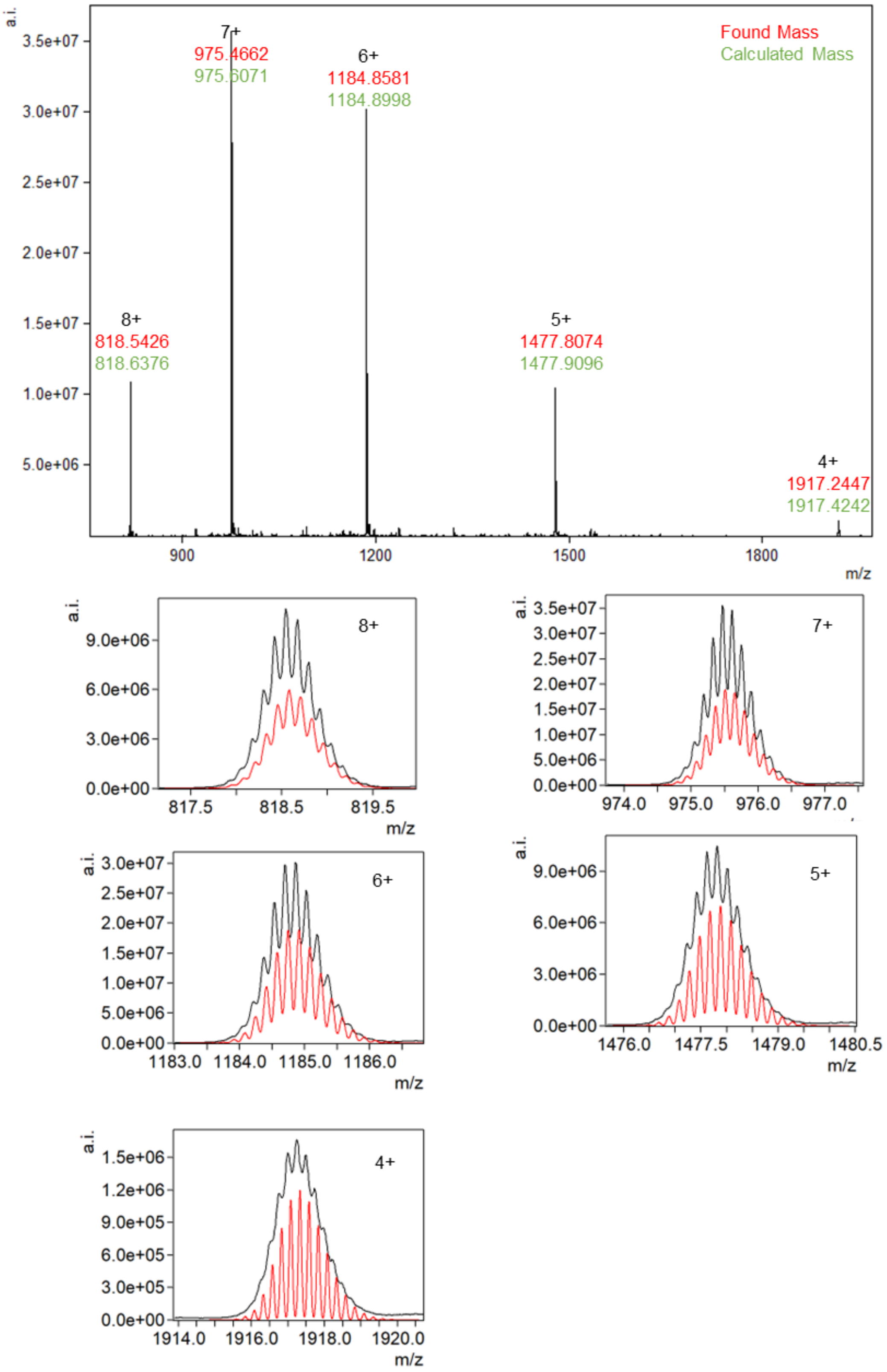

Figure S30. High-resolution ESI-MS spectrum of cage **2** in MeCN.

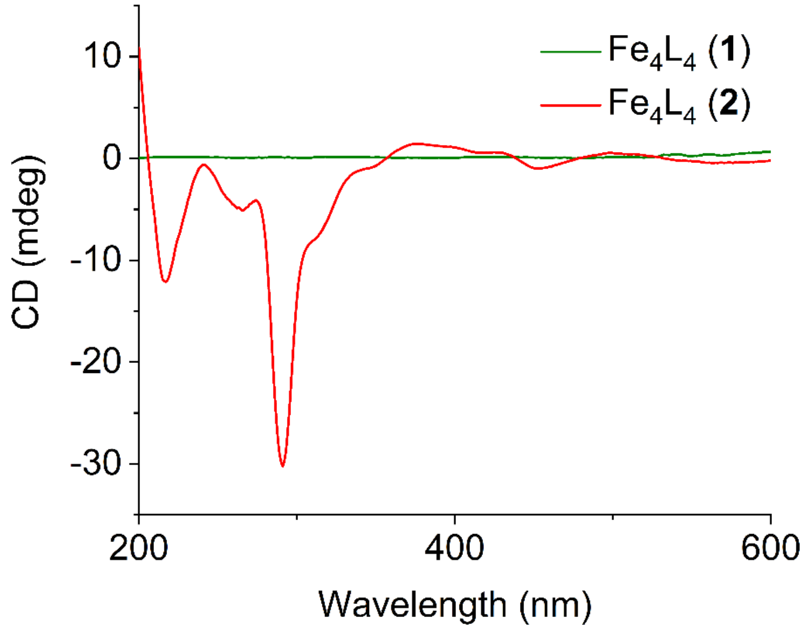

Figure S31. CD spectra of cages **1** and **2** in MeCN.

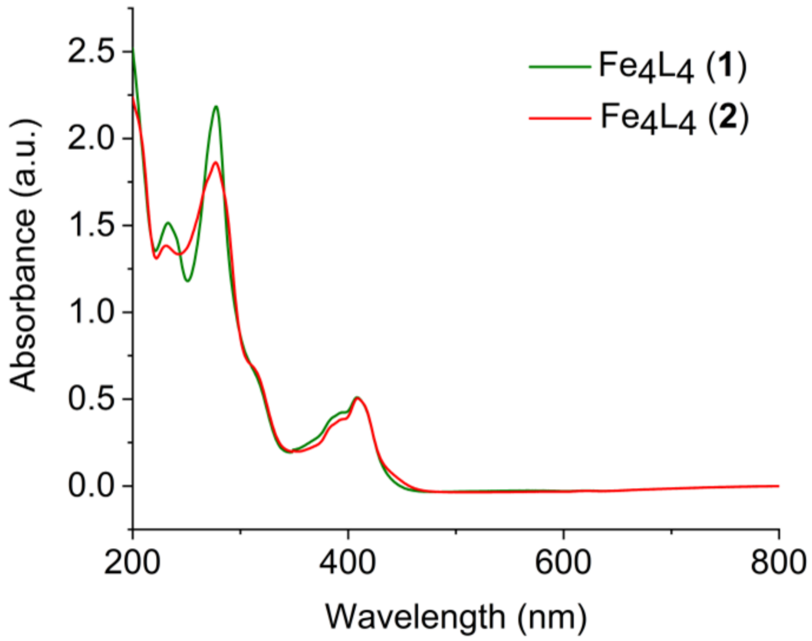

Figure S32. UV-vis spectra of cages **1** and **2** in MeCN.

4 Investigation of Temperatures and Chiral Side Chains

4.1 Investigation of Temperatures

The self-assembly of cage **2** was also performed at different temperatures. After cooling down to room temperature, the ^{1}H NMR spectra were recorded. We observed that elevating reaction temperatures did not result in changes in diastereometric ratio.

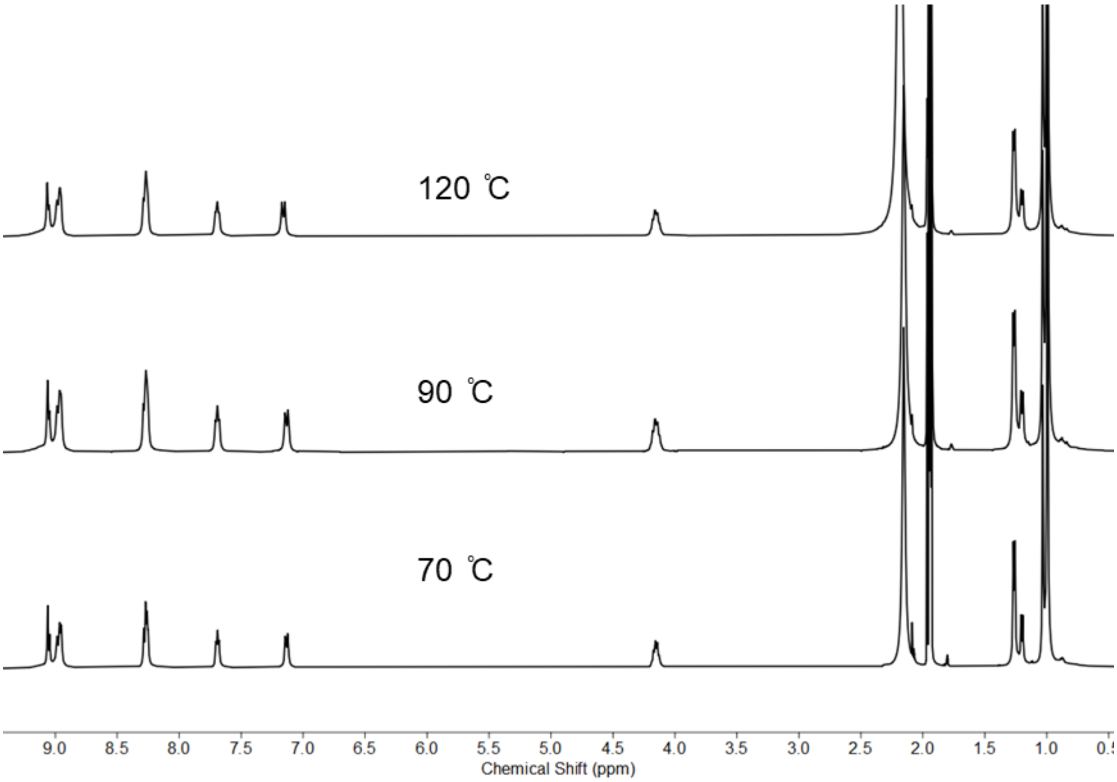

Figure S33. Comparison of ^{1}H NMR spectra of **2** assembled at different temperatures (400 and 500 MHz, CD_3CN, 25 °C).

4.2 Investigation of Chiral Side Chains

We also tried to use different *N*-heterotriangulene based chiral ligands bearing modified chiral directing groups; however, lower diastereomeric ratios were observed.

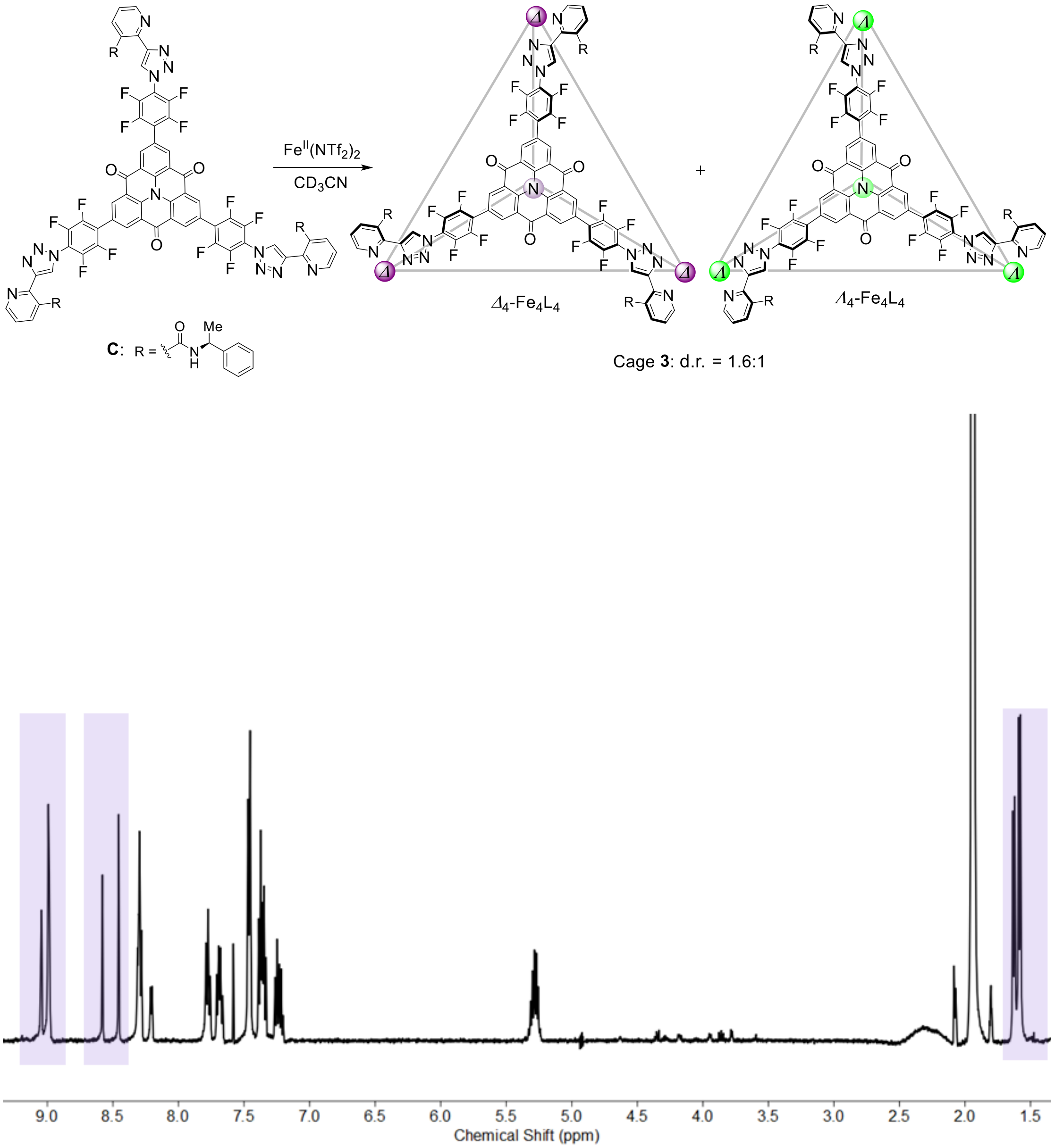

Figure S34. Self-assembly of cage **3** and ^{1}H NMR spectrum of **3** (500 MHz, CD_3CN, 25 °C).

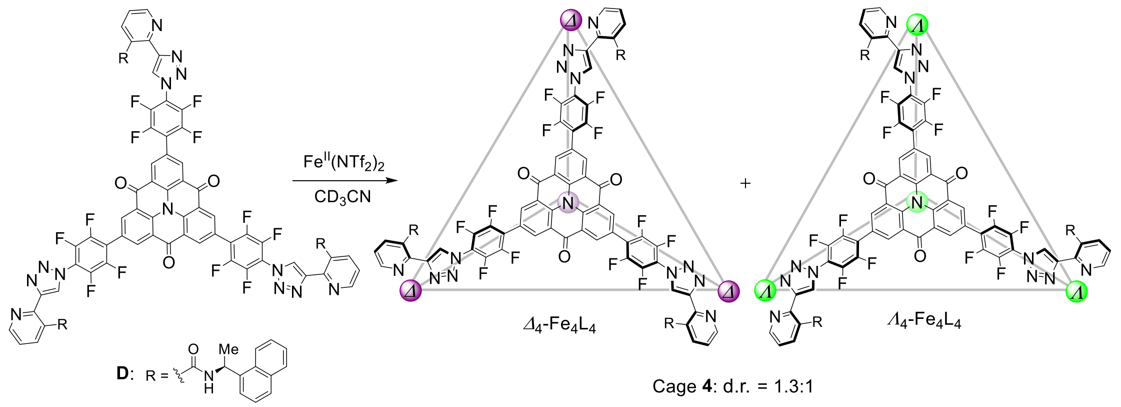

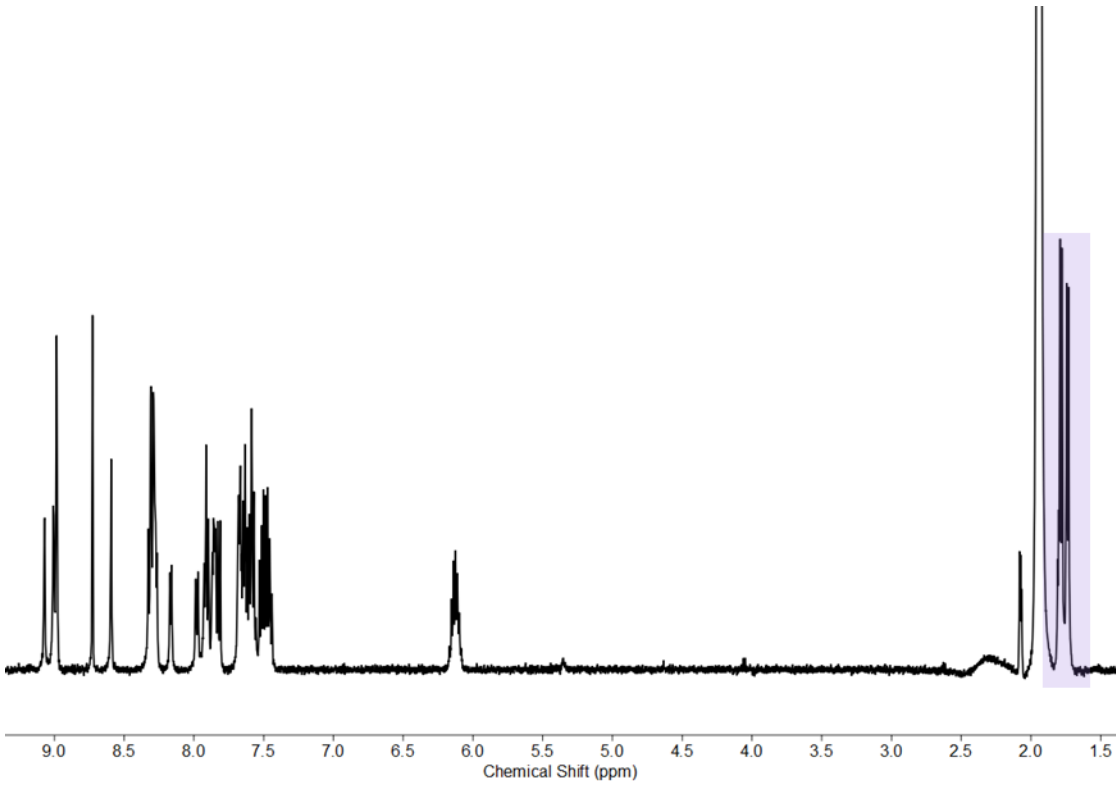

Figure S35. Self-assembly of cage **4** and ^{1}H NMR spectrum of **4** (500 MHz, CD_3CN, 25 °C).

5 Investigation of the Monomeric $Fe^{II}L_3$ Complex

A monomeric pyridyl-triazole ligand (**S5**) bearing the same chiral directing group was synthesized according to a reported procedure.[1] The self-assembly **S5** (2.10 mg, 6.0 µmol, 3.0 equiv) and $Fe(NTf_2)_2·4.5H_2O$ (1.4 mg, 2.0 µmol, 1.0 equiv) in CD_3CN at 70 °C for 30 mins gave rise to an $Fe^{II}L_3$ complex. Signals in the ^1H NMR spectrum of the FeL_3 complex are broad, likely due to the overlap of peaks corresponding to up to four different diastereomers, *fac-Δ*, *fac-Λ*, *mer-Δ* and *mer-Λ*. Weaker Cotton effects, corresponding to π-π* transitions (240–340 nm) and metal-to-ligand charge-transfer (MLCT) and d-d transitions (360–540 nm), are observed for the $Fe^{II}L_3$ complex compared to those of cage **2**. These results reflect that the diastereoselectivity of **2** emerges as a result of higher-order assembly. During the self-assembly process, both the stereochemical information transfer from ligand to metal vertex and stereochemical communication between metal centers may cooperatively play a pivotal role in amplifying the energy differences between the $Δ_4$ and $Λ_4$ configurations.

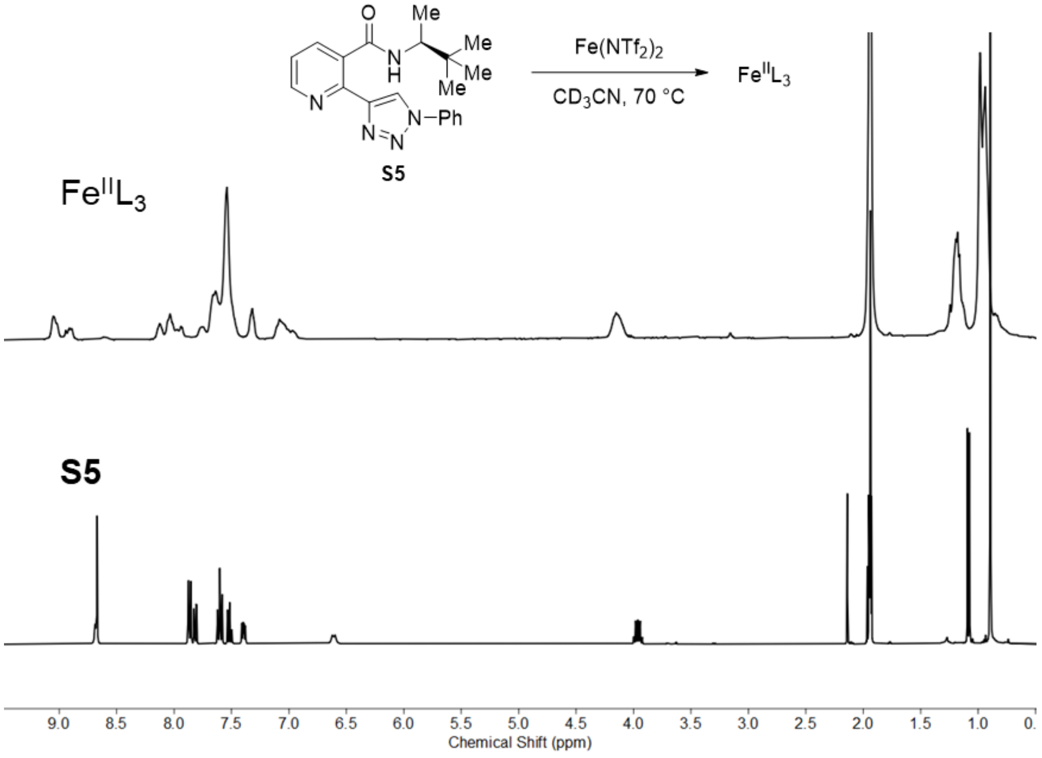

Figure S36. Comparison of the ^1H NMR spectra of **S5** and its corresponding $Fe^{II}L_3$ complex (400 MHz, CD_3CN, 25 °C).

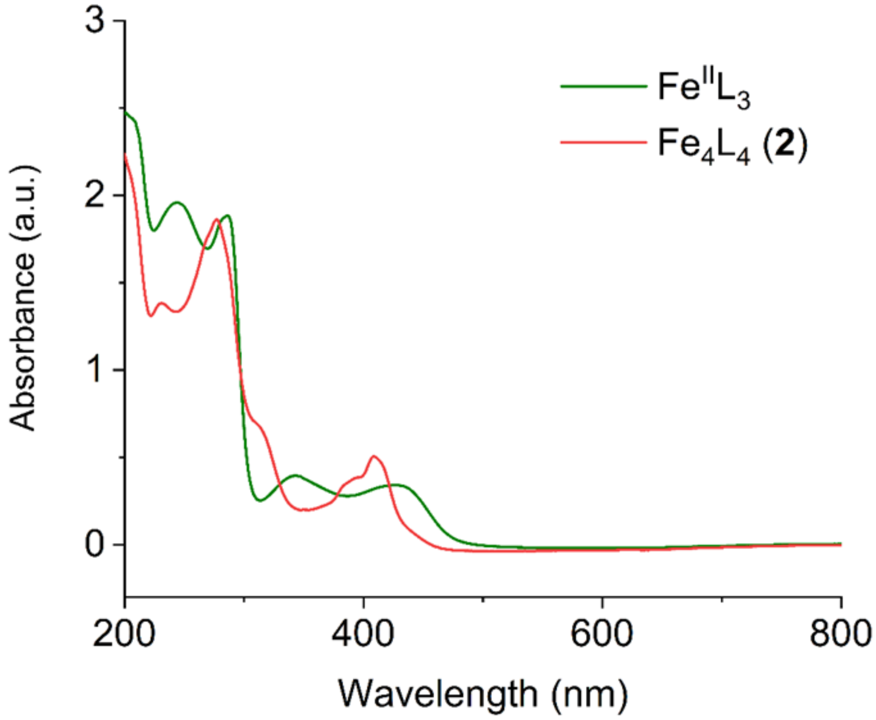

Figure S37. CD spectra of **2** and Fe^{II}L$_3$ complex in MeCN.

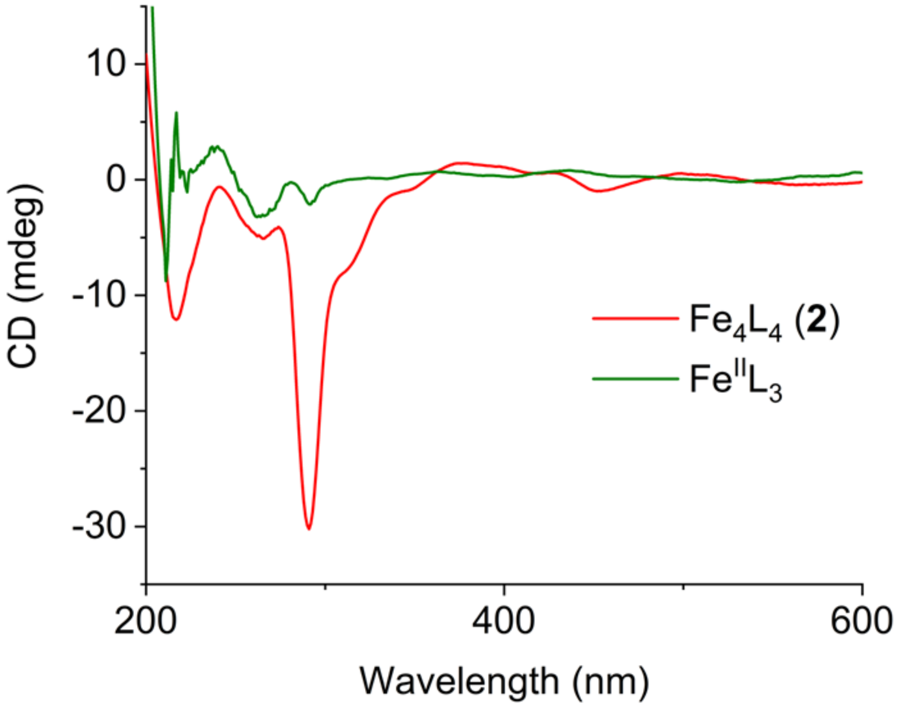

Figure S38. UV-vis spectra of **2** and Fe^{II}L$_3$ complex in MeCN.

6 Host-Guest Studies Using Cage 2

6.1 Encapsulation of Fullerenes and Fullerene Adducts

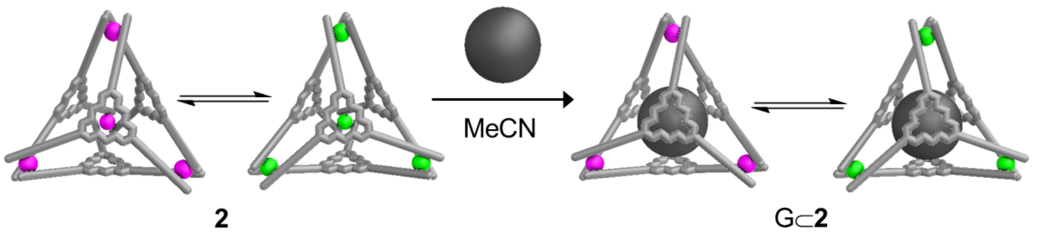

Complex	C_{60}⊂**2**	IC_{60}MA⊂**2**	C_{60}PCBM⊂**2**	Bis-C_{60}PCBM⊂**2**	C_{70}⊂**2**	C_{70}PCBM⊂**2**
d.r. (Δ_4:Λ_4)	3.3:1	2.8:1	2.4:1	1.7:1	4.1:1	3.6:1

General procedure for the encapsulation of fullerenes and fullerene adducts (GP1): Cage **2** (8.8 mg, 1.0 μmol, 1.0 equiv) and the guest molecule (**G**, 1.0 μmol, 1.0 equiv) were combined in MeCN (2 mL) in a 15 mL tube. The reaction mixture was stirred at 70 °C for 30 mins. After cooling down to room temperature, the reaction mixture was filtered through a short pipette filled with glass microfibre filter. The filtrate was evaporated to around 0.5 mL, and Et_2O (10 mL) was then added. The precipitate was collected by centrifugation and washed with excess Et_2O, affording the host-guest complex **G**⊂**2**.

The diastereomeric ratio of each host-guest complex was determined by ^{1}H NMR. In all cases, shifts in the signals in the ^{1}H NMR and ^{19}F NMR spectra were observed compared to the signals of empty cage **2**. In each case, the DOSY spectrum confirmed that all proton signals corresponding to the host-guest complex have the same diffusion coefficient. Either low-resolution ESI-MS or high-resolution ESI-MS spectra confirmed that a 1:1 host-guest complex was formed in all cases.

Characterization data for **G**⊂**2** are listed below:

$C_{60}\subset$**2** (8.5 mg, 89%) was prepared according to method **GP1**. The d.r. was determined to be 3.3:1 (Δ_4:Λ_4) by ^{1}H NMR.

^{1}H NMR (400 MHz, CD_3CN): δ 0.98 (s, 83H, $C_{60}\subset\Delta_4$-**2**), 1.03 (s, 25H, $C_{60}\subset\Lambda_4$-**2**), 1.18 (d, J = 6.8 Hz, 8.3H, $C_{60}\subset\Lambda_4$-**2**), 1.26 (d, J = 6.8 Hz, 27.7H, $C_{60}\subset\Delta_4$-**2**), 4.09–4.21 (m, 12H), 7.15 (d, J = 9.6 Hz, 12H), 7.65–7.77 (m, 12H), 8.22–8.32 (m, 12H), 8.39–8.49 (m, 12H), 8.96–9.02 (m, 24H), 9.03–9.08 (m, 12H).

ESI-MS: m/z = 909.08 $[C_{60}\subset$**2**$\text{-8(NTf}_2)]^{8+}$, 1079.02 $[C_{60}\subset$**2**$\text{-7(NTf}_2)]^{7+}$, 1305.32 $[C_{60}\subset$**2**$\text{-6(NTf}_2)]^{6+}$, 1622.46 $[C_{60}\subset$**2**$\text{-5(NTf}_2)]^{5+}$.

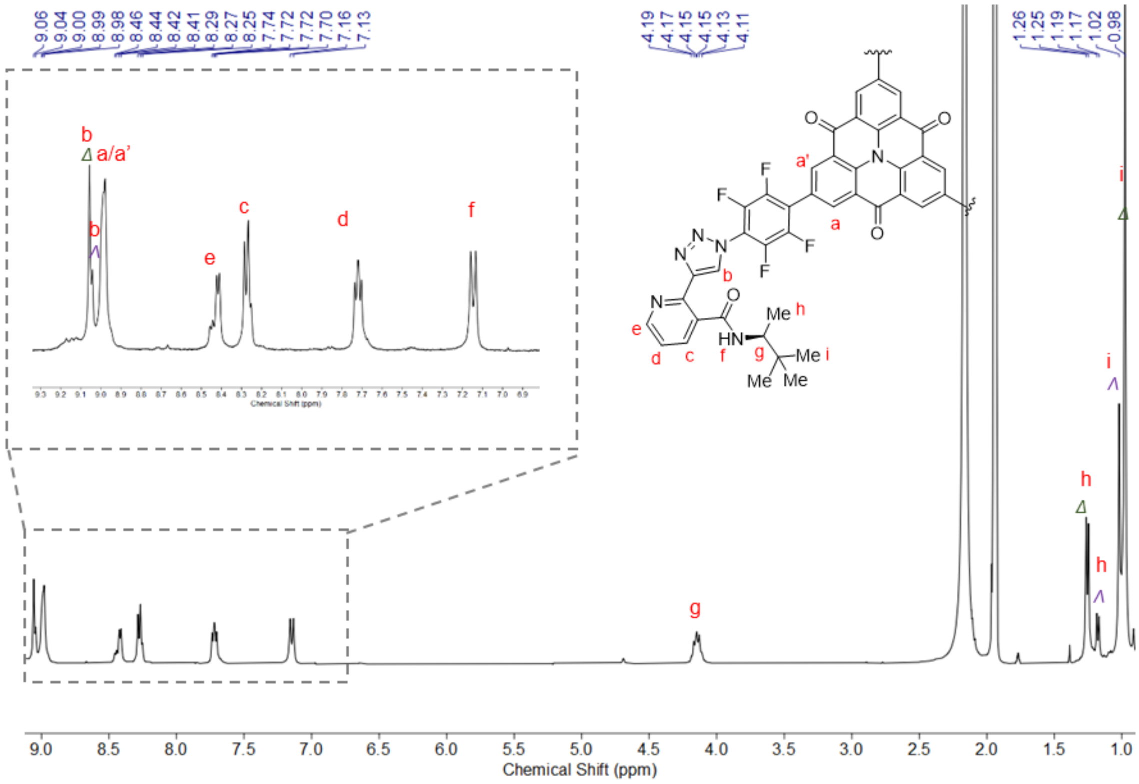

Figure S39. ^{1}H NMR spectrum of $C_{60}\subset$**2** (400 MHz, CD_3CN, 25 °C).

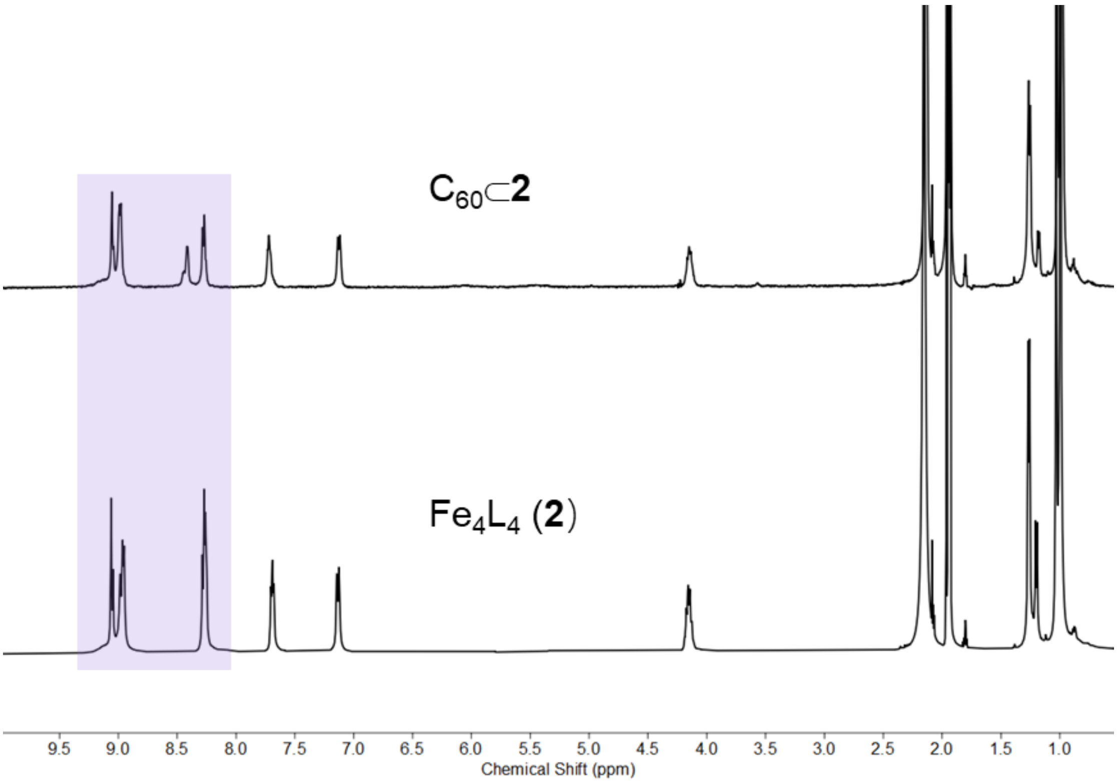

Figure S40. Comparison of the ^{1}H NMR spectra of **2** and C_{60}⊂**2**.

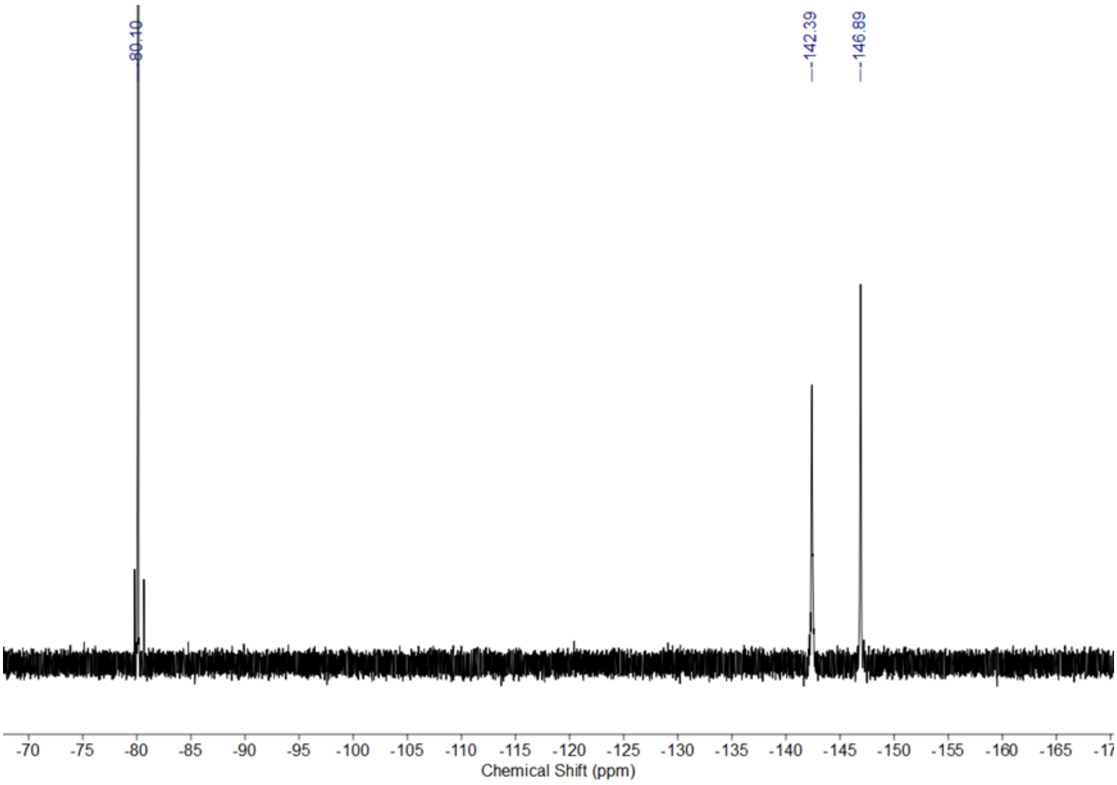

Figure S41. ^{19}F NMR spectrum of C_{60}⊂**2** (376 MHz, CD_3CN, 25 °C).

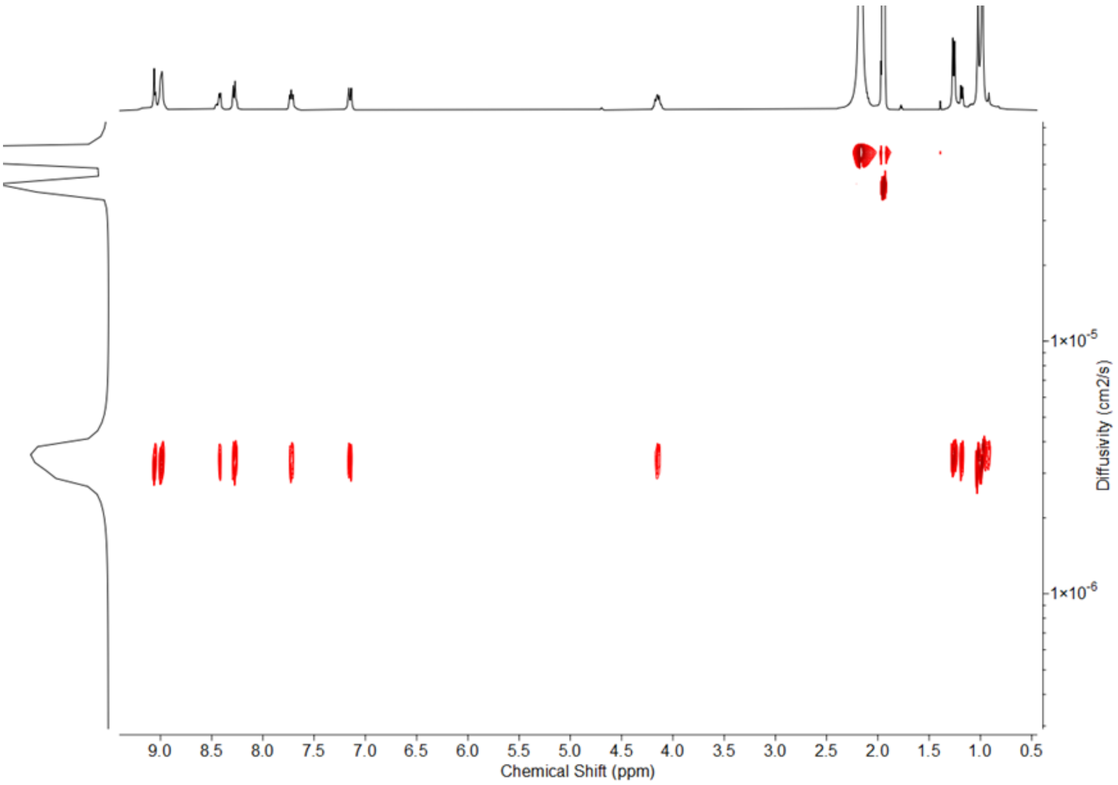

Figure 42. ^{1}H DOSY spectrum of $C_{60}\subset$**2** (400 MHz, CD_3CN, 25 °C). The diffusion coefficient for both diastereomers in CD_3CN was measured to be 3.45×10^{-6} cm^2/s.

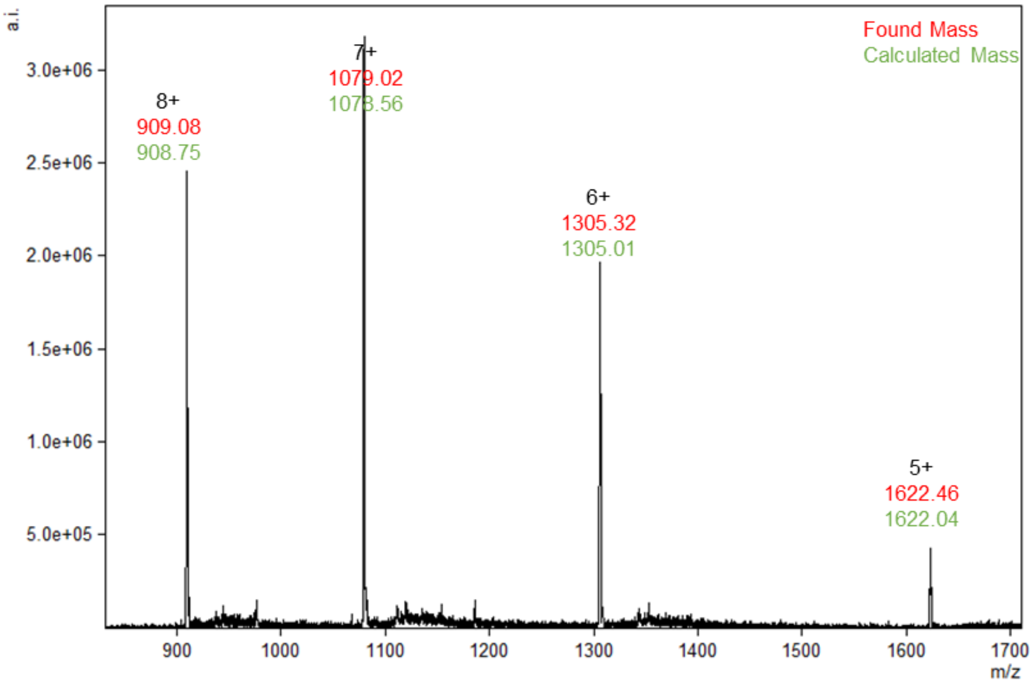

Figure S43. Low-resolution ESI-MS spectrum of $C_{60}\subset$**2** in MeCN.

$C_{60}PCBM\subset$**2** (8.9 mg, 92%) was prepared according to method **GP1**. The d.r. was determined to be 2.4:1 (Δ_4:Λ_4) by ^{1}H NMR.

^{1}H NMR (700 MHz, CD_3CN): δ 0.98 (s, 76.2H, $C_{60}PCBM\subset\Delta_4$-**2**), 0.99 (s, 31.8H, $C_{60}PCBM\subset\Lambda_4$-**2**), 1.15–1.21 (m, 8.3H, $C_{60}PCBM\subset\Lambda_4$-**2**), 1.22–1.31 (m, 27.7H, $C_{60}PCBM\subset\Delta_4$-**2**), 1.44–1.80 (m, 6H, $C_{60}PCBM$), 3.27 (s, 3H, $C_{60}PCBM$), 3.96–4.27 (m, 12H), 6.55–6.93 (m, 5H, $C_{60}PCBM$), 7.04–7.27 (m, 12H), 7.53–7.84 (m, 12H), 8.04–8.48 (m, 24H), 8.78–9.32 (m, 36H).

ESI-MS: m/z = 932.54 $[C_{60}PCBM\subset$**2**$\text{-}8(NTf_2)]^{8+}$, 1105.81 $[C_{60}PCBM\subset$**2**$\text{-}7(NTf_2)]^{7+}$, 1336.76 $[C_{60}PCBM\subset$**2**$\text{-}6(NTf_2)]^{6+}$, 1660.31 $[C_{60}PCBM\subset$**2**$\text{-}5(NTf_2)]^{5+}$.

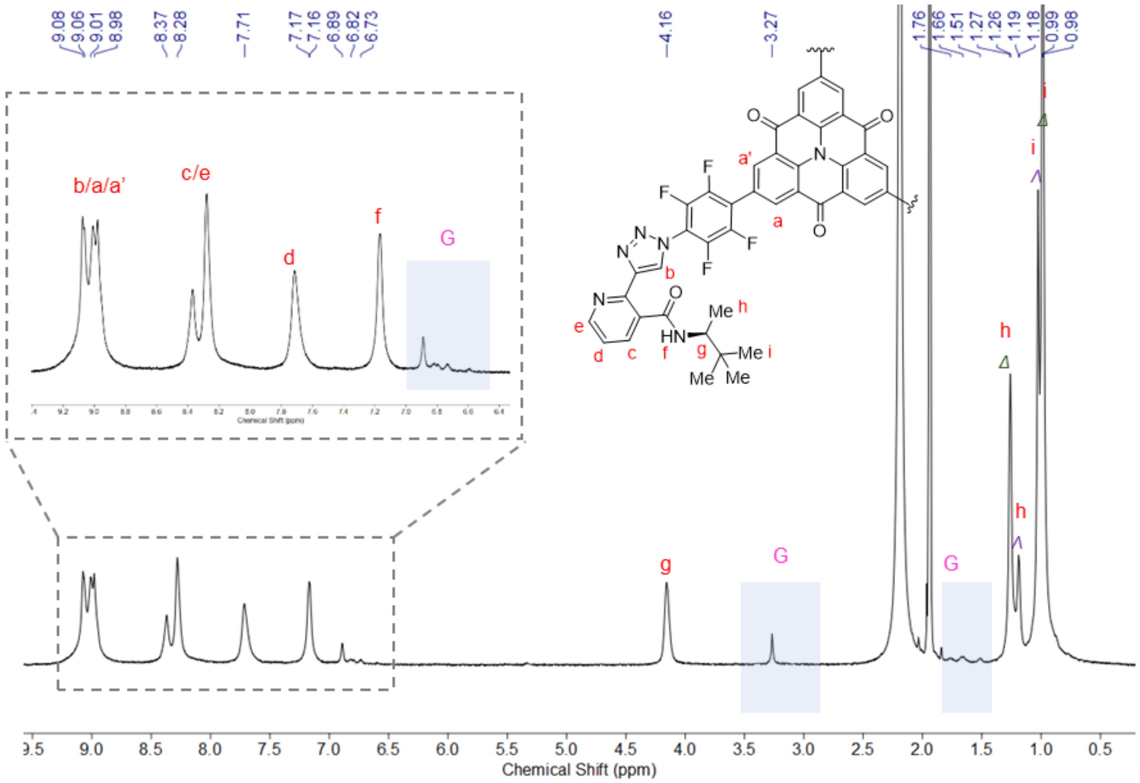

Figure S44. ^{1}H NMR spectrum of $C_{60}PCBM\subset$**2** (700 MHz, CD_3CN, 25 °C).

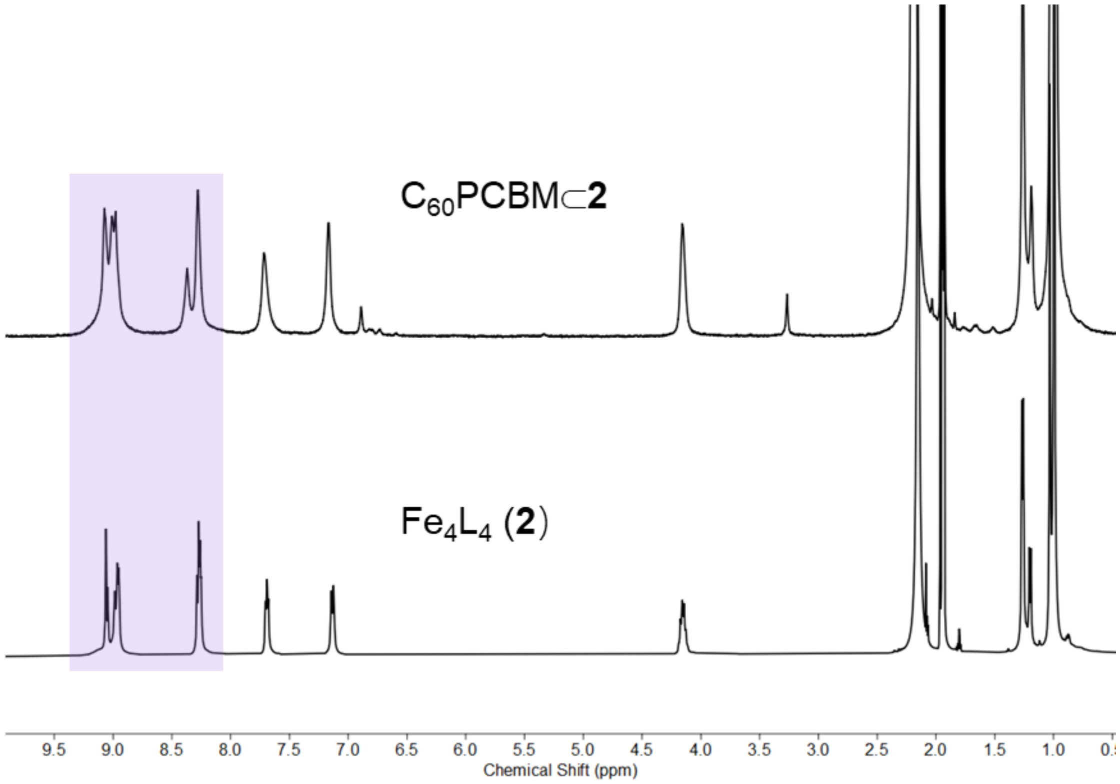

Figure S45. Comparison of the ^{1}H NMR spectra of **2** and C_{60}PCBM⊂**2**.

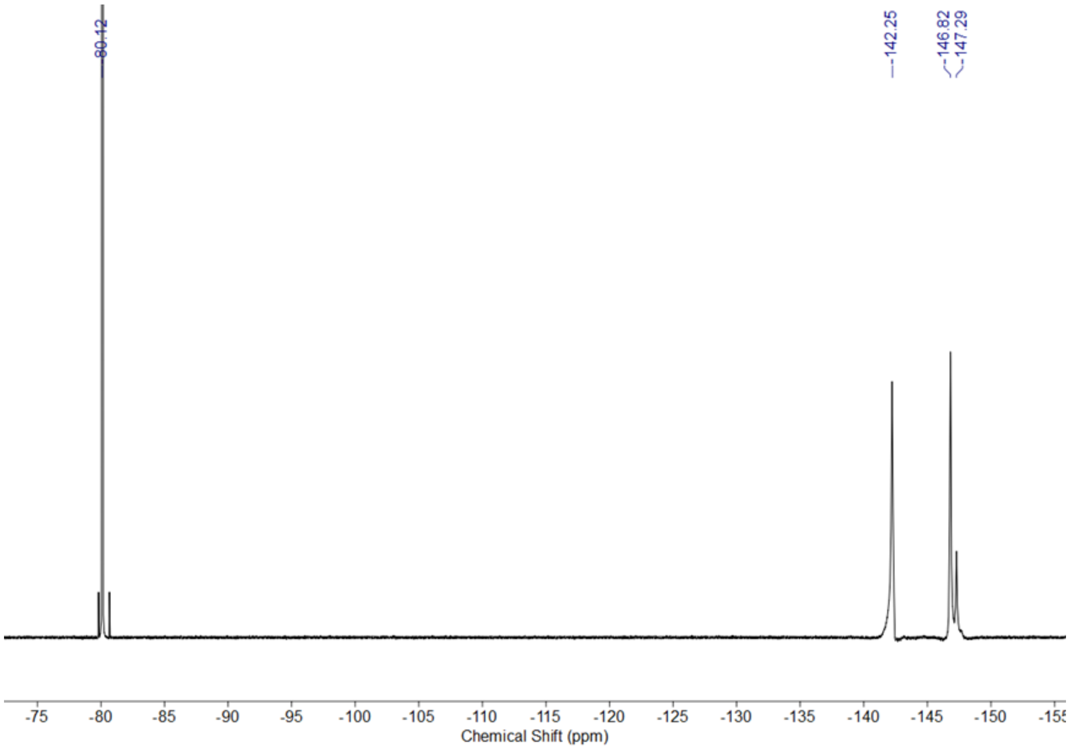

Figure S46. ^{19}F NMR spectrum of C_{60}PCBM⊂**2** (376 MHz, CD_3CN, 25 °C).

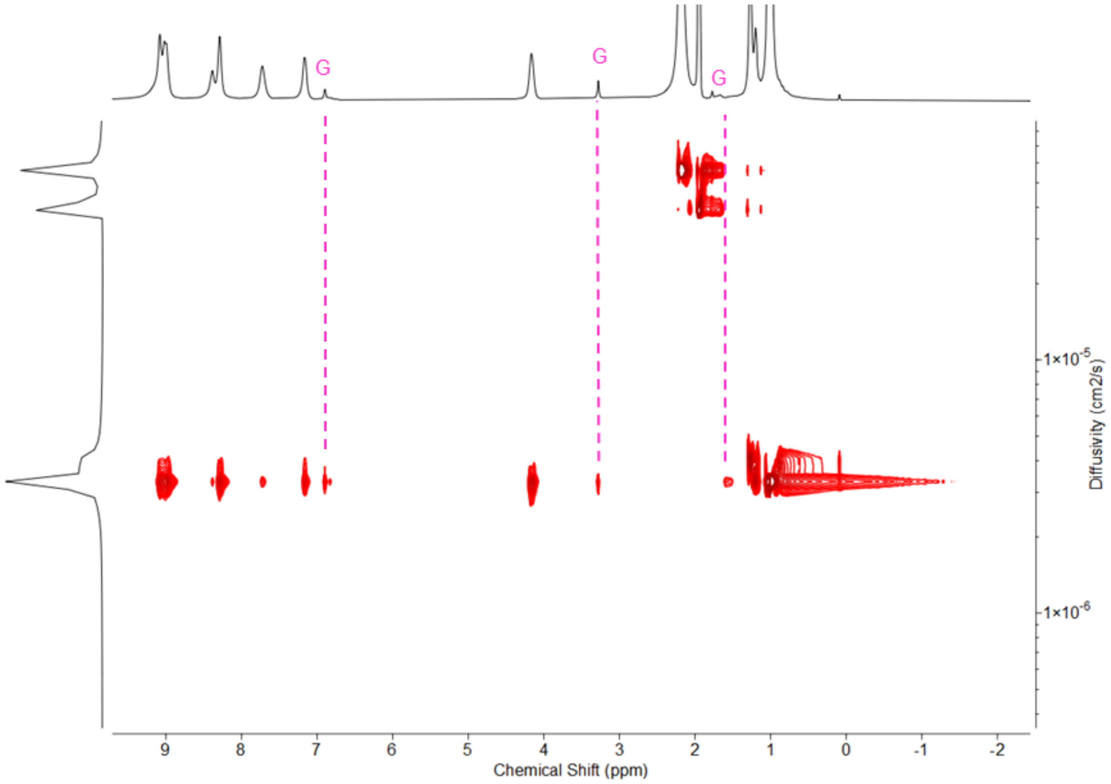

Figure 47. ^{1}H DOSY spectrum of C_{60}PCBM⊂**2** (400 MHz, CD_3CN, 25 °C). The diffusion coefficient for both diastereomers in CD_3CN was measured to be 3.31×10^{-6} cm^2/s.

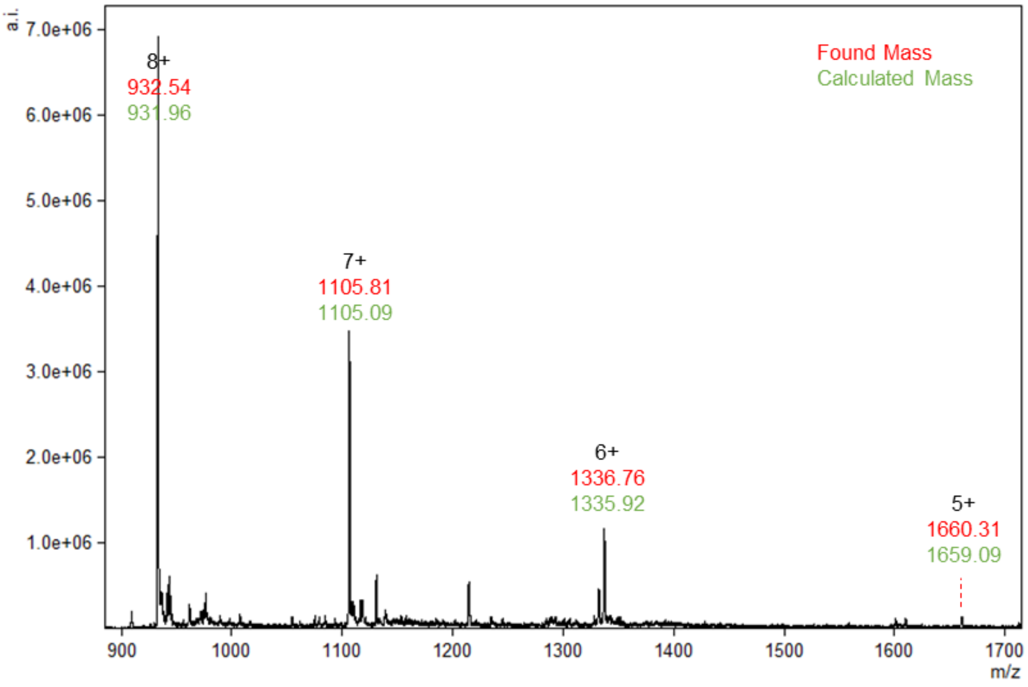

Figure S48. Low-resolution ESI-MS spectrum of C_{60}PCBM⊂**2** in MeCN.

Bis-C_{60}PCBM$\subset$**2** (9.4 mg, 95%) was prepared according to method **GP1**. Bis-C_{60}PCBM is mixture of different regioisomers. The d.r. was determined to be 1.7:1 (Δ_4:Λ_4) by ^{1}H NMR. After encapsulation, the signals of bound bis-C_{60}PCBM became broad; the following report ^{1}H NMR data only includes the peaks from the host cage.

^{1}H NMR (400 MHz, CD_3CN): δ 0.99 (s, 68H, C_{60}PCBM$\subset$$\Delta_4$-**2**), 1.03 (s, 40H, bis-C_{60}PCBM$\subset$$\Lambda_4$-**2**), 1.19 (d, J = 6.6 Hz, 22.7H, bis-C_{60}PCBM$\subset$$\Lambda_4$-**2**), 1.26 (d, J = 6.6 Hz, 23.6H, bis-C_{60}PCBM$\subset$$\Delta_4$-**2**), 4.06–4.23 (m, 12H), 7.07–7.26 (m, 12H), 7.59–7.77 (m, 12H), 8.15–8.42 (m, 24H), 8.88–9.31 (m, 36H).

ESI-MS: m/z = 956.40 [bis-C_{60}PCBM$\subset$**2**-8(NTf_2)]$^{8+}$, 1133.08 [bis-C_{60}PCBM$\subset$**2**-7(NTf_2)]$^{7+}$, 1368.60 [bis-C_{60}PCBM$\subset$**2**-6(NTf_2)]$^{6+}$, 1698.65 [bis-C_{60}PCBM$\subset$**2**-5(NTf_2)]$^{5+}$.

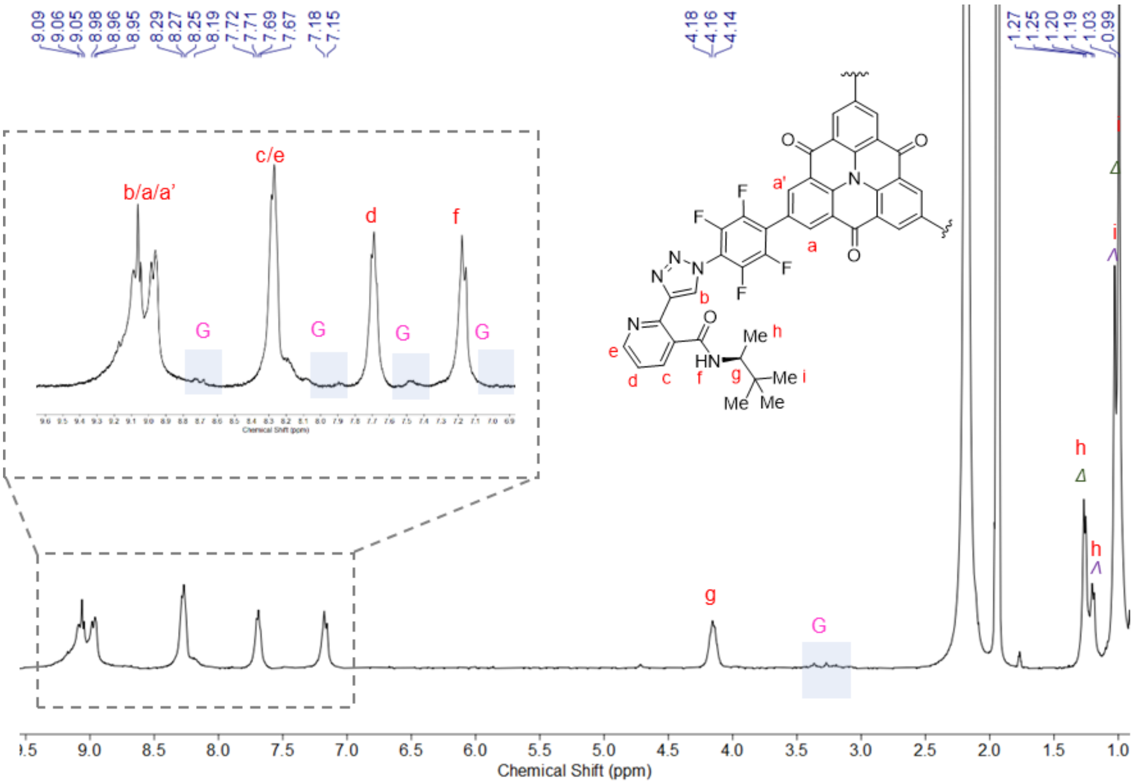

Figure S49. ^{1}H NMR spectrum of bis-C_{60}PCBM$\subset$**2** (400 MHz, CD_3CN, 25 °C). Signals from the encapsulated guest are highlighted.

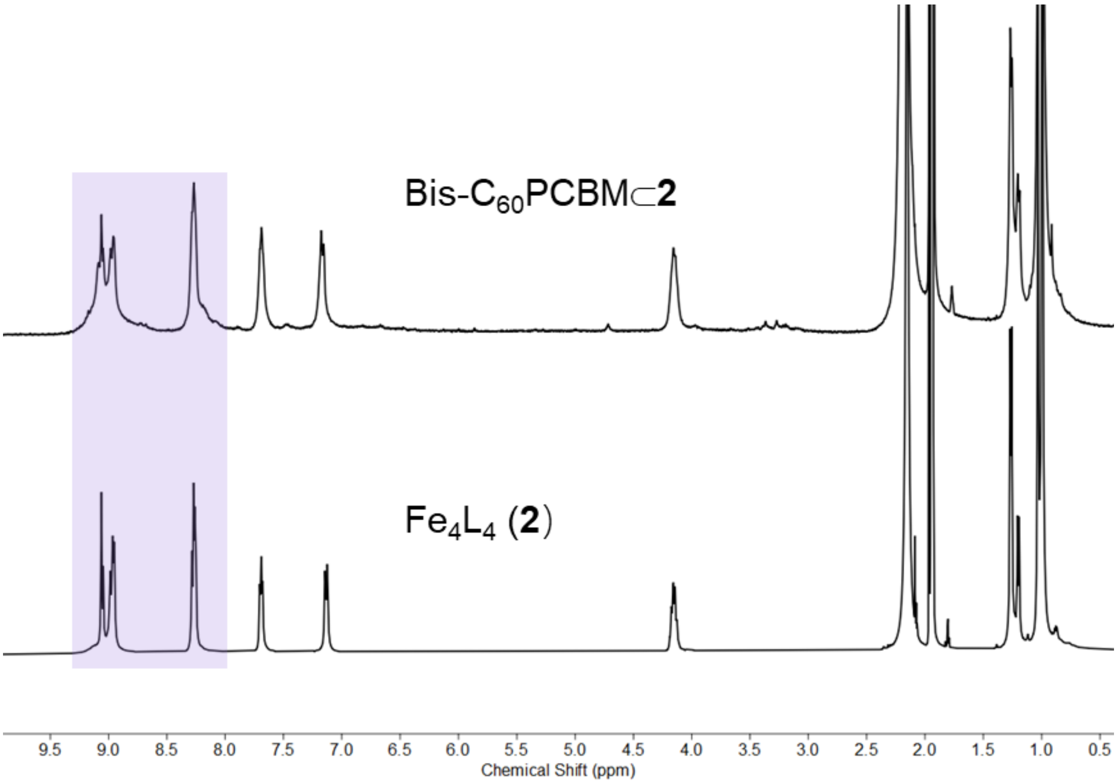

Figure S50. Comparison of the ^{1}H NMR spectra of **2** and bis-C_{60}PCBM⊂**2**.

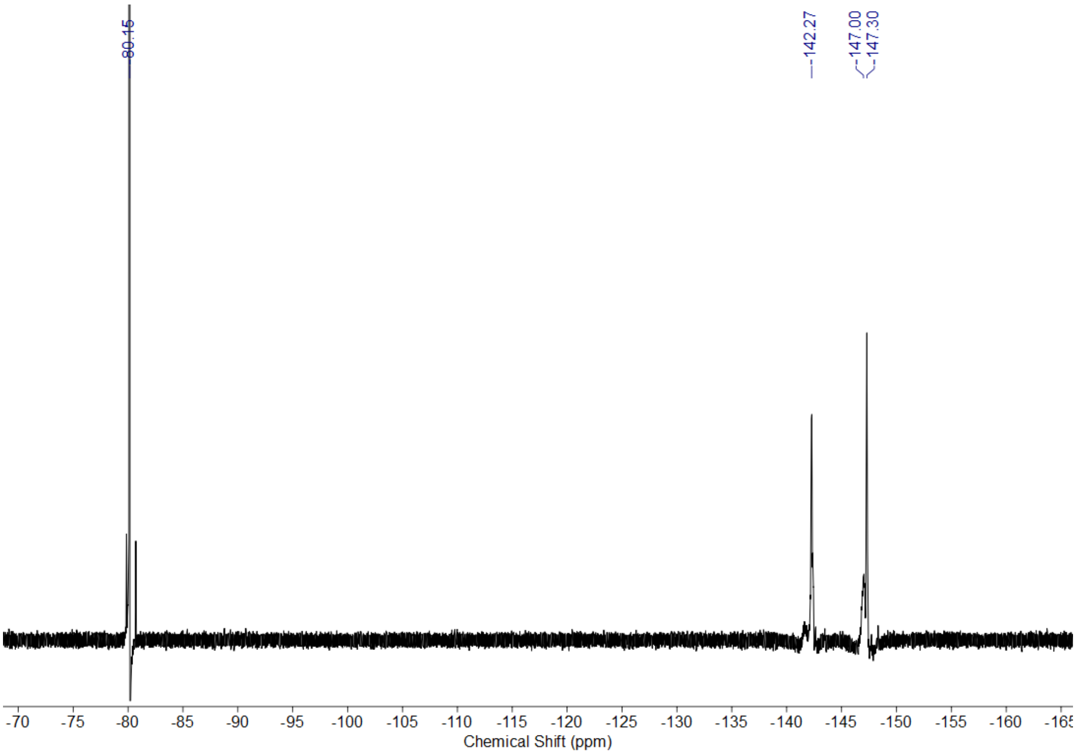

Figure S51. ^{19}F NMR spectrum of C_{60}PCBM⊂**2** (376 MHz, CD_3CN, 25 °C).

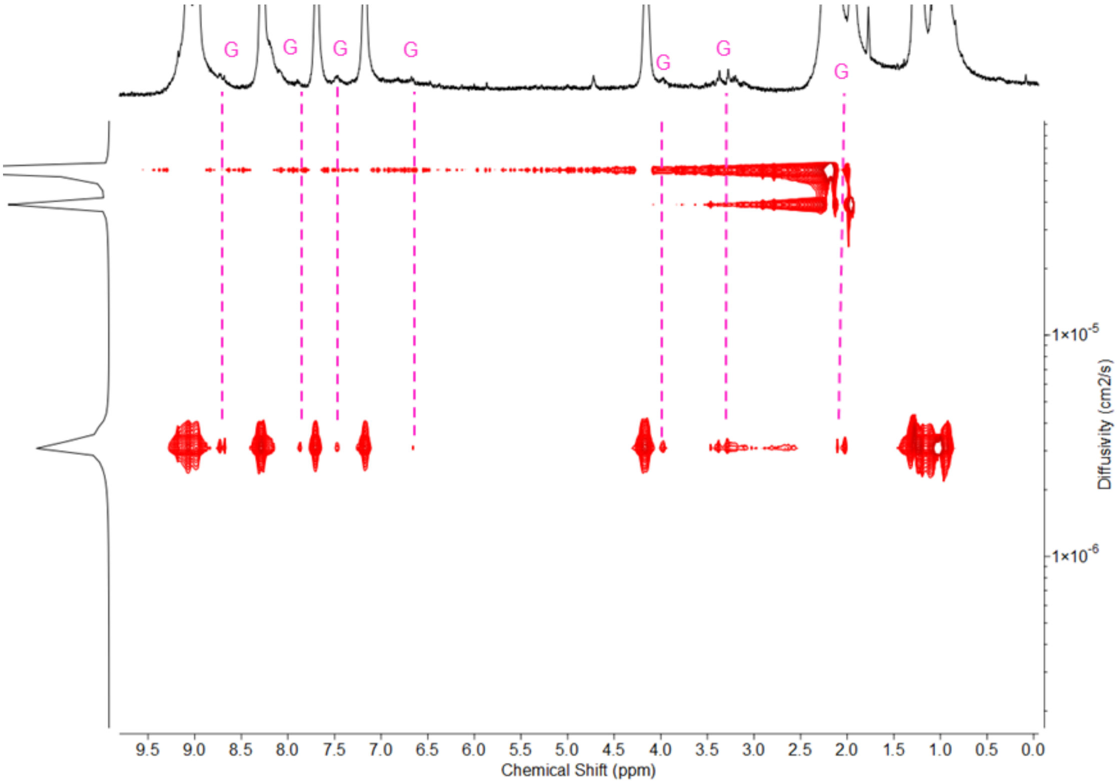

Figure 52. ^{1}H DOSY spectrum of bis-C_{60}PCBM⊂**2** (400 MHz, CD_3CN, 25 °C). The diffusion coefficient for all diastereomers in CD_3CN was measured to be 3.08×10^{-6} cm^2/s.

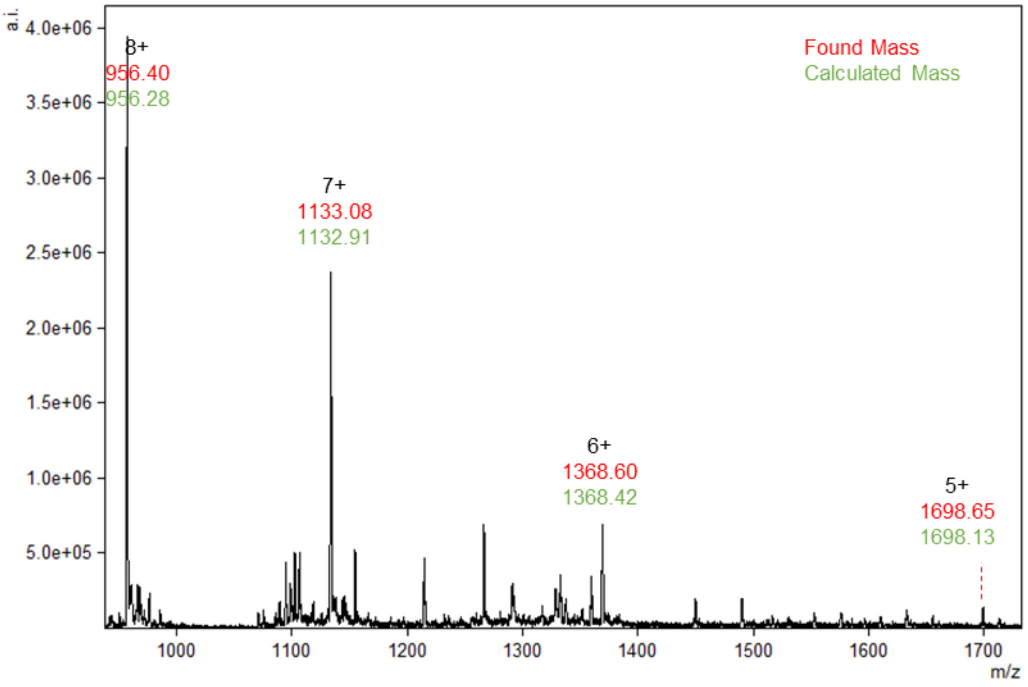

Figure S53. Low-resolution ESI-MS spectrum of bis-C_{60}PCBM⊂**2** in MeCN.

$IC_{60}MA \subset$ **2** (8.3 mg, 86%) was prepared according to method **GP1**. The d.r. was determined to be 2.8:1 (Δ_4:Λ_4) by ^{1}H NMR. After encapsulation, the signals of bound $IC_{60}MA$ became broad; the following report ^{1}H NMR data only includes the peaks from the host cage.

^{1}H NMR (400 MHz, CD_3CN): δ 1.00 (s, 80H, $IC_{60}MA \subset \Delta_4$-**2**), 1.03 (s, 28H, $IC_{60}MA \subset \Lambda_4$-**2**), 1.20 (d, *J* = 6.9 Hz, 9.5H, $IC_{60}MA \subset \Lambda_4$-**2**), 1.27 (d, *J* = 6.8 Hz, 26.5H, $IC_{60}MA \subset \Delta_4$-**2**), 4.08–4.23 (m, 12H), 7.14 (d, *J* = 9.3 Hz, 12H), 7.60–7.75 (m, 12H), 8.13–8.39 (m, 24H), 8.78–9.31 (m, 36H).

HR-ESI-MS: m/z = 923.2085 [$IC_{60}MA \subset$ **2**-8(NTf_2)]$^{8+}$, 1095.0814 [$IC_{60}MA \subset$ **2**-7(NTf_2)]$^{7+}$, 1324.4145 [$IC_{60}MA \subset$ **2**-6(NTf_2)]$^{6+}$, 1646.0933 [$IC_{60}MA \subset$ **2**-5(NTf_2)]$^{5+}$, 2126.3550 [$IC_{60}MA \subset$ **2**-4(NTf_2)]$^{4+}$.

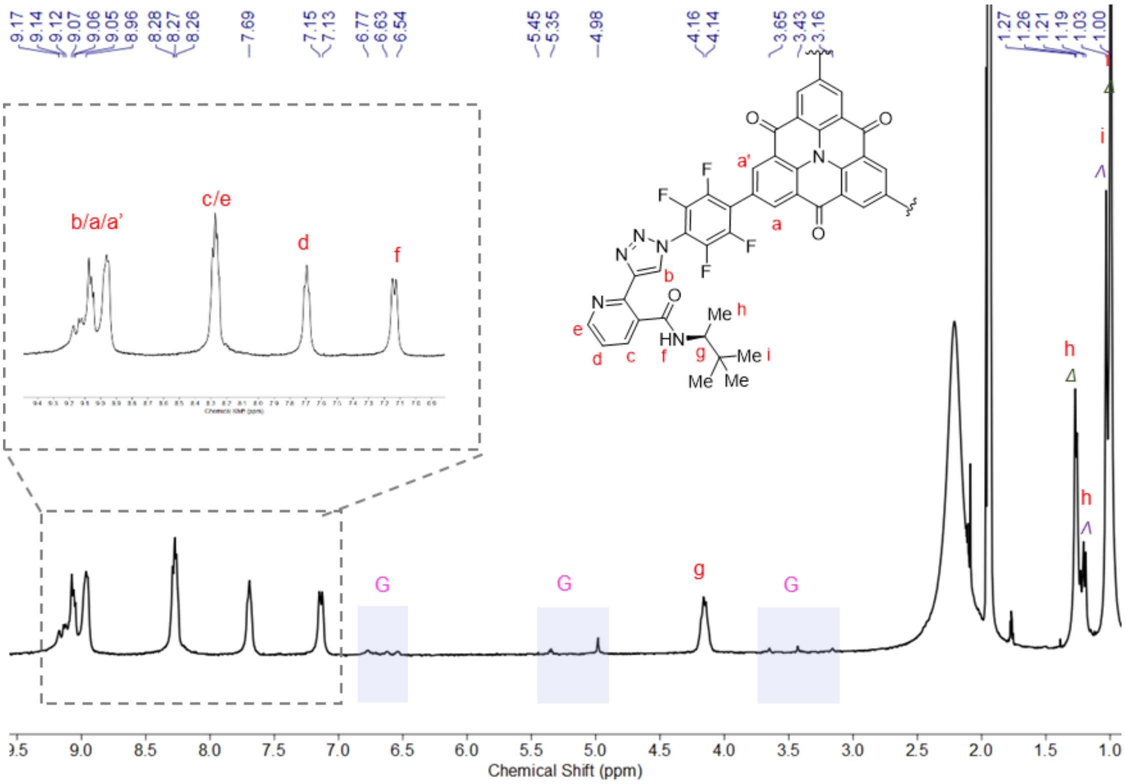

Figure S54. ^{1}H NMR spectrum of $IC_{60}MA \subset$ **2** (400 MHz, CD_3CN, 25 °C).

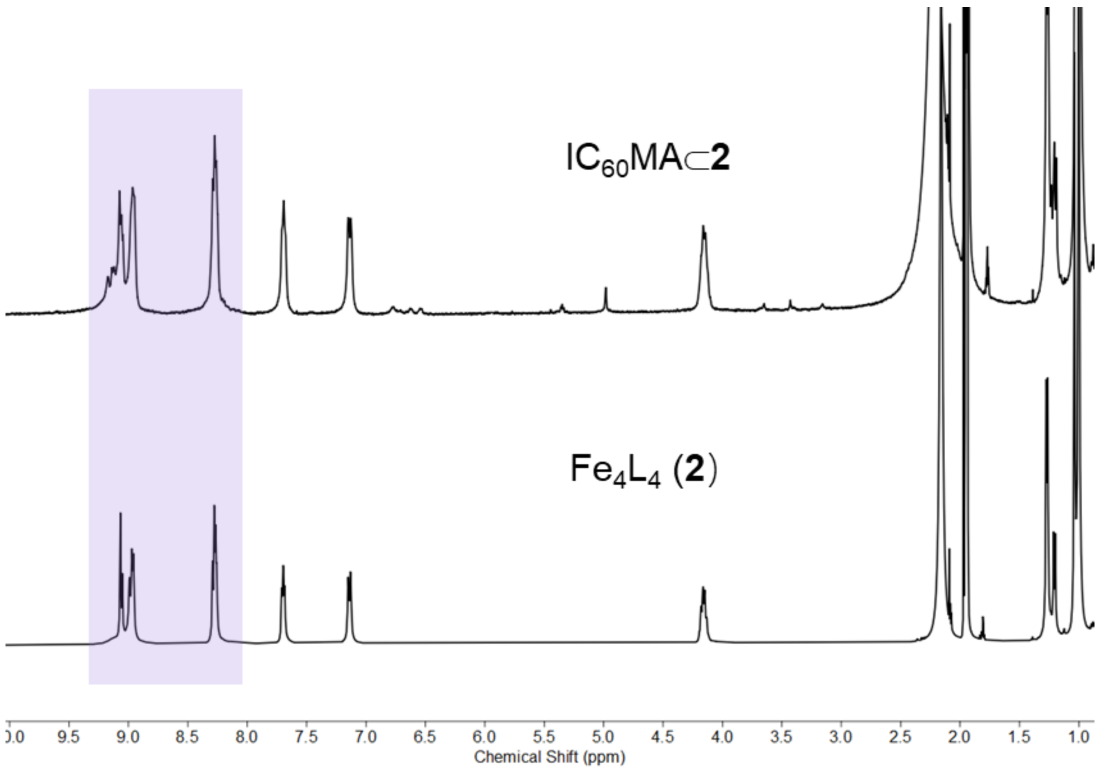

Figure S55. Comparison of the ^{1}H NMR spectra of **2** and IC$_{60}$MA⊂**2**.

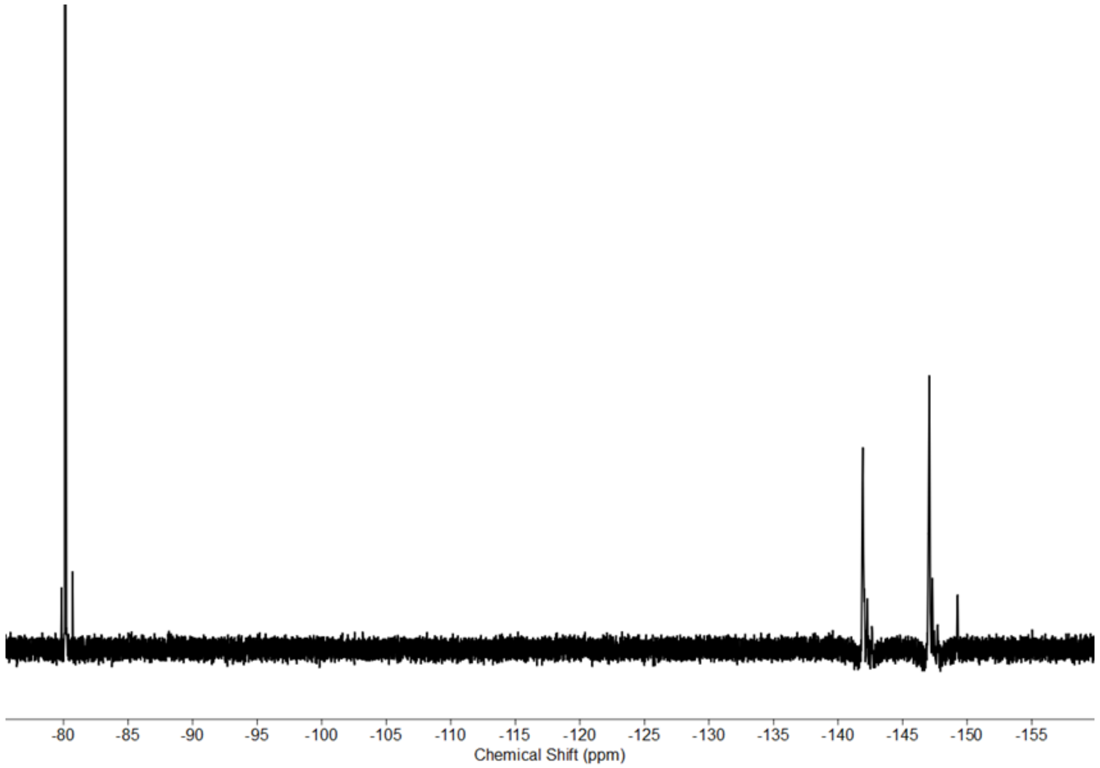

Figure S56. ^{19}F NMR spectrum of IC$_{60}$MA⊂**2** (376 MHz, CD_3CN, 25 °C).

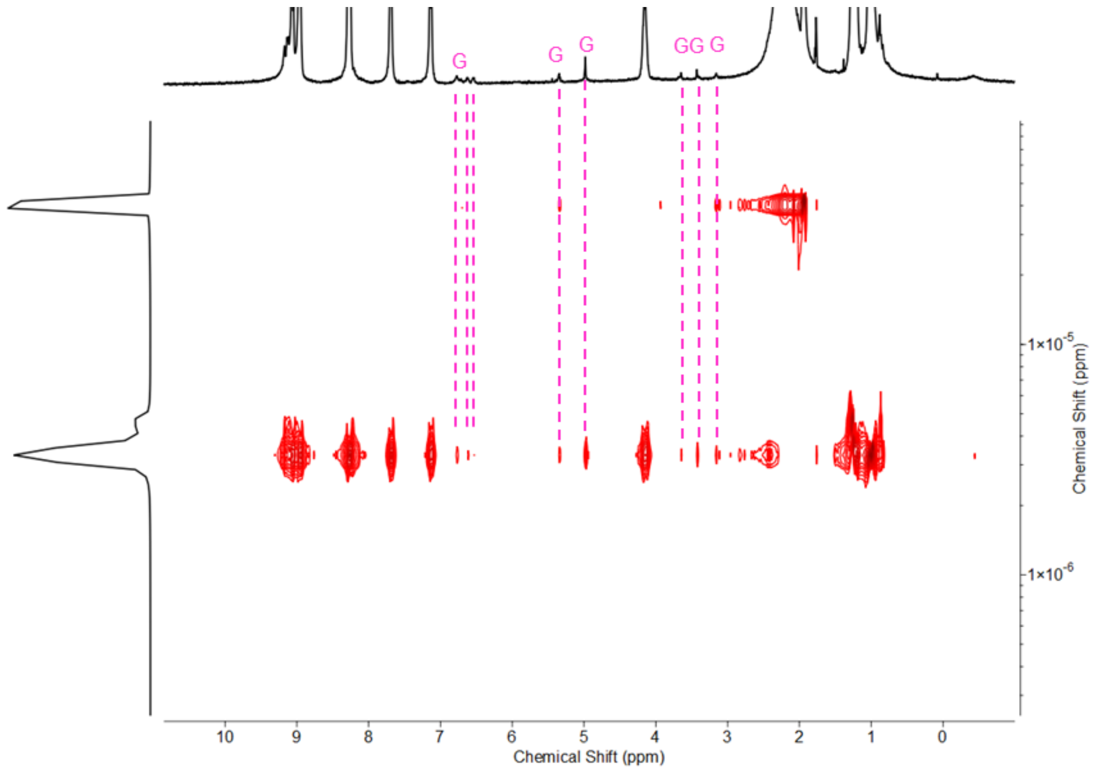

Figure 57. ^{1}H DOSY spectrum $IC_{60}MA \subset$**2** (400 MHz, CD_3CN, 25 °C). The diffusion coefficient for both diastereomers in CD_3CN was measured to be 3.28×10^{-6} cm^2/s.

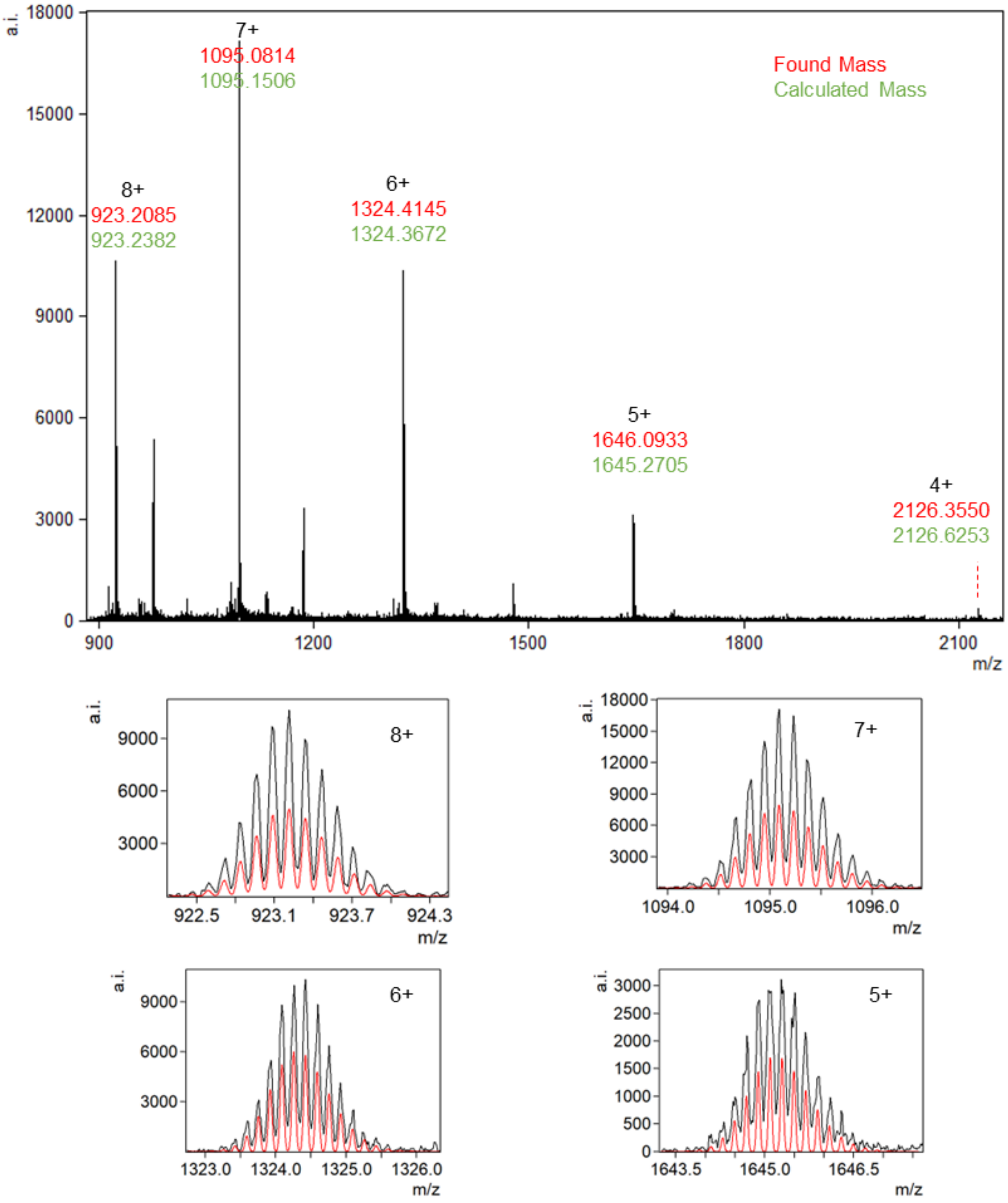

Figure S58. High-resolution ESI-MS spectrum of $IC_{60}MA \subset$**2** in MeCN. Two sets of peaks were observed in the spectrum; the other set correspond to empty cage **2**.

$C_{70}\subset$**2** (8.6 mg, 89%) was prepared according to method **GP1**. The d.r. was determined to be 4.1:1 (Δ_4:Λ_4) by ^{1}H NMR.

^{1}H NMR (400 MHz, CD_3CN): δ 0.98 (s, 87H, $C_{70}\subset\Delta_4$-**2**), 1.02 (s, 21H, $C_{70}\subset\Lambda_4$-**2**), 1.18 (d, *J* = 6.6 Hz, 7H, $C_{70}\subset\Lambda_4$-**2**), 1.26 (d, *J* = 6.6 Hz, 29H, $C_{70}\subset\Delta_4$-**2**), 4.07–4.20 (m, 12H), 7.15 (d, *J* = 9.6 Hz, 12H), 7.71 (dd, *J* = 7.7, 5.7 Hz, 12H), 8.22–8.30 (m, 12H), 8.31–8.39 (m, 12H), 8.94–9.00 (m, 24H), 9.03 (s, 2.3H, $C_{70}\subset\Lambda_4$-**2**), 9.05 (s, 9.7H, $C_{70}\subset\Delta_4$-**2**).

ESI-MS: m/z = 924.19 $[C_{70}\subset$**2**$-8(NTf_2)]^{8+}$, 1096.20 $[C_{70}\subset$**2**$-7(NTf_2)]^{7+}$, 1325.42 $[C_{70}\subset$**2**$-6(NTf_2)]^{6+}$, 1646.45 $[C_{70}\subset$**2**$-5(NTf_2)]^{5+}$.

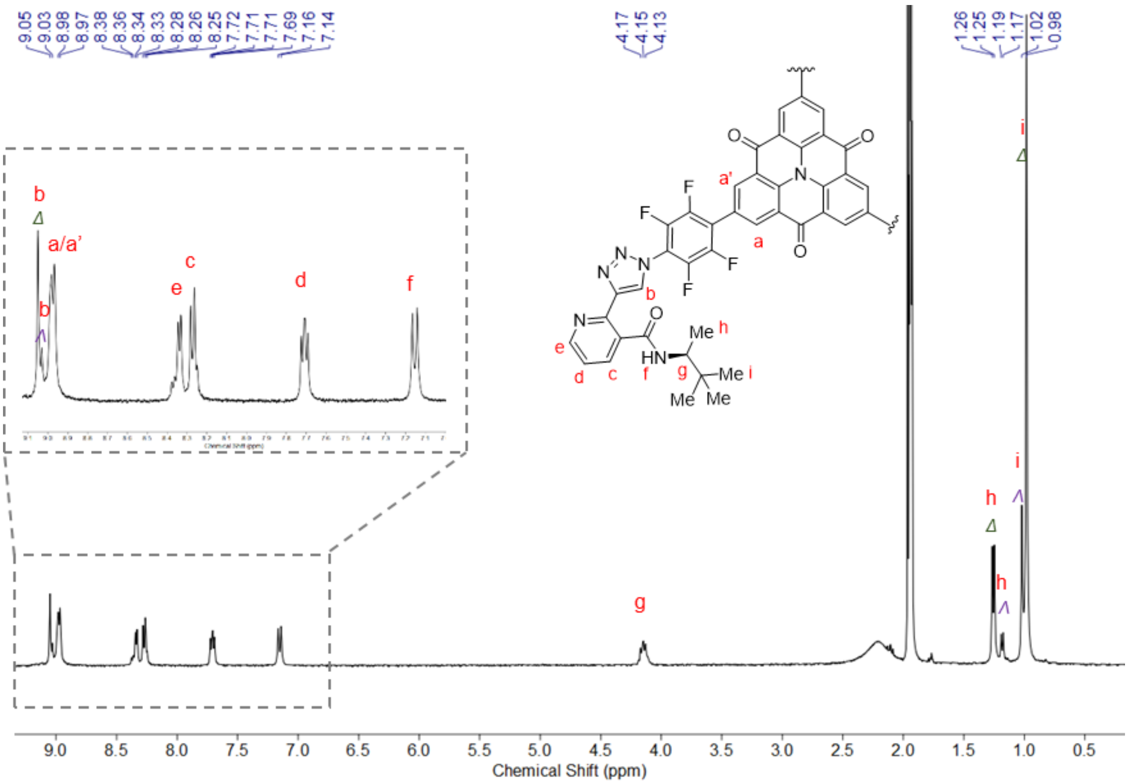

Figure S59. ^{1}H NMR spectrum of $C_{70}\subset$**2** (400 MHz, CD_3CN, 25 °C).

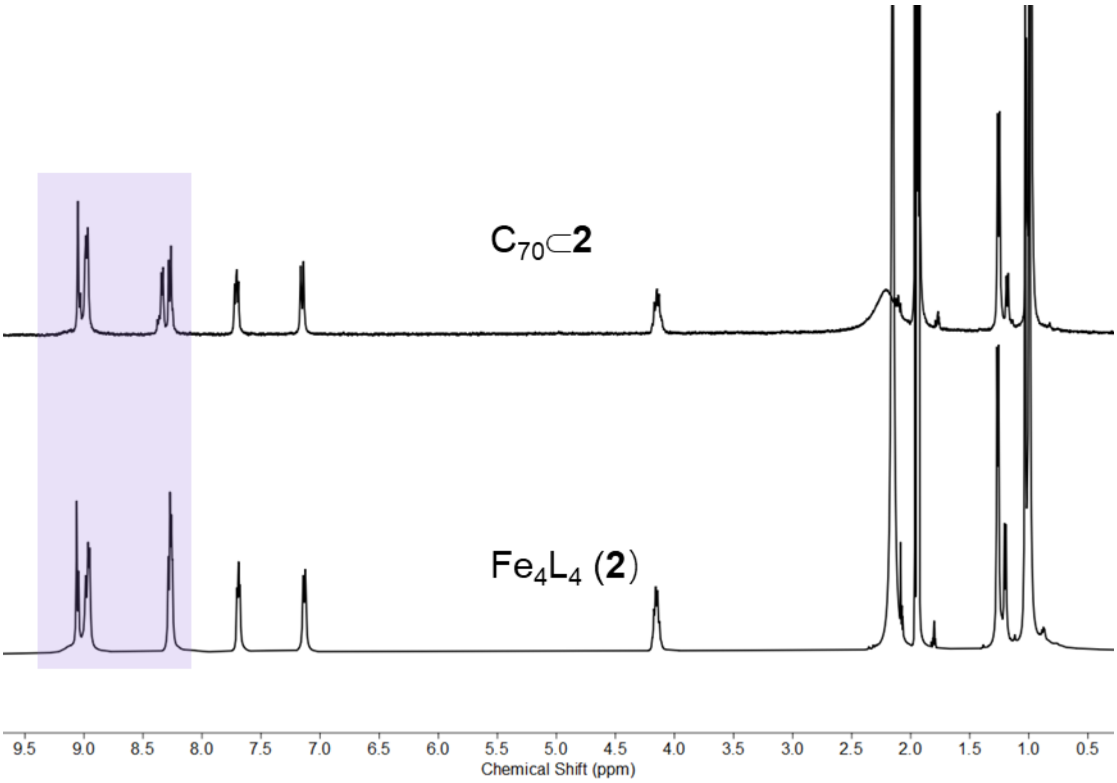

Figure S60. Comparison of the ^{1}H NMR spectra of **2** and C_{70}⊂**2**.

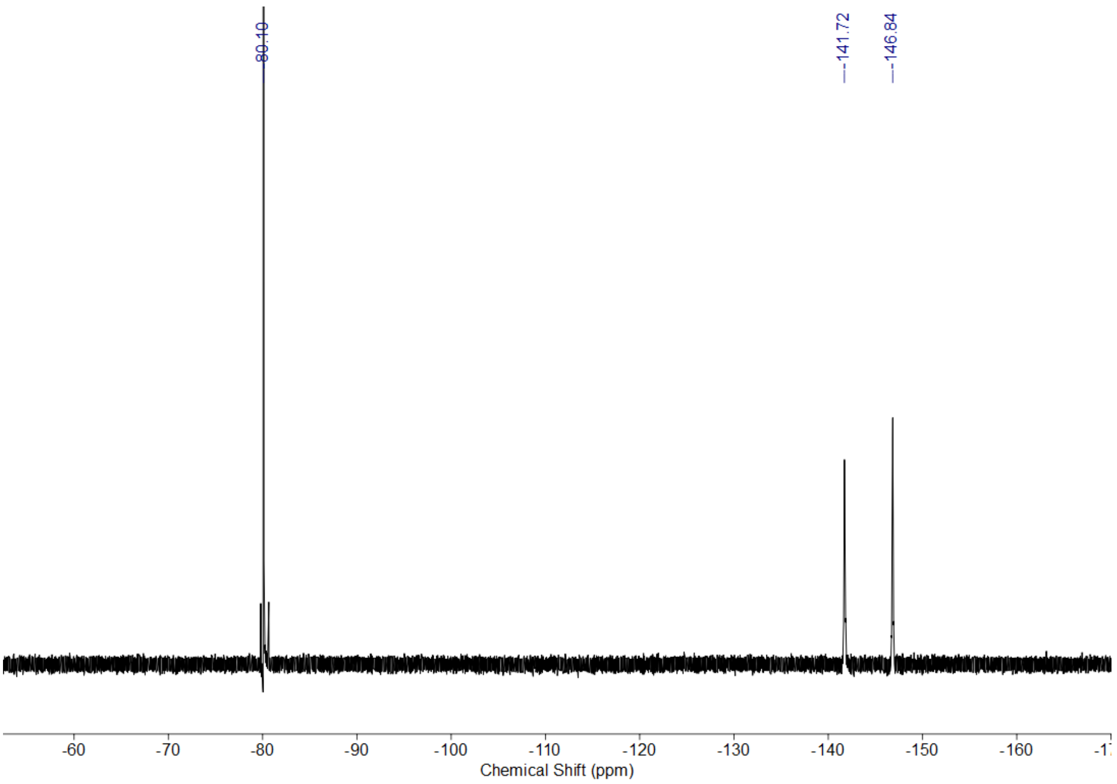

Figure S61. ^{19}F NMR spectrum of C_{70}⊂**2** (376 MHz, CD_3CN, 25 °C).

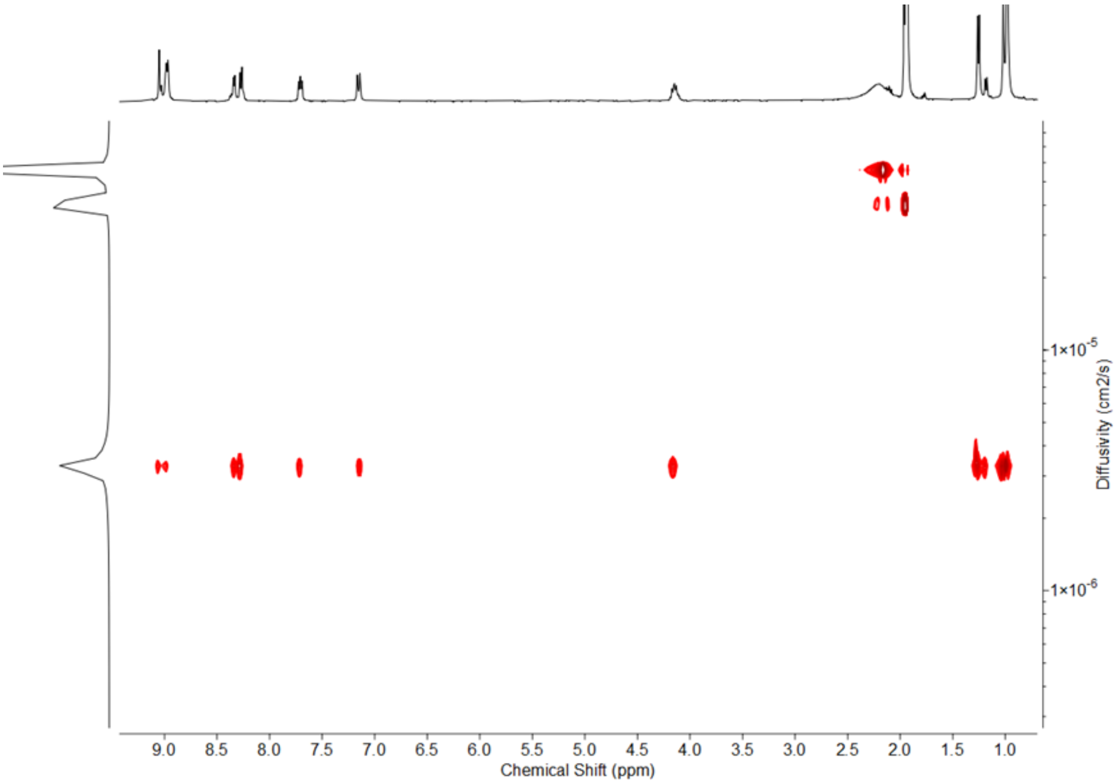

Figure 62. ^{1}H DOSY spectrum $C_{70}\subset$**2** (400 MHz, CD_3CN, 25 °C). The diffusion coefficient for both diastereomers in CD_3CN was measured to be 3.30×10^{-6} cm^2/s.

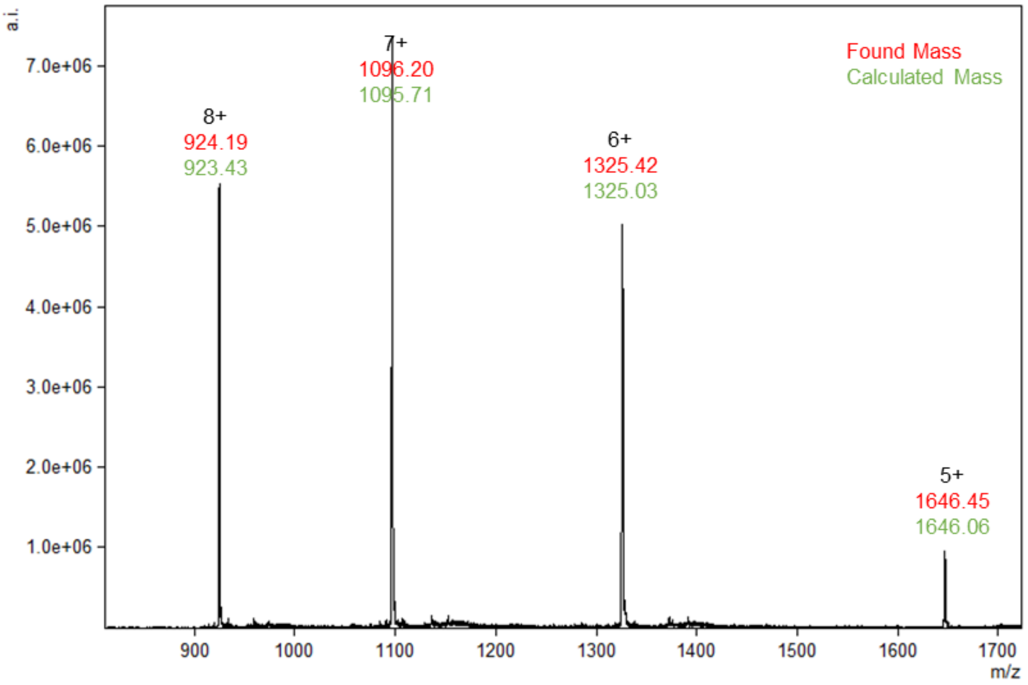

Figure S63. Low-resolution ESI-MS spectrum of $C_{70}\subset$**2** in MeCN.

C_{70}PCBM⊂**2** (9.1 mg, 90%) was prepared according to method **GP1**. C_{70}PCBM is mixture of different regioisomers, with α-C_{70}PCBM as a major isomer (ca. 85%). The d.r. was determined to be 3.6:1 (Δ_4:Λ_4) by ^{1}H NMR.

^{1}H NMR (400 MHz, CD_3CN): δ 0.98 (s, 84.5H, C_{70}PCBM⊂Δ_4-**2**), 1.02 (s, 23.5H, C_{70}PCBM⊂Λ_4-**2**), 1.18 (d, *J* = 6.8 Hz, 7.8H, C_{70}PCBM⊂Λ_4-**2**), 1.26 (d, *J* = 6.8 Hz, 28.2H, C_{70}PCBM⊂Δ_4-**2**), 1.69–2.20 (m, 6H, C_{70}PCBM), 3.00–3.38 (m, 3H, C_{70}PCBM),, 4.11–4.20 (m, 12H), 6.95–7.60 (m, 5H, C_{70}PCBM), 7.17 (d, *J* = 9.7 Hz, 12H), 7.62–7.78 (m, 12H), 8.16–8.43 (m, 24H), 8.87–9.21 (m, 36H).

ESI-MS: m/z = 947.65 [C_{70}PCBM⊂**2**-8(NTf_2)]$^{8+}$, 1123.04 [C_{70}PCBM⊂**2**-7(NTf_2)]$^{7+}$, 1356.85 [C_{70}PCBM⊂**2**-6(NTf_2)]$^{6+}$, 1684.34 [C_{70}PCBM⊂**2**-5(NTf_2)]$^{5+}$.

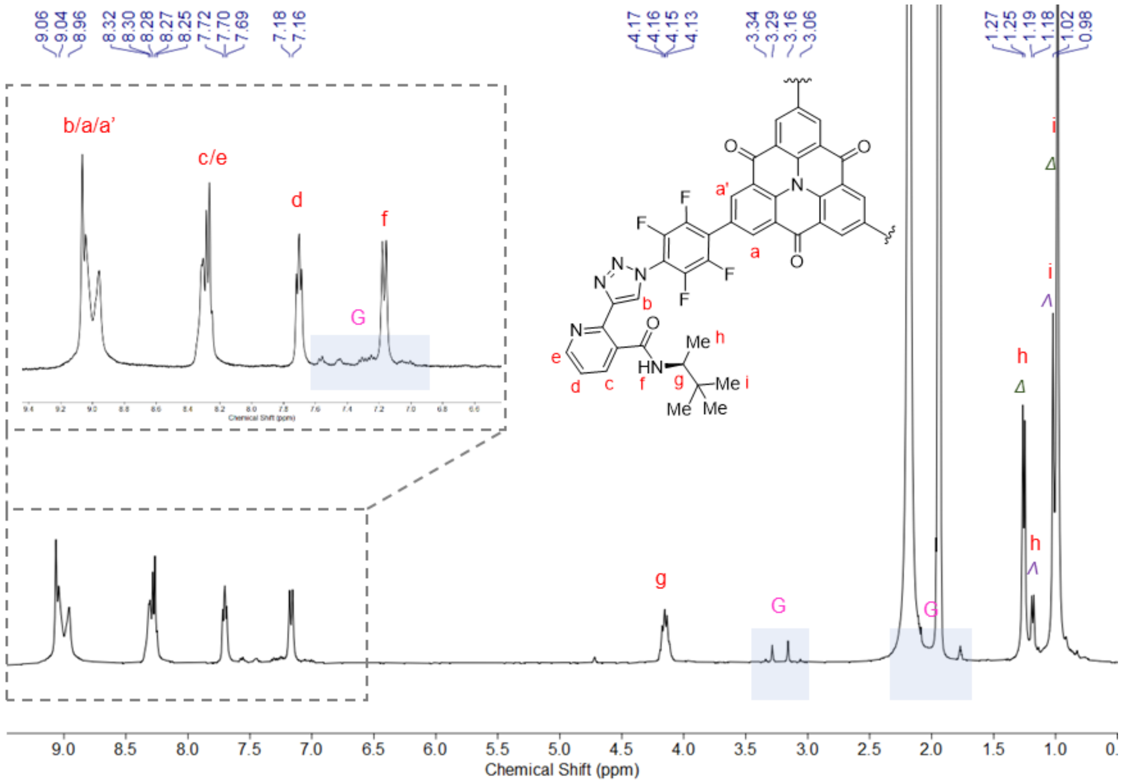

Figure S64. ^{1}H NMR spectrum of C_{70}PCBM⊂**2** (400 MHz, CD_3CN, 25 °C).

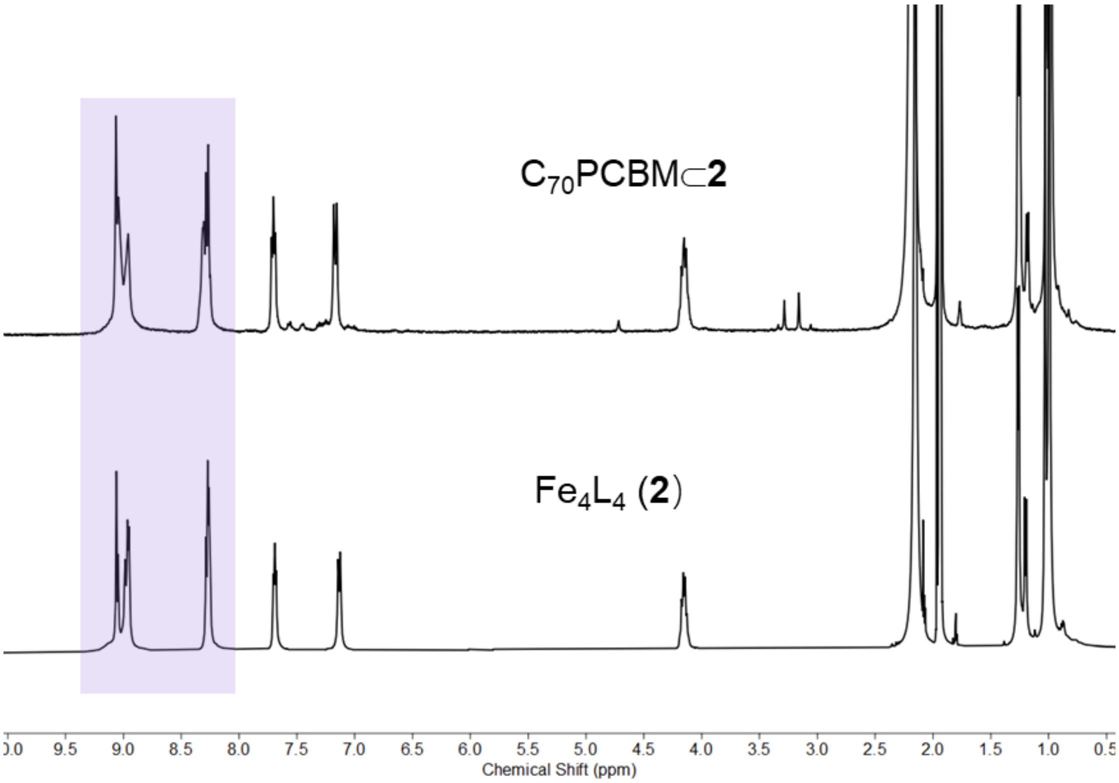

Figure S65. Comparison of the ^{1}H NMR spectra of **2** and C_{70}PCBM⊂**2**.

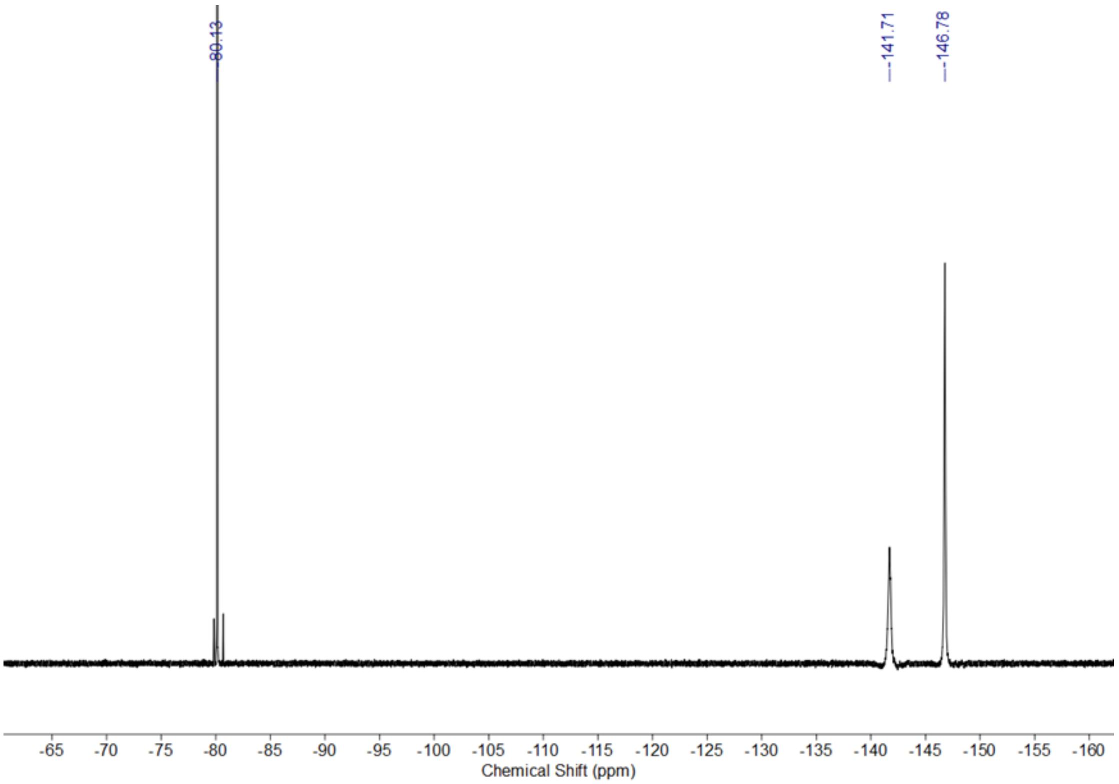

Figure S66. ^{19}F NMR spectrum of C_{70}PCBM⊂**2** (376 MHz, CD_3CN, 25 °C).

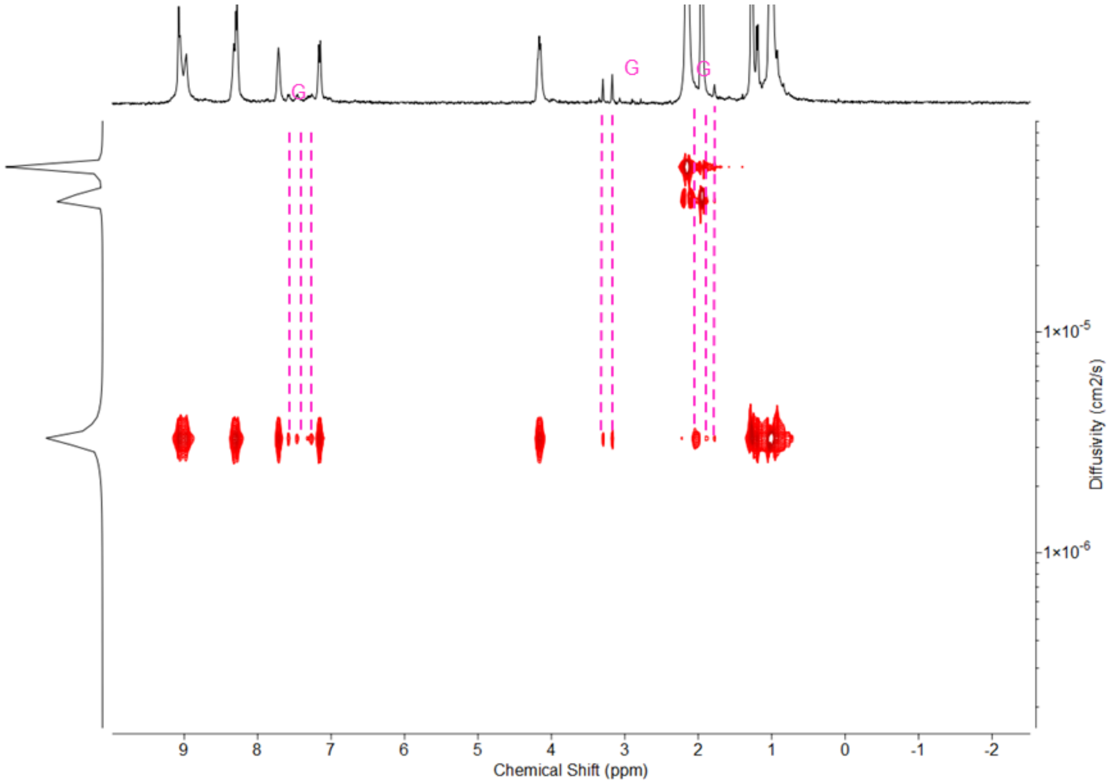

Figure 67. ^{1}H DOSY spectrum C_{70}PCBM⊂**2** (400 MHz, CD_3CN, 25 °C). The diffusion coefficient for all diastereomers in CD_3CN was measured to be 3.32×10^{-6} cm^2/s.

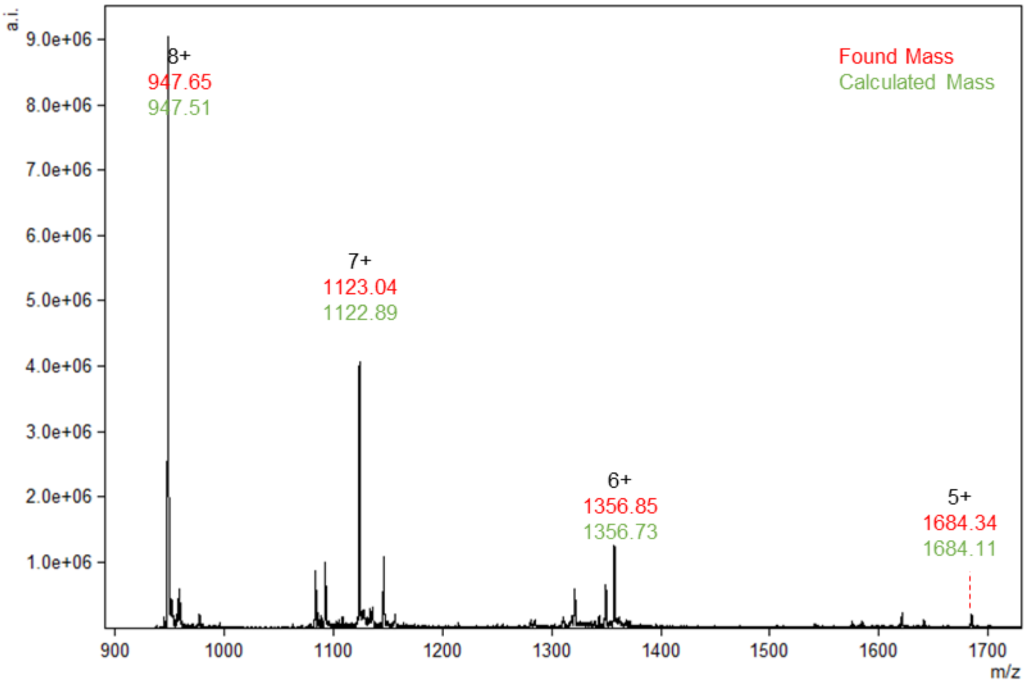

Figure S68. Low-resolution ESI-MS spectrum of C_{70}PCBM⊂**2** in MeCN.

^{1}H NMR and MS spectra confirmed that both Δ_4-**2** and Λ_4-**2** were able to encapsulate guests, with **G**$\subset\Delta_4$-**2** as the major species and **G**$\subset\Lambda_4$-**2** as the minor species, as confirmed by CD spectra of host-guest complexes.

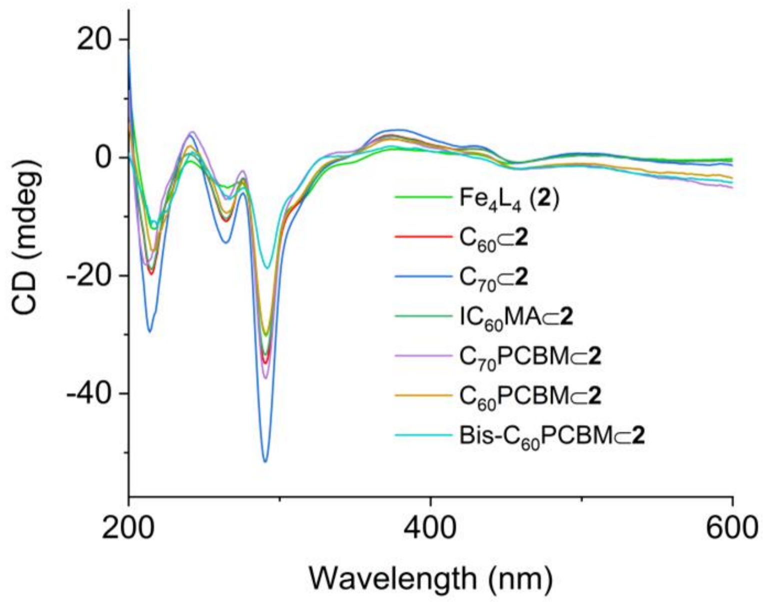

Figure S69. CD spectra of **2** and **G**$\subset$**2** at the same concentration in MeCN.

We also observed $\Delta_4 \rightleftarrows \Lambda_4$ interconversion upon encapsulation, with the equilibrium position governed by guests. The encapsulation of guests resulted in changes in the energy difference between the Δ_4 and Λ_4 configurations. The energy difference between the Δ_4 and Λ_4 configurations can be expressed by the standard Gibbs free energy change (ΔG^o), using the following equation (Equation 1):

$$\Delta G^o = -RT\ln([\Lambda_4\text{-}\mathbf{2}]/[\Delta_4\text{-}\mathbf{2}]) \qquad \text{(Eq 1)}$$

where R is the gas constant (8.314 J mol^{-1} K^{-1}), T is the operating temperature (298 K) and $[\Lambda_4\text{-}\mathbf{2}]/[\Delta_4\text{-}\mathbf{2}]$ is the concentration ratio of Λ_4-**2** to Δ_4-**2**. The concentration ratio corresponds to the diastereoselectivity, which was determined by ^{1}H NMR integration. The diastereomeric ratios and standard Gibbs free energy changes of **2** and its host-guest complexes are shown in Table S1.

Table S1. Diastereomeric ratios and standard Gibbs energy changes.

Cage and Host-Guest Complex	d.r. = Δ_4:Λ_4	$\Delta G°$ (kJ mol^{-1})
**2**	2.4:1	2.17
C_{60}⊂**2**	3.3:1	2.96
IC_{60}MA⊂**2**	2.8:1	2.55
C_{60}PCBM⊂**2**	2.4:1	2.17
Bis-C_{60}PCBM⊂**2**	1.8:1	1.46
C_{70}⊂**2**	4.1:1	3.50
C_{70}PCBM⊂**2**	3.6:1	3.17

The cavity volumes of Δ_4-**2** and Λ_4-**2** were calculated to be1281 Å^3 for and 1266 Å^3, respectively (Figure S107). The van der Waals volumes V_{vdw} and molecular volumes V_{mol} (including cavities) of the fullerenes and their adducts were calculated based on reported crystal structures (C_{60},[6] C_{60}PCBM,[7] C_{70},[8] and C_{70}PCBM[9]) or MM3-optimized molecular models (IC_{60}MA and bis-C_{60}PCBM), using the MoloVol program.[10]

Considering the molecular volume V_{mol} when comparing sizes of fullerenes and their adducts is crucial, because their cavities will not appear in their van der Waals volumes but have a considerable contribution to their sizes. We therefore decide to use V_{mol} to investigate the guest-induced $\Delta_4 \rightleftarrows \Lambda_4$ interconversion. The molecular volumes of the investigated guests increase in the order of C_{60} < IC_{60}MA < C_{70} < C_{60}PCBM < C_{70}PCBM < bis-C_{60}PCBM (Table S2).

Tetrahedron cage generally has near-spherical inner cavity (Figure S107). To further investigate the impact of shape of guest molecules on $\Delta_4 \rightleftarrows \Lambda_4$ interconversion, we herein introduce the sphericity Ψ (Equation S2)[11]:

$$\Psi = \pi^{1/3} \cdot (6V_{\text{mol}})^{2/3}/S_{\text{excl}} \qquad \text{(Eq 1)}$$

where V_{mol} is the molecular volume and S_{excl} is the probe excluded molecular surface. The V_{vdw}, V_{mol}, S_{excl} and Ψ of guest molecules were shown in Table S2.

Table S2. Calculated volumes of the fullerenes and their adducts by MoloVol.

Guest	V_{vdw} (Å^3)	V_{mol} (Å^3)	S_{excl} (Å^2)	Ψ
C_{60}	529	554	386	0.85
C_{70}	612	648	438	0.83
IC_{60}MA	620	645	457	0.79
C_{60}PCBM	698	722	543	0.72
C_{70}PCBM	787	822	598	0.71
Bis-C_{60}PCBM	887	905	715	0.64

6.2 Encapsulation of Enantiopure Cryptophane-A

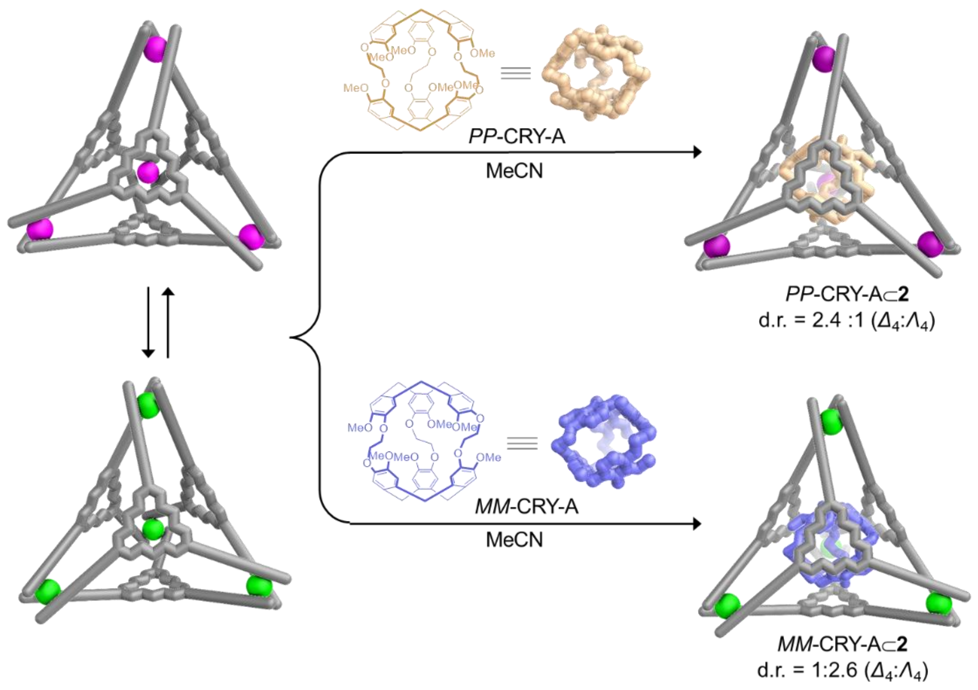

General procedure for the encapsulation of cryptophane-A (GP2): Cage **2** (8.8 mg, 1.0 µmol, 1.0 equiv) and enantiopure cryptophane-A[12] (CRY-A, 0.90 mg, 1.0 µmol, 1.0 equiv) were combined in MeCN (2 mL) in a 15 mL tube. The reaction mixture was stirred at 70 °C for 30 mins. After cooling down to room temperature, the reaction mixture was filtered through a short pipette filled with glass microfibre filter. The filtrate was evaporated to around 0.5 mL, and Et_2O (10 mL) was then added. The precipitate was collected by centrifugation and washed with excess Et_2O, affording the host-guest complex CRY-A⊂**2**.

The diastereomeric ratio of each host-guest complex was determined by ^{1}H NMR. Low-resolution ESI-MS confirmed that the 1:1 host-guest complex was formed. The characterization data for *PP*-CRY-A⊂**2** and *MM*-CRY-A⊂**2** are listed below:

PP-CRY-A$\subset$**2** (8.2 mg, 87%) was prepared according to method **GP2**. The encapsulation of *PP*-CRY-A retained the stereochemical configuration of the parent cage **2**. The d.r. was determined to be 2.4:1 (Δ_4:Λ_4) by ^{1}H NMR.

^{1}H NMR (500 MHz, CD_3CN): δ 1.02 (s, 76.2H, *PP*-CRY-A$\subset\Delta_4$-**2**), 1.05 (s, 31.8H, *PP*-CRY-A$\subset\Lambda_4$-**2**), 1.22 (d, *J* = 6.6 Hz, 10.6H, *PP*-CRY-A$\subset\Lambda_4$-**2**), 1.28 (d, *J* = 6.6 Hz, 25.4H, *PP*-CRY-A$\subset\Lambda_4$-**2**), 1.64–1.89 (m, 6H, *PP*-CRY-A), 2.64 (s, 12.7H, *PP*-CRY-A), 2.76 (s, 5.3H, *PP*-CRY-A), 3.03–3.25 (m, 6H, *PP*-CRY-A), 3.33–3.63 (m, 12H, *PP*-CRY-A), 4.09–4.22 (m, 12H,), 4.88–5.03 (m, 6H, *PP*-CRY-A), 5.19–5.30 (m, 6H, *PP*-CRY-A), 7.17 (d, *J* = 9.5 Hz, 12H), 7.61–7.74 (m, 12H), 8.11–8.21(m, 12H), 8.24–8.36 (m, 12H), 8.94–9.23 (m, 36H).

ESI-MS: m/z = 930.67 [*PP*-CRY-A$\subset$**2**-8(NTf_2)]$^{8+}$, 1103.68 [*PP*-CRY-A$\subset$**2**-7(NTf_2)]$^{7+}$, 1334.28 [*PP*-CRY-A$\subset$**2**-6(NTf_2)]$^{6+}$, 1657.19 [*PP*-CRY-A$\subset$**2**-5(NTf_2)]$^{5+}$.

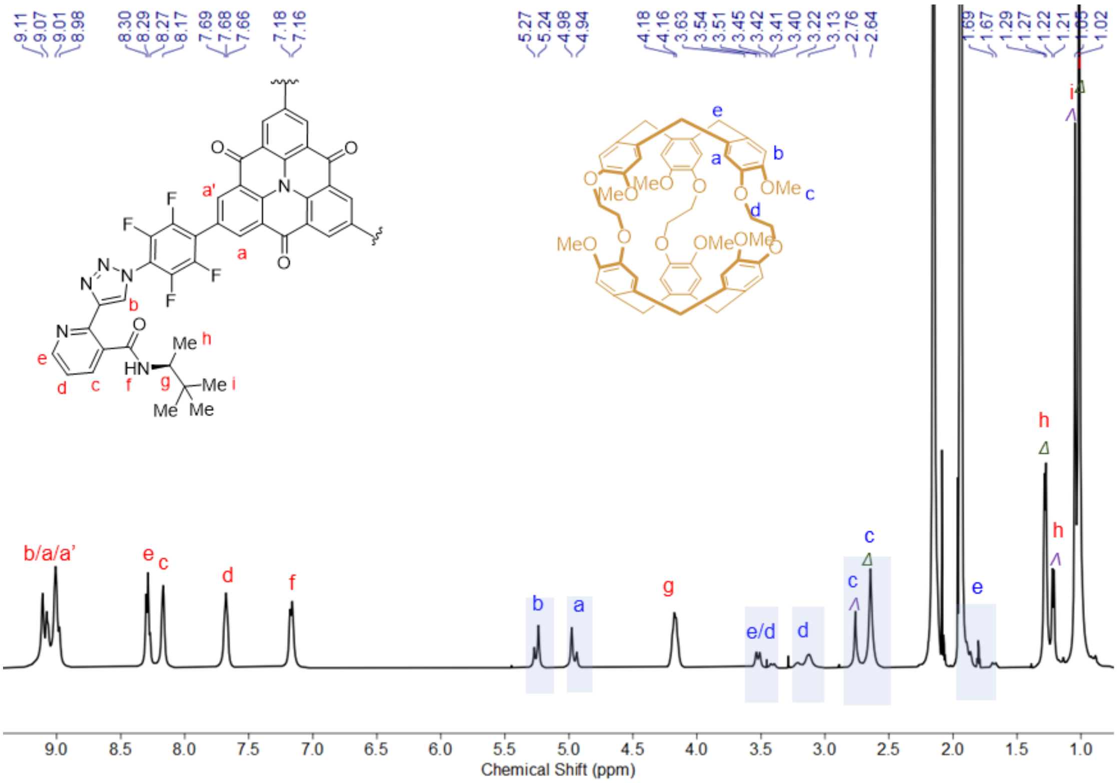

Figure S70. ^{1}H NMR spectrum of *PP*-CRY-A$\subset$**2** (500 MHz, CD_3CN, 25 °C).

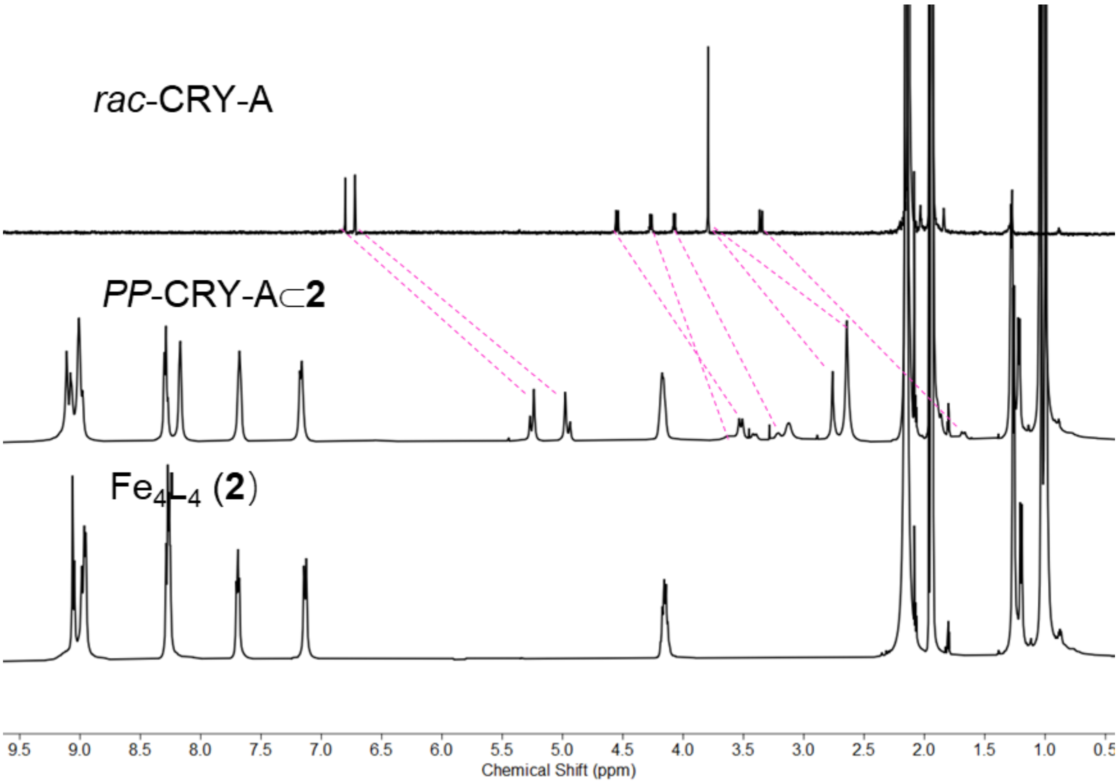

Figure S71. Comparison of the ^{1}H NMR spectra of **2**, *PP*-CRY-A and *PP*-CRY-A⊂**2**.

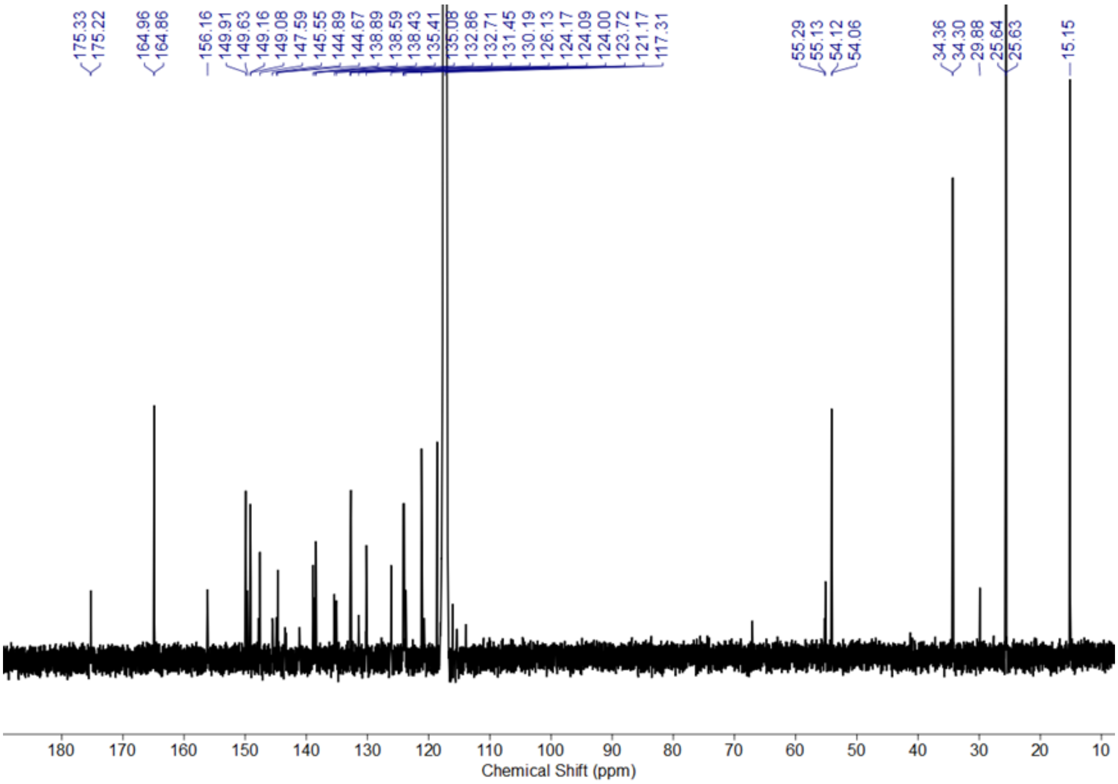

Figure S72. ^{13}C NMR spectrum of *PP*-CRY-A⊂**2** (126 MHz, CD_3CN, 25 °C).

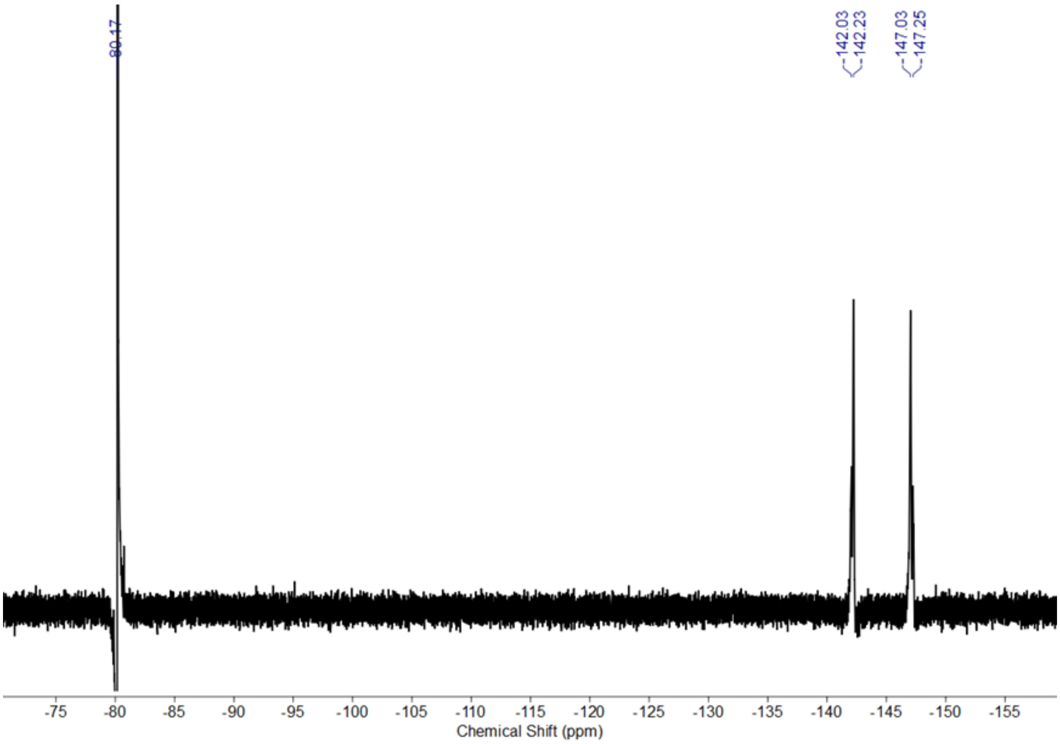

Figure S73. ^{19}F NMR spectrum of *PP*-CRY-A⊂**2** (376 MHz, CD_3CN, 25 °C).

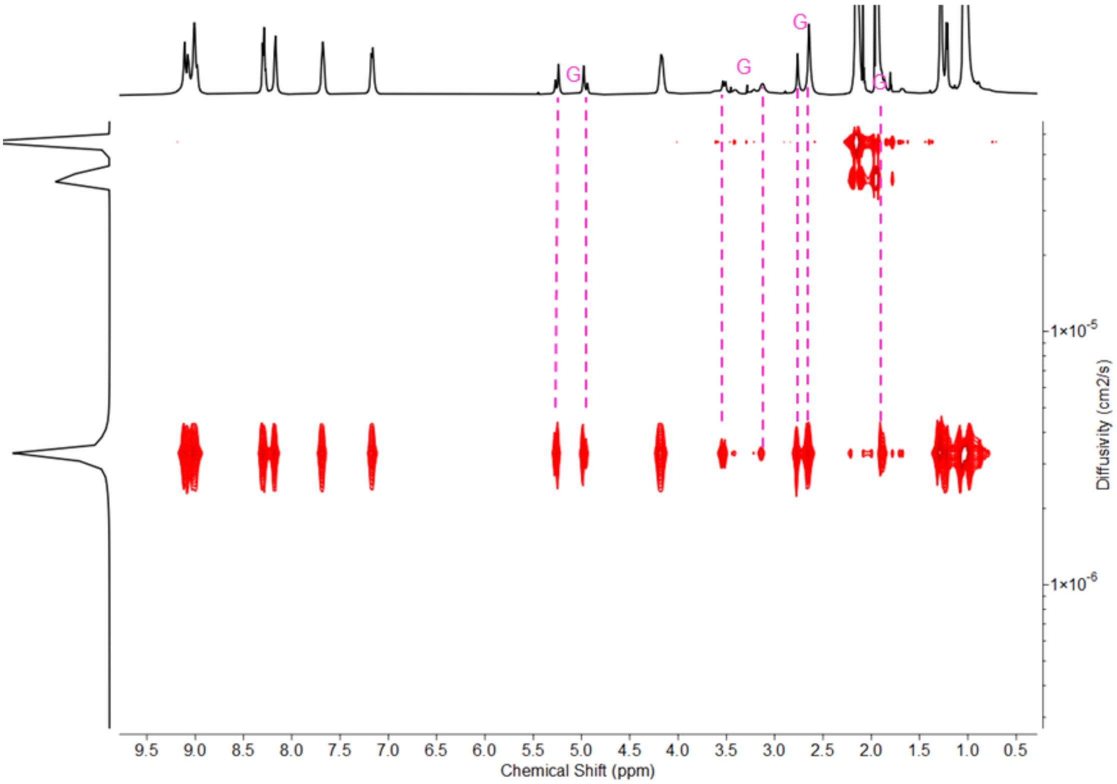

Figure 74. ^{1}H DOSY spectrum of *PP*-CRY-A⊂**2** (400 MHz, CD_3CN, 25 °C). The diffusion coefficient for both diastereomers in CD_3CN was measured to be 3.35×10^{-6} cm^2/s.

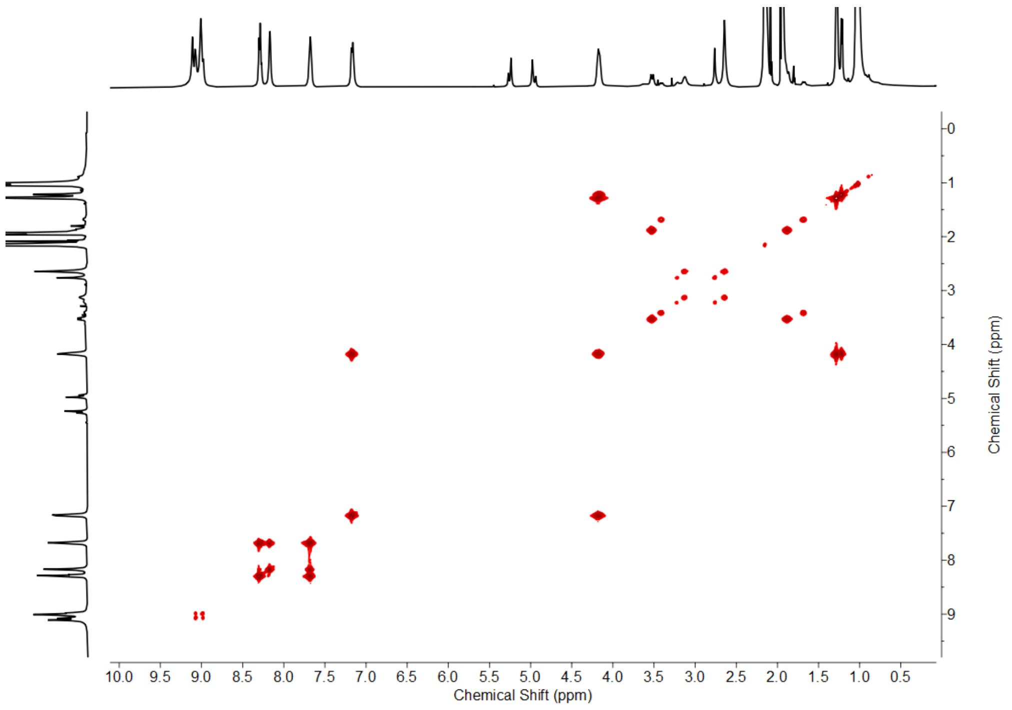

Figure S75. ^{1}H-^{1}H COSY NMR spectrum of *PP*-CRY-A⊂**2** (500 MHz, CD_3CN, 25 °C).

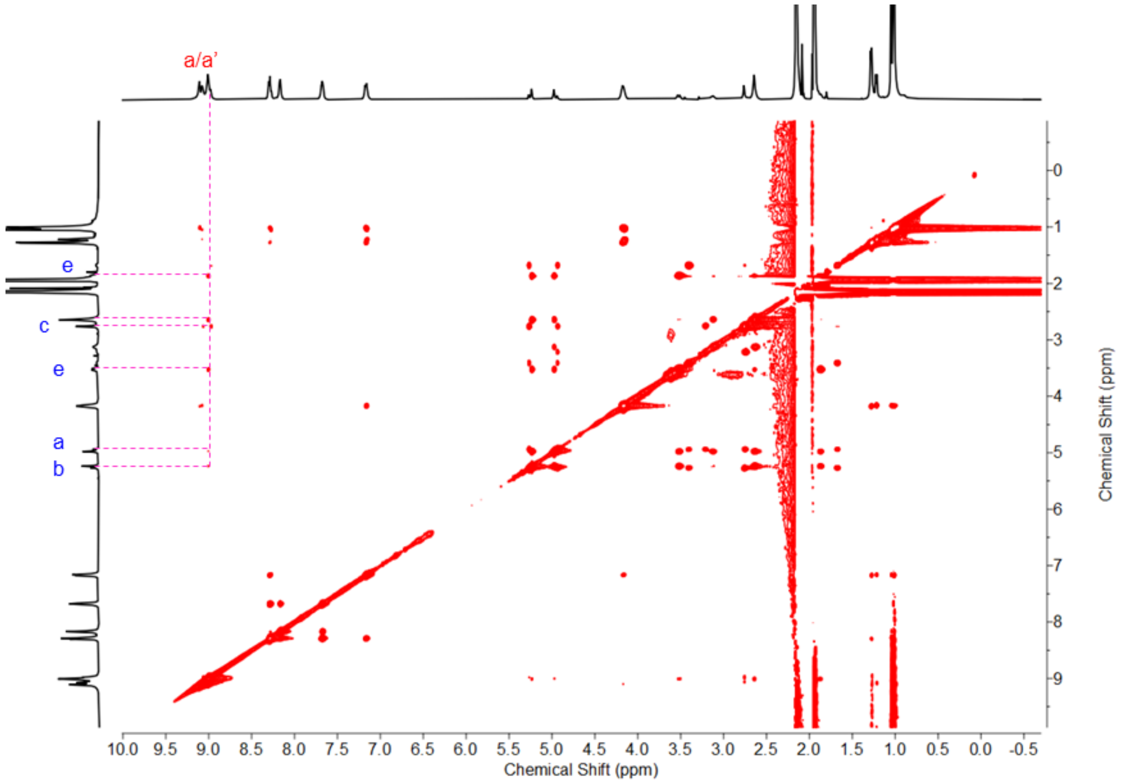

Figure S76. ^{1}H-^{1}H NOESY NMR spectrum of *PP*-CRY-A⊂**2** (500 MHz, CD_3CN, 25 °C). Host−guest interactions are highlighted with pink lines.

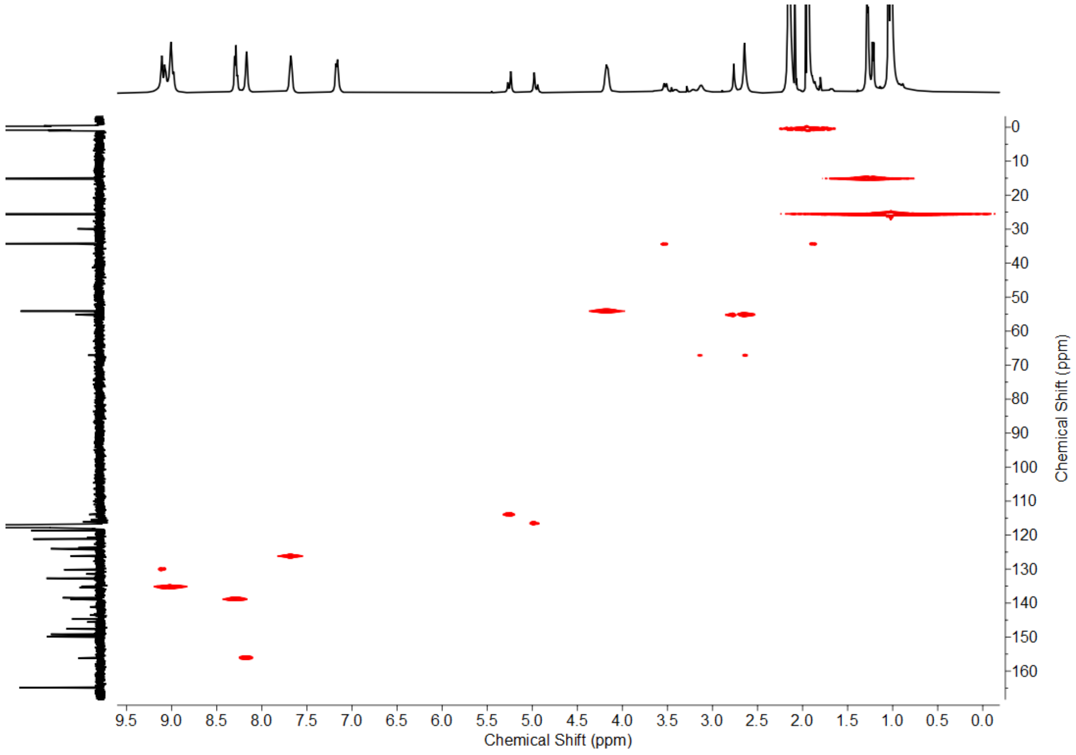

Figure S77. ^{1}H-^{13}C HSQC NMR spectrum of *PP*-CRY-A⊂**2** (500 MHz, CD_3CN, 25 °C).

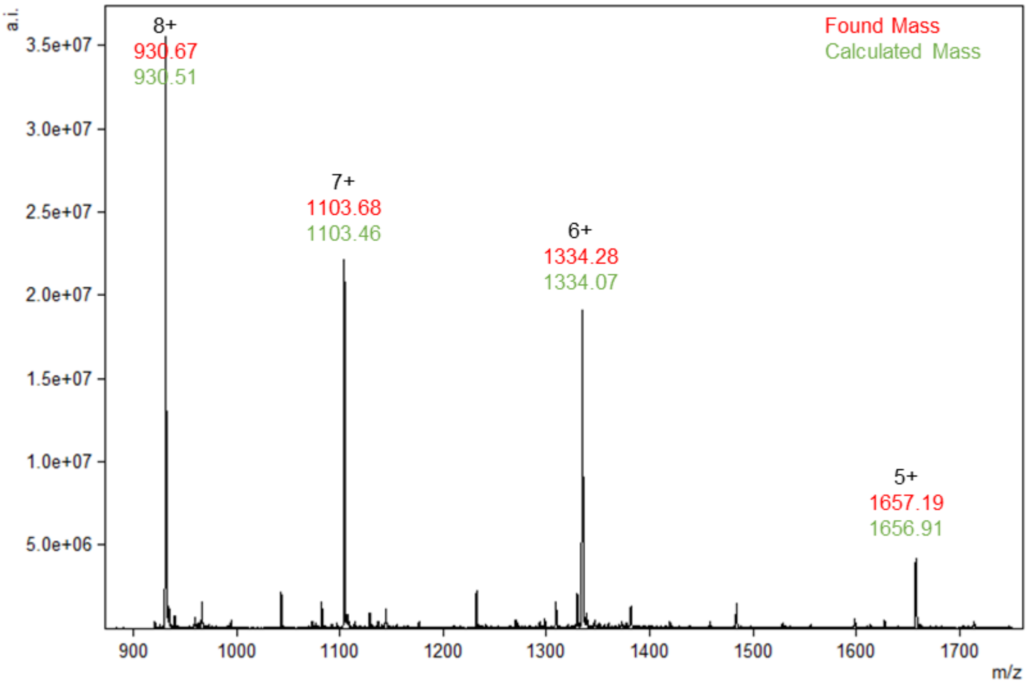

Figure S78. Low-resolution ESI-MS spectrum of *PP*-CRY-A⊂**2** in MeCN.

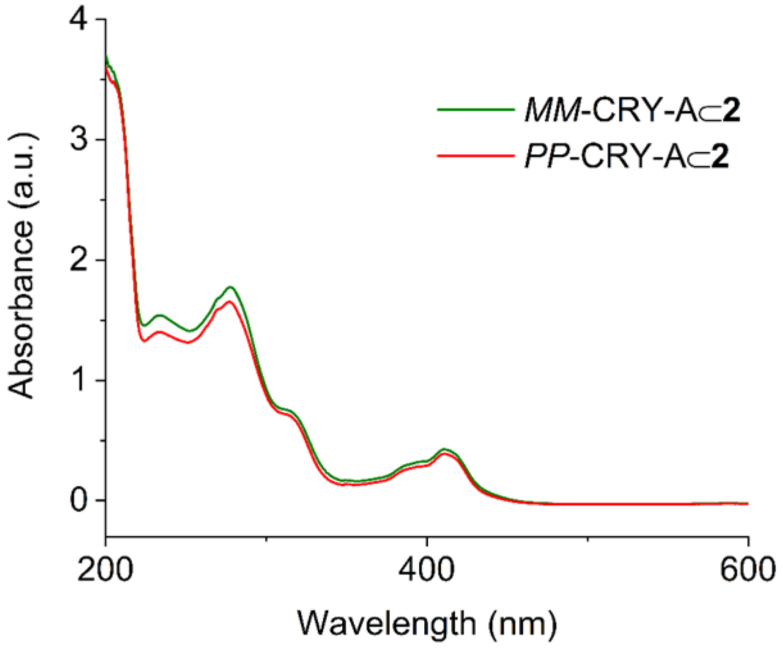

Figure S79. UV-vis spectra of *PP*-CRY-A⊂**2** and *MM*-CRY-A⊂**2** in MeCN.

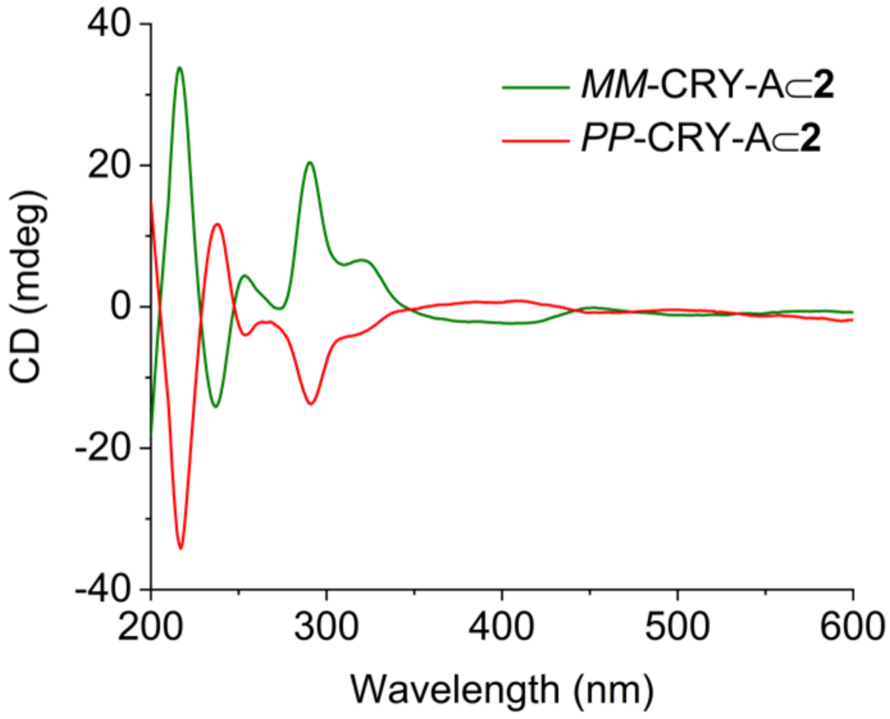

Figure S80. CD spectra of *PP*-CRY-A⊂**2** and *MM*-CRY-A⊂**2** in MeCN.f

MM-CRY-A⊂**2** (8.7 mg, 90%) was prepared according to method **GP2**. The encapsulation of *MM*-CRY-A occurred in a stereoinvertive manner. The d.r. was determined to be 1:2.5 (Δ_4:Λ_4) by ^{1}H NMR.

^{1}H NMR (500 MHz, CD_3CN): δ 1.01 (s, 31H, *MM*-CRY-A⊂Δ_4-**2**), 1.05 (s, 77H, *MM*-CRY-A⊂Λ_4-**2**), 1.22 (d, *J* = 6.6 Hz, 25.7H, *MM*-CRY-A⊂Λ_4-**2**), 1.28 (d, *J* = 6.6 Hz, 10.3H, *MM*-CRY-A⊂Λ_4-**2**), 1.62–1.89 (m, 6H, *MM*-CRY-A), 2.61 (s, 12.9H, *MM*-CRY-A), 2.76 (s, 5.1H, *MM*-CRY-A), 3.04–3.27 (m, 6H, *MM*-CRY-A), 3.35–3.70 (m, 12H, *MM*-CRY-A), 4.07–4.22 (m, 12H,), 4.89–5.00 (m, 6H, *MM*-CRY-A), 5.20–5.30 (m, 6H, *MM*-CRY-A), 7.16 (d, *J* = 9.5 Hz, 12H), 7.61–7.75 (m, 12H), 8.11–8.21(m, 12H), 8.28 (d, *J* = 7.2 Hz, 12H), 8.90–9.14 (m, 36H).

ESI-MS: m/z = 930.74 [*MM*-CRY-A⊂**2**-8(NTf_2)]$^{8+}$, 1103.75 [*MM*-CRY-A⊂**2**-7(NTf_2)]$^{7+}$, 1334.36 [*MM*-CRY-A⊂**2**-6(NTf_2)]$^{6+}$, 1657.32 [*MM*-CRY-A⊂**2**-5(NTf_2)]$^{5+}$.

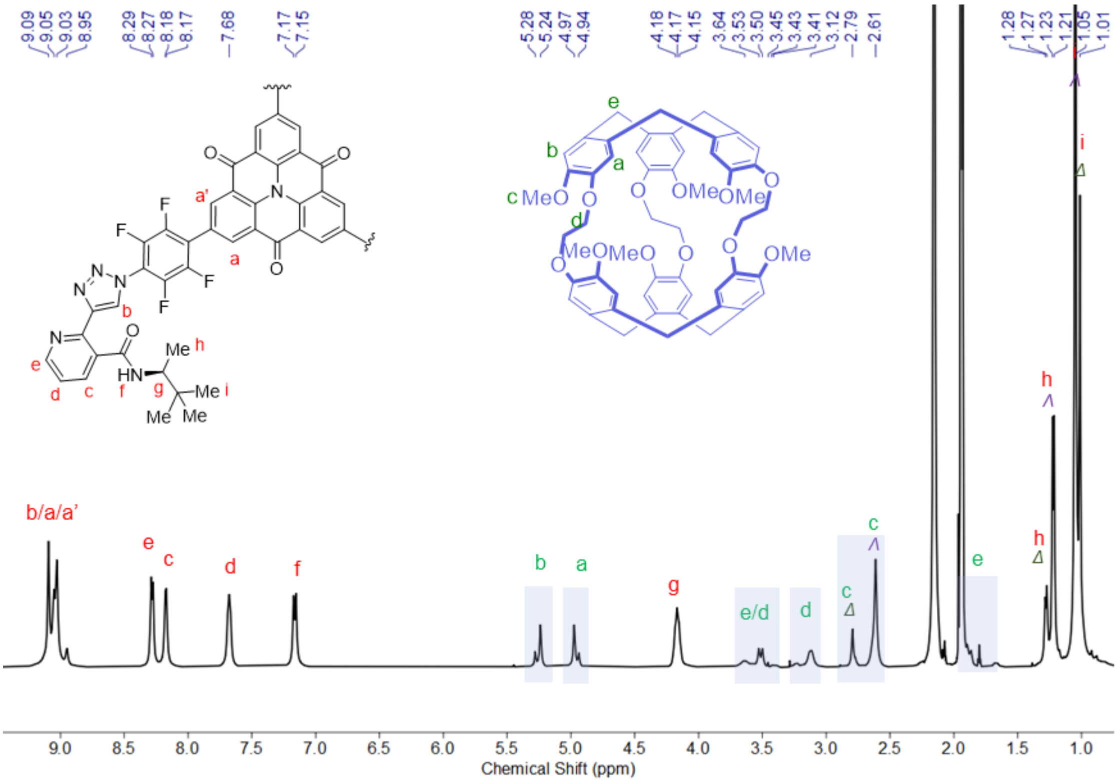

Figure S81. ^{1}H NMR spectrum of *MM*-CRY-A⊂**2** (500 MHz, CD_3CN, 25 °C).

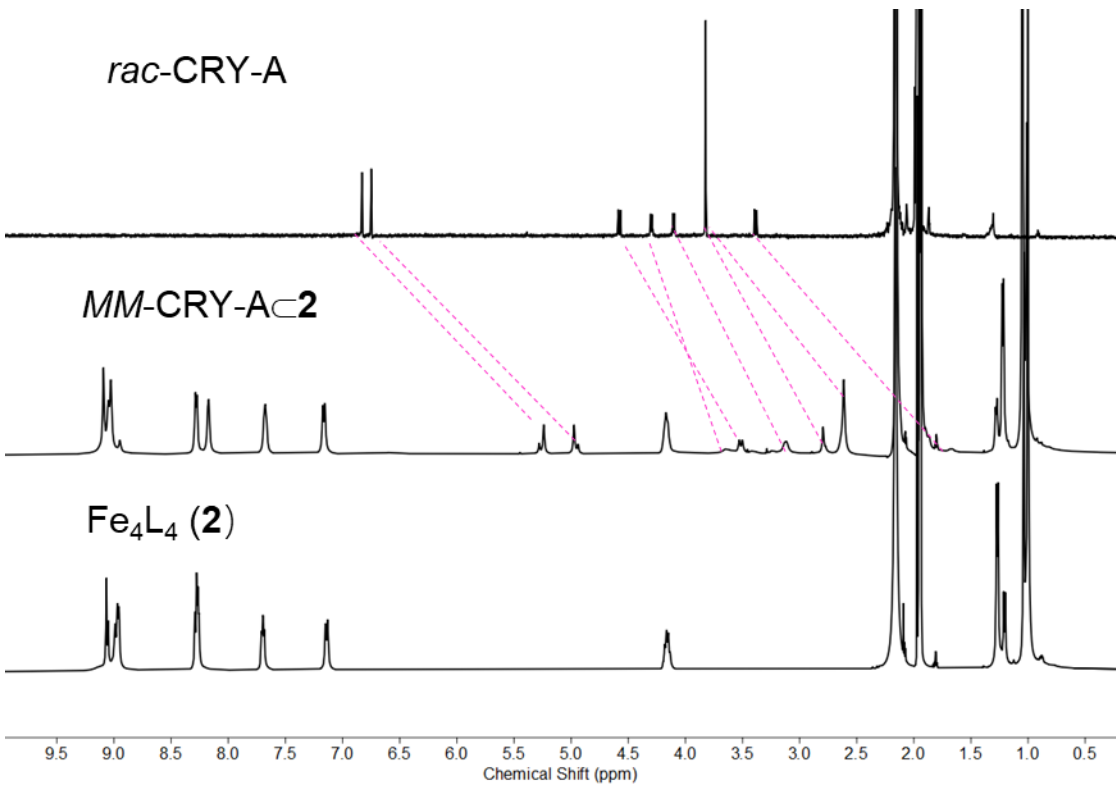

Figure S82. Comparison of the ^{1}H NMR spectra of **2**, *MM*-CRY-A and *MM*-CRY-A⊂**2**.

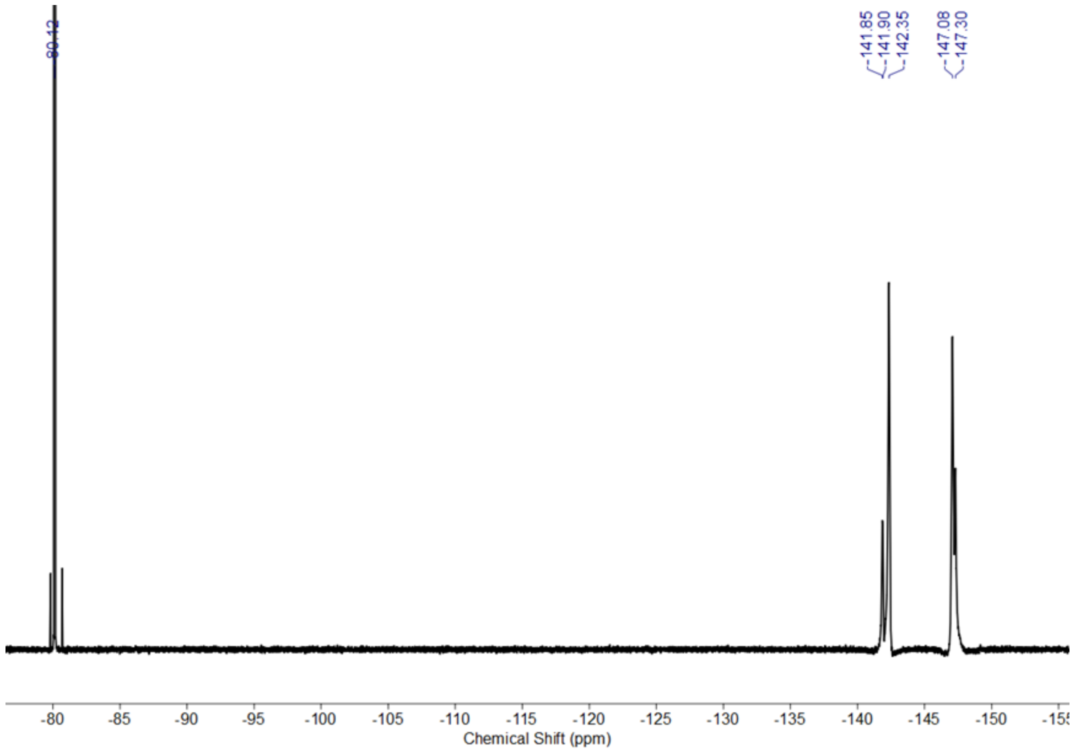

Figure S83. ^{19}F NMR spectrum of *MM*-CRY-A⊂**2** (376 MHz, CD_3CN, 25 °C).

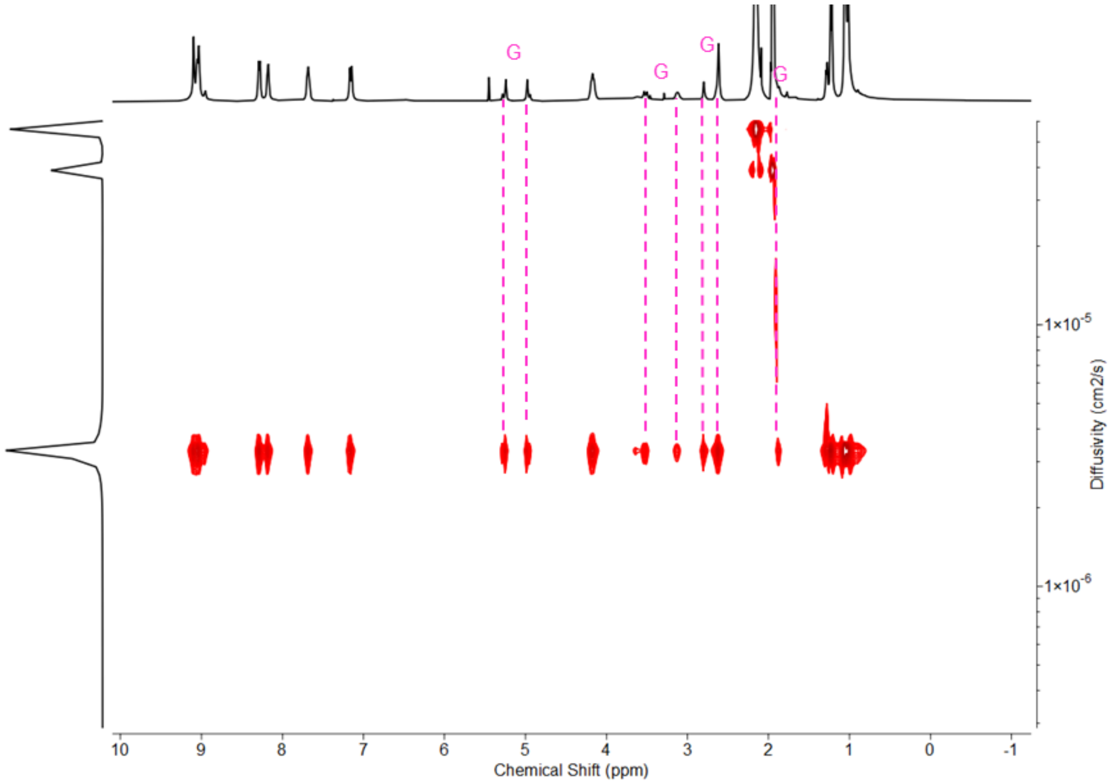

Figure 84. ^{1}H DOSY spectrum of *MM*-CRY-A⊂**2** (400 MHz, CD_3CN, 25 °C). The diffusion coefficient for both diastereomers in CD_3CN was measured to be 3.32×10^{-6} cm^2/s.

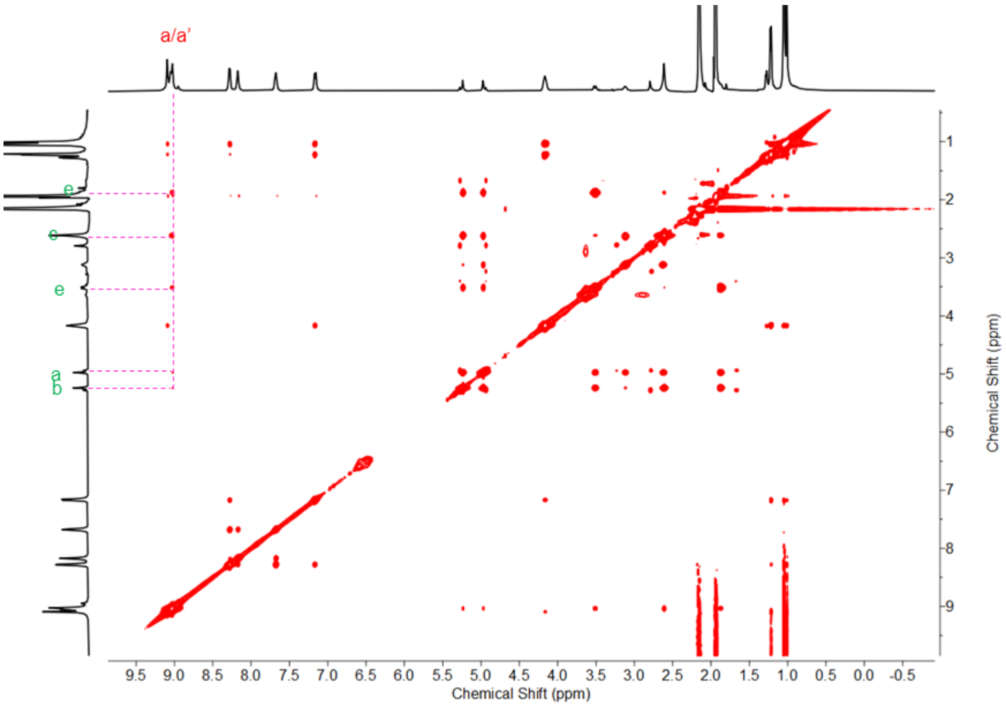

Figure S85. ^{1}H-^{1}H NOESY NMR spectrum of *MM*-CRY-A⊂**2** (500 MHz, CD_3CN, 25 °C). Host−guest interactions are highlighted with pink lines.

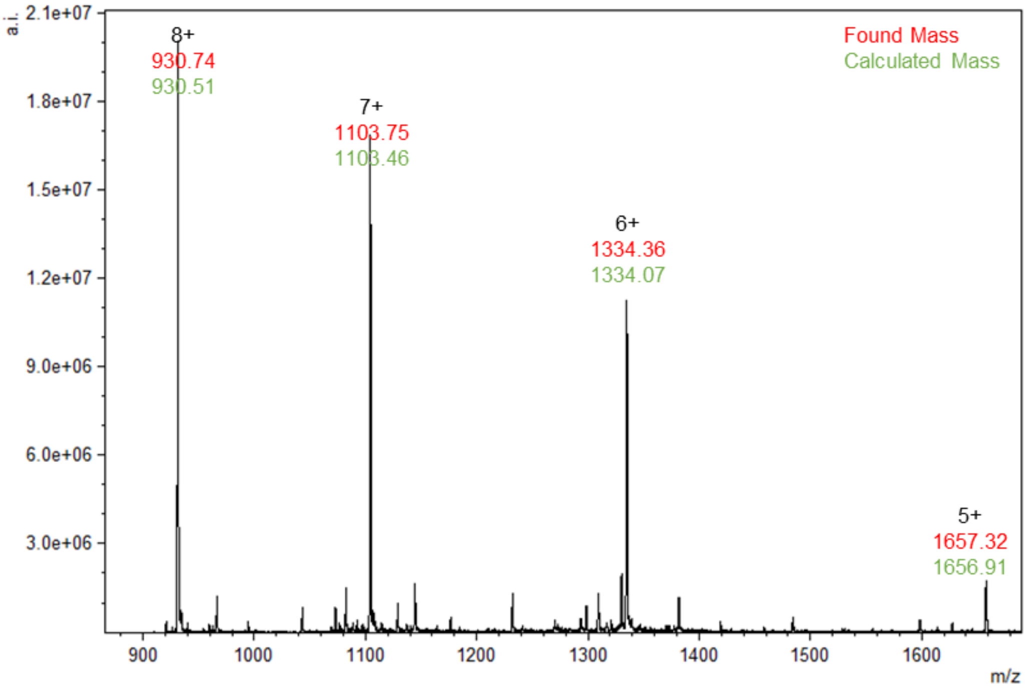

Figure S86. Low-resolution ESI-MS spectrum of *MM*-CRY-A⊂**2** in MeCN.

We also ran ^{1}H NMR titration experiments for the encapsulation of *MM*-CRY-A and *PP*-CRY-A. The guest was gradually added to an acetonitrile solution of **2** (4×10^{-4} M/L) and the reaction mixture was kept at 70 °C for 30 mins. After cooling down to room temperature, the ^{1}H NMR spectrum was measured. We observed complete encapsulation of CRY-A (from 0.25 equiv to 1.0 equiv) without free guest in solution. These results indicate that the binding constants are relatively large, precluding their calculation by ^{1}H NMR titration experiments.

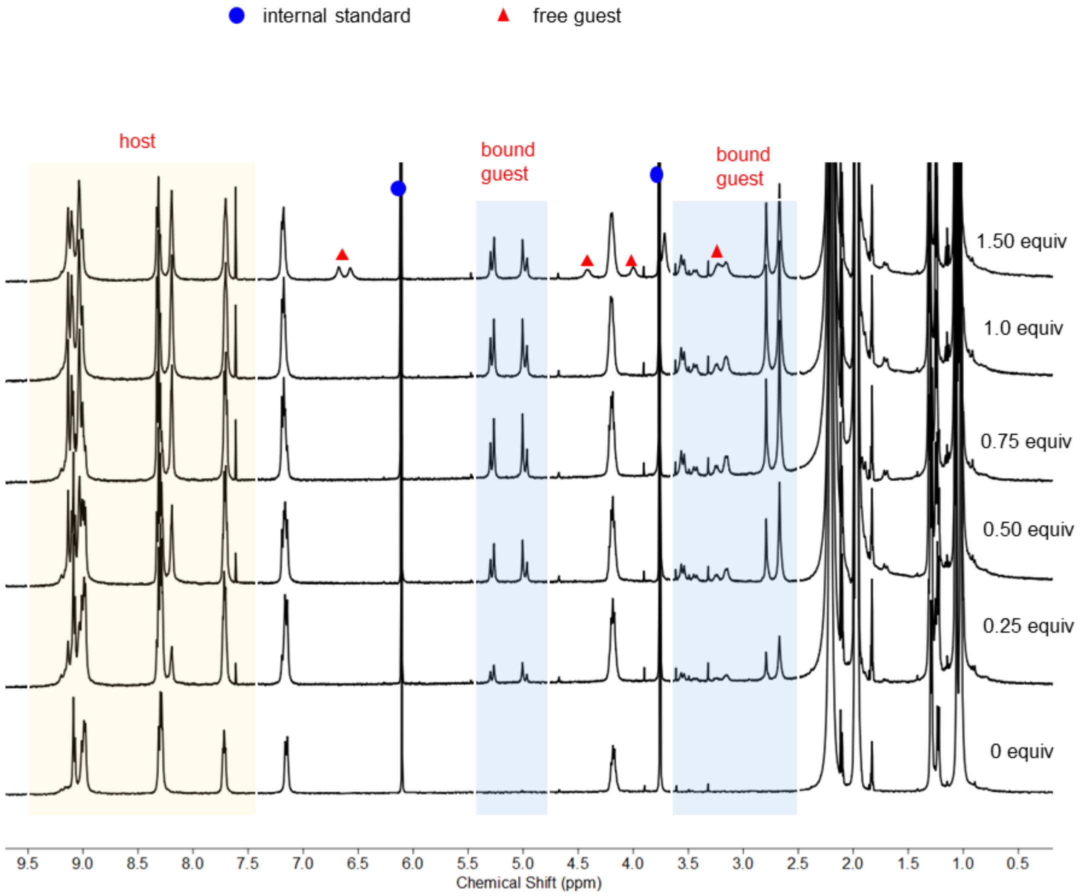

Figure 87. ^{1}H NMR titration experiments for the encapsulation of *PP*-CRY-A by cage **2** (400 MHz, CD_3CN, 25 °C). 1,3,5-Trimethoxybenzene was used as the internal standard.

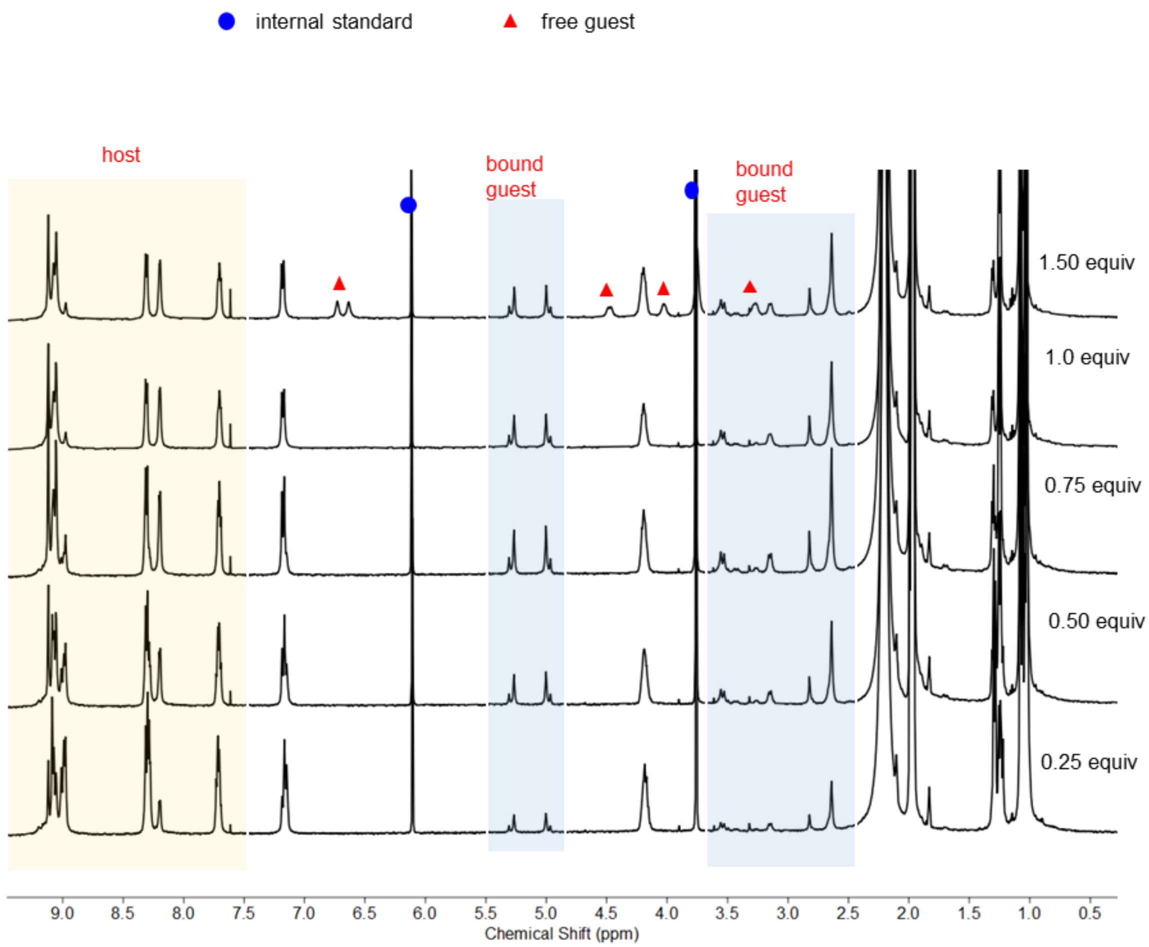

Figure 88. ^{1}H NMR titration experiments for the encapsulation of *MM*-CRY-A by cage **2** (400 MHz, CD_3CN, 25 °C). 1,3,5-Trimethoxybenzene was used as the internal standard.

7 Selective Encapsulation of Functionalized Fullerenes

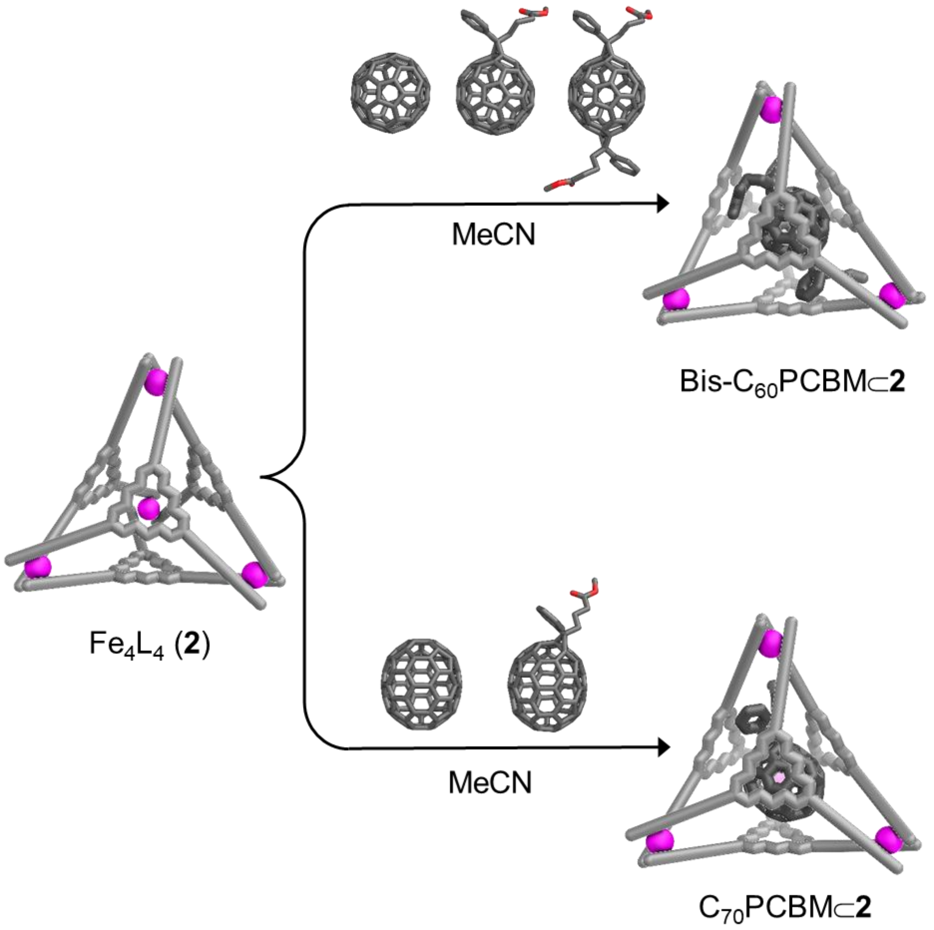

Cage **2** (8.8 mg, 1.0 μmol, 1.0 equiv), C_{60} (0.72 mg, 1.0 μmol, 1.0 equiv), C_{60}PCBM (0.91 mg, 1.0 μmol, 1.0 equiv), and bis-C_{60}PCBM (1.10 mg, 1.0 μmol, 1.0 equiv) were combined in MeCN (2 mL) in a 15 mL tube. The reaction mixture was stirred at 70 °C for 30 mins. After cooling down to room temperature, the reaction mixture was filtered twice through a short pipette filled with glass microfibre filter. The filtrate was evaporated to around 0.5 mL, and Et_2O (10 mL) was then added. The precipitate was collected by centrifugation and washed with excess Et_2O, affording the host-guest complex bis-C_{60}PCBM⊂**2**.

^{1}H NMR and HR-ESI-MS spectra were in line with the exclusive formation of bis-C_{60}PCBM⊂**2**. C_{60}⊂**2** and C_{60}PCBM⊂**2** were not observed.

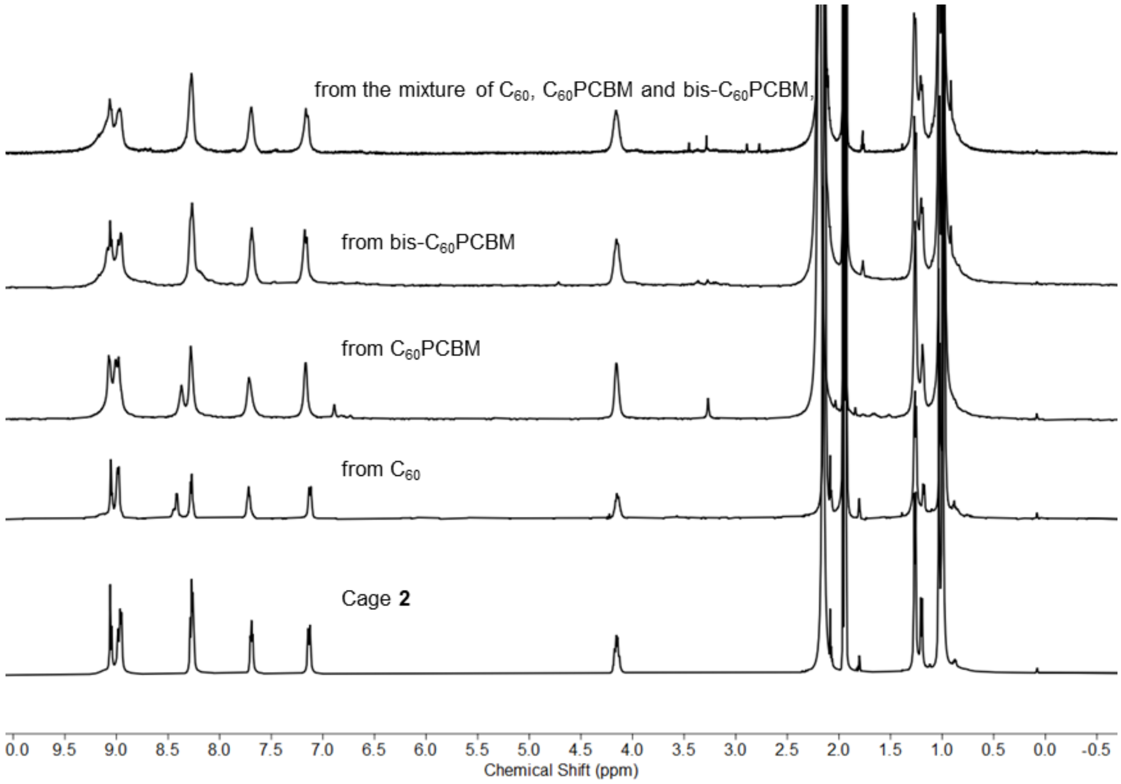

Figure S89. Comparison of ^{1}H NMR spectra for the selective encapsulation of bis-C_{60}PCBM from mixtures by cage **2** (400 MHz, CD_3CN, 25 °C).

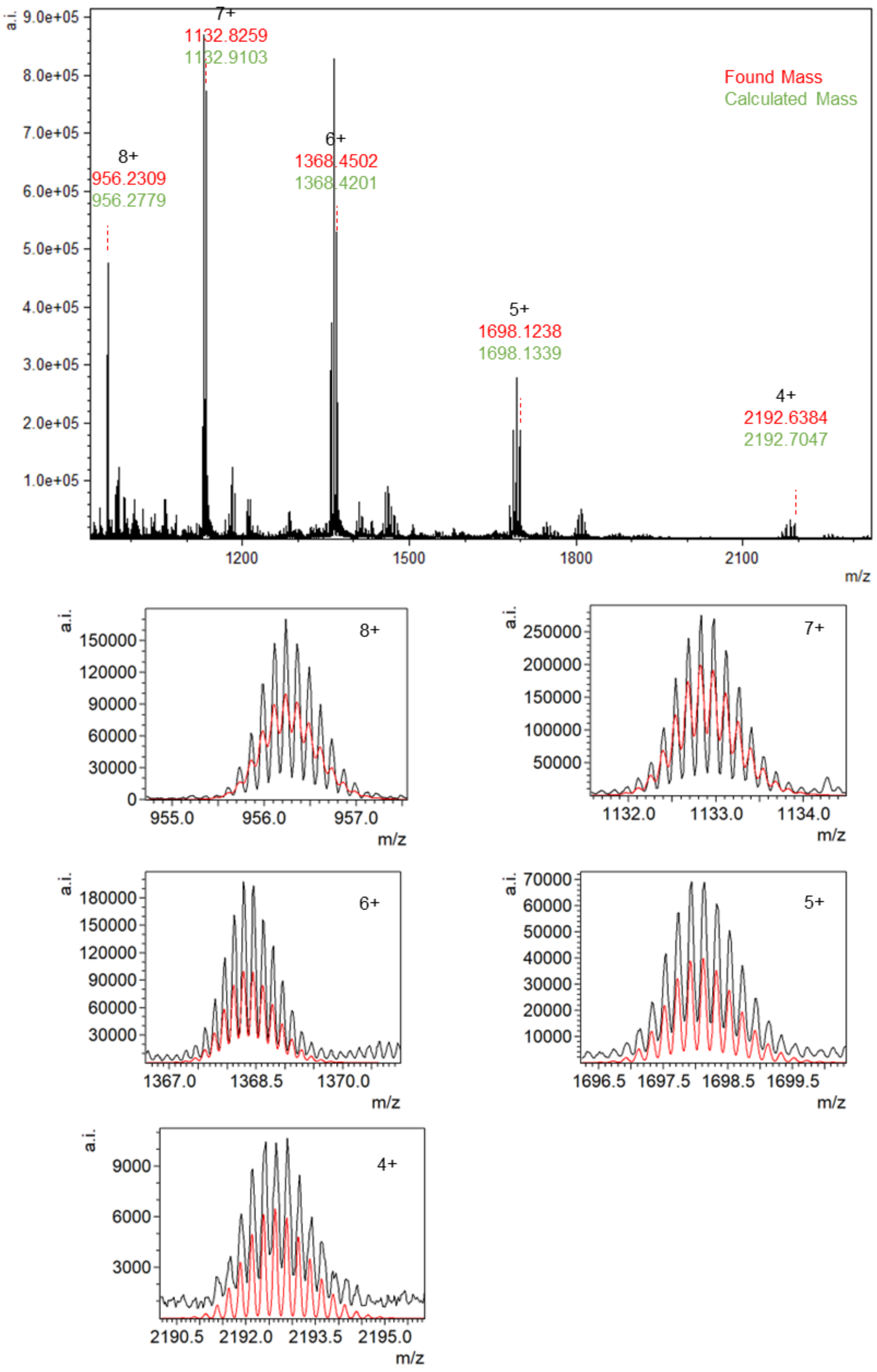

Figure S90. High-resolution ESI-MS spectrum for the selective encapsulation of bis-C_{60}PCBM in MeCN. Fragments correspond to the loss of a methyl or methoxy group within bis-C60PCBM under the test conditions.

Cage **2** (8.8 mg, 1.0 μmol, 1.0 equiv), C_{70} (0.84 mg, 1.0 μmol, 1.0 equiv) and C_{70}PCBM (1.03 mg, 1.0 μmol, 1.0 equiv) were combined in MeCN (2 mL) in a 15 mL tube. The reaction mixture was stirred at 70 °C for 30 mins. After cooling down to room temperature, the reaction mixture was filtered twice through a short pipette filled with glass microfibre filter. The filtrate was evaporated to around 0.5 mL, and Et_2O (10 mL) was then added. The precipitate was collected by centrifugation and washed with excess Et_2O, affording the host-guest complex C_{70}PCBM⊂**2**.

^{1}H NMR and HR-ESI-MS spectra were consistent with the exclusive formation of C_{70}PCBM⊂**2**. C_{70}⊂**2** was not observed.

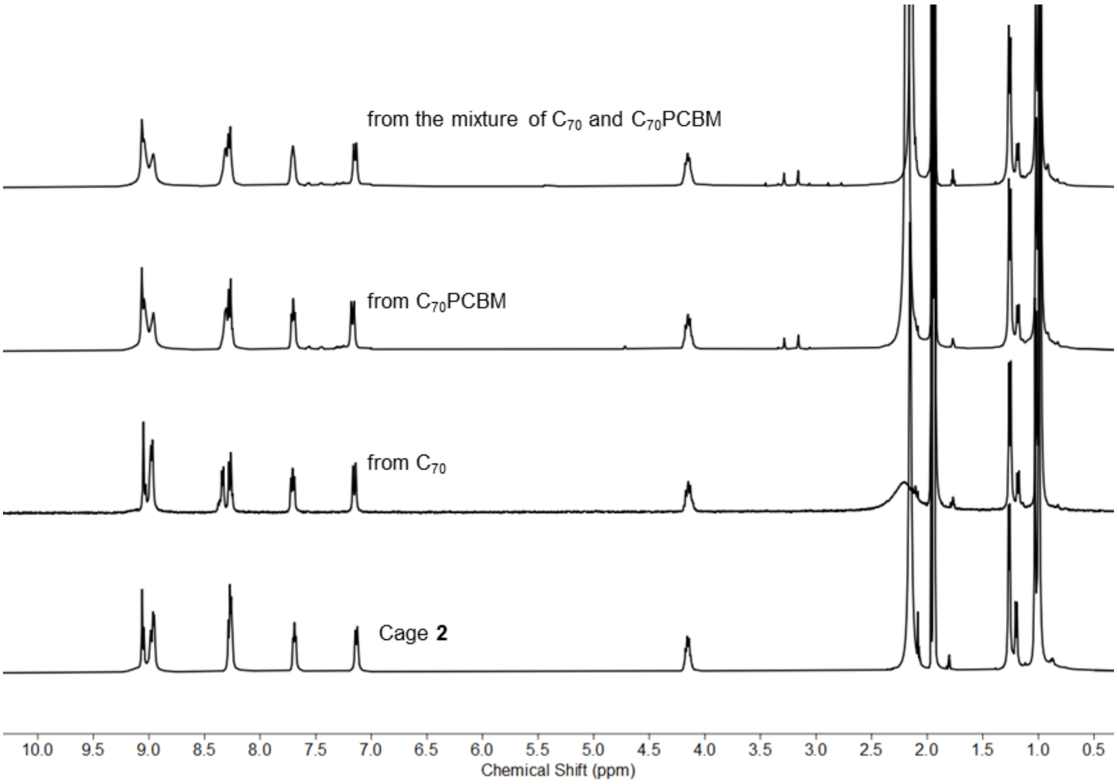

Figure S91. Comparison of ^{1}H NMR spectra for the selective encapsulation of C_{70}PCBM from mixtures and encapsulation of C_{70}PCBM (400 MHz, CD_3CN, 25 °C).

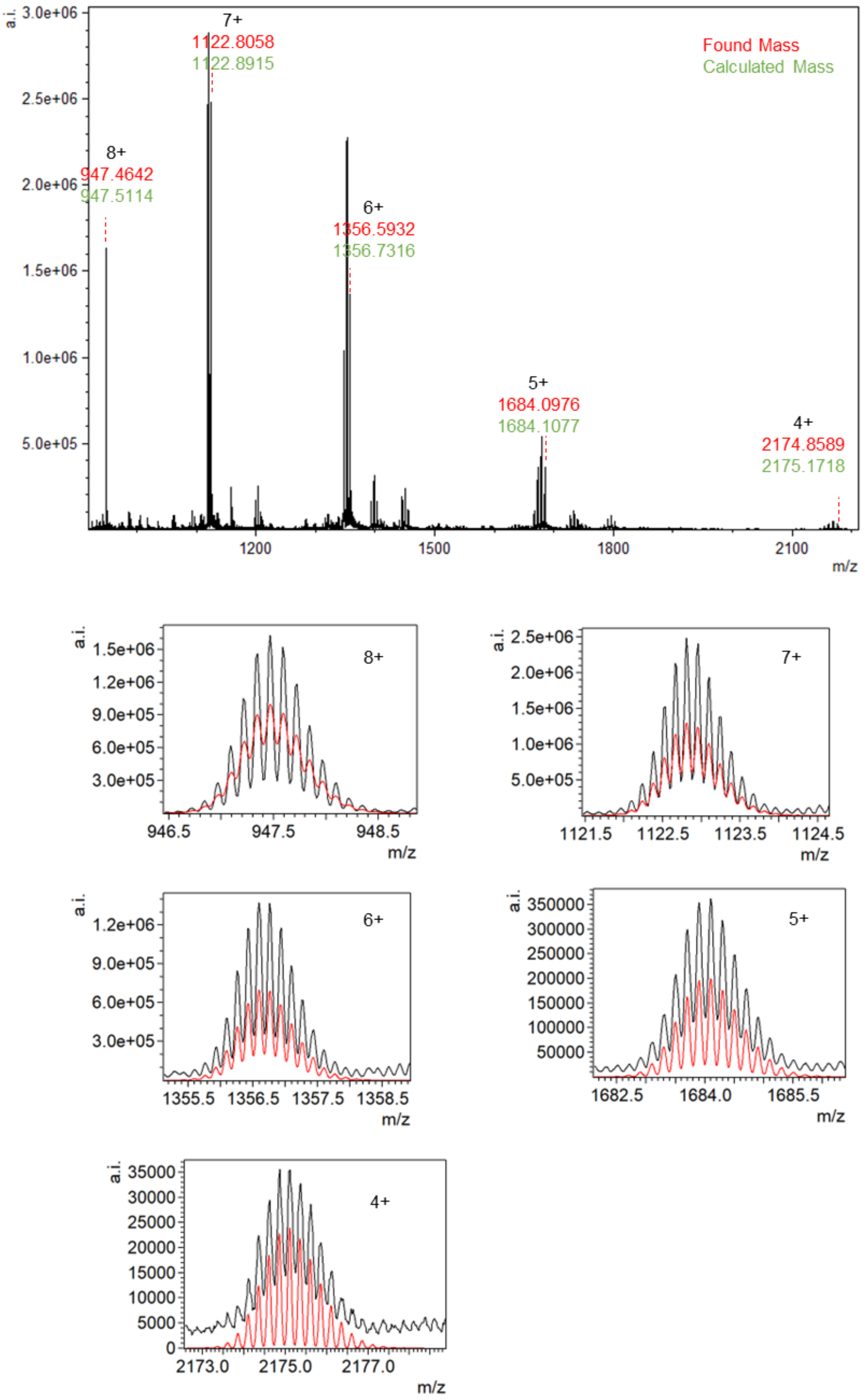

Figure S92. High-resolution ESI-MS spectrum for the selective encapsulation of C_{70}PCBM in MeCN. Fragments correspond to the loss of a methyl or methoxy group within C_{60}PCBM under the test conditions.

8 Enantioselective Separation of Racemic Cryptophane-A

First round of enantioselective separation: Cage **2** (35.1 mg, 4.0 μmol, 1.0 equiv) and racemic cryptophane-A (7.2 mg, 8.0 μmol, 2.0 equiv) were combined in MeCN (6 mL) in a 25 mL flask. The reaction mixture was stirred at 70 °C for 30 mins. After cooling down to room temperature, the solvent was evaporated to around 2 mL, and Et_2O (30 mL) was then added. The precipitate was collected by centrifugation and washed 3 times with excess Et_2O, affording the host-guest complex CRY-A⊂**2**. Evaporating the combined diethyl ether supernatant layers subsequently afforded unbound CRY-A, which was purified by preparative TLC.

Comparison of the ^{1}H NMR spectra with those of *PP*-CRY-A⊂**2** and *MM*-CRY-A⊂**2** allowed us to identify each diastereomer in solution. The NMR spectrum clearly showed that more *MM*-CRY-A was encapsulated, when two equivalents of racemic guest were used. Cotton effects assigned to *MM*-CRY-A were also observed in the CD spectrum of CRY-A⊂**2**. The enantiomeric excess (ee) of the unbound CRY-A was determined to be 32% by chiral HPLC.

HPLC analysis of unbound CRY-A: Chiralpak ID, ethanol/dichloromethane (50/50), 1 mL/min, UV (254 nm), CD (254 nm), retention times R_t in minutes.

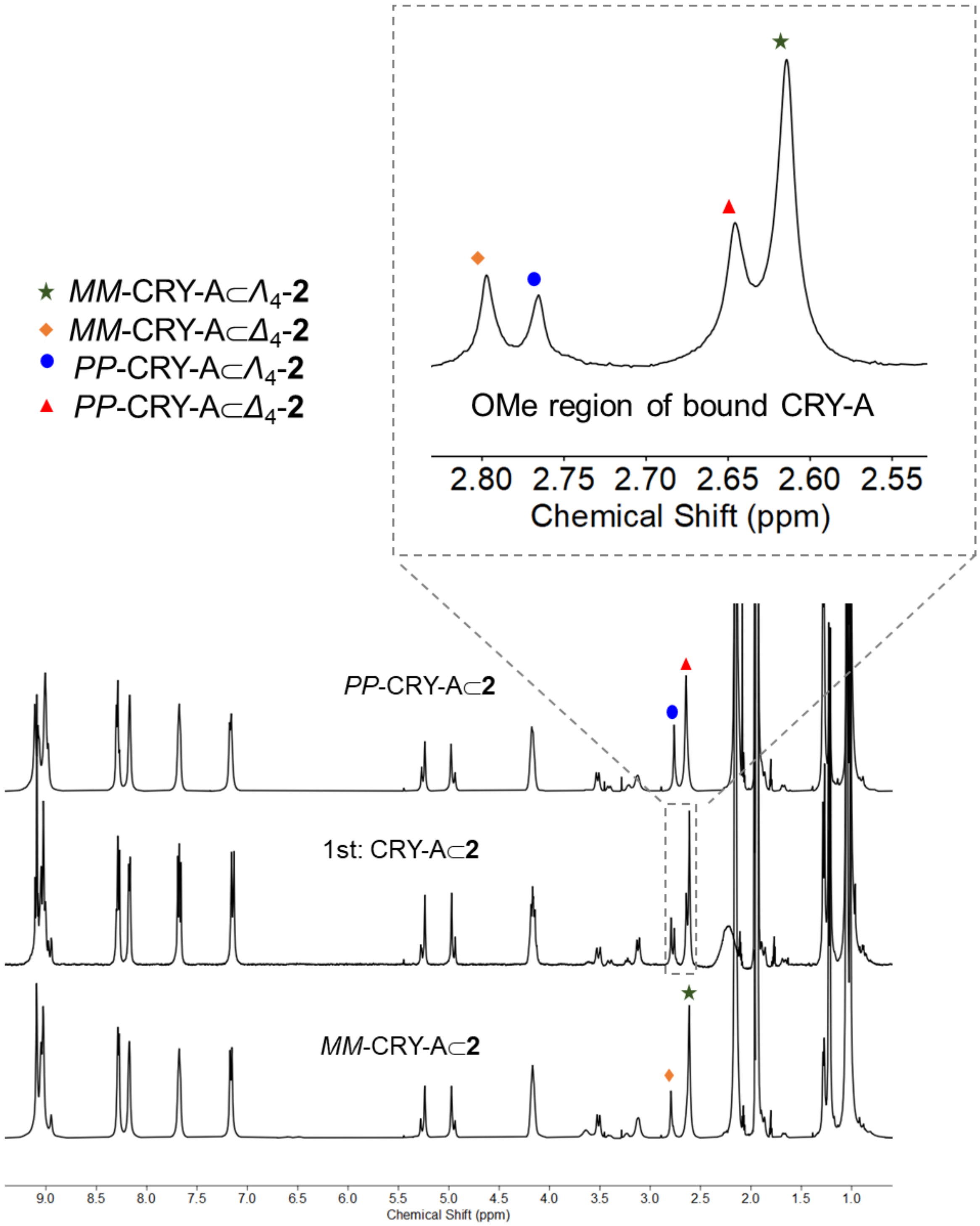

Figure S93. Comparison of the ^{1}H NMR spectra of *PP*-CRY-A⊂**2**, *MM*-CRY-A⊂**2** and CRY-A⊂**2** obtained in the first round of the resolution experiment.

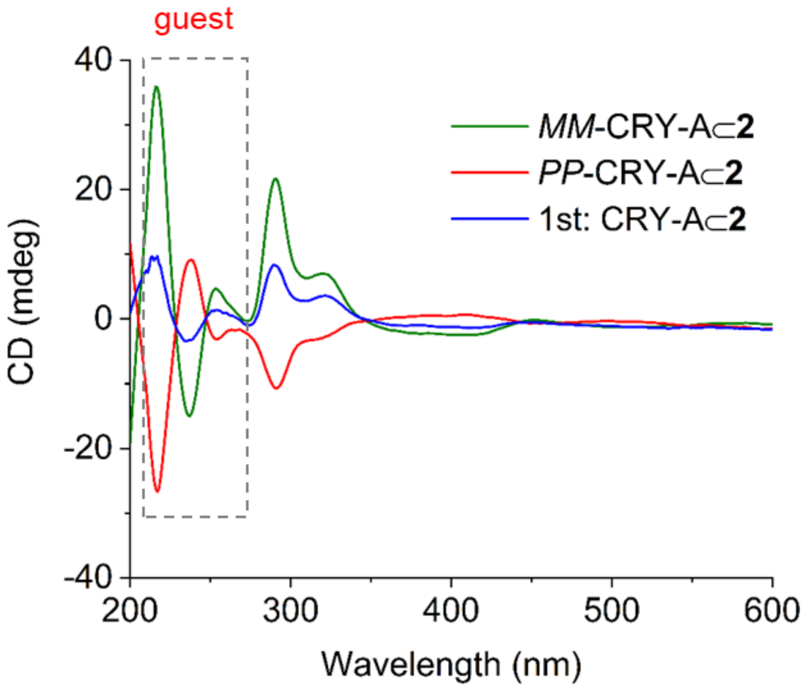

Figure S94. Comparison of the CD spectra of *PP*-CRY-A⊂**2**, *MM*-CRY-A⊂**2** and CRY-A⊂**2** obtained after the first round of the resolution experiment in MeCN.

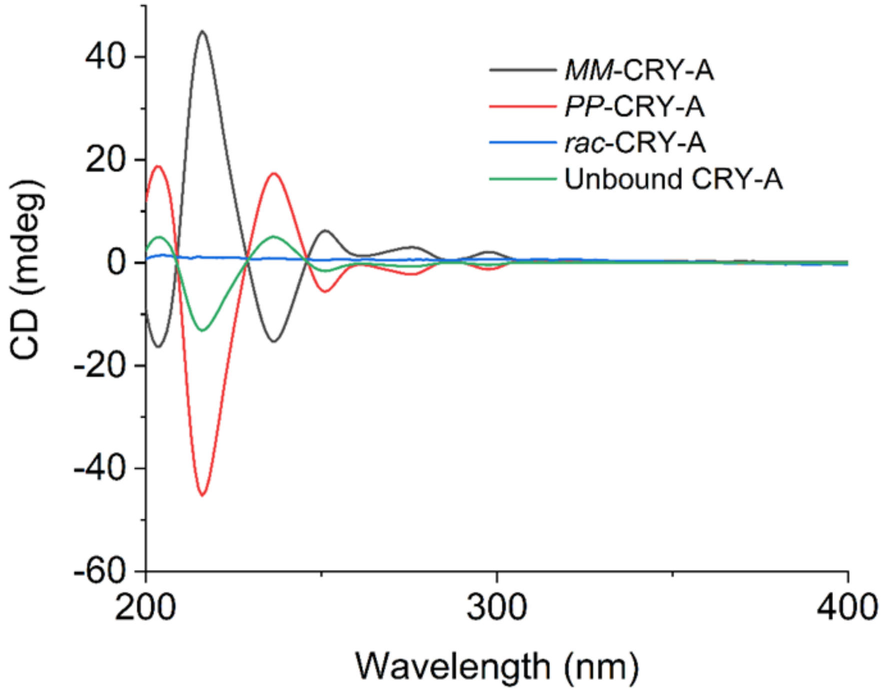

Figure S95. Comparison of the CD spectra of *PP*-CRY-A, *MM*-CRY-A, *rac*-CRY-A and unbound CRY-A in MeCN.

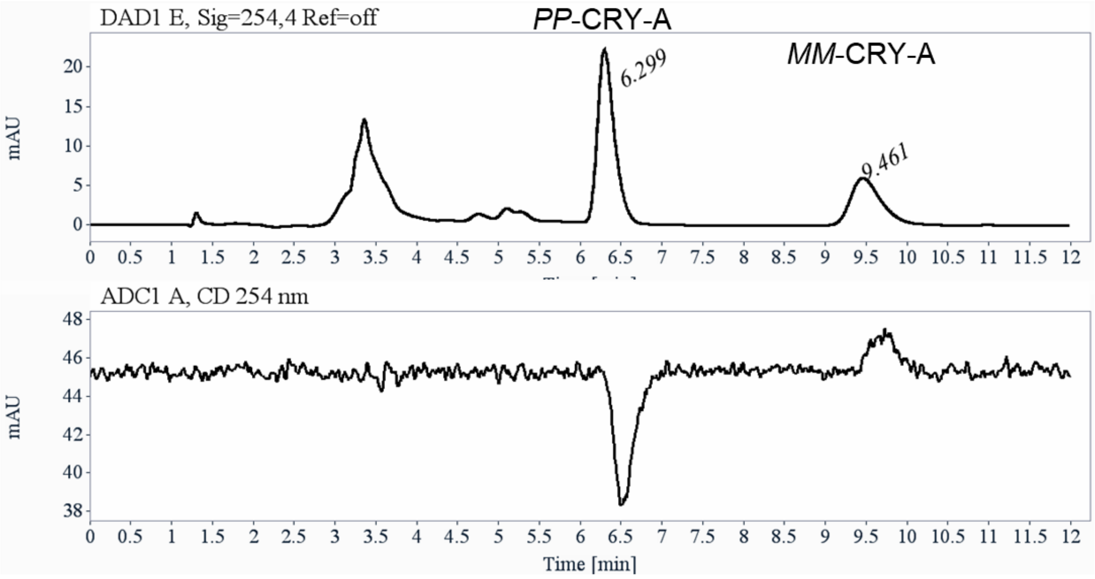

RT [min]	Area	Area%	Capacity Factor	Enantioselectivity	Resolution (USP)
6.30	319	65.98	1.14		
9.46	164	34.02	2.21	1.94	5.91
Sum	483	100.00			

Figure S96. HPLC analysis of the unbound CRY-A obtained in the first round of the resolution experiment.

Second round of enantioselective separation: Cage **2** (26.3 mg, 3 μmol, 1.0 equiv) and cryptophane-A (32% ee, 4.1 mg, 4.5 μmol, 1.5 equiv) were combined in CD_3CN (4 mL) in a 15 mL tube. The reaction mixture was stirred at 70 °C for 30 mins. After cooling down to room temperature, the solvent was evaporated to around 2 mL, and Et_2O (30 mL) was then added. The precipitate was collected by centrifugation and washed 3 times with excess Et_2O, affording the host-guest complex CRY-A⊂**2**. Evaporating the combined diethyl ether supernatant layers subsequently afforded unbound CRY-A, which was purified by preparative TLC. The ee of the unbound CRY-A was determined to be 77% by chiral HPLC.

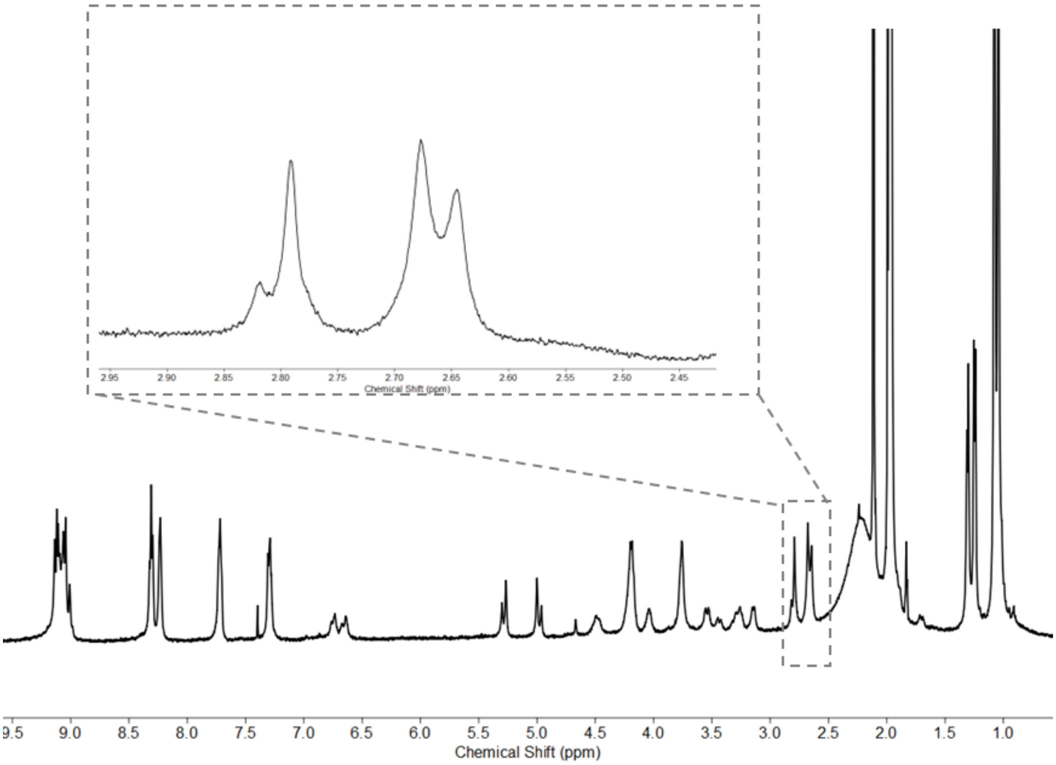

Figure S97. Crude ^{1}H NMR spectrum after encapsulation in the second round of the resolution experiment (400 MHz, CD_3CN, 25 °C).

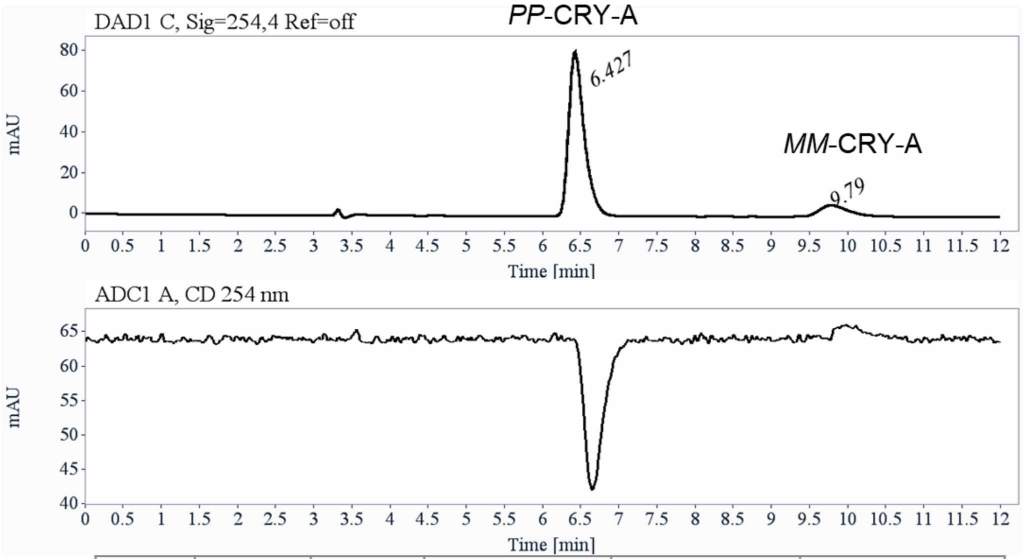

RT [min]	Area	Area%	Capacity Factor	Enantioselectivity	Resolution (USP)
6.43	1153	88.16	1.18		
9.79	155	11.84	2.32	1.97	6.33
Sum	1308	100.00			

Figure S98. HPLC analysis of unbound CRY-A obtained in the second round of the resolution experiment.

Third round of enantioselective separation: Cage **2** (17.5 mg, 2 μmol, 1.0 equiv) and cryptophane-A (77% ee, 2.7 mg, 3 μmol, 1.5 equiv) were combined in CD_3CN (2 mL) in a 15 mL tube. The reaction mixture was stirred at 70 °C for 30 mins. After cooling down to room temperature, the solvent was evaporated to around 1 mL, and Et_2O (30 mL) was then added. The precipitate was collected by centrifugation and washed 3 times with excess Et_2O, affording the host-guest complex CRY-A⊂**2**. Evaporating the combined diethyl ether supernatant layers subsequently afforded unbound CRY-A, which was purified by preparative TLC. The ee of unbound CRY-A was determined to be 78% by chiral HPLC.

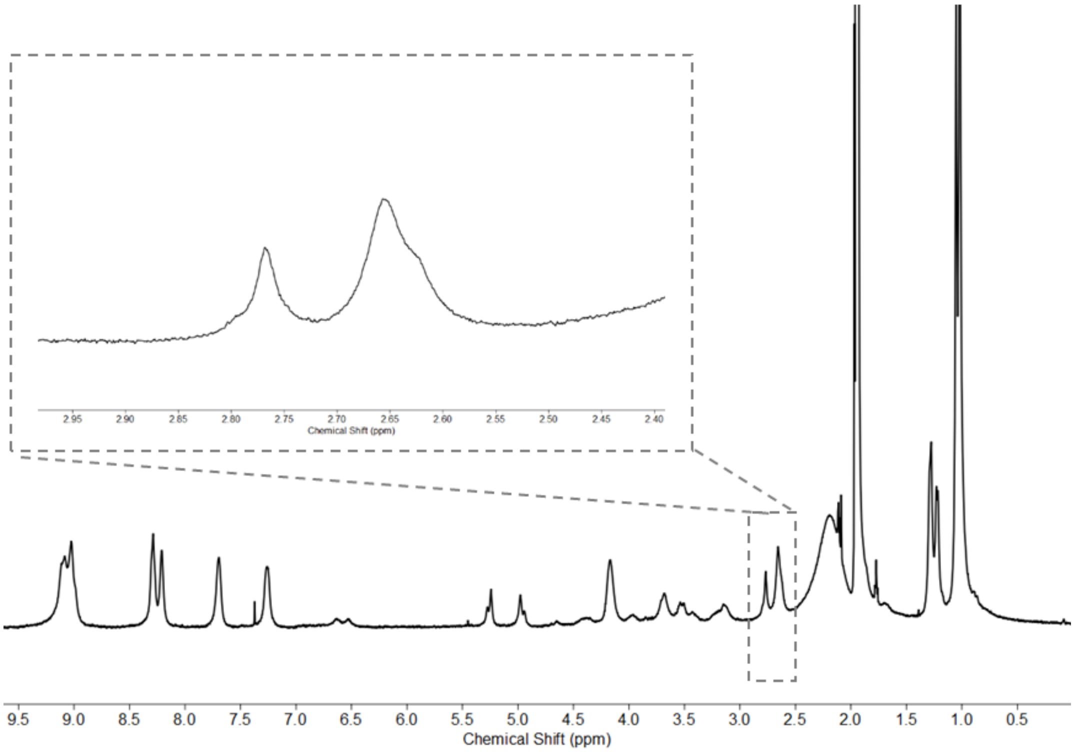

Figure S99. Crude ^1H NMR spectrum after encapsulation in the third round of the resolution experiment (400 MHz, CD_3CN, 25 °C).

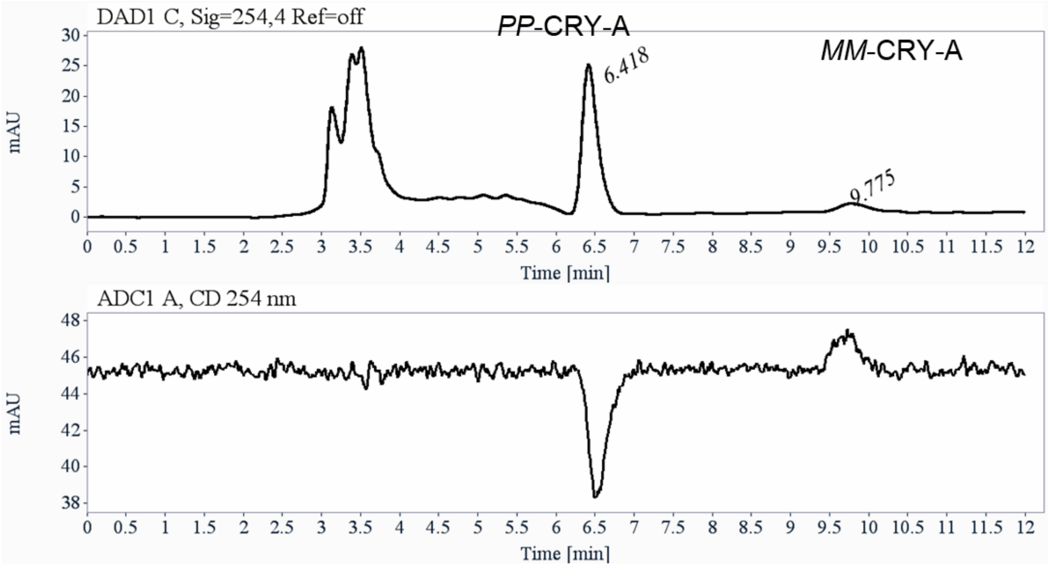

RT [min]	Area	Area%	Capacity Factor	Enantioselectivity	Resolution (USP)
6.42	345	89.03	1.18		
9.77	42	10.97	2.31	1.97	6.26
Sum	387	100.00			

Figure S100. HPLC analysis of unbound CRY-A obtained after the third round of the resolution experiment.

Control experiments: Cage **2** (3.52 mg, 0.4 μmol, 1.0 equiv) and racemic cryptophane-A (0.36 mg, 0.4 μmol, 1.0 equiv) were combined in CD_3CN (0.5 mL) in a NMR tube. The NMR tube was sonicated for 5 mins to dissolve the cage, and the ^1H NMR spectrum was measured. The NMR tube was then kept at 70˚C in an oil bath. The reaction mixture was monitored by ^1H NMR until no further changes were observed in the OMe region of host-guest complex. Afterwards, another 1 equiv cryptophane-A was added to the NMR tube. The NMR tube was then kept at 70˚C in an oil bath, and the reaction mixture was monitored by ^1H NMR.

Four sets of signals from the methoxy groups of CRY-A were observed in the 2.55–2.83 ppm region, indicating that CRY-A⊂**2** is comprised of four diastereomers, *PP*-CRY-A⊂Δ_4-**2**, *PP*-CRY-A⊂Λ_4-**2**, *MM*-CRY-A⊂Δ_4-**2**, and *MM*-CRY-A⊂Λ_4-**2**. We observed that *PP*-CRY-A was encapsulated kinetically faster than *MM*-CRY-A upon addition of 1 equiv *rac*-CRY-A to cage **2** at room temperature. After the mixture was kept at 70 ˚C for 5 mins, *rac*-CRY-A was completely encapsulated with

MM-CRY-A⊂*Λ*$_4$-**2** as the major diastereomer, indicating that *MM*-CRY-A⊂*Λ*$_4$-**2** is the thermodynamically favored diastereomer of the four diastereomers for CRY-A⊂**2**.

Another 1 equiv *rac*-CRY-A was then added to the 1:1 host-guest complex. Upon heating the mixture at 70 °C, we observed that the amount of *MM*-CRY-A⊂*Λ*$_4$-**2** increased, with a decrease in *PP*-CRY-A⊂*Δ*$_4$-**2** and *PP*-CRY-A⊂*Λ*$_4$-**2**. These results reflect that the chiral resolution process is driven by the formation of the thermodynamically favored diastereomer *MM*-CRY-A⊂*Λ*$_4$-**2**.

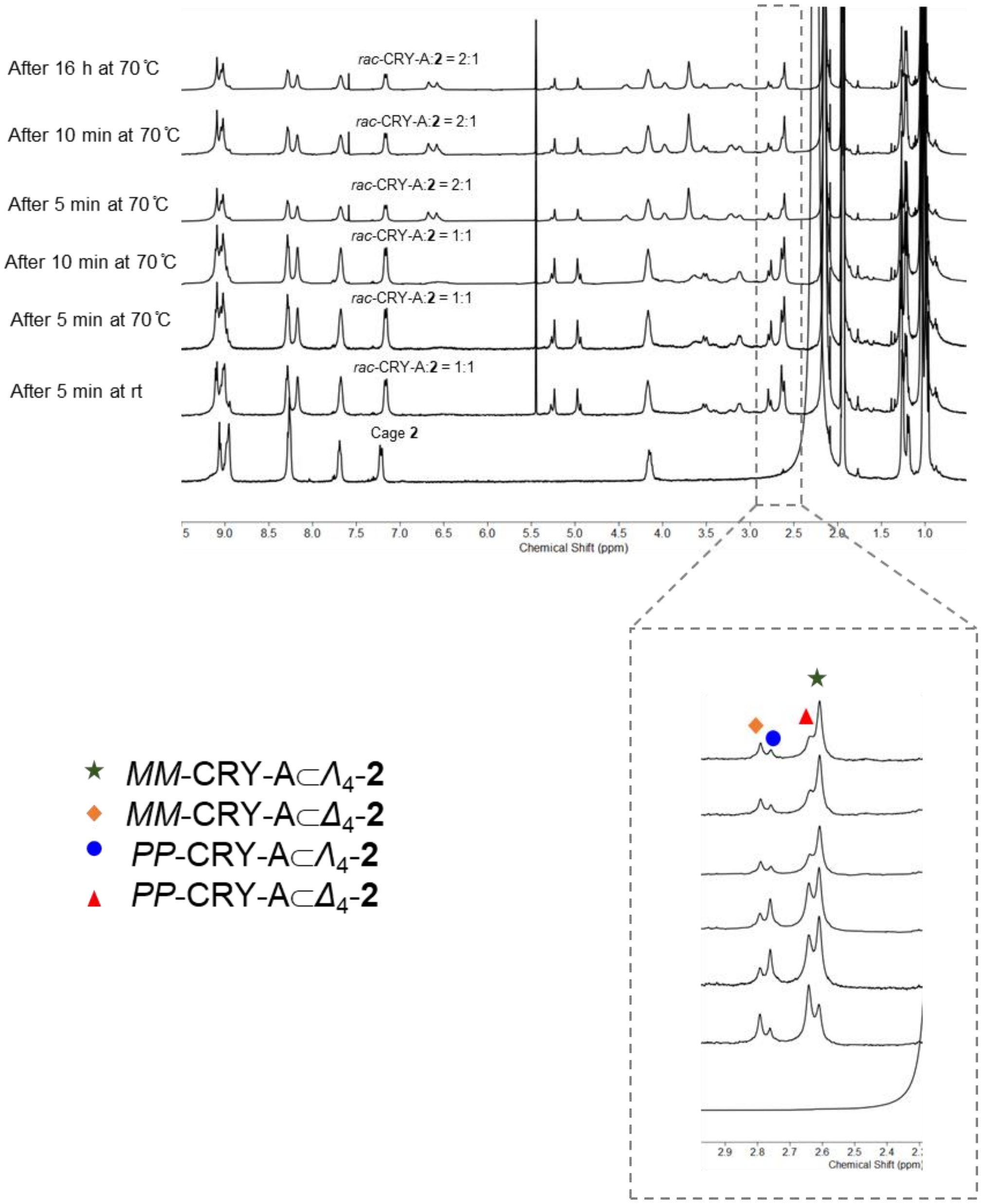

Figure S101. Comparison of the ^{1}H NMR spectra of control experiments (400 MHz, CD_3CN, 25 °C).

Guest release and cage recycling: Host-guest complex (20 mg) obtained in the first round of the resolution experiment was suspended in $CHCl_3$ (5 mL) in a 15 mL tube. The tube was sonicated at room temperature for 45 mins to extract the guest from cage. The precipitate was collected by centrifugation and suspended again in $CHCl_3$ (5 mL). The reaction mixture was sonicated at room temperature for another 45 mins to extract the guest from cage. The precipitate was collected by centrifugation, affording cage **2** (17 mg). Evaporating the combined $CHCl_3$ supernatant layers subsequently afforded bound CRY-A. Recycled cage **2** can be used for host-guest studies without further purification.

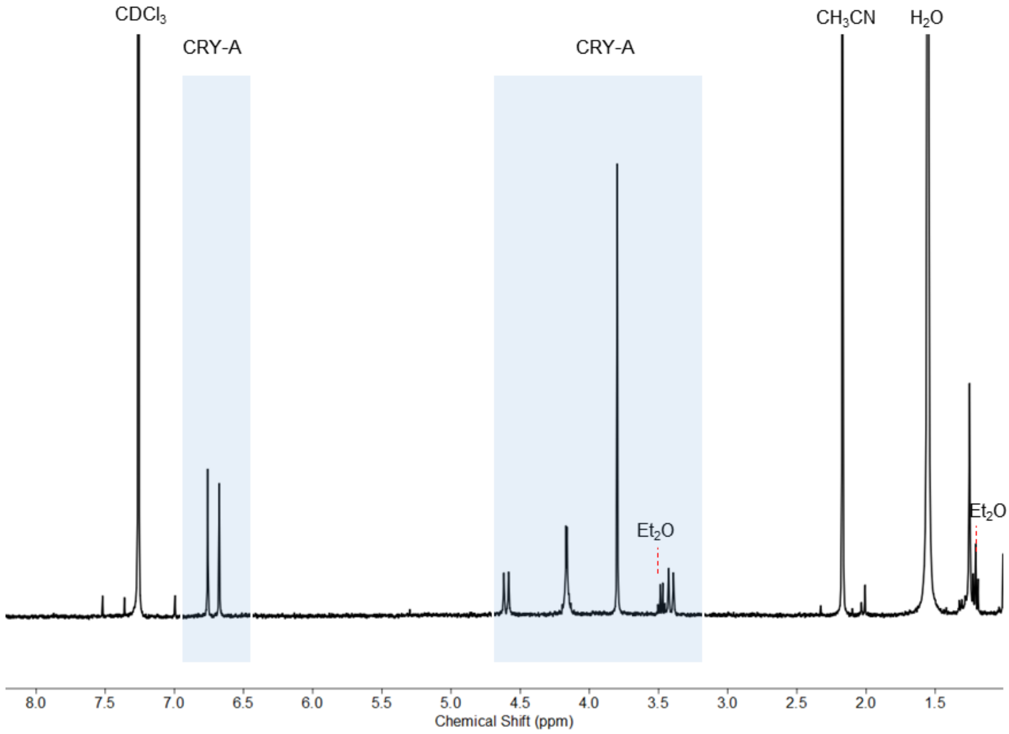

Figure S102. ^{1}H NMR spectrum of released CRY-A, with the peaks for the released guest highlighted with a light blue background (400 MHz, CD_3CN, 25 °C).

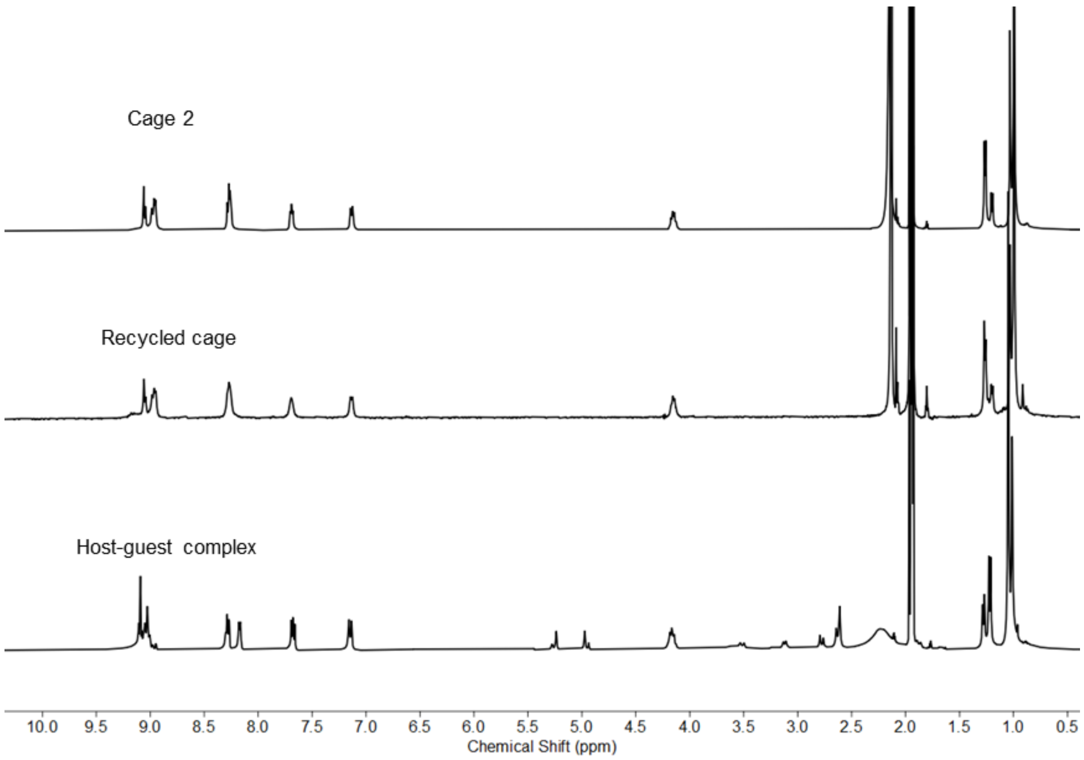

Figure S103. Comparison of the ^{1}H NMR spectra of cage **2**, recycled cage and host-guest complex (400 and 500 MHz, CD_3CN, 25 °C).

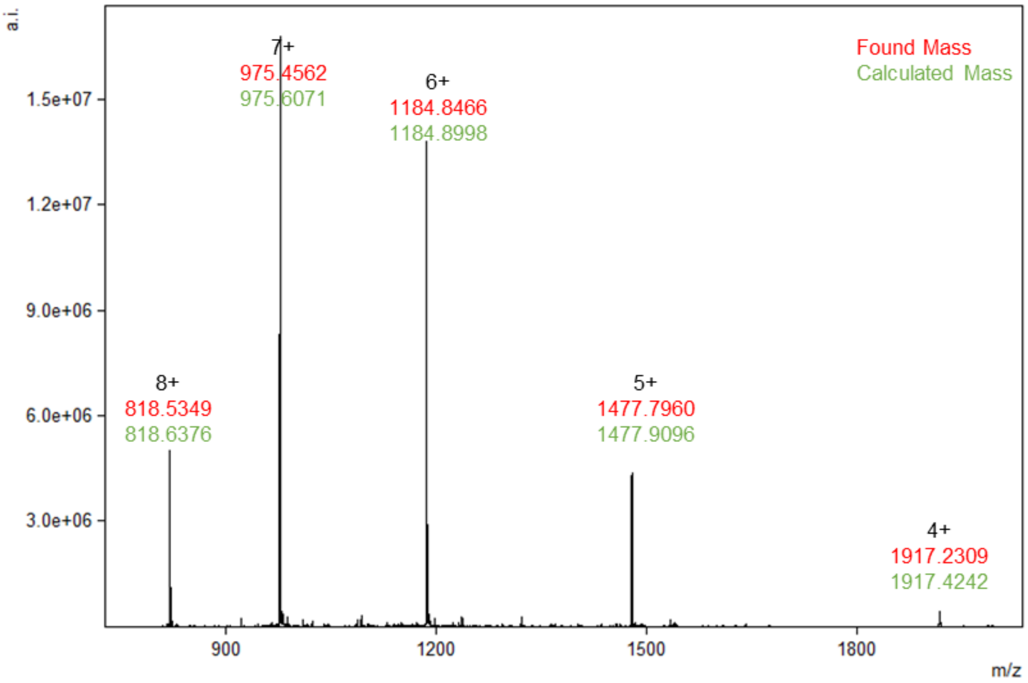

Figure S104. HR-ESI-MS spectrum of recycled cage.

We also attempted the enantioselective separation of racemic C_{70}PCBM (2 equiv) by cage **2** (1 equiv). However, the ^{1}H NMR spectrum of the resulting host-guest complex

was the same as that following the encapsulation of 1 equiv racemic C_{70}PCBM, as shown in Figure S59. Cotton effects corresponding to C_{70}PCBM were also not observed in the CD spectrum of the host-guest complex. These results indicate that cage **2** shows no binding preference for (*R*)- over (*S*)-C_{70}PCBM.

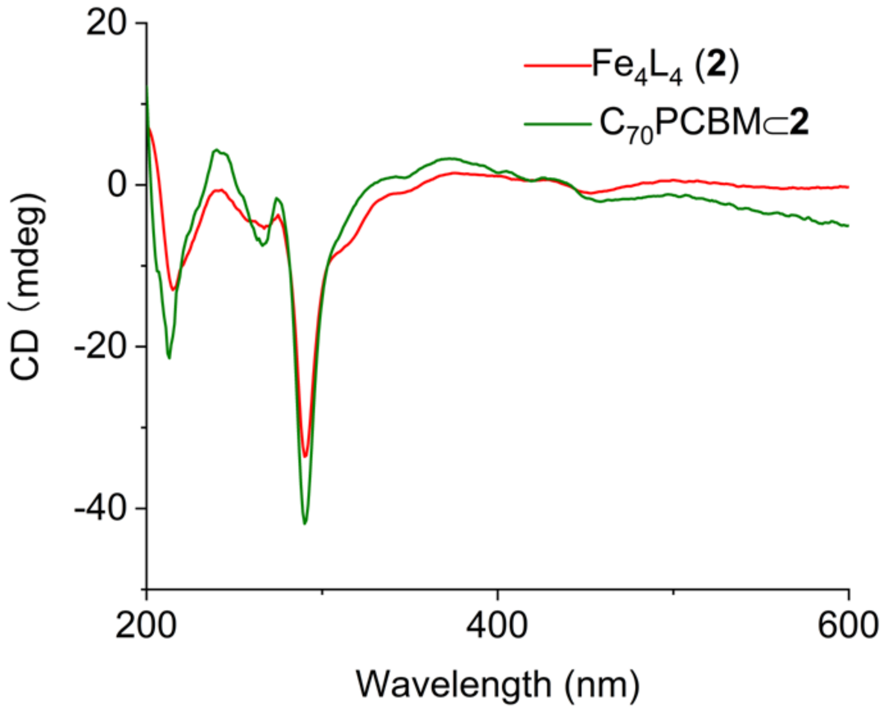

Figure S105. Comparison of the CD spectra of **2** and C_{70}PCBM⊂**2** obtained in the resolution experiment in MeCN.

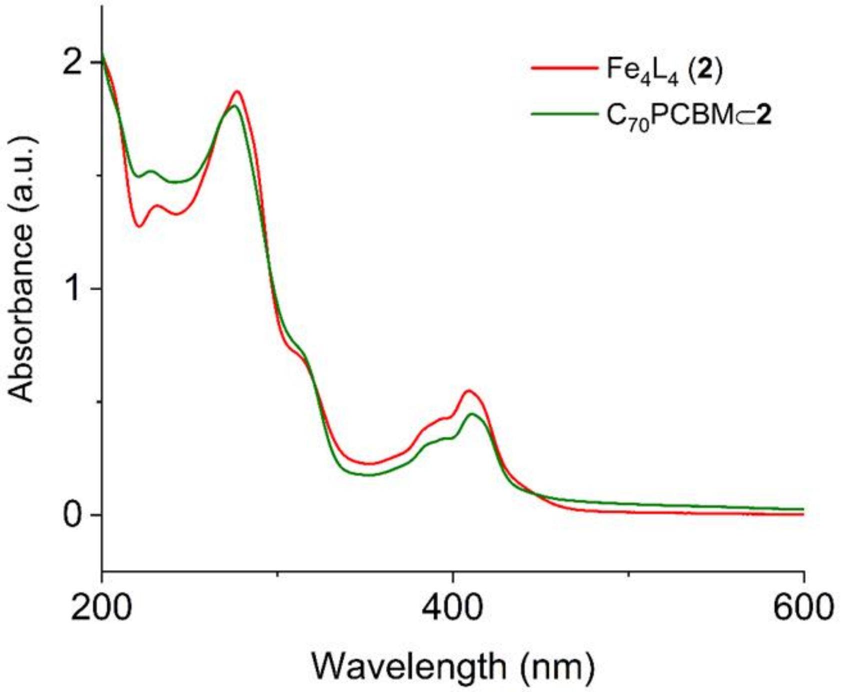

Figure S106. Comparison of the UV-vis spectra of **2** and C_{70}PCBM⊂**2** obtained in the resolution experiment in MeCN.

9 Volume Calculations

In order to determine the available void spaces within the structures of *Δ*$_4$-**2** and *Λ*$_4$-**2**, Molovol[10] calculations based on the DFT-optimized molecular structures were performed. A probe with a radius of 2.4 Å was employed for both diastereomers. The standard parameters are tabulated below, and the results are shown in Figure 88.

Probe mode: one probe

Probe radius: 2.4 Å

Grid resolution: 0.1 Å

Optimization depth: 4

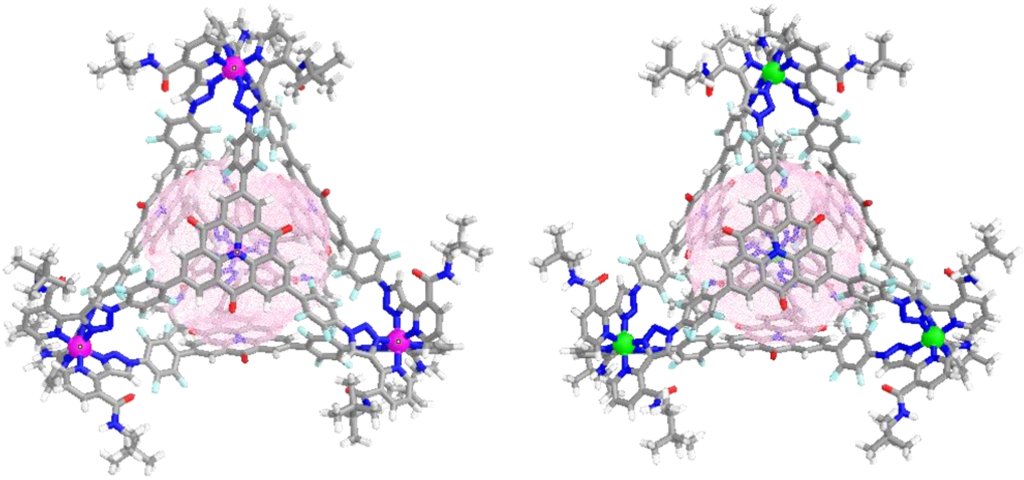

Δ$_4$-**2** 1281 Å^3 *Λ*$_4$-**2** 1266 Å^3

Figure 107. MoloVol-calculated void space (pink mesh) within the DFT-optimized molecular structures of *Δ*$_4$-**2** and *Λ*$_4$-**2**.

10 Computational Studies

The parametrization of the atomistic models of **2** and C_{70} was based on the General Amber Force Field (GAFF).[13] After a geometry optimization of each structure we estimated the partial charges by using the RESP approach.[14] The quantum mechanical estimations for this purpose were performed using Gaussian16 software[15] with a (U)B3LYP functional.[16] A 6-31G* basis set was used for this purpose. The bonded parameters of the organic components of the cage and C_{70} were considered using the parameters of GAFF, while the metal-coordination centers of **2** were parametrized following a variant of Seminario's method.[17,18] This estimation was done by analyzing a small portion of the coordination site, which consists of three triazole-pyridine ligands coordinating octahedrally to Fe^{II}. For this purpose, we used a 6-311G** basis set during geometry optimization and the estimation of the Hessian matrix needed within the Seminario's method framework. In such a coordination system, the central Fe^{II} ion can undergo a spin-state transition during the isomerization from *Δ* to *Λ* conformers (and *vice versa*) through Bailar Twist pathway[19] – i.e., different spin states may be more or less favored as function of the torsional angle. Preliminary tests demonstrated that the singlet state is more favored in the *Λ* and *Δ* configurations (see Figure S108: cyan), while the quintet spin state becomes more favored during the torsion at intermediate torsional configurations (in red). Capturing such complex electronic transitions via a classical all atom force field is not possible. Thus, in our simulations we parametrized the bonded terms of the metal center in such a way to have the equilibrium configuration of the cage fitting with the values of the singlet state, while in intermediate configurations during torsion the coordination metal group reproduces a behavior similar to the quintet state (this being more favored during torsion-induced deformations of the octahedral metal center). Complete details of the force field parameters used in all simulations conducted herein are available, see ref. 20

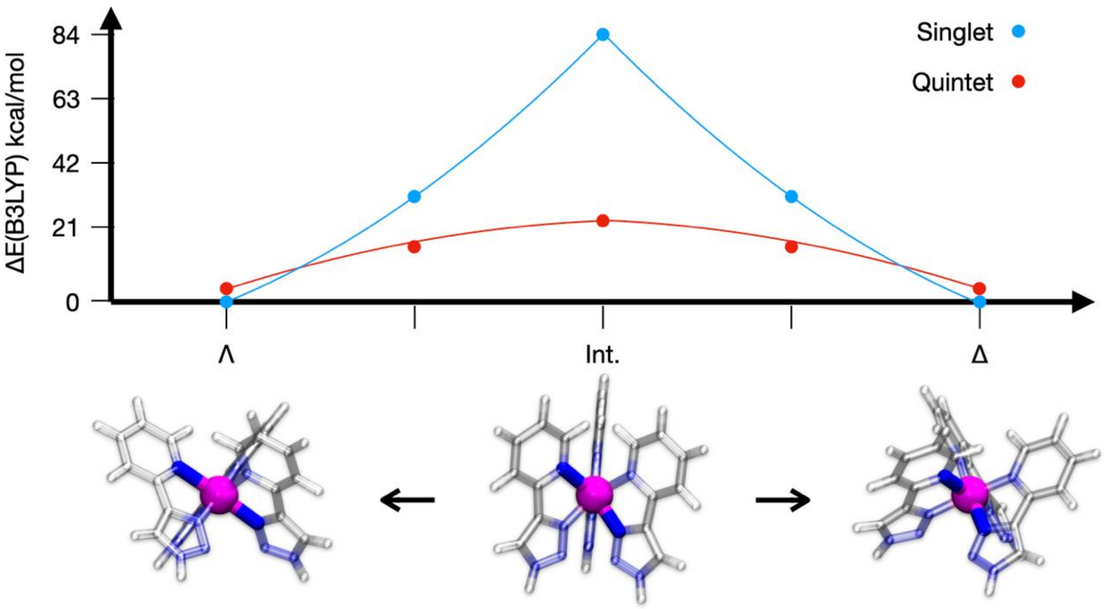

Figure S108. B3LYP Energy estimation of the configurations leading the transition from *Λ* to *Δ* passing through two intermediates. For each orientation we optimized the configurations for the singlet (blue) and quintet (red) spin states. The energies refer to the singlet *Δ* (equal to *Λ*) value. In order to perform the geometry optimization, the orientations of the coordinating molecules were restrained (i.e. by restraining the dihedrals involving the Fe as one of the two central atoms).

All the classical molecular dynamics (MD) simulations have been conducted in explicit acetonitrile solvent including 8 Tf_2N^- anions to neutralize the charge of **2**. We used the GROMACS-2020.2 software package[21,22] patched with Plumed-2.7.[23] The simulations were performed in cubic boxes with 6.1 nm sides. For every MD simulation geometrical optimization of the system followed by 10 ns of equilibration in the NVT at a 300 K temperature thermostat were performed, prior to a 0.5 μs production run in NPT at a 300 K temperature thermostat and a 1 atm pressure barostat. As a thermostat we used v-rescale[24] for NVT and NPT simulations, while a Berendsen barostat was used for NPT simulations.[25] The electrostatic interactions were treated using a Particle Mesh Ewald approach.[26] The cutoff of the real part of the electrostatic interaction was set to 1.0 nm. The cutoff of the van der Waals interaction was set to 1.0 nm. All bonds involving hydrogens were restrained by using the LINCS

algorithm.[27] The integration of the equation of motion was performed using the leap-frog algorithm.

In order to investigate the rare transition from *Δ* to *Λ* (and vice versa) we employed a validated approach[28-30] based on running replica infrequent well-tempered metadynamics simulations.[31-34] This biased simulation method allows exploration of transitions accompanied by high free energy barriers at high (atomistic) resolution along a reaction coordinate defined by a combination of chosen collective variables (CVs: descriptors of the transition process), and to reconstruct the unbiased kinetics expected for the transition from the biased transition trajectories.[28-34] In particular, considering one specific vertex of the coordination cage, we measured the 6-angles between the atoms of the coordination-site (centered on Fe) that should undergo transition passing from *Δ* to *Λ* (see the highlighted angle in Figure S108). Specifically, these angles are the intermolecular Pyridine(N)-Fe-Triazole(N) bond-angles (intramolecular ones are excluded as they do not undergo any transition during the *Δ*-*Λ* isomerization). In the minimum-energy *Δ* configuration three of these angles have a value of 180° (angles a_1, a_2, a_3) while the other three have a value of 90° (angles a_4, a_5, a_6). After the transition to Λ configuration, the new values are: angles a_1, a_2, a_3 = 90° and angles a_4, a_5, a_6 = 180°. We thus defined a CV as:

$$CV = \sum_{i=1}^{3} a_i - \sum_{i=4}^{6} a_i$$

Thus, we obtain a continuous CV ranging from -270° to +270° during the torsion between the two configurations *Λ* and *Δ* respectively, and well suited to activate the transition of one metal center (first necessary step for the transition) during the infrequent metadynamics simulations. In particular, we ran 50 infrequent metadynamics simulations biasing the *Λ*→*Δ* transitions and 50 biasing the *Δ*→*Λ* transitions. The metadynamics simulations were performed by depositing along the CV one Gaussian bias (1 kJ/mol height, 0.34° sigma, with 24 as bias factor) every 20 ps. In each simulation, we considered the time of the transition event (escape from the minimum configuration) when CV = 0. From the transition times observed during the biased simulations ($t_{i\mathrm{MTD}}$) we recalculated the corresponding transition time (t) as:

$$t = \sum_{i}^{n\mathrm{MTD}} \mathrm{d}t\, e^{\beta V(s(t_{i\mathrm{MTD}}),t_{i\mathrm{MTD}})}$$

Where β is the inverse of the product of Boltzman's constant and temperature, $V(s(t),t)$ is the time-dependent bias potential constructed during the metadynamics run in the system coordinates s at the time t, and $n\mathrm{MTD}$ is the total number of steps in the metadynamics.[28,35]

From the Poissonian fit (cumulative distribution function-best fit, $\mathrm{CDF_{BF}}$) of the empirical cumulative distribution of $\{t_i\}$, we can then estimate the characteristic transition times τ for the $\Delta_4 \rightarrow \Delta_3\Lambda$ and $\Lambda_3 \rightarrow \Lambda_3\Delta$:

$$CDF_{BF}(t) = 1 - e^{-\frac{t}{\tau}}$$

The associated energy barriers ($\Delta G^{\ddagger}$) are associated to the Eyring equation from the estimated τ:[36]

$$\frac{1}{\tau} = \frac{\kappa}{\beta h} e^{-\boldsymbol{\beta}\Delta \mathrm{G}^{\ddagger}}$$

where κ is the transmission coefficient (set to 1 in all cases studied herein, based on the fundamental no-recrossing assumption of transition state theory), and h is Planck's constant.

The DFT-optimized Cartesian coordinates of *Δ*$_4$-**2** and *Λ*$_4$-**2** are shown in Tables S3 and S4, respectively.

Table S3. Cartesian coordinates of *Δ*$_4$-**2**.

C	-5.428	4.59	3.68	C	-3.37	-4.831	-4.339
C	-4.304	5.298	3.248	C	-2.412	-4.775	-5.377
C	-4.187	5.691	1.895	C	-1.411	-5.745	-5.466
C	-5.249	5.378	1.017	C	-1.302	-6.775	-4.529
C	-6.374	4.693	1.486	C	-4.25	-6.034	-2.304
C	-6.475	4.271	2.813	C	-5.273	-4.973	-2.234
C	-3.247	5.594	4.234	C	-5.306	-3.914	-3.169
C	-2.068	6.295	3.69	N	-4.372	-3.847	-4.222
C	-1.983	6.647	2.324	C	-6.191	-5.014	-1.181
N	-3.037	6.361	1.433	C	-7.143	-4.008	-0.999
C	-1.005	6.565	4.556	C	-7.179	-2.968	-1.931
C	0.176	7.157	4.104	C	-6.292	-2.915	-3.009
C	0.253	7.512	2.756	C	-4.414	-2.773	-5.133
C	-0.803	7.28	1.872	C	-3.461	-2.664	-6.171
C	-2.922	6.706	0.072	C	-2.405	-3.676	-6.362
C	-3.949	6.39	-0.846	C	-6.404	-1.78	-3.945
C	-5.189	5.715	-0.418	C	-5.399	-1.765	-5.025
C	-0.635	7.692	0.465	C	-5.413	-0.693	-5.922
C	-1.77	7.363	-0.417	C	-4.45	-0.564	-6.925
C	-1.662	7.679	-1.775	C	-3.484	-1.567	-7.035
C	-2.659	7.334	-2.69	O	-4.192	-6.951	-1.491
C	-3.799	6.694	-2.201	O	-1.571	-3.599	-7.258
O	-3.332	5.262	5.412	O	-7.255	-0.905	-3.822
O	-6.091	5.434	-1.201	H	-2.236	-7.614	-2.757
O	0.381	8.244	0.056	H	-0.703	-5.642	-6.282
H	-5.449	4.303	4.726	H	-6.109	-5.85	-0.495
H	-7.151	4.479	0.76	H	-7.9	-2.162	-1.854
H	-1.132	6.265	5.591	H	-6.192	0.049	-5.782
H	1.138	7.986	2.343	H	-2.715	-1.533	-7.799
H	-0.75	8.178	-2.087	C	-0.176	-7.745	-4.58
H	-4.616	6.409	-2.856	C	0.668	-7.927	-3.479
C	-7.633	3.462	3.276	C	1.762	-8.786	-3.506
C	-7.444	2.215	3.882	C	2.031	-9.548	-4.652
C	-8.503	1.406	4.281	C	1.185	-9.392	-5.758
C	-9.825	1.847	4.123	C	0.12	-8.495	-5.723
C	-10.032	3.101	3.534	F	0.46	-7.228	-2.355
C	-8.958	3.876	3.105	F	2.547	-8.843	-2.428
F	-6.204	1.74	4.062	F	1.4	-10.103	-6.871
F	-8.223	0.204	4.79	F	-0.637	-8.378	-6.816
F	-11.276	3.566	3.369	N	3.139	-10.433	-4.706

F	-9.223	5.053	2.533	N	3.561	-11.05	-3.576
N	-10.924	1.043	4.523	N	4.612	-11.727	-3.918
N	-10.792	0.202	5.576	C	4.887	-11.595	-5.257
N	-11.915	-0.439	5.656	C	3.934	-10.741	-5.772
C	-12.804	-0.026	4.693	H	3.8	-10.34	-6.761
C	-12.155	0.933	3.945	C	6.064	-12.334	-5.72
H	-12.466	1.486	3.076	N	6.737	-12.908	-4.677
C	-14.101	-0.707	4.72	C	7.85	-13.62	-4.934
N	-14.114	-1.727	5.632	C	8.343	-13.812	-6.218
C	-15.234	-2.458	5.774	C	7.645	-13.253	-7.284
C	-16.39	-2.218	5.044	C	6.484	-12.508	-7.053
C	-16.391	-1.163	4.136	H	8.356	-14.043	-4.074
C	-15.244	-0.381	3.964	H	9.25	-14.385	-6.374
H	-15.19	-3.264	6.498	H	8.013	-13.375	-8.299
H	-17.266	-2.842	5.186	C	5.757	-11.854	-8.213
H	-17.276	-0.958	3.541	O	5.375	-10.686	-8.12
C	-15.232	0.732	2.934	N	5.602	-12.629	-9.306
O	-14.268	0.86	2.176	H	5.899	-13.596	-9.265
N	-16.333	1.512	2.915	C	5.002	-12.165	-10.575
H	-17.047	1.364	3.616	H	4.832	-11.095	-10.423
C	-16.565	2.598	1.939	C	6.039	-12.346	-11.692
H	-15.723	2.528	1.245	H	6.295	-13.399	-11.851
C	-17.861	2.291	1.176	H	6.958	-11.807	-11.442
H	-18.738	2.288	1.834	H	5.669	-11.947	-12.64
H	-17.792	1.307	0.7	C	3.608	-12.832	-10.827
H	-18.039	3.028	0.39	C	3.72	-14.362	-10.976
C	-16.496	4.005	2.623	H	4.119	-14.839	-10.071
C	-17.597	4.182	3.688	H	4.352	-14.651	-11.822
H	-17.507	3.452	4.503	H	2.73	-14.796	-11.151
H	-18.602	4.1	3.264	C	2.67	-12.505	-9.647
H	-17.521	5.175	4.145	H	3.035	-12.935	-8.707
C	-15.115	4.178	3.287	H	2.563	-11.422	-9.508
H	-14.958	3.452	4.094	H	1.673	-12.919	-9.832
H	-14.303	4.06	2.559	C	2.998	-12.238	-12.114
H	-15.029	5.178	3.725	H	2.967	-11.142	-12.074
C	-16.655	5.093	1.54	H	3.552	-12.53	-13.01
H	-15.933	4.957	0.726	H	1.969	-12.594	-12.239
H	-17.659	5.103	1.106	C	-8.052	-4.014	0.178
H	-16.482	6.083	1.975	C	-8.109	-2.921	1.049
C	1.335	7.352	5.015	C	-8.907	-2.909	2.189
C	2.592	6.822	4.705	C	-9.733	-4.005	2.48
C	3.687	6.939	5.556	C	-9.702	-5.101	1.608
C	3.57	7.649	6.76	C	-8.865	-5.107	0.496
C	2.323	8.202	7.078	F	-7.343	-1.844	0.822

C	1.23	8.037	6.231	F	-8.844	-1.847	2.994
F	2.766	6.135	3.568	F	-10.479	-6.165	1.843
F	4.828	6.343	5.203	F	-8.867	-6.19	-0.285
F	2.167	8.893	8.213	N	-10.559	-4.018	3.634
F	0.064	8.572	6.6	N	-11.07	-2.857	4.108
N	4.671	7.785	7.645	N	-11.712	-3.172	5.189
N	5.93	7.831	7.148	C	-11.662	-4.522	5.433
N	6.713	7.885	8.18	C	-10.899	-5.076	4.427
C	6.002	7.902	9.355	H	-10.577	-6.088	4.255
C	4.669	7.824	9.009	C	-12.367	-4.956	6.641
H	3.781	7.77	9.614	N	-12.828	-3.889	7.363
C	6.819	7.963	10.569	C	-13.496	-4.119	8.509
N	8.153	7.856	10.281	C	-13.752	-5.395	8.99
C	9.04	7.888	11.292	C	-13.31	-6.484	8.245
C	8.667	8.039	12.621	C	-12.615	-6.282	7.048
C	7.314	8.177	12.915	H	-13.828	-3.242	9.052
C	6.363	8.15	11.889	H	-14.286	-5.53	9.925
H	10.083	7.786	11.015	H	-13.485	-7.495	8.601
H	9.419	8.049	13.402	C	-12.086	-7.47	6.267
H	6.989	8.284	13.945	O	-10.936	-7.453	5.822
C	4.886	8.245	12.219	N	-12.946	-8.501	6.135
O	4.083	7.48	11.681	H	-13.889	-8.398	6.487
N	4.559	9.185	13.129	C	-12.608	-9.787	5.489
H	5.282	9.804	13.473	H	-11.54	-9.71	5.265
C	3.193	9.39	13.658	C	-12.816	-10.911	6.514
H	2.605	8.579	13.221	H	-13.864	-10.998	6.824
C	3.234	9.205	15.181	H	-12.213	-10.72	7.408
H	3.86	9.958	15.673	H	-12.509	-11.877	6.107
H	3.634	8.216	15.429	C	-13.363	-9.96	4.129
H	2.232	9.274	15.612	C	-14.893	-10	4.32
C	2.581	10.74	13.153	H	-15.28	-9.071	4.761
C	3.387	11.958	13.646	H	-15.21	-10.836	4.951
H	4.423	11.945	13.28	H	-15.39	-10.12	3.352
H	3.411	12.026	14.738	C	-13	-8.787	3.195
H	2.934	12.884	13.276	H	-13.34	-7.826	3.599
C	2.556	10.739	11.611	H	-11.917	-8.724	3.034
H	3.567	10.7	11.188	H	-13.477	-8.919	2.218
H	1.99	9.884	11.22	C	-12.899	-11.273	3.464
H	2.081	11.653	11.24	H	-11.806	-11.312	3.375
C	1.129	10.85	13.663	H	-13.227	-12.157	4.018
H	0.541	9.964	13.392	H	-13.317	-11.35	2.455
H	1.078	10.972	14.749	C	-4.42	0.628	-7.813
H	0.64	11.722	13.217	C	-3.272	1.421	-7.916
C	-2.488	7.591	-4.145	C	-3.222	2.573	-8.694

C	-2.589	6.552	-5.076	C	-4.337	2.959	-9.453
C	-2.384	6.742	-6.439	C	-5.49	2.167	-9.376
C	-2.107	8.023	-6.936	C	-5.53	1.041	-8.558
C	-2.02	9.079	-6.021	F	-2.175	1.102	-7.216
C	-2.188	8.858	-4.656	F	-2.103	3.299	-8.677
F	-2.853	5.305	-4.66	F	-6.575	2.493	-10.088
F	-2.437	5.676	-7.24	F	-6.665	0.339	-8.513
F	-1.764	10.32	-6.451	N	-4.312	4.128	-10.257
F	-2.074	9.902	-3.832	N	-3.149	4.529	-10.823
N	-1.897	8.246	-8.322	N	-3.419	5.643	-11.428
N	-2.541	7.477	-9.232	C	-4.744	5.982	-11.299
N	-2.103	7.86	-10.389	C	-5.327	5.001	-10.524
C	-1.198	8.886	-10.274	H	-6.329	4.894	-10.15
C	-1.052	9.132	-8.925	C	-5.127	7.232	-11.958
H	-0.419	9.82	-8.393	N	-4.037	7.886	-12.465
C	-0.657	9.375	-11.544	C	-4.219	9.059	-13.098
N	-1.072	8.606	-12.596	C	-5.47	9.636	-13.275
C	-0.656	8.92	-13.836	C	-6.584	8.96	-12.785
C	0.17	10.004	-14.103	C	-6.432	7.737	-12.123
C	0.572	10.806	-13.04	H	-3.325	9.546	-13.469
C	0.157	10.51	-11.737	H	-5.566	10.589	-13.783
H	-1	8.272	-14.634	H	-7.574	9.391	-12.897
H	0.488	10.211	-15.12	C	-7.642	7.032	-11.542
H	1.228	11.654	-13.215	O	-7.594	6.563	-10.403
C	0.641	11.354	-10.575	N	-8.726	6.992	-12.345
O	1.047	10.81	-9.545	H	-8.649	7.353	-13.287
N	0.608	12.687	-10.778	C	-10.035	6.432	-11.946
H	0.204	13.04	-11.635	H	-9.914	6.178	-10.889
C	1.113	13.687	-9.812	C	-11.09	7.539	-12.07
H	1.569	13.093	-9.015	H	-11.214	7.878	-13.105
C	2.209	14.511	-10.502	H	-10.802	8.403	-11.463
H	1.822	15.085	-11.352	H	-12.064	7.195	-11.715
H	2.999	13.85	-10.871	C	-10.347	5.105	-12.715
H	2.667	15.218	-9.805	C	-10.456	5.332	-14.236
C	-0.057	14.51	-9.176	H	-9.519	5.712	-14.667
C	-0.828	15.326	-10.233	H	-11.259	6.03	-14.495
H	-1.294	14.686	-10.994	H	-10.675	4.387	-14.743
H	-0.188	16.055	-10.742	C	-9.228	4.081	-12.434
H	-1.638	15.888	-9.756	H	-8.259	4.422	-12.819
C	-1.032	13.542	-8.474	H	-9.119	3.891	-11.36
H	-1.508	12.857	-9.185	H	-9.457	3.127	-12.922
H	-0.521	12.942	-7.711	C	-11.678	4.527	-12.19
H	-1.83	14.105	-7.977	H	-11.665	4.417	-11.099
C	0.526	15.468	-8.117	H	-12.536	5.15	-12.459

H	1.142	14.93	-7.386	H	-11.851	3.535	-12.621
H	1.138	16.257	-8.563	C	2.607	-0.431	7.555
H	-0.286	15.959	-7.569	C	1.673	-1.418	7.233
C	6.304	-2.592	-4.202	C	2.073	-2.574	6.525
C	6.091	-1.216	-4.312	C	3.439	-2.709	6.19
C	6.527	-0.344	-3.289	C	4.356	-1.718	6.55
C	7.208	-0.903	-2.184	C	3.957	-0.558	7.218
C	7.43	-2.281	-2.115	C	0.269	-1.202	7.631
C	6.968	-3.149	-3.107	C	-0.667	-2.261	7.208
C	5.382	-0.716	-5.506	C	-0.227	-3.389	6.479
C	5.147	0.741	-5.523	N	1.133	-3.554	6.149
C	5.579	1.572	-4.465	C	-2.023	-2.1	7.508
N	6.275	1.04	-3.362	C	-2.983	-3.018	7.078
C	4.438	1.28	-6.6	C	-2.543	-4.136	6.366
C	4.108	2.636	-6.655	C	-1.192	-4.339	6.075
C	4.545	3.456	-5.613	C	1.546	-4.678	5.406
C	5.279	2.951	-4.537	C	2.898	-4.841	5.028
C	6.681	1.886	-2.311	C	3.934	-3.864	5.415
C	7.343	1.366	-1.175	C	-0.818	-5.55	5.32
C	7.66	-0.07	-1.053	C	0.619	-5.665	5.003
C	5.702	3.892	-3.482	C	1.04	-6.762	4.247
C	6.424	3.275	-2.354	C	2.368	-6.906	3.841
C	6.813	4.098	-1.293	C	3.284	-5.934	4.249
C	7.434	3.581	-0.153	O	-0.096	-0.206	8.248
C	7.695	2.209	-0.118	O	5.112	-3.99	5.098
O	4.998	-1.457	-6.404	O	-1.642	-6.386	4.963
O	8.234	-0.538	-0.076	H	2.233	0.439	8.084
O	5.452	5.092	-3.529	H	5.389	-1.88	6.259
H	5.93	-3.208	-5.013	H	-2.296	-1.21	8.066
H	7.954	-2.647	-1.238	H	-3.235	-4.894	6.013
H	4.13	0.587	-7.376	H	0.278	-7.482	3.966
H	4.333	4.52	-5.6	H	4.333	-5.992	3.978
H	6.582	5.154	-1.385	C	4.925	0.532	7.512
H	8.184	1.746	0.732	C	4.695	1.839	7.069
C	7.128	-4.621	-2.976	C	5.601	2.873	7.278
C	6.02	-5.474	-3.035	C	6.782	2.639	7.997
C	6.123	-6.851	-2.864	C	7.021	1.34	8.464
C	7.378	-7.444	-2.666	C	6.119	0.311	8.207
C	8.5	-6.607	-2.622	F	3.583	2.124	6.377
C	8.369	-5.227	-2.754	F	5.324	4.071	6.76
F	4.795	-4.966	-3.223	F	8.132	1.072	9.161
F	5.001	-7.574	-2.872	F	6.412	-0.909	8.662
F	9.719	-7.13	-2.442	N	7.723	3.676	8.227
F	9.475	-4.482	-2.683	N	7.296	4.955	8.355

N	7.511	-8.846	-2.492	N	8.367	5.672	8.482
N	6.644	-9.685	-3.107	C	9.501	4.897	8.467
N	6.966	-10.876	-2.71	C	9.084	3.595	8.287
C	8.046	-10.853	-1.862	H	9.641	2.681	8.179
C	8.396	-9.527	-1.707	C	10.756	5.639	8.607
H	9.154	-9.06	-1.105	N	10.544	6.991	8.591
C	8.47	-12.165	-1.369	C	11.601	7.816	8.704
N	7.605	-13.151	-1.759	C	12.904	7.359	8.849
C	7.851	-14.419	-1.381	C	13.121	5.985	8.894
C	8.955	-14.779	-0.62	C	12.045	5.098	8.782
C	9.853	-13.784	-0.246	H	11.384	8.877	8.672
C	9.63	-12.455	-0.623	H	13.725	8.063	8.926
H	7.13	-15.161	-1.702	H	14.13	5.596	8.995
H	9.105	-15.813	-0.331	C	12.292	3.602	8.776
H	10.719	-14.032	0.36	O	11.727	2.886	7.946
C	10.58	-11.366	-0.166	N	13.164	3.158	9.705
O	10.135	-10.308	0.285	H	13.534	3.815	10.38
N	11.892	-11.664	-0.272	C	13.614	1.754	9.82
H	12.16	-12.536	-0.708	H	13.163	1.251	8.96
C	12.984	-10.783	0.195	C	15.141	1.724	9.665
H	12.474	-9.954	0.692	H	15.649	2.267	10.47
C	13.809	-11.551	1.237	H	15.431	2.18	8.713
H	14.299	-12.433	0.808	H	15.516	0.698	9.668
H	13.163	-11.886	2.055	C	13.049	1.077	11.113
H	14.587	-10.916	1.668	C	13.557	1.767	12.394
C	13.791	-10.187	-1.006	H	13.246	2.819	12.451
C	14.489	-11.285	-1.833	H	14.647	1.727	12.483
H	13.773	-11.99	-2.277	H	13.144	1.269	13.278
H	15.209	-11.857	-1.24	C	11.507	1.136	11.083
H	15.043	-10.835	-2.664	H	11.139	2.168	11.108
C	12.83	-9.398	-1.92	H	11.106	0.653	10.184
H	12.072	-10.049	-2.371	H	11.092	0.618	11.955
H	12.313	-8.605	-1.367	C	13.479	-0.405	11.127
H	13.387	-8.929	-2.738	H	13.197	-0.913	10.197
C	14.853	-9.21	-0.458	H	14.558	-0.523	11.266
H	14.401	-8.454	0.196	H	12.988	-0.93	11.954
H	15.637	-9.724	0.106	C	-4.431	-2.787	7.321
H	15.341	-8.684	-1.285	C	-5.345	-2.763	6.262
C	3.272	3.179	-7.758	C	-6.698	-2.497	6.443
C	2.085	3.869	-7.49	C	-7.207	-2.284	7.733
C	1.249	4.346	-8.495	C	-6.31	-2.322	8.808
C	1.605	4.184	-9.842	C	-4.952	-2.549	8.598
C	2.8	3.511	-10.129	F	-4.919	-2.961	5.007
C	3.6	3.006	-9.107	F	-7.48	-2.431	5.364

F	1.692	4.056	-6.222	F	-6.752	-2.13	10.056
F	0.105	4.933	-8.14	F	-4.145	-2.553	9.662
F	3.184	3.339	-11.4	N	-8.584	-2.013	7.947
F	4.717	2.357	-9.447	N	-9.51	-2.55	7.118
N	0.773	4.663	-10.887	N	-10.65	-2.076	7.512
N	0.018	5.77	-10.691	C	-10.508	-1.254	8.603
N	-0.675	5.919	-11.775	C	-9.158	-1.2	8.881
C	-0.382	4.948	-12.702	H	-8.607	-0.648	9.621
C	0.555	4.119	-12.12	C	-11.756	-0.679	9.108
H	1.026	3.221	-12.477	N	-12.814	-0.98	8.292
C	-1.128	5.05	-13.958	C	-14.036	-0.519	8.615
N	-2.079	6.033	-13.898	C	-14.28	0.242	9.75
C	-2.857	6.251	-14.974	C	-13.213	0.526	10.597
C	-2.733	5.53	-16.154	C	-11.929	0.062	10.294
C	-1.749	4.549	-16.229	H	-14.837	-0.771	7.931
C	-0.92	4.297	-15.13	H	-15.281	0.6	9.963
H	-3.604	7.029	-14.873	H	-13.37	1.129	11.487
H	-3.391	5.735	-16.991	C	-10.76	0.42	11.19
H	-1.635	3.959	-17.133	O	-9.698	0.805	10.694
C	0.113	3.189	-15.196	N	-10.992	0.306	12.514
O	0.236	2.404	-14.254	H	-11.878	-0.073	12.823
N	0.829	3.137	-16.339	C	-10.022	0.685	13.564
H	0.695	3.862	-17.032	H	-9.19	1.138	13.017
C	1.825	2.089	-16.648	C	-10.669	1.757	14.452
H	1.749	1.384	-15.815	H	-11.551	1.377	14.982
C	1.389	1.377	-17.937	H	-10.981	2.611	13.843
H	1.388	2.051	-18.801	H	-9.965	2.124	15.202
H	0.378	0.974	-17.82	C	-9.466	-0.573	14.311
H	2.054	0.542	-18.168	C	-10.578	-1.34	15.054
C	3.28	2.664	-16.648	H	-11.351	-1.716	14.37
C	3.476	3.737	-17.738	H	-11.067	-0.726	15.818
H	2.815	4.602	-17.592	H	-10.157	-2.213	15.564
H	3.307	3.342	-18.744	C	-8.798	-1.515	13.289
H	4.503	4.117	-17.71	H	-9.519	-1.902	12.559
C	3.579	3.284	-15.267	H	-7.997	-1.005	12.738
H	2.926	4.139	-15.055	H	-8.356	-2.376	13.801
H	3.451	2.55	-14.462	C	-8.396	-0.117	15.325
H	4.613	3.645	-15.23	H	-7.623	0.494	14.844
C	4.273	1.507	-16.887	H	-8.825	0.462	16.149
H	4.116	0.69	-16.173	H	-7.903	-0.99	15.767
H	4.195	1.096	-17.898	C	2.785	-8.028	2.958
H	5.301	1.864	-16.762	C	3.425	-7.785	1.738
C	7.757	4.452	1.008	C	3.783	-8.804	0.861
C	7.272	4.155	2.287	C	3.545	-10.143	1.204

C	7.509	4.973	3.387	C	2.92	-10.406	2.43
C	8.297	6.125	3.252	C	2.534	-9.368	3.274
C	8.806	6.427	1.982	F	3.68	-6.526	1.356
C	8.522	5.616	0.886	F	4.33	-8.469	-0.309
F	6.514	3.067	2.479	F	2.68	-11.669	2.802
F	6.95	4.641	4.553	F	1.926	-9.683	4.419
F	9.569	7.513	1.807	N	3.901	-11.202	0.33
F	9.02	5.969	-0.301	N	4.978	-11.07	-0.482
N	8.556	6.975	4.358	N	5.01	-12.148	-1.2
N	8.633	6.451	5.605	C	3.993	-13.01	-0.868
N	8.805	7.46	6.4	C	3.26	-12.389	0.122
C	8.869	8.645	5.708	H	2.365	-12.69	0.636
C	8.694	8.333	4.376	C	3.964	-14.254	-1.639
H	8.637	8.958	3.503	N	4.895	-14.246	-2.642
C	9.062	9.834	6.541	C	4.991	-15.318	-3.449
N	9.005	9.529	7.873	C	4.196	-16.446	-3.303
C	9.158	10.516	8.774	C	3.267	-16.471	-2.267
C	9.385	11.838	8.414	C	3.141	-15.374	-1.408
C	9.472	12.149	7.061	H	5.733	-15.257	-4.237
C	9.32	11.147	6.096	H	4.304	-17.282	-3.985
H	9.091	10.227	9.816	H	2.622	-17.334	-2.135
H	9.491	12.601	9.177	C	2.09	-15.383	-0.315
H	9.636	13.175	6.747	O	1.381	-14.39	-0.133
C	9.359	11.498	4.622	N	1.997	-16.532	0.386
O	8.515	11.033	3.854	H	2.662	-17.272	0.2
N	10.344	12.346	4.258	C	0.986	-16.786	1.435
H	11.024	12.63	4.952	H	0.338	-15.906	1.41
C	10.513	12.886	2.892	C	0.165	-18.017	1.025
H	9.642	12.519	2.343	H	0.777	-18.924	0.973
C	10.441	14.418	2.967	H	-0.286	-17.855	0.04
H	11.26	14.841	3.559	H	-0.645	-18.202	1.733
H	9.496	14.727	3.425	C	1.641	-16.849	2.856
H	10.487	14.862	1.97	C	2.644	-18.013	2.977
C	11.788	12.299	2.199	H	3.479	-17.918	2.269
C	13.08	12.687	2.946	H	2.171	-18.988	2.818
H	13.097	12.301	3.974	H	3.083	-18.029	3.98
H	13.228	13.771	2.984	C	2.371	-15.52	3.136
H	13.951	12.261	2.436	H	3.201	-15.357	2.439
C	11.674	10.761	2.149	H	1.689	-14.664	3.06
H	11.658	10.322	3.154	H	2.79	-15.525	4.148
H	10.765	10.444	1.624	C	0.528	-17.025	3.91
H	12.533	10.335	1.62	H	-0.246	-16.253	3.808
C	11.857	12.829	0.752	H	0.042	-18.003	3.842
H	10.924	12.634	0.209	H	0.949	-16.942	4.917

H	12.056	13.904	0.712	Fe	-2.237	7.011	-12.126
H	12.667	12.33	0.209	Fe	5.988	-12.545	-2.825
C	-2.256	-6.833	-3.51	Fe	-12.374	-2.053	6.626
C	-3.285	-5.894	-3.411	Fe	8.622	7.587	8.325

Table S4. Cartesian coordinates of Λ_4-**2**.

C	7.469	1.146	2.59	C	-0.067	-0.307	-7.288
C	6.514	1.881	3.297	C	-1.289	0.386	-7.444
C	6.004	3.086	2.763	C	-2.48	-0.319	-7.638
C	6.513	3.532	1.522	C	-2.511	-1.716	-7.654
C	7.488	2.789	0.851	C	1.14	-2.52	-7.198
C	7.967	1.58	1.359	C	2.369	-1.735	-6.971
C	6.046	1.348	4.591	C	2.343	-0.324	-6.901
C	5	2.151	5.251	N	1.139	0.389	-7.07
C	4.5	3.338	4.668	C	3.567	-2.429	-6.779
N	5.004	3.811	3.441	C	4.76	-1.765	-6.485
C	4.474	1.683	6.458	C	4.732	-0.37	-6.426
C	3.43	2.344	7.109	C	3.556	0.352	-6.64
C	2.945	3.521	6.535	C	1.134	1.795	-6.979
C	3.469	4.029	5.344	C	-0.069	2.526	-7.104
C	4.48	4.988	2.871	C	-1.359	1.857	-7.36
C	4.942	5.458	1.62	C	3.62	1.824	-6.561
C	6.005	4.757	0.875	C	2.329	2.513	-6.744
C	2.897	5.281	4.812	C	2.304	3.906	-6.634
C	3.465	5.724	3.524	C	1.112	4.628	-6.721
C	2.94	6.877	2.935	C	-0.066	3.916	-6.962
C	3.369	7.323	1.683	O	1.138	-3.746	-7.242
C	4.378	6.598	1.044	O	-2.412	2.476	-7.472
O	6.479	0.305	5.07	O	4.664	2.428	-6.336
O	6.419	5.15	-0.211	H	-1.253	-3.48	-7.523
O	2.003	5.894	5.385	H	-3.384	0.271	-7.746
H	7.807	0.222	3.046	H	3.518	-3.511	-6.841
H	7.833	3.184	-0.099	H	5.626	0.208	-6.213
H	4.897	0.764	6.85	H	3.254	4.397	-6.449
H	2.143	4.089	6.995	H	-1.024	4.416	-7.051
H	2.16	7.394	3.486	C	-3.796	-2.455	-7.768
H	4.765	6.896	0.076	C	-4.171	-3.401	-6.809
C	8.936	0.753	0.591	C	-5.388	-4.074	-6.853
C	8.654	-0.58	0.276	C	-6.278	-3.85	-7.913
C	9.511	-1.37	-0.484	C	-5.911	-2.919	-8.893
C	10.736	-0.852	-0.93	C	-4.706	-2.226	-8.806
C	11.042	0.476	-0.608	F	-3.362	-3.659	-5.772
C	10.151	1.261	0.119	F	-5.683	-4.911	-5.856

F	7.5	-1.132	0.675	F	-6.726	-2.679	-9.927
F	9.124	-2.61	-0.788	F	-4.423	-1.338	-9.762
F	12.203	1.011	-1.006	N	-7.524	-4.526	-7.985
F	10.494	2.525	0.377	N	-7.638	-5.776	-7.477
N	11.631	-1.636	-1.704	N	-8.885	-6.1	-7.616
N	11.679	-2.976	-1.516	C	-9.603	-5.103	-8.23
N	12.516	-3.425	-2.398	C	-8.72	-4.069	-8.461
C	13.048	-2.41	-3.156	H	-8.867	-3.092	-8.886
C	12.462	-1.242	-2.712	C	-11.026	-5.398	-8.417
H	12.563	-0.226	-3.05	N	-11.375	-6.577	-7.815
C	14.002	-2.843	-4.18	C	-12.653	-6.992	-7.884
N	14.068	-4.209	-4.255	C	-13.642	-6.282	-8.551
C	14.892	-4.774	-5.156	C	-13.287	-5.098	-9.189
C	15.694	-4.034	-6.013	C	-11.966	-4.638	-9.142
C	15.652	-2.646	-5.922	H	-12.88	-7.924	-7.379
C	14.81	-2.023	-4.994	H	-14.661	-6.651	-8.57
H	14.897	-5.857	-5.184	H	-14.041	-4.514	-9.707
H	16.334	-4.534	-6.731	C	-11.602	-3.319	-9.798
H	16.257	-2.04	-6.589	O	-10.864	-2.522	-9.216
C	14.735	-0.51	-4.939	N	-12.164	-3.104	-11.007
O	13.643	0.052	-4.834	H	-12.68	-3.856	-11.444
N	15.919	0.129	-5.044	C	-11.968	-1.875	-11.806
H	16.77	-0.419	-5.029	H	-11.323	-1.245	-11.187
C	16.072	1.599	-5.044	C	-11.211	-2.246	-13.089
H	15.048	1.982	-5.019	H	-11.791	-2.92	-13.73
C	16.786	2.017	-3.751	H	-10.973	-1.355	-13.675
H	16.234	1.646	-2.881	H	-10.268	-2.744	-12.84
H	17.807	1.622	-3.697	C	-13.319	-1.111	-12.01
H	16.846	3.105	-3.668	C	-13.041	0.189	-12.793
C	16.729	2.098	-6.375	H	-12.74	-0.003	-13.828
C	16.809	3.639	-6.343	H	-13.946	0.804	-12.829
H	17.523	4.004	-5.598	H	-12.255	0.786	-12.313
H	17.138	4.015	-7.317	C	-14.349	-1.958	-12.783
H	15.831	4.087	-6.128	H	-13.999	-2.229	-13.784
C	18.146	1.521	-6.57	H	-14.613	-2.882	-12.25
H	18.828	1.809	-5.764	H	-15.28	-1.395	-12.908
H	18.143	0.425	-6.643	C	-13.897	-0.734	-10.631
H	18.577	1.894	-7.505	H	-14.146	-1.619	-10.034
C	15.838	1.682	-7.563	H	-13.19	-0.127	-10.053
H	15.762	0.593	-7.658	H	-14.817	-0.152	-10.752
H	14.823	2.082	-7.46	C	6.007	-2.519	-6.192
H	16.255	2.064	-8.501	C	6.706	-2.319	-4.997
C	2.818	1.785	8.344	C	7.848	-3.042	-4.664
C	1.443	1.537	8.418	C	8.37	-3.986	-5.56

C	0.841	0.967	9.536	C	7.693	-4.186	-6.77
C	1.61	0.657	10.667	C	6.529	-3.481	-7.064
C	2.985	0.916	10.617	F	6.257	-1.43	-4.1
C	3.572	1.449	9.473	F	8.406	-2.825	-3.472
F	0.656	1.812	7.369	F	8.158	-5.071	-7.659
F	-0.467	0.709	9.486	F	5.92	-3.731	-8.226
F	3.757	0.644	11.676	N	9.534	-4.735	-5.248
F	4.892	1.654	9.482	N	10.493	-4.185	-4.466
N	1.022	0.079	11.823	N	11.382	-5.112	-4.295
N	-0.261	0.374	12.139	C	11.05	-6.264	-4.967
N	-0.541	-0.346	13.179	C	9.838	-6.024	-5.58
C	0.536	-1.098	13.581	H	9.205	-6.66	-6.173
C	1.554	-0.832	12.689	C	12.009	-7.361	-4.815
H	2.551	-1.229	12.612	N	12.99	-7.046	-3.913
C	0.292	-1.943	14.753	C	13.949	-7.952	-3.648
N	-1.023	-1.902	15.133	C	13.998	-9.198	-4.258
C	-1.42	-2.632	16.19	C	13.02	-9.513	-5.196
C	-0.552	-3.425	16.929	C	12.01	-8.593	-5.501
C	0.79	-3.449	16.564	H	14.693	-7.659	-2.918
C	1.24	-2.698	15.473	H	14.785	-9.9	-4.003
H	-2.473	-2.576	16.442	H	13.025	-10.485	-5.679
H	-0.921	-4.006	17.767	C	10.921	-8.967	-6.488
H	1.491	-4.07	17.113	O	9.744	-8.692	-6.244
C	2.697	-2.771	15.054	N	11.344	-9.624	-7.588
O	2.994	-2.862	13.861	H	12.339	-9.72	-7.746
N	3.587	-2.757	16.068	C	10.449	-10.103	-8.664
H	3.252	-2.585	17.007	H	9.445	-9.83	-8.328
C	5.052	-2.848	15.889	C	10.77	-9.322	-9.947
H	5.192	-2.936	14.808	H	10.678	-8.246	-9.765
C	5.682	-1.53	16.362	H	11.786	-9.518	-10.307
H	5.531	-1.362	17.434	H	10.076	-9.584	-10.749
H	6.757	-1.519	16.172	C	10.497	-11.663	-8.788
H	5.239	-0.686	15.822	C	11.906	-12.169	-9.154
C	5.625	-4.149	16.545	H	12.652	-11.914	-8.389
C	7.141	-4.219	16.269	H	11.904	-13.261	-9.234
H	7.698	-3.442	16.8	H	12.254	-11.776	-10.115
H	7.536	-5.185	16.6	C	10.061	-12.288	-7.448
H	7.358	-4.124	15.197	H	10.749	-12.035	-6.633
C	5.385	-4.179	18.068	H	9.058	-11.951	-7.157
H	5.867	-3.342	18.583	H	10.037	-13.379	-7.528
H	4.316	-4.168	18.321	C	9.5	-12.105	-9.879
H	5.797	-5.1	18.494	H	9.813	-11.797	-10.881
C	4.955	-5.377	15.897	H	9.417	-13.197	-9.888
H	3.875	-5.404	16.086	H	8.497	-11.7	-9.694

H	5.106	-5.388	14.811	C	1.086	6.099	-6.51
H	5.38	-6.3	16.304	C	0.258	6.674	-5.54
C	2.737	8.498	1.027	C	0.244	8.041	-5.278
C	2.18	8.395	-0.252	C	1.05	8.91	-6.029
C	1.535	9.458	-0.878	C	1.872	8.353	-7.017
C	1.461	10.707	-0.246	C	1.9	6.978	-7.234
C	2.029	10.833	1.028	F	-0.535	5.896	-4.791
C	2.634	9.745	1.652	F	-0.533	8.486	-4.289
F	2.221	7.227	-0.908	F	2.651	9.144	-7.763
F	0.979	9.243	-2.072	F	2.719	6.508	-8.177
F	1.989	12.008	1.666	N	1.053	10.308	-5.785
F	3.139	9.925	2.875	N	-0.074	10.912	-5.339
N	0.812	11.809	-0.863	N	0.246	12.151	-5.139
N	0.814	11.916	-2.213	C	1.558	12.395	-5.467
N	0.095	12.959	-2.484	C	2.09	11.191	-5.877
C	-0.368	13.567	-1.342	H	3.086	10.923	-6.185
C	0.089	12.811	-0.282	C	1.994	13.779	-5.263
H	-0.068	12.907	0.777	N	1.02	14.526	-4.656
C	-1.196	14.755	-1.571	C	1.263	15.821	-4.387
N	-1.447	14.939	-2.904	C	2.459	16.447	-4.708
C	-2.205	15.982	-3.287	C	3.443	15.701	-5.35
C	-2.74	16.896	-2.39	C	3.224	14.351	-5.649
C	-2.464	16.73	-1.036	H	0.465	16.364	-3.894
C	-1.675	15.659	-0.601	H	2.614	17.492	-4.461
H	-2.386	16.075	-4.351	H	4.394	16.159	-5.6
H	-3.355	17.715	-2.745	C	4.322	13.535	-6.303
H	-2.879	17.421	-0.309	O	4.564	12.393	-5.906
C	-1.422	15.46	0.881	N	4.993	14.162	-7.292
O	-1.493	14.333	1.375	H	4.66	15.062	-7.612
N	-1.154	16.587	1.574	C	6.117	13.561	-8.043
H	-1.022	17.452	1.065	H	6.26	12.579	-7.584
C	-0.899	16.625	3.03	C	5.679	13.365	-9.501
H	-1.031	15.59	3.359	H	4.768	12.758	-9.542
C	0.563	17.033	3.258	H	5.472	14.318	-10.002
H	0.82	16.994	4.319	H	6.45	12.848	-10.077
H	1.232	16.348	2.727	C	7.437	14.376	-7.834
H	0.768	18.049	2.903	C	7.782	14.398	-6.331
C	-1.971	17.494	3.77	H	7.018	14.919	-5.741
C	-1.95	18.961	3.297	H	7.883	13.383	-5.929
H	-2.185	19.057	2.228	H	8.732	14.919	-6.167
H	-2.708	19.539	3.835	C	8.583	13.668	-8.585
H	-0.985	19.445	3.48	H	8.463	13.719	-9.672
C	-1.69	17.445	5.286	H	9.539	14.143	-8.342
H	-0.765	17.964	5.555	H	8.657	12.611	-8.299

H	-2.505	17.932	5.832	C	7.31	15.823	-8.35
H	-1.622	16.412	5.648	H	7.092	15.865	-9.423
C	-3.369	16.893	3.514	H	6.531	16.388	-7.82
H	-3.642	16.925	2.453	H	8.249	16.363	-8.191
H	-3.421	15.849	3.844	C	0.46	-5.647	5.63
H	-4.13	17.457	4.065	C	0.882	-6.079	4.37
C	-7.868	0.584	-1.257	C	-0.066	-6.457	3.393
C	-7.333	1.671	-0.563	C	-1.435	-6.416	3.742
C	-7.077	1.575	0.823	C	-1.825	-6.005	5.019
C	-7.41	0.369	1.479	C	-0.893	-5.596	5.975
C	-7.97	-0.693	0.763	C	2.331	-6.103	4.094
C	-8.19	-0.613	-0.614	C	2.695	-6.519	2.726
C	-7.025	2.898	-1.324	C	1.712	-6.865	1.771
C	-6.413	3.982	-0.533	N	0.342	-6.848	2.102
C	-6.152	3.833	0.848	C	4.048	-6.52	2.377
N	-6.491	2.646	1.527	C	4.471	-6.829	1.082
C	-6.05	5.157	-1.197	C	3.497	-7.179	0.144
C	-5.397	6.2	-0.537	C	2.14	-7.214	0.471
C	-5.149	6.054	0.83	C	-0.622	-7.18	1.129
C	-5.525	4.903	1.526	C	-2.002	-7.129	1.427
C	-6.206	2.514	2.901	C	-2.491	-6.754	2.768
C	-6.5	1.317	3.591	C	1.181	-7.598	-0.584
C	-7.138	0.17	2.916	C	-0.236	-7.559	-0.177
C	-5.226	4.836	2.97	C	-1.205	-7.865	-1.137
C	-5.604	3.569	3.624	C	-2.57	-7.779	-0.856
C	-5.308	3.415	4.981	C	-2.947	-7.413	0.439
C	-5.564	2.219	5.656	O	3.168	-5.78	4.93
C	-6.168	1.183	4.941	O	-3.685	-6.705	3.045
O	-7.242	2.999	-2.527	O	1.535	-7.904	-1.717
O	-7.401	-0.876	3.5	H	1.236	-5.355	6.329
O	-4.684	5.755	3.574	H	-2.891	-5.991	5.222
H	-8.024	0.716	-2.323	H	4.752	-6.241	3.154
H	-8.199	-1.593	1.325	H	3.759	-7.44	-0.876
H	-6.273	5.206	-2.258	H	-0.843	-8.143	-2.122
H	-4.657	6.831	1.404	H	-3.99	-7.339	0.727
H	-4.841	4.26	5.476	C	-1.326	-5.074	7.298
H	-6.404	0.233	5.408	C	-0.92	-3.811	7.742
C	-8.7	-1.781	-1.38	C	-1.345	-3.266	8.95
C	-7.997	-2.284	-2.479	C	-2.18	-4.006	9.8
C	-8.416	-3.406	-3.19	C	-2.581	-5.28	9.38
C	-9.609	-4.055	-2.841	C	-2.176	-5.789	8.148
C	-10.335	-3.553	-1.754	F	-0.122	-3.056	6.974
C	-9.876	-2.454	-1.032	F	-0.956	-2.027	9.255
F	-6.85	-1.706	-2.862	F	-3.371	-6.025	10.162

F	-7.643	-3.848	-4.183	F	-2.609	-7.002	7.797
F	-11.484	-4.135	-1.39	N	-2.626	-3.477	11.039
F	-10.605	-2.037	0.006	N	-1.836	-2.613	11.72
N	-10.061	-5.198	-3.55	N	-2.525	-2.248	12.755
N	-9.773	-5.326	-4.867	C	-3.75	-2.869	12.795
N	-10.241	-6.481	-5.219	C	-3.823	-3.663	11.669
C	-10.859	-7.119	-4.171	H	-4.613	-4.287	11.292
C	-10.732	-6.289	-3.077	C	-4.588	-2.5	13.939
H	-11.038	-6.413	-2.053	N	-4.015	-1.498	14.675
C	-11.411	-8.438	-4.494	C	-4.662	-1.033	15.759
N	-11.076	-8.821	-5.765	C	-5.888	-1.532	16.177
C	-11.497	-10.016	-6.218	C	-6.462	-2.57	15.449
C	-12.27	-10.882	-5.457	C	-5.815	-3.082	14.319
C	-12.636	-10.486	-4.175	H	-4.174	-0.231	16.299
C	-12.221	-9.248	-3.672	H	-6.378	-1.117	17.052
H	-11.194	-10.279	-7.225	H	-7.426	-2.971	15.743
H	-12.575	-11.841	-5.862	C	-6.473	-4.178	13.503
H	-13.228	-11.148	-3.551	O	-6.465	-4.123	12.272
C	-12.583	-8.848	-2.255	N	-7.064	-5.155	14.224
O	-11.739	-8.325	-1.524	H	-6.936	-5.163	15.228
N	-13.849	-9.135	-1.882	C	-7.786	-6.302	13.634
H	-14.495	-9.478	-2.581	H	-7.72	-6.142	12.554
C	-14.402	-8.857	-0.54	C	-7.028	-7.588	13.991
H	-13.567	-8.423	0.017	H	-7.481	-8.457	13.508
C	-15.506	-7.799	-0.679	H	-5.99	-7.52	13.649
H	-15.89	-7.501	0.299	H	-7.017	-7.775	15.07
H	-15.109	-6.903	-1.168	C	-9.303	-6.278	14.021
H	-16.352	-8.161	-1.273	C	-9.93	-4.963	13.516
C	-14.817	-10.18	0.188	H	-9.49	-4.084	14.001
C	-15.926	-10.933	-0.574	H	-9.801	-4.848	12.433
H	-15.605	-11.239	-1.58	H	-11.005	-4.949	13.728
H	-16.192	-11.851	-0.039	C	-9.511	-6.391	15.544
H	-16.841	-10.34	-0.672	H	-9.053	-5.555	16.09
C	-13.58	-11.09	0.323	H	-10.58	-6.369	15.78
H	-13.19	-11.401	-0.653	H	-9.11	-7.326	15.951
H	-12.769	-10.585	0.862	C	-10.013	-7.456	13.322
H	-13.836	-11.999	0.877	H	-9.7	-8.426	13.719
C	-15.321	-9.831	1.604	H	-11.096	-7.38	13.47
H	-16.269	-9.283	1.586	H	-9.827	-7.451	12.241
H	-15.49	-10.749	2.176	C	5.905	-6.736	0.699
H	-14.587	-9.229	2.153	C	6.314	-5.941	-0.377
C	-4.933	7.405	-1.275	C	7.651	-5.788	-0.733
C	-3.593	7.804	-1.233	C	8.649	-6.481	-0.033
C	-3.11	8.894	-1.951	C	8.256	-7.296	1.036

C	-3.986	9.672	-2.722	C	6.916	-7.402	1.401
C	-5.335	9.299	-2.76	F	5.406	-5.255	-1.085
C	-5.788	8.179	-2.067	F	7.946	-4.956	-1.733
F	-2.708	7.101	-0.513	F	9.173	-7.982	1.728
F	-1.803	9.155	-1.898	F	6.609	-8.18	2.441
F	-6.208	10.016	-3.476	N	10.02	-6.347	-0.377
F	-7.082	7.865	-2.16	N	10.366	-6.11	-1.665
N	-3.522	10.792	-3.461	N	11.653	-5.963	-1.662
N	-2.47	11.509	-2.999	C	12.179	-6.121	-0.402
N	-2.231	12.405	-3.903	C	11.113	-6.36	0.44
C	-3.117	12.319	-4.95	H	11.069	-6.504	1.505
C	-3.953	11.258	-4.669	C	13.633	-5.965	-0.32
H	-4.759	10.822	-5.232	N	14.16	-5.558	-1.517
C	-2.905	13.301	-6.016	C	15.487	-5.357	-1.611
N	-1.767	14.032	-5.803	C	16.357	-5.553	-0.547
C	-1.42	14.975	-6.698	C	15.831	-5.993	0.663
C	-2.171	15.253	-7.831	C	14.456	-6.218	0.796
C	-3.343	14.533	-8.038	H	15.852	-5.023	-2.575
C	-3.739	13.548	-7.127	H	17.419	-5.366	-0.668
H	-0.504	15.516	-6.49	H	16.485	-6.142	1.517
H	-1.844	16.015	-8.53	C	13.888	-6.65	2.135
H	-3.944	14.718	-8.923	O	12.851	-6.137	2.561
C	-4.992	12.734	-7.389	N	14.609	-7.582	2.792
O	-4.988	11.515	-7.206	H	15.394	-8.012	2.319
N	-6.047	13.438	-7.85	C	14.252	-8.124	4.12
H	-5.984	14.447	-7.879	H	13.346	-7.582	4.404
C	-7.355	12.841	-8.195	C	13.907	-9.612	3.961
H	-7.223	11.77	-8.023	H	13.109	-9.736	3.222
C	-8.408	13.373	-7.211	H	14.769	-10.202	3.63
H	-9.376	12.895	-7.377	H	13.557	-10.037	4.905
H	-8.101	13.16	-6.182	C	15.352	-7.791	5.183
H	-8.55	14.455	-7.305	C	14.894	-8.325	6.556
C	-7.684	13.037	-9.713	H	14.877	-9.419	6.594
C	-7.788	14.527	-10.093	H	15.583	-7.986	7.337
H	-6.845	15.066	-9.927	H	13.894	-7.958	6.816
H	-8.02	14.628	-11.159	C	16.712	-8.421	4.822
H	-8.579	15.045	-9.541	H	16.664	-9.514	4.765
C	-6.58	12.369	-10.558	H	17.105	-8.045	3.867
H	-5.603	12.841	-10.405	H	17.456	-8.171	5.585
H	-6.481	11.304	-10.315	C	15.508	-6.26	5.284
H	-6.818	12.448	-11.624	H	15.863	-5.821	4.345
C	-9.025	12.339	-10.025	H	14.557	-5.778	5.542
H	-9.875	12.839	-9.551	H	16.236	-6.004	6.06
H	-9.207	12.347	-11.105	C	-3.589	-8.016	-1.911

H	-9.017	11.292	-9.699	C	-4.556	-7.05	-2.21
C	-5.159	2.036	7.075	C	-5.494	-7.214	-3.225
C	-4.336	0.972	7.458	C	-5.526	-8.402	-3.969
C	-3.893	0.796	8.765	C	-4.577	-9.388	-3.671
C	-4.307	1.68	9.773	C	-3.621	-9.186	-2.678
C	-5.147	2.741	9.411	F	-4.577	-5.894	-1.534
C	-5.544	2.921	8.088	F	-6.333	-6.208	-3.477
F	-3.908	0.094	6.54	F	-4.574	-10.542	-4.349
F	-3.057	-0.212	9.019	F	-2.732	-10.157	-2.456
F	-5.573	3.604	10.34	N	-6.473	-8.598	-5.007
F	-6.329	3.963	7.805	N	-7.693	-8.015	-4.918
N	-3.874	1.522	11.116	N	-8.302	-8.304	-6.024
N	-3.619	0.282	11.595	C	-7.526	-9.088	-6.842
N	-3.177	0.454	12.8	C	-6.326	-9.271	-6.185
C	-3.156	1.784	13.148	H	-5.429	-9.786	-6.479
C	-3.6	2.484	12.045	C	-8.15	-9.452	-8.117
H	-3.705	3.54	11.871	N	-9.35	-8.815	-8.285
C	-2.663	2.056	14.5	C	-10.052	-9.031	-9.412
N	-2.196	0.918	15.101	C	-9.619	-9.884	-10.418
C	-1.696	0.996	16.347	C	-8.414	-10.556	-10.24
C	-1.64	2.183	17.065	C	-7.66	-10.36	-9.078
C	-2.139	3.337	16.471	H	-10.988	-8.492	-9.503
C	-2.671	3.293	15.177	H	-10.214	-10.018	-11.315
H	-1.327	0.07	16.772	H	-8.043	-11.219	-11.015
H	-1.217	2.198	18.064	C	-6.324	-11.059	-8.917
H	-2.096	4.283	17.002	O	-5.351	-10.442	-8.476
C	-3.164	4.568	14.52	N	-6.299	-12.349	-9.314
O	-2.887	4.804	13.341	H	-7.169	-12.795	-9.573
N	-3.873	5.391	15.321	C	-5.096	-13.208	-9.27
H	-4.129	5.068	16.245	H	-4.307	-12.558	-8.884
C	-4.421	6.696	14.894	C	-5.344	-14.339	-8.261
H	-4.085	6.81	13.86	H	-6.161	-14.999	-8.573
C	-5.954	6.608	14.908	H	-4.45	-14.954	-8.134
H	-6.348	6.444	15.917	H	-5.602	-13.92	-7.283
H	-6.403	7.526	14.521	C	-4.671	-13.662	-10.707
H	-6.289	5.78	14.276	C	-4.374	-12.413	-11.562
C	-3.8	7.87	15.723	H	-5.265	-11.79	-11.702
C	-4.359	9.206	15.19	H	-3.597	-11.79	-11.103
H	-5.425	9.327	15.405	H	-4.022	-12.708	-12.556
H	-3.836	10.044	15.663	C	-5.765	-14.503	-11.394
H	-4.216	9.296	14.106	H	-6.7	-13.941	-11.527
C	-2.27	7.87	15.526	H	-5.436	-14.802	-12.394
H	-1.805	6.957	15.915	H	-5.993	-15.421	-10.842
H	-2.003	7.958	14.466	C	-3.378	-14.498	-10.602

H	-1.82	8.717	16.055	H	-3.543	-15.46	-10.108
C	-4.122	7.748	17.225	H	-2.989	-14.71	-11.604
H	-5.199	7.772	17.424	H	-2.596	-13.959	-10.052
H	-3.712	6.83	17.667	Fe	-0.683	13.539	-4.16
H	-3.675	8.584	17.774	Fe	-9.899	-7.537	-6.806
C	-1.3	-2.397	-7.509	Fe	12.83	-5.233	-3.015
C	-0.09	-1.719	-7.341	Fe	-2.252	-0.772	13.979

11 References

1. Xue, W.; Ronson, T. K.; Lu, Z.; Nitschke, J. R. Solvent Drives Switching between *Λ* and *Δ* Metal Center Stereochemistry of M_8L_6 Cubic Cages. *J. Am. Chem. Soc.* **2022**, *144*, 6136–6142.

2. Chou, C.-M.; Saito, S.; Yamaguchi, S. Heterotriangulenes π-Expanded at Bridging Positions. *Org. Lett.* **2014**, *16*, 2868–2871.

3. Sibi, M. P.; Petrovic, G. Enantioselective Radical Reactions: the Use of Metal Triflimides as Lewis Acids. *Tetrahedron Asymmetry* **2003**, *14*, 2879–2882.

4. Ziegler, M.; von Zelewsky, A. Charge-Transfer Excited State Properties of Chiral Transition Metal Coordination Compounds Studied by Chiroptical Spectroscopy. *Coord. Chem. Rev.* **1998**, *177*, 257–300.

5. (a) Hidaka, J.; Douglas, B. E. Circular Dichroism of Coordination Compounds. II. Some Metal Complexes of 2,2'-Dipyridyl and 1,10-Phenanthroline. *Inorg. Chem*. **1964**, *3*, 1180–1184. (b) Mason, S. F.; Peart, B. J. Optical Rotatory Power of Coordination Compounds. Part XVII. The Circular Dichroism of Trisbipyridyl and Trisphenanthroline Complexes. *J. Chem. Soc., Dalton Trans.* **1973**, 949–955.

6. Jayaratna, N. B.; Olmstead, M. M.; Kharisov, B. I.; Dias, H. V. R. Coinage Metal Pyrazolates $[(3,5\text{-}(CF_3)_2Pz)M]_3$ (M = Au, Ag, Cu) as Buckycatchers. *Inorg. Chem.* **2016**, *55*, 8277–8280.

7. Rispens, M. T.; Meetsma, A.; Rittberger, R.; Brabec, C. J.; Sariciftci, N. S.; Hummelen, J. C. Influence of the Solvent on the Crystal Structure of PCBM and the Efficiency of MDMO-PPV: PCBM 'Olastic' Solar Cells. *Chem. Commun.* **2003**, 2116–2118.

8. Feng, L.; Zhang, M.; Hao, Y.; Tang, Q.; Chen, N.; Slanina, Z.; Uhlík, F. Endohedrally Stabilized C_{70} Isomer with Fused Pentagons Characterized by Crystallography. *Dalton Trans.* **2016**, *45*, 8142–8148.

9. Umeyama, T.; Miyata, T.; Jakowetz, A. C.; Shibata, S.; Kurotobi, K.; Higashino, T.; Koganezawa, T.; Tsujimoto, M.; Gélinas, S.; Matsuda, W.; Seki, S.; Friend, R. H.; Imahori, H. Regioisomer Effects of

[70]Fullerene Mono-adduct Acceptors in Bulk Heterojunction Polymer Solar Cells. *Chem. Sci.* **2017**, *8*, 181–188.

10. Maglic, J. B.; Lavendomme, R. MoloVol: An Easy-to-Use Program for Analyzing Cavities, Volumes and Surface Areas of Chemical Structures. *J. Appl. Cryst.* **2022***, 55,* 1033–1044.

11. Sabirov, D. S.; Garipova, R. R., The Increase in the Fullerene Cage Volume upon its Chemical Functionalization. *Fuller. Nanotub. Carbon Nanostructures* **2019**, *27*, 702–709.

12. Brotin, T.; Vanthuyne, N.; Cavagnat, D.; Ducasse, L.; Buffeteau, T. Chiroptical Properties of Nona- and Dodecamethoxy Cryptophanes. *J. Org. Chem.* **2014**, *79*, 6028−6036.

13. Wang, J.; Wolf, R. M.; Caldwell, J. W.; Kollman, P. A.; Case, D. A. Development and Testing of a General Amber Force Field. *J. Comput. Chem.* **2004**, *25*, 1157–1174.

14. Bayly, C. I.; Cieplak, P.; Cornell, W.; Kollman, P. A. A Well-Behaved Electrostatic Potential Based Method Using Charge Restraints for Deriving Atomic Charges: The RESP Model. *J. Phys. Chem.* **1993**, *97*, 10269–10280.

15. *Gaussian 16, Revision C.01*; Gaussian, Inc.: Wallingford CT, 2016. https://gaussian.com/gaussian16.

16. Becke, A. D., Density-Functional Thermochemistry. III. The Role of Exact Exchange. *J. Chem. Phys.* **1993**, *98*, 5648–5652.

17. Seminario, J. M. Calculation of Intramolecular Force Fields from Second-Derivative Tensors. *Int. J. Quantum Chem.* **1996**, *60*, 1271–1277.

18. Allen, A. E. A.; Payne, M. C.; Cole, D. J. Harmonic Force Constants for Molecular Mechanics Force Fields via Hessian Matrix Projection. *J. Chem. Theory Comp.* **2018**, *14,* 274–281.

19. Ashley, D. C.; Jakubikova, E. Ray-Dutt and Bailar Twists in Fe(II)-Tris(2,2’-bipyridine): Spin States, Sterics, and Fe–N Bond Strengths. *Inorg. Chem.* **2018**, *57*, 5585-5596.

20. Pesce, L.; Pavan, G. M., Computational data supporting: "Subtle Stereochemical Effects Influence Binding and Purification Abilities of an FeII4L4 Cage", v1.0, *Zenodo* **2021**, https://zenodo.org/record/7350671.

21. Hess, B.; Kutzner, C.; van der Spoel, D.; Lindahl, E. GROMACS 4: Algorithms for Highly Efficient, Load-Balanced, and Scalable Molecular Simulation. *J. Chem. Theory Comput.* **2008**, *4*, 435–447.

22. *GROMACS Release 2020.2*; GROMACS development team: Uppsala SE, 2020. https://manual.gromacs.org/2020.2.
23. Tribello, G. A.; Bonomi, M.; Branduardi, D.; Camilloni, C.; Bussi, G. PLUMED 2: New Feathers for an Old Bird. *Comput. Phys. Commun.* **2014**, *185*, 604–613.
24. Bussi, G.; Donadio, D.; Parrinello, M. Canonical Sampling through Velocity Rescaling. *J. Chem. Phys.* **2007**, *126*, 014101.
25. Berendsen, H. J. C.; Postma, J. P. M.; van Gunsteren, W. F.; Di Nola, A.; Haak, J. R. Molecular Dynamics with Coupling to an External Bath. *J. Chem. Phys.* **1984**, *81*, 3684–3690.
26. Essmann, U.; Perera, L.; Berkowitz, M. L.; Darden, T.; Lee, H.; Pedersen, L. G. A Smooth Particle Mesh Ewald Method. *J. Chem. Phys.* **1995**, *103*, 8577–8593.
27. Hess, B.; Bekker, H.; Berendsen, H.; Fraaije, J. LINCS: A Linear Constraint Solver for Molecular Simulations. *J. Comput. Chem.* **1998**, *18*, 1463–1472.
28. Casasnovas, R.; Limongelli, V.; Tiwary, P.; Carloni, P.; Parrinello, M. Unbinding Kinetics of a p38 MAP Kinase Type II Inhibitor from Metadynamics Simulations *J. Am. Chem. Soc.* **2017**, *139*, 4780–4788.
29. Bochicchio, D.; Salvalaglio, M.; Pavan, G. M. Into the Dynamics of a Supramolecular Polymer at Submolecular Resolution. *Nat. Commun.* **2017**, *8*, 147.
30. Pesce, L.; Perego, C.; Grommet, A. B.; Klajn, R.; Pavan, G. M. Molecular Factors Controlling the Isomerization of Azobenzenes in the Cavity of a Flexible Coordination Cage *J. Am. Chem. Soc.* **2020**, *142*, 9792–9802.
31. Tiwary, P.; Parrinello, M. From Metadynamics to Dynamics. *Phys. Rev. Lett.* **2013**, *111*, 230602.
32. Salvalaglio, M.; Tiwary, P.; Parrinello, M. Assessing the Reliability of the Dynamics Reconstructed from Metadynamics. *J. Chem. Theory Comput.* **2014**, *10*, 1420–1425.
33. Laio, A.; Parrinello, M. Escaping Free-Energy Minima. *Proc. Natl. Acad. Sci. USA* **2002**, *99*, 12562–12566.
34. Barducci, A.; Bussi, G.; Parrinello, M. Well-Tempered Metadynamics: A Smoothly Converging and Tunable Free-Energy Method. *Phys. Rev. Lett.* **2008**, *100*, 020603.
35. Valsson, O.; Tiwary, P.; Parrinello, M. Enhancing Important Fluctuations: Rare Events and Metadynamics from a Conceptual Viewpoint. *Annu. Rev. Phys. Chem.* **2016**, *67*, 159–184.
36. Raymond, C. Physical Chemistry for the Biosciences. USA. *University Science Books*, **2005**, 338–

342.
